# ’Who Cares?' The experiences of caregivers of adults living with heart failure, chronic obstructive pulmonary disease and coronary artery disease: a mixed methods systematic review

**DOI:** 10.1136/bmjopen-2017-020927

**Published:** 2018-07-11

**Authors:** Miriam Catherine Noonan, Jennifer Wingham, Rod S Taylor

**Affiliations:** 1 European Centre for Environment and Human Health, University of Exeter Medical School, Knowledge Spa, Royal Cornwall Hospital, Truro, UK; 2 Royal Cornwall Hospitals NHS Trust, Research, Development and Innovation, F37, Knowledge Spa, Royal Cornwall Hospital, Truro, Cornwall & University of Exeter, Exeter, UK; 3 University of Exeter Medical School, Exeter, UK

**Keywords:** caregivers, heart failure, coronary heart disease, chronic obstructive pulmonary disease, mixed methods systematic review

## Abstract

**Objective:**

To assess the experiences of unpaid caregivers providing care to people with heart failure (HF) or chronic obstructive pulmonary disease (COPD) or coronary artery disease (CAD).

**Design** Mixed methods systematic review including qualitative and quantitative studies.

**Data sources** Databases searched: Medline Ebsco, PsycInfo, CINAHL Plus with Full Text, Embase, Web of Science, Ethos: The British Library and ProQuest. Grey literature identified using: Global Dissertations and Theses and Applied Sciences Index and hand searches and citation checking of included references. Search time frame: 1 January 1990 to 30 August 2017.

**Eligibility criteria for selecting studies:**

Inclusion was limited to English language studies in unpaid adult caregivers (>18 years), providing care for patients with HF, COPD or CAD. Studies that considered caregivers for any other diagnoses and studies undertaken in low-income and middle-income countries were excluded. Quality assessment of included studies was conducted by two authors.

**Data analysis/synthesis:**

A results-based convergent synthesis was conducted.

**Results:**

Searches returned 8026 titles and abstracts. 54 studies—21 qualitative, 32 quantitative and 1 mixed method were included. This totalled 26 453 caregivers who were primarily female (63%), with median age of 62 years. Narrative synthesis yielded six concepts related to caregiver experience: (1) mental health, (2) caregiver role, (3) lifestyle change, (4) support for caregivers, (5) knowledge and (6) relationships. There was a discordance between paradigms regarding emerging concepts. Four concepts emerged from qualitative papers which were not present in quantitative papers: (1) expert by experience, (2) vigilance, (3) shared care and (4) time.

**Conclusion:**

Caregiving is life altering and complex with significant health implications. Health professionals should support caregivers who in turn can facilitate the recipient to manage their long-term condition. Further longitudinal research exploring the evolution of caregiver experiences over time of patients with chronic cardiopulmonary conditions is required.

**Trial registration number:**

CRD42016053412

**Strengths and limitations of this study**

- This mixed methods systematic review provides the opportunity for a broadened and deeper understanding of the qualitative and quantitative literature on the experiences of unpaid caregivers’ providing care to people with heart failure, chronic obstructive pulmonary disease and coronary artery disease.
- This review provides an integration of the type and extent of caregiver’s experiences and predictors of caregiver’s experiences.
- To maximise applicability we included studies from higher income countries only.
- Quality of evidence limited by assessment of caregiver experience at single point of time and there is need for future studies that employ longitudinal or repeated measures design.

## Introduction

A caregiver is anyone providing unpaid care, to a friend or family member who is unable to care for themselves.^1^ This may be emotional support; someone to talk to, or practical support; dressing wounds, mobility assistance or medication checking.^2^ There are 43.5 million caregivers in the USA, 2.86 million in Australia and 6.5 million in the UK.^3^ Between 2001 and 2011, the number of unpaid caregivers in the UK grew at a faster rate than population growth.^4^ The annual value of unpaid care provided to an individual with a chronic illness is estimated to be £132 billion.^5^

Focus groups examining a caregivers’ life conducted by ‘The Institute of Public Care’ (2017), based at Oxford Brookes University; described caregivers as the ‘Skilled Helper’ performing a series of roles.^6^ Seltzer and Li describe a dynamic process of transitions to being a caregiver.^7^ These transitions comprise participating in the role before identifying as a caregiver, acceptance of the role, engaging in it with awareness and sometimes moving beyond the caregiving role when the patient moving to paid care settings or bereavement occurs. This process is not linear and people move through the different transitions at varying rates. Acknowledging this, it is imperative for caregivers to receive a caregiver needs assessment as legally stipulated by the 2014 Care Act.^8^ Additionally, the National Institute for Health and Care Excellence clinical guidelines for heart failure (HF) (CG108)^9^ and chronic obstructive pulmonary disease (COPD) (CG101)^10^ both recommend that family members or caregivers are provided with support and included in discussions about care.

Cardiopulmonary disease is a primary cause of illness. Cardiovascular disease is responsible for 45% deaths in Europe^11^ and one in four deaths in the USA.^12^ By 2020, COPD is projected to be in the global top five of diagnoses causing years lost through early mortality or disability-adjusted life years.^13^ Caregivers of patients with HF have a multitude of unmet needs due to fluctuations in the trajectory of HF.^14^ COPD has frequent unplanned hospital admissions and a high morbidity rate.^15^ Caregivers experience depressed mood, greater anxiety and increased subjective burden when their support needs are not met.^16 17^ The unpredictability of HF and COPD leads to caregivers constantly adjusting their role, creating a need to continuously reassess what caregiver needs are.^18 19^ Spousal caregivers of patients with myocardial infarction experience increased levels of stress, lifestyle impact and emotional distress.^20^ Caring for coronary artery bypass graft patients in tasks such as monitoring and provision of emotional support increased caregiver burden to a level described as moderate.^21^ COPD and cardiovascular disease are both increasing in prevalence and frequently coexist.^22 23^ We know of no systematic review that synthesises quantitative and qualitative studies to combine caregivers’ experiences of people with HF, COPD or coronary artery disease (CAD).

Using a mixed methods systematic review methodology including both qualitative and quantitative literature, this study aims to understand the experiences of adult caregivers when supporting people with HF, COPD or CAD.

## Methods

We conducted and reported this systematic review in accordance with the Preferred Reporting Items for Systematic Reviews and Meta-Analyses (PRISMA) statement.^24^

### Patient and public involvement

There was no patient and/or public involvement in this systematic review.

### Study design

This study employed a mixed methods systematic review assessing both qualitative and quantitative studies.^25^ The rationale for using a mixed methods review approach was multifaceted. First, to gain a qualitative assessment of the type and extent of caregiver’s experiences. Second, to assess the quantitative predictors of caregiver’s experiences. Third, to develop a holistic perspective of what caregiver experiences. Finally, we wanted to assess the degree of convergence between qualitative and quantitative experiences.

### Search strategy

Our search strategy was designed in conjunction with a Health Services Librarian and Information Specialists. Search terms included condition-specific terms, that is, ‘heart failure’, ‘COPD’ and ‘coronary artery disease’, caregiver-specific, plus experience related terms, ‘experience’, ‘quality of life’ ‘activities of daily living’, ‘occupational engagement’, ‘time use’, ‘self-efficacy’, ‘coping strategies’, ‘leisure activity’, ‘information exchange’ and ‘caregiver expectation’ (see online supplementary file 1, table 1 for complete list of search terms). Databases searched included: Medline Ebsco, PsycInfo, CINAHL Plus with Full Text, Embase, Web of Science, Ethos: The British Library and ProQuest. Grey literature was identified using Global Dissertations and Theses and Applied Sciences Index and hand searches and citation checking of included references. To ensure the contemporary nature of the evidence considered, the search time frame was January 1990 to August 2017. A single researcher (MN) initially screened titles and abstracts. Selection of full papers was performed by two researchers (MN and either JW or RST) and cross-checked with the eligibility criteria.

10.1136/bmjopen-2017-020927.supp1 Supplementary file 1

**Table 1:** Narrative formation (Chung, 2016)

Profile	Explanation of profile	Modal narrative extracted from study	Emerging from study	Concept
Modal	This is a narrative description of the group being studied	102 dyads, predominantly spousal caregivers. Group is caregivers of patients with HF. Comparing depressed and non-depressed patients with HF.	This study considers whether caregiver experiences are different for caregivers when caring for depressed or non-depressed patients Spouse as a caregiver. Impact of depression on the caregiver.	
Average	This is a detailed narrative description based on the mean (average) attributes of the individuals/situations being studied	Caregiver mean age 56.7, 78% female, white (94%). 41% of patients New York Heart Association III–IV. '42% caregivers reported severe burden (the Zarit Burden Interview (ZBI)≥17)'.^36^	Caregiving resulted in burden experienced by caregivers in this study.	Mental health (burden)
Comparative	This is a description comparing studies and comparing individuals being studied	Caregivers who provided care for depressed patients reported higher burden than those caring for non-depressed patients. Caregivers related their burden to social life limitations, poor perceived control, stress of family obligations and patients’ dependency. ’Caregivers of patients with depressive symptoms had a higher level of burden (25±13 vs 13.5±12 on the ZBI; p<0.001), spent more time caregiving (37±12 vs 30±11 on the Oberst Caregiving Burden Scale; p=0.004) and reported worse mental quality of life (46±10 vs 51±10 on the SF-12v2; p=0.026) than those of patients without depressive symptoms'.^36^	Patient illness severity impacts on caregiver. Life changes negatively impacted on caregivers. Depressive symptoms of patients are associated with poor outcomes of caregivers. Caregiver’s subjective and objective response to the patient’s illness severity.	Lifestyle adjustment Mental health
Normative	A comparison of the study individuals with the general population	27% of patients in this study scored 14 or higher on Becks Depression Index (a score of 14 or higher is clinically significant for depression). Family members caring for patients with HF with depressive symptoms had significantly higher levels of caregiving burden and worse quality of life compared with those caring for patients without depressive symptoms. ’Most difficult task for both sets of caregivers—providing emotional support (M=3.3, SD=1.2)'.^36^	Greater impact on caregivers' lives when patients were depressed.	Mental health Role of caregivers
Holistic (also called inferential or summative)	A description of the overall perception of the investigator		Female, white caregivers, experienced greater levels of burden, loss of roles and greater distress when patients were depressed. This could be due to the increased need for practical and emotional support, feeling they need to be constantly present.	

### Study selection

Studies were included if they addressed ‘caregiver experience’, which was defined as encompassing the daily activities of caregivers and the impact of these activities on their lives. These were English language studies involving unpaid adult caregivers (aged >18 years), providing care for patients with HF, COPD or CAD living in the community and not residential settings with paid care staff. Qualitative, quantitative and grey literature studies were all included in the search strategy. Conference papers were excluded. Outcomes of interest included psychological and physical outcomes reported, occupational engagement and routine. As we sought to inform the practice of the UK and other high-income countries, we excluded studies undertaken in low-income and middle-income countries.^26^

### Data extraction

Data extracted from retained studies included: study design, sample and recruitment, study description, method, findings, discussion and authors' conclusions and limitations. Caregiver quotes were extracted from qualitative studies. For quantitative studies, data extraction also included details of attrition and data analysis.

### Study quality assessment

Qualitative studies were appraised using the Critical Appraisal Tool.^27^ In absence of an existing quality tool that could be used to appraise quantitative studies addressing the specific question of this study, a quality assessment tool was developed by the research team based on what were deemed to be the appropriate core biases, that is, (1) was the study design longitudinal (score of 1) or cross-sectional (score of 0); (2) how was the sample recruited? Purposive (score of 1) or convenience (score of 0); (3) was the level of attrition/response rate acceptable? Attrition of 20%/lower or response rate of 80% or above (score of 1) or attrition of >20% or response rate <80% (score of 0); (4) was a validated quantitative outcome(s) used? Validated (score of 1), non-validated (score of 0); (5) were the methods of data analysis appropriate? Multivariate (score of 1) or univariate (score of 0). Based on their quality assessment, scores were totalled and studies were ranked: 1 or 2 ‘low quality’, 3 ‘medium quality’ and 4 or 5 ‘high quality’. Data extraction and quality appraisal was first conducted by a single researcher (MN) and checked by one of two researchers (JW or RST).

### Data analysis and synthesis

The methodology of mixed methods data synthesis is an emerging one and no single approach has yet been universally accepted.^28^ In this study, a results-based convergent design was chosen.^29 30^ This requires transformation of one method into another. Due to the heterogeneity of the quantitative methods, a meta-analysis was not appropriate. Instead, applying a narrative profile formation, quantitative data were converted into qualitative data.^31^ Extracted data from quantitative and qualitative studies were imported into separate spreadsheets. A meta-ethnographic approach was used to synthesise qualitative studies.^32^ A narrative formation approach^33^ was used to synthesise the quantitative data into a qualitative data set. Narrative formation is a verbal description via the use of profiles of each of the studies. The five profiles are modal, average, holistic, comparative and normative.^33^ Table 1 provides an example of this approach. This resulted in two qualitative data sets^34^ from which concepts emerged. A mapping table was completed in order to provide an audit trail of how the overall concepts across all papers were derived (see online supplementary file 1, table 2a, b and c). Initial synthesis was conducted by a single researcher (MN) and corroborated by two experienced researchers in quantitative (RST) and qualitative (JW) research.

## Results

### Study selection

**Figure 1:**
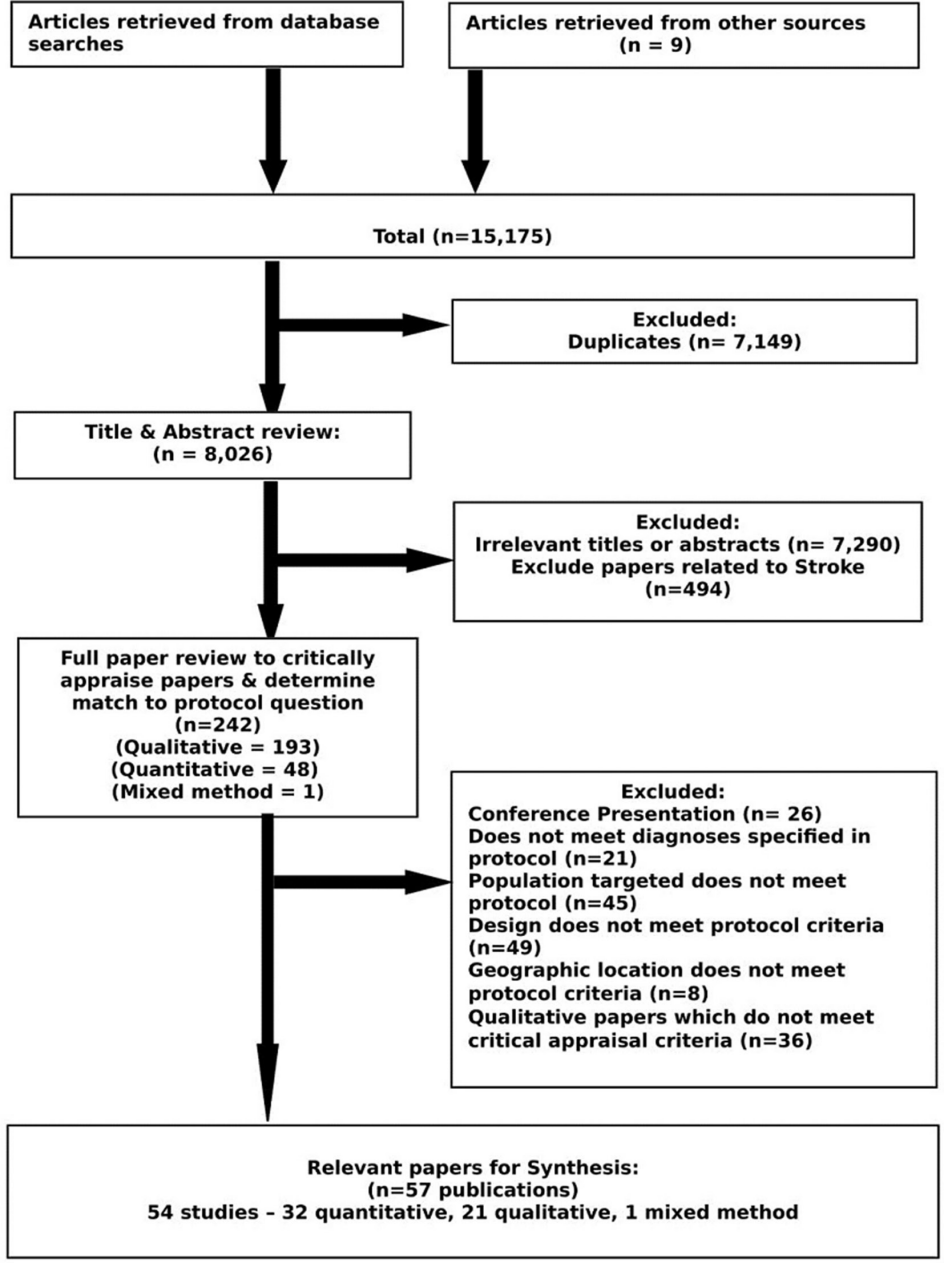
Preferred Reporting Items for Systematic Reviews and Meta-Analyses flow diagram.

Study selection process is summarised in a PRISMA flow diagram shown in figure 1. Following removal of duplicates, the search strategy yielded a total of 8026 titles and abstracts. Of these, 242 full papers were reviewed, of which 57 papers (54 studies) were included for synthesis. A detailed summary of included studies is provided in table 2. A comprehensive outline of study results and concepts generated by each study is included in online supplementary file 2).

10.1136/bmjopen-2017-020927.supp2 Supplementary file 2

**Table 2:** Summary of included studies

First author/ref. no.	Diagnosis	Aims (as stated by authors)	Methods	Country	Data collection sampling	Participants caregiver (time caring)	Mean age (caregiver/recipient)
Ågren^37^	HF (NYHA II–IV)	Describe the levels and identify independent predictors of cg burden in partners of pts with HF.	QUANT (correlational)	Sweden	Cross-sectional purposive	135 (101 F, 34 M) (NS)	69/71
Al-Rawashdeh^38^	HF (NYHA I–IV)	To examine whether individuals’ disturbance predicted their own and their partners’ QoL in HF	QUANT	USA	Cross-sectional purposive	78 dyads (58 F, 20 M) (NS)	62.2/59.5
Andersen^63^	HF	Obtain knowledge on experiences and views and the desire for knowledge of family cgs of pts with HF, their competence and support required	QUAL	Norway	Interviews convenience	19 (17 F, 2 M) (NS)	63/NS
Badr *et al*^39^	COPD	Individual-level predictors of pt and and cgs depression in COPD as well as how dyad members effect each other’s depression	QUANT	USA	Cohort study purposive	89 (68 F, 21 M) (NS)	54.8/67
Bakas *et al*^40^	HF (NYHA II–IV)	Examine relationships among age, perceived control over managing HF perceived difficulty with tasks, perceived outcomes and perceived mental and general health among cgs of persons with HF	QUANT (descriptive correlational)	USA	Cross-sectional convenience	21 (20 F, 1 M) (NS)	59.6/62.7
Baker *et al*^64^	HF (LVAD in situ)	To describe experiences of cgs of pts who received LVAD therapy as a bridge to transplantation	QUAL (phenomenological) (descriptive)	USA	Interviews convenience	6 (5 F, 1 M) (26–372 days)	51/NS
Bove *et al*^65^	COPD (GOLD C&D)	Explore how spouses of pts with severe COPD experience their role	QUAL	Denmark	Focus groups purposive	22 (13 F, 9 M) (NS)	69.4/NS
Burke *et al*^66^	HF (NYHA II–IV)	Understand what roles cgs perceive and desire for themselves, and to compare and contrast these roles with those they perceive to be desired by the healthcare system	QUAL (inductive)	USA	Interviews purposive	20 (18 F, 1 M, 1 NS) (<1to >8 hours/week)	59/64
Chung *et al*^36^	HF (NYHA II– -IV)	Examine differences in cg outcomes between cgs who care for pts with HF with and without depressive symptoms	QUANT	USA	Cross-sectional convenience	102 dyads (79 F, 23 M) (NS)	56.7/61.4
Clark *et al*^85^	HF (NYHA II–IV)	To examine the complexity of caregiving for pts with HF	QUAL	Scotland	Interviews convenience	30 (23 F, 7 M) (NS)	68 F, 67 M/NS
Cossette^41^	COPD (GOLD III–V)	Examine relationship between type, number and disturbance of caring tasks and impact on mental health of cgs. Examine influence of social support	QUANT	Canada	Cross-sectional convenience	89 (F) (mean=13 years)	65/68.6
Evangelista *et al*^42^	HF (NYHA I–IV)	Describe emotional well-being of (descriptive) pts with HF and cgs (correlational) Identify factors associated with emotional well-being of pts. Determine gender differences in emotional well-being of pts and cgs	QUANT (descriptive) (correlational)	USA	Cross-sectional convenience	103 dyads (73 F, 30 M) (NS)	59.4/57.6
Figueiredo *et al*^43^	COPD (GOLD I–IV)	Examine coping strategies of family cgs of pts with early and advanced COPD. To analyse subjective burden of family cgs of pts with early and advanced COPD and its predictor variables and how those relate to their subjective health	QUANT (correlational)	Portugal	Cross-sectional convenience	158 (120 F, 38 M) (>4 years)	58.4 (early COPD) 60.8 (advanced COPD)69.4
Figueiredo *et al*^44^	COPD (GOLD I–IV)	To analyse subjective burden of family cgs of pts with early and advanced COPD and its predictor variables	QUANT (correlational)	Portugal	Cross-sectional convenience	167 (125 F, 42 M) (>4 years)	58.3 (early COPD) 60.5 (advanced COPD)/69.3
Figueiredo *et al*^67^	COPD (moderate to severe)	Obtain knowledge on experience of husbands and sons providing care to a family member	QUAL	Portugal	Interviews purposive	12 (M) (>4 years)	70.9 (husbands) 43.4 (sons)/72.1
Grigorovich *et al*^45^	HF (NYHA II–IV)	To examine changes in cg’s well-being over time. Identify pt and cg factors associated with positive and negative outcomes	QUANT (repeated measures)	Canada	Longitudinal convenience	50 (31 F, 19 M) (mean=18 months)	58/61.6
Halm *et al*^46^ ^47^*	CAD	To determine cg burden after CABG surgery	QUANT	USA	Cross-sectional convenience	166 (136 F, 30 M) (≤12 months)	64.7/66.8
Halm^68^ ^69^*	CAD	To describe the concerns needs, strategies and advice of CABG cgs during the first 3 months postsurgery. To explore cg burden by age and gender	QUAL	USA	Interviews purposive	32 (16 F, 16 M)(NS)	60.6 (M<70)/60.1 61.5 (F<70)/62.5 75.9 (M>70)/74.4 73.6 (F>70)/77.6
Hess^86^	HF	To examine the association between cg literacy and medication administration	QUANT (correlational)	USA	Cross-sectional convenience	5 (F) (NS)	65/72.8
Hooley^48^	HF (NYHA III or IV)	To explore if greater cg burden is associated with increasing disease burden and depressive symptoms in pts and cg	QUANT	Canada	Cohort study convenience	50 (40 F, 10 M) (NS)	61/72
Hwang *et al*^49^	HF (NYHA I–IV)	To identify factors associated with the impact of caregiving	QUANT	USA	Cross-sectional convenience	76 dyads (54 F, 22 M) (mean=53.4 months)	53.4/53.8
Hynes^70^	COPD	To explore the experiences of cgs providing care to a family member with COPD	QUAL (phenomenological)	Ireland	Interviews convenience	11 (9 F, 2 M) (1–15 years)	NS/NS
Imes *et al*^71^	HF (NYHA III–IV)	To describe cg’s experience of living with HF	QUAL (descriptive)	USA	Interviews convenience	14 (11 F, 3 M) (NS)	64.8/68
Karmilovich^50^	HF (NYHA III or IV)	To examine cg demands and components of caring. Assess stress and correlation with cg burden.	QUANT (correlational)	USA	Descriptive survey purposive	41 (30 F, 11 M) (NS)	56.7/NS
Kitko^72^	HF	To gain a deeper understanding of the type of work in spousal caregiving	QUAL	USA	Interviews convenience	20 (14 F, 6 M) (2 months–9 years	67/70
Kneeshaw^87^	CAD	To examine cg mutuality and preparedness for caring post-CABG surgery	QUANT	USA	Longitudinal convenience	49 (32 F, 17 M) (NS)	50.1/72.6
Liljeroos *et al*^73^	HF	To understand perceived caring needs in dyads and understand areas of support for cgs	QUAL	Sweden	Focus groups convenience	19 dyads (NS) (7 F, 12 M)	70/72
Lindqvist *et al*^74^	COPD (mild to severe)	To describe conceptions of daily life for women caring for men with COPD	QUAL (phenomenological)	Sweden	Interviews purposive	21(F) (NS)	72/NS
Loftus^51^	HF (NYHA II–IV)	Investigate outcomes of caregiving in late-stage HF	QUANT (correlational)	UK	Longitudinal convenience	53 (41 F, 12 M) (6.66 hours/day)	66.7/76.3
Lum *et al*^91^	HF (NYHA II–IV)	Measure of relationship quality and cg benefit, burden and depressive	QUANT (correlational)	USA	Cross-sectional purposive	19 (7 F, 12 M) (<1 to >8 hours/week)	59/69
Luttik *et al*^75^	HF	Experience and needs of cg’s well-being factors	QUAL	The Netherlands	Interviews convenience	13 (10 F, 3 M) (NS)	66/88.6
Luttik *et al*^92^	HF (NYHA II–IV)	Investigate QoL in cgs of pts with HF vs QoL in people with healthy partners	QUANT	The Netherlands	Cross-sectional purposive	303 (NS)	67/69
Marcuccilli^76^	HF-LVAD in situ	Explore life adjustments of cgs caring for long-term LVAD pts.	QUAL (phenomenological)	USA	Interviews convenience	5 (5 F) (NS)	56.6/NS
Marcuccilli *et al*^77^	HF-LVAD as DT	Explore experience of caring for family member with HF	QUAL (phenomenological)	USA	Interviews purposive	7 (6 F, 1 M) (18–24 hours/day)	65/NS
Miravitlles *et al*^90^	COPD	Analyse burden of cgs	QUANT	Spain	Survey representative (mean=12.7 hours daily, severe COPD)	22 795	56.5/72
Nakken *et al*^52^	COPD	Investigate differences in male and female cgs and their perception of pts' symptoms	QUANT (correlational)	The Netherlands	Cross-sectional c onvenience	188 dyads (103 F, 85 M) (NS)	65.4/63.3 F 65.1/68.7 M
Nӓsstrӧm *et al*^84^	HF	Cg’s participation and perspective of home care services	Mixed methods	Sweden	Interviews purposive	15 (11 F, 4 M) (NS)	77/NS
Park *et al*^88^	CAD	Difficulty and demands of cg tasks for older cgs of CABG pts	QUANT	USA	Cross-sectional convenience	35 (29 F, 6 M) (mean=19 days)	60/NS
Pattenden^78^	HF	Explore how pts and cgs cope with daily life with HF	QUAL	UK	Interviews purposive	20 (18 F, 2 M) (NS)	67.8/NS
Yeh^53^	HF	Explore burden on family cgs of older pts with HF	QUANT (correlational)	USA	Cross-sectional purposive	50 (35 F, 1 5M) (<6 months to >1 year)	60.3/77.6
Pressler *et al*^54^	HF (NYHA I–IV)	Examine changes in cg burden and HRQoL. Determine different perceptions between cg’s of pts. with HF andto estimate time spent on cg tasks	QUANT (repeated measures)	USA	Longitudinal convenience	65 (48 F, 17 M) (mean=9.3 years)	59.7/69
Riegner^89^	COPD	To understand QoL and its association with role strain, humour and support in cgs and pts.	QUANT (correlational)	USA	Cross-sectional convenience	83 dyads (50 F, 33 M) (NS)	63.2/65.6
Rolley *et al*^79^	CAD	Describe experience of cgs of pts undergoing percutaneous coronary intervention	QUAL	Australia	Focus groups convenience	18 (F) (NS)	NS/NS
Saunders^55 56^	HF (NYHA I–IV)	To determine indicators of cg HRQoL	QUANT (correlational)	USA	Cross-sectional purposive	50 (42 F, 8 M) (mean=5.9 years)	58.1/77.6
Saunders^57^	HF	Compare employed and unemployed cgs on depression and well-being	QUANT	USA	Cross-sectional convenience	41 (37 F, 4 M) (2.9–6 years)	59 (unemployed)/78 52 (employed)/77
Schwarz^58^	HF	Evaluate support on stress for cgs	QUANT	USA	Cross-sectional convenience	75 (55 F, 20 M) (mean=6 years)	63/NS
Scott^59^	HF	HRQoL of cgs and pts receiving community-based inotropic infusions	QUANT	USA	Cross-sectional purposive	18 (16 F, 2 M)	63/69.3
Spence *et al*^80^	COPD (advanced)	Needs and experiences of family cgs	QUAL (descriptive)	Northern Ireland	Interviews purposive	7 (6 F, 1 M) (1–4 years)	NS/NS
StrØm (2015)	HF	Next of kin’s experience and responsibilities when caring	QUAL	Norway	Interviews convenience	19 (17 F, 2 M) (NS)	Median 63/NS
Takata *et al*^60^	COPD	Explore cg burden (long-term O2 therapy)	QUANT	Japan	Cross-sectional convenience	45 dyads (37 F, 8 M) (NS)	68/75.2
Vellone *et al*^61^	HF (NYHA I–IV)	Examine cg self-efficacy and contribution to pt self-care	QUANT (correlation)	Italy	Cross-sectional convenience	515 dyads (270 F, 245 M)	56.6/75.6
Wallin *et al*^82^	CAD	To describe cg’s need for support and impact after a cardiac event	QUAL (descriptive)	Sweden	Interviews purposive	20 (14 F, 6 M) (NS)	55/NS
Woolfe^62^	COPD	Identify needs of cgs and how this impacts cg well-being	QUANT (descriptive)	Australia	Cross-sectional convenience	63 (39 F, 24 M) (NS)	NS/NS
Wingham *et al*^83^	HF	Identify needs of cgs to inform development of a caregiver resource for use in a home- based self-management intervention	QUAL	UK	Interviews (I) focus groups (FG) (purposive)	22 (16 F, 6 M) 6 months–8 years)	(I) 67/NS (FG) 62/NS

*Same study.

ADL, activities of daily living; CABG, coronary artery bypass graft; CAD, coronary artery disease; cg, caregiver; COPD, chronic obstructive pulmonary disease; HF, heart failure; pt, patient; HRQoL, health-related quality of life; LVAD DT, left ventricular assist device destination therapy; LVAD, left ventricular assist device; NS, not stated; QoL, quality of life; NYHA, New York Heart Association; GOLD, Global Inititative for Chronic Lung Disease, QUANT, quantitative; QUAL, qualitative.

### Study characteristics

Of the 54 studies, 21 were qualitative, 32 quantitative and 1 mixed methods. Thirty-four focused on HF, 14 COPD and 6 CAD. The total number of caregiver participants was 26 453. Caregivers were primarily female (63%), with a median age of 62 years. Patient median age was 69 years. A summary of study characteristics is provided in table 3.

**Table 3:** Study characteristics

Summary of study characteristics	n=54 studies	
Aetiology of patients		
CAD, n (%)	6 (11)	
HF, n (%)	34 (63)	
COPD, n (%)	14 (26)	
Caregiver participants*		Patients†
Median age (range)	62 (43–77)	69 (36–93)
Median % of female (range)	63% (5–270)	38% (1–229)
Relationship between patient and caregiver (n=26 008 caregivers)		
Spousal/partner, n (%)	2321 (9)	
Son/daughter, n (%)	610 (2)	
Sibling, n (%)	22 (<1)	
Parent, n (%)	10 (<1)	
Friend/relative, n (%)	228 (<1)	
Not stated	22 961 (88)	
Type of study		
Qualitative, n (%)	21 (39)	
Quantitative, n (%)	32 (59)	
Mixed	1 (2)	
Study design		
Cross-sectional, n (%)	24 (44)	
Longitudinal, n (%)	4 (7)	
Cohort, n (%)	2 (4)	
Quantitative (survey), n (%)	2 (4)	
Qualitative (interview/focus group), n (%)	16 (31)	
Phenomenological, n (%)	5 (8)	
Inductive, n (%)	1 (2)	
Continents of publication		
Europe	22 (41)	
North America, n (%)	29 (54)	
Australasia, n (%)	3 (5)	
Date of publication	n=57 publications‡	
1990–1995	2	
1996–2001	3	
2002–2007	10	
2008–2013	22	
2014–2017	20	

*Caregiver data based on data collected in 50 studies.

†Patient data based on data collected in 35 studies.

‡There were 57 publications, however there were 54 studies. The following studies used the same data but produced two publications: Halm, 2006 and 2007, Saunders, 2008 and 2009, Halm 2016 and 2017.

CAD, coronary artery disease; COPD, chronic obstructive pulmonary disease; HF, heart failure.

### Quality assessment

Studies of insufficient quality were excluded, qualitative papers were appraised and only high-quality qualitative studies were included.^35^ A total of 21 out of 193 qualitative studies were classified as high quality. Quantitative studies were classified as follows: 3 high quality, 12 medium quality and 17 low quality (see table 4(a) and (b) for quality appraisal). Given the number of high-quality qualitative studies and in accord with current guidelines for the synthesis of qualitative evidence, we limited inclusion to qualitative studies of high quality only.^35^ In contrast, given the low number of high-quality quantitative studies, to ensure comprehensiveness of our review, we included all quantitative studies, regardless of quality.

**Table 4:** (a) Quality appraisal—qualitative papers

First author/ref. no.	Design	Recruitment	Data collection	Data analysis	Findings	Total
Andersen^63^	1	1	1	0	1	4 (H)
Baker *et al*^64^	1	0	1	1	1	4 (H)
Bove *et al*^65^	1	1	1	1	1	5 (H)
Burke *et al*^66^	1	1	1	1	1	5 (H)
Clark *et al*^85^	1	1	1	1	0	4 (H)
Figueiredo *et al*^67^	0	1	1	1	1	4 (H)
Halm^68^*	1	1	1	1	1	5 (H)
Halm^69^*	1	1	1	1	1	5 (H)
Hynes^70^	1	1	1	0	1	4 (H)
Imes *et al*^71^	1	1	1	1	1	5 (H)
Kitko^72^	1	1	1	1	1	5 (H)
Liljeroos *et al*^73^	1	1	1	1	1	5 (H)
Lindqvist *et al*^74^	1	1	1	1	1	5 (H)
Luttik *et al*^75^	1	1	1	1	1	5 (H)
Marcuccilli^76^	1	0	1	1	1	4 (H)
Marcuccilli^77^	1	1	1	1	1	5 (H)
Nӓsstrӧm *et al*^84^†	1	1	1	1	1	5 (H)
Pattenden^78^	1	1	1	1	0	4 (H)
Rolley *et al*^79^	1	1	1	1	0	4 (H)
Spence *et al*^80^	1	0	1	1	1	4 (H)
Strøm (2015)	1	1	1	1	1	5 (H)
Wallin *et al*^82^	1	0	1	1	1	4 (H)
Wingham *et al*^83^	1	1	1	1	1	5 (H)

*Same study.

†Mixed methods study—qualitative component.

H, high quality, 4/ 5 out of 5 quality criteria achieved.

**Table 4:** (b) Quality appraisal—quantitative papers

First author/ref. no.	Study design	Participant sampling	Participant attrition	Outcome measures	Data analysis	Overall score
Ågren^37^	CS	Purp (+1)	0% (+1)	Non-V	MV (+1)	3 (M)
Al-Rawashdeh^38^	CS	Purp (+1)	NS	V (+1)	MV (+1)	3 (M)
Badr *et al*^39^	CS	Con	15.5% (+1)	Non-V	MV (+1)	2 (L)
Bakas *et al*^40^	CS	Con	NS	V (+1)	MV (+1)	2 (L)
Chung *et al*^36^	CS	Con	NS	V (+1)	UV	1 (L)
Cossette^41^	CS	Con	NS	V (+1)	MV (+1)	2 (L)
Evangelista *et al*^42^	CS	Con	20% (+1)	V (+1)	MV (+1)	3 (M)
Figueiredo *et al*^43^	CS	Con	17% (+1)	V (+1)	MV (+1)	3 (M)
Figueiredo *et al*^44^	CS	Con	11% (+1)	Non-V	MV (+1)	2 (L)
Grigorovich *et al*^45^	LS (+1)	Con	NS	V (+1)	MV (+1)	3 (M)
Halm *et al*^46^*	CS	Con	64%	V (+1)	MV (+1)	2 (L)
Halm^47^*	CS	Con	64%	V (+1)	MV (+1)	2 (L)
Hess^86^	CS	Con	NS	V (+1)	MV (+1)	2 (L)
Hooley^48^	CS	Con	0% (+1)	V (+1)	UV	2 (L)
Hwang *et al*^49^	CS	Con	35%	V (+1)	MV (+1)	2 (L)
Karmilovich^50^	CS	Purp (+1)	24%	V (+1)	MV (+1)	3 (M)
Kneeshaw^87^	LS (+1)	Con	32.70%	V (+1)	MV (+1)	3 (M)
Loftus^51^	LS (+1)	Con	36%	V (+1)	MV (+1)	3 (M)
Lum *et al*^91^	CS	Purp (+1)	5% (+1)	V (+1)	MV (+1)	4 (H)
Luttik *et al*^92^	CS	Purp (+1)	31%	Non-V	MV (+1)	3 (M)
Miravitlles *et al*^90^	CS	Rand (+1)	0% [+1]	Non-V	MV (+1)	3 (M)
Nakken *et al*^52^	CS	Con	58%	Non-V	MV (+1)	1 (L)
Nӓsstrӧm *et al*^84^*	LS (+1)	Purp (+1)	7% (+1)	V (+1)	MV (+1)	5 (H)
Park *et al*^88^	CS	Con	NS	V (+1)	UV	1 (L)
Yeh^53^	CS	Purp (+1)	39%	V (+1)	MV (+1)	4 (H)
Pressler *et al*^54^	LS (+1)	Con	16% (+1)	V (+1)	MV (+1)	4 (H)
Riegner^89^	CS	Con	71.80%	V (+1)	MV (+1)	2 (L)
Saunders^55^†	CS	Purp (+1)	36%	V (+1)	MV (+1)	3 (M)
Saunders^56^†	CS	Purp (+1)	36%	V (+1)	MV (+1)	3 (M)
Saunders^57^	CS	Con	NS	V (+1)	UV	1 (L)
Schwarz^58^	CS	Con	NS	V (+1)	MV (+1)	2 (L)
Scott^59^	CS	Purp (+1)	10% (+1)	Non-V	MV (+1)	3 (M)
Takata *et al*^60^	CS	Con	NS	V (+1)	UV	1 (L)
Vellone *et al*^61^	CS	Con	NS	V (+1)	MV (+1)	2 (L)
Woolfe^62^	CS	Con	37%	V (+1)	UV	1 (L)

Studt design: CS, LS.

Participant sampling: Purp, Rand, Cons, Con, NS.

Attrition: 20% or less=+1; NS.

Outcome measures: V, non-V, NS.

Data analysis: MV, UV.

*Same study.

†Mixed methods study—quantitative component.

CS, cross-sectional design; Cons, consecutive; Con, convenience; H, high quality, 4/ 5 out of 5 quality criteria achieved; L, low quality, 1 or 2 out of 5 quality criteria achieved; LS, longitudinal design; M, medium quality, 3 out of 5 quality criteria achieved; MV, multivariate; non-V, some or all non-validated; NS, not stated/unclear; Purp, purposive; Rand: random; V, all validated outcomes; UV, univariate.

### Findings

**Figure 2:**
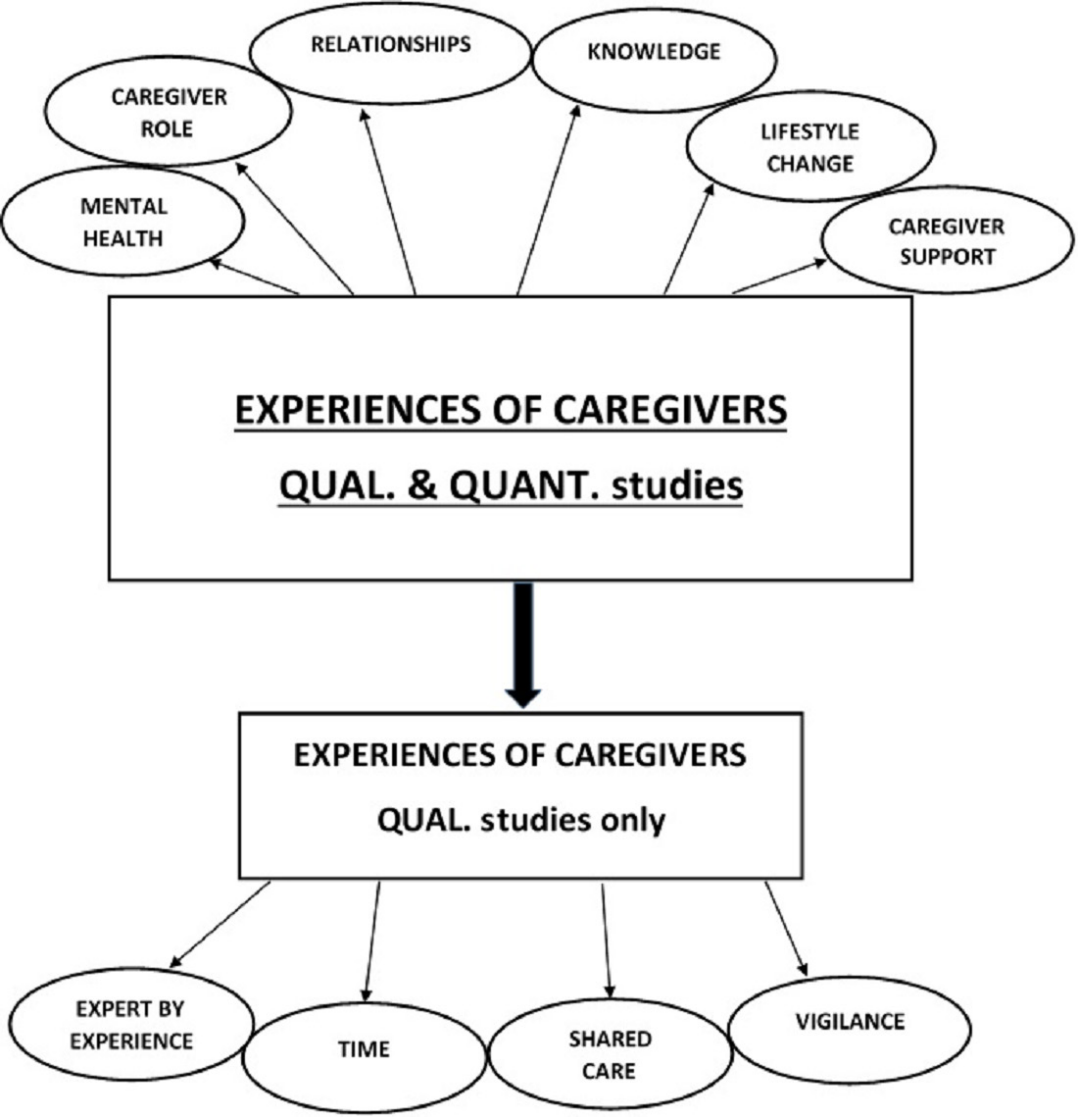
Conceptual experiences of caregivers.

Six concepts relating to caregiver experience were identified: (1) mental health, (2) caregiver role, (3) lifestyle change, (4) support for caregivers, (5) knowledge and (6) relationships. Four additional concepts were identified from qualitative papers only (6) expert by experience, (7) vigilance, (8) time and (9) shared-care (figure 2). The concepts are reflected in caregiver quotes in table 5.

**Table 5:** Illustrative quotes of caregiver experience—by concept

Mental health	*“The mental strain is difficult. I feel so trapped”*. *“You fall into a huge hole, then the world gets so tiny, it all gets sonarrow that it is almost unbearable”*. *“I feel like sleeping beauty. The hawthorn hedge has closed around me, and I cannot do anything about it.”*.
Role	*“I can sum my role up in three words, I am a cheerleader, drill sergeant, and negotiator”*.
Lifestyle change	*“Our life has come down. The two of us used to go out dancing. We loved dancing and then it all stopped"*.
Knowledge	*“I wish I had had more education on the ‘what ifs’. When I was leaving the hospital nobody really said, ’OK now this is what’s going to happen and this is what you’re going to have to do'. If there would’ve been any kind of complications I would’ve been totally in the dark. I didn’t know all the things I needed to know"*.
Relationships	*“I just love him and I find that every day when I see him, what else could I do to try and make him a wee bit … better? It’s very satisfying to know that he appreciates what I do and it’s nice to know that you are helping someone”.* *“It’s like having another child sometimes because you are sort of responsible and I feel he is my responsibility. I feel that he is not anybody else’s responsibility…”*
Support	*“And then I really felt alone in it all. Because everybody would call and come over and ask, how is John? Hardly anyone asked 'how are you doing'"?* *“Doctors (do) not realize that 1 day your life is jut normal and then this comes and smashes everything to bits, you know and there are so many questions"*. *“I would be lost without, our heart failure nurse, and, all the other input we’ve had from all the other professionals, like the podiatrist and GP… You can do it, but in partnership with everybody else"*.
Vigilance	*“Every morning I put my ear to his chest and listen to his heart, that is how we first discovered he was in atrial fibrillation so now I do it every morning before I leave. I monitor him very closely and there are days in which I do not feel comfortable leaving for work so those days I work at home. I call everyday from work and we have our routine, if I am not aware of anything he had planned for the day, I then immediately call my neighbour to check on him”.*
Shared care	*“There were days I thought to myself, where are we going from here? But we mastered it together and tried to do things at his pace"*.
Time	*“At first it was overwhelming. I didn’t think I could do it. When they first told me I was like, 'I can’t do that', you know. And then they explained to me, like, yes you can. It’s like getting a new baby. You know, you learn how to take care of them step by step and then it’s just part of the routine. And that’s really the way it was"*.
Expert by experience	*“It’s so frustrating when she goes into hospital and the nurses and the doctors say it’s her condition, you know. I’m like I’m with her twenty-four hours a day, I know how breathless she is without infection and I know how breathless she is with an infection there’s a major difference"*. *“I see him every day, they are just little subtle changes, they are not showing up in the numbers the doctors are concerned with but I see it”*.

### Mental health

Twenty-five quantitative,^36–62^ 20 qualitative^63–83^ and 1 mixed methods^84^ study addressed mental health. This encompassed depression and burden. Caregivers described an internal and external conflict of emotions, recognising a psychological change within themselves and the care recipient. Maintaining hope and positivity, versus managing worries, fears and anxieties was predominant.^62–82^ The HF study by Pressler *et al* identified caregivers had moderately poor health at baseline and at 8 months but they had fewer depressive symptoms over time.^54^ Burden arose due to greater responsibilities.^65 68 73 81 82^ Yeh and Bull noted the quality of relationship and lack of family support significantly predicted greater family caregiver burden.^53^ Nӓsstrӧm *et al* reported caregiver burden was concerned with the future and their fears of potential demands.^84^ Those with greater resiliency appeared to adjust and cope better with the illness trajectory.^64 65 76 77^ Caregivers described mental adjustment after an acute event.^77^ Living through an acute event was long lasting and some experienced post-traumatic symptoms.^80^

### Caregiver role

This is addressed in 18 qualitative^64–72 74 75 77–83 85^ and 14 quantitative studies.^36 39–41 49–51 54 57 59 60 86–89^ Caregiver role is complex and requires much coordination.^74 81 83^ Caregivers describe significant role change such as increasing domestic tasks.^63 66 69 71 76–79 82^ Role loss is prevalent^64 65 70^ and caregivers need to reframe their identity.^72 80^ Societal expectation regarding the relationship and gender, influences caregivers adjusting to their roles.^65 67 68 74 79^ Caring can be positive and rewarding. Caregivers learn about themselves and strengths they have.^65 75–77 80 81 83^ Pressler *et al* described the tasks involved: domestic, emotional support, managing dietary needs and transport.^54^ Pressler *et al* also reported that caregivers of persons with greater HF symptoms experienced more difficulty with their role.^54^

### Lifestyle changes

Fourteen quantitative^36 38–40 43 45 52 54 59 60 62 87 88 90^ and 21 qualitative^63–83 85^ studies addressed lifestyle changes. Caregivers experienced leisure, social and work-related problems.^36 39 90^ Caring interrupted and eliminated tasks from their routine.^36 39 59^ Contrastingly, Pressler *et al* reported caregivers' perceptions of how their lives changed as a result of caregiving was neutral and improved from baseline to 4 and 8 months.^54^ Caregivers became adaptable in their new role.^72 80 85^ There was less personal time for leisure and hobbies either alone or with the care recipient.^67–70 76 77 82 83^ Caregivers described daily ‘ups and downs’ and had to adjust their routines dependent on the presentation of the care recipient.^63 64 66 71 73 75 78 79 81 83^

### Support for caregivers

Fifteen quantitative,^41 45–47 49 51–53 55 56 58–60 62 87 89^ 21 qualitative^63–83 85^ and 1 mixed methods^84^ study examine support. This includes healthcare, family and social support. The weight of perceived external expectations, the necessity of being proactive in obtaining support and maintaining a social role was described across all diagnoses.^45 46 48–50 53–57 61 89^ Yeh and Bull identified lack of family support as a significant issue.^53^ Caregivers felt abandoned by healthcare teams. After hospital discharge they had to provide care without advice or medical support.^66 72 78^ Positive interactions were reported, namely access to healthcare professionals via telephone or home support.^63 64 77 84^

### Knowledge

This was addressed in 17 qualitative,^63 65–75 77 79 80 82 83 85^ 5 quantitative^50 60–62 87^ and 1 mixed methods^84^ study. This describes caregivers' understanding of the diagnosis and need for knowledge throughout the duration of illness.^63 67 70 75 83–85^ Caregivers report information from health professionals was often inadequate.^71 73 74^ Timing and format of information was significant. Caregivers received information verbally or by leaflets in hospital but describe being left alone to provide care in the long term.^65 68 69 79 82^ Caregivers had difficulty understanding how to navigate the care system.^72 80^ They had to make decisions without full knowledge of the consequences of their decision making, particularly during acute exacerbations.^65^ The quantitative element of mixed methods study by Nӓsstrӧm *et al* correlated with qualitative studies; receipt of sufficient information was central to managing HF and was associated with better perceived health of caregivers.^84^

### Relationships

Twenty qualitative,^64–83 85^ 22 quantitative^35 37 38 42–44 46–52 54–57 61 87 90–92^ and 1 mixed methods^84^ study examined relationships. In HF studies caring for individuals with more symptoms resulted in poorer perceived experiences.^54 91^ Higher relationship quality resulted in less burden and more benefit from the relationship. The relationship prior to diagnosis influenced the current relationship. Perspective of the relationship was either a sense of duty^65 74 80 81 84^ or this was a valuable second chance.^66 75 82 83^ Caregivers reported difficulty communicating about the condition leading to isolation, stress and conflict between caregiver and care recipient.^71 73^ The relationship requires negotiation.^69 85^ Caregivers prioritised the care recipient over their own needs.^64 72 74 77 82^

### Expert by experience

Twelve qualitative studies^65–70 72 75 76 80 81 83 85^ addressed this concept. Caregivers learnt new skills. They became ‘experts by experience’ discovering through ‘doing’ and observing health professionals.^66 68 83^ They developed ‘proto-professional skills’; in medication administration^65 80 85^ judging care recipients' level of functioning^79^ and decision making in times of exacerbations.^70^ Caregivers observed the nuances of change in the care recipient often not perceived by healthcare teams or other family members such as skin colour or irritability.^72 75 81^

### Vigilance

Vigilance was recurring in caregivers’ narrative across all diagnoses and was present in 19 qualitative studies.^64–81 83 85^ Caregivers were always on the alert observing the care recipient.^66 67 70 72–74 77–79 81^ They lay awake at night listening for their partners’ breath.^69 71 75 85^ This impacted on caregivers’ health creating constant fatigue, worry and stress.^65 79^ Caregivers recognised that the need for vigilance came from themselves and their insecurities.^64 76 83^

### Time

Time explores how caregivers adjusted to living with the illness and was present in 15 qualitative studies.^65 67–77 80 82 83 85^ Caregivers adapted to a new life, referring to ‘then’, how life was and ‘now’ their current life.^69 70 75 76 83^ The duration of caregiving and severity of illness influenced caregiver’s ability to adjust.^66 73 76^ Caregivers lived day by day^83^ and viewed the future, with hope or uncertainty about what lay ahead.^65 70 72 79 82^

### Shared care

Shared care was present in 16 qualitative studies.^63–66 68–76 80 81 83 85^ This demonstrates caregiver and care recipient working together managing the illness, jointly administering medication^68 81^ and attending appointments.^73^ The presence of illness was a process they adjusted to together.^76 80^ Caregivers referred to themselves and the care recipient as ‘we’, when discussing dealing with the illness.^63 71 75^ The mutual perspective between caregiver and care recipient served to isolate them from the world, the illness was *‘taking a life of its own…it’s like this third person’* (Hynes, 2012, p. 1071).

There were differences in caregiver experience for each of the diagnoses and these are discussed below.

### Heart failure

HF caregivers experienced an ‘ebb and flow’ in caring, an underlying worry, fear and anxiety, which at times of change or illness heightened.^49 51 59 71 77 81 83 85 92^ Lifestyle changes were long lasting and sustained.^39 59 64 71 77 81 83 85 92^ Obtaining knowledge was necessary throughout all stages of the illness.^50 63 66 73 83 85 92^ Sourcing information and communication with health professionals was often difficult.^63 66 71 85 92^ In spousal relationships, they predominantly viewed the care recipient as another child or as a ‘duty’.^50 51 64 66 71 73 77 84^

### Chronic obstructive pulmonary disease

COPD caregivers experienced a prolonged impact on their mental health similar to HF caregivers.^41 44 52 60 65 70 80^ Severity of illness was influential on their experience of burden.^38 43 44 60^ Role change was long lasting and profound for many.^65 67 70 80^ They expressed concerns with perceived lack of knowledge.^62 65 70 74 80^ During exacerbations, COPD caregivers experienced anxiety and fear of their loved one dying.^65 67 70 74^ COPD caregivers highlighted the loss of social roles while trying to maintain the dignity of their loved ones.^65 70 74 80 89 90^ The coughing and spitting associated with COPD often left the care recipient embarrassed.^65 67 80^ The caregiver tried to avoid situations where this would happen. The dynamics of spousal relationships changed, caregivers described losing the intimate love they had for their partner.^65 70 74 80^

### Coronary artery disease

Caregivers of patients with CAD experienced intense role change on discharge from hospital and in the acute phase of illness.^47 68 79 82 87 88^ They initially engaged with a high volume of tasks which reduced over time.^46 47 68 79 82 87 88^ CAD caregivers experienced post-traumatic symptoms if they witnessed the recipient experience an acute event.^79 82^ Caregivers described being unable to talk about this and reliving the event in their heads. Anxiety did ease over time for many.^79 82^ Caregivers felt unprepared at hospital discharge and highlighted not realising how much their routine would be disrupted.^68 79 82^ Caregivers reported viewing the experience as a second chance and had a renewed sense of love and appreciation for the relationship.^79 82^

## Discussion

This mixed methods systematic review demonstrates the similarities and differences in caregiver experiences across three common cardiorespiratory conditions. It highlighted the differences in experiences obtained from qualitative and quantitative research. Commonly occurring experiences included the exacerbation of caregiver physical and mental health due to the role. This correlates with systematic review of HF caregivers by Kang *et al* identifying that caregiving resulted in a multitude of changes in caregiver’s lives regardless of age, gender and ethnicity.^93^ Addressing both patient and caregiver needs in order to maintain well-being for both is important^19^ and recognises the value of ‘shared-care’ between patient and caregiver. The prevalence of mental health needs in this review demonstrates the need for psychosocial support for caregivers. This concurs with the studies by AasbØ *et al*, identifying caregivers need to be in ‘emotional control'^94^ and Wingham *et al*, describing the ‘enduring anguish’ experienced by caregivers.^95^ Lawton *et al* attribute caregiver well-being to the commitment of the caregiver to the role and dealing with competing demands, which can increase burden and negatively impact affect. Spousal caregivers may be more ready to accept the role of caregiving than adult children who may view it as an imposition on an already established lifestyle.^96^ In this review, societal expectations had an impact on how caregivers adjust to their role. Additionally, the quality of the relationship prior to becoming a caregiver had an influence on the caregiver subjective experience of burden.

Caregivers had predominantly negative experiences of support and described uncertainty of how to obtain this. Caregivers need greater support and knowledge transfer to conduct their role.^97^ They should be included in clinical appointments^98^ to ensure they are not isolated in providing care and to allow for knowledge exchange. Giacomini *et al* in their review of caregivers living and dying with COPD reported increasing isolation in addition to their own health issues.^18^ They described pressure balancing their variety of roles; similar experiences to the caregivers in this synthesis across all diagnoses. Caregivers emphasised their need to be vigilant. This falls into five categories as defined by Mahoney’s study of Alzheimer’s caregivers; ‘watchful supervision’, ‘protective intervening’, ‘anticipating’, ‘on duty’ and ‘being there’. Caregivers in this synthesis described overt vigilance, putting one’s head on the chest of the recipient to check breathing or covert vigilance; observing them throughout the day.^99^ Healthcare professionals must be aware of these levels of vigilance and the constant presence of them to support caregivers in their role.

Caregivers are valuable providers of care. Caregiver’s needs should be assessed systematically and in a formalised manner in healthcare settings.^16^ When developing collaborative models of care the inclusion of caregivers is imperative.^100^

### Strengths and limitations

This review demonstrates the complexity of what it means to be a caregiver and should inform clinical care development of interventions. A mixed methods review can be contentious^101^ due to the synthesis of differing paradigms. In this review, it required transformation of quantitative data into qualitative data.^15 102^ We aimed to present a convergence of caregiver experiences by conducting a mixed methods synthesis. However, it demonstrated four differing concepts between the two paradigms. This highlights the challenge of synthesising multiple methods. It is worth exploring how the four additional qualitative concepts could be captured quantitatively in order to inform healthcare intervention. This mixed methods synthesis is, to our knowledge, the first to combine caregiver experiences in HF, COPD or CAD. It examines the differences and similarities in experiences, establishing a comprehensive assessment of the knowledge base of caregiver experiences in common cardiorespiratory conditions.

There are limitations to this study; both in our review methods and the nature of included studies. First, we acknowledge that the inclusion of lower quality quantitative studies may lead to risk of bias: the majority of quantitative studies used convenience sampling, had a high attrition and low response rate. Non-validated outcome measures were used in some quantitative studies with the majority of studies conducting univariate rather than multivariate analysis. However, given the limited number of high-quality quantitative studies (four studies), we believe this broader inclusion increased the scope of our review in order to achieve a holistic understanding of caregiver experiences. Furthermore, we would note that the conclusions of this review were broadly the same with consideration of only the high-quality quantitative studies. Second, studies were restricted to English language only, from high-income countries and excluding caregivers of nursing home residents. This may limit the applicability of findings to other settings. Third, converting quantitative data into a qualitative data set risks the quantitative data set being oversimplified. This was managed with regular research team meetings to review each stage of this process. Fourth, qualitative synthesis is an interpretation of other researcher’s interpretations. To minimise individual interpretative bias, a second researcher was used to seek confirmation of the results. Finally, included qualitative and quantitative studies were primarily cross-sectional in design, therefore considering caregiver experience only at a single point of time.

### Implications for practice and future research

There are a number implications following this review. It has demonstrated there are similarities and differences in the caregiver experience in HF, COPD or CAD. The impact on caregiver’s lives of those with HF and COPD appears longer lasting and more turbulent than caring for patients with CAD. CAD caregiver’s experience of hospital during exacerbations increased distress at discharge. This review reflects the complexity of the caregiver’s role. The mixed methods approach indicted differences in what is being investigated. This is important in demonstrating an understanding of the caregiver experience when dealing with complex conditions. Future research should focus on involving caregivers in the design and delivery of interventions for patients with cardiopulmonary disease. Best practice interventions for CAD caregivers in the discharge process from hospital to home must be formalised. There appears to be a focus on the mental health of caregivers of those with HF; however, further research is needed to explore this in COPD and CAD caregivers. Exploration of this via support groups for caregivers of cardiorespiratory conditions is merited. Clinically, the healthcare team need to identify who the caregiver is and be aware of their needs with the use of a carer’s needs assessment. There must be a greater understanding of caregiver support needs, what they avail of and are they aware of what is available to them in the community. This can be achieved in conversation between the healthcare team and caregivers and warrants further research as to how and whether caregivers avail of external supports.

Consideration needs to be given as to whether quantitative research tools to explore caregiver expertise, view of the future, experience of shared care and vigilance can be developed to capture these qualitative concepts to inform the development of self-management interventions for patients and caregivers. Repeated measures examining perceived control and caregiver needs may contribute to a greater understanding of caregiver experiences, which arose in qualitative studies. Additionally, longitudinal studies with repeated assessment need to be conducted to assess the stability of caregiver experiences and whether they are liable to much change over time. In this review, only 4 out of 32 quantitative studies examined caregiver’s experiences longitudinally. Understanding whether there are caregiver changes over time will facilitate greater understanding of caregiver needs for health professionals when working with this population. The emergence of additional concepts from qualitative studies emphasises the role of mixed methods research when examining lived experiences. The additional concepts also demonstrated the nuanced expertise of the caregiving experience. It is important for researchers to consider how to reflect this in quantitative investigation so as to inform funders in order to develop and trial interventions in HF, COPD and CAD. The quality of quantitative studies in COPD and CAD were medium or low. There is a need for more empirically robust studies examining the experiences of these caregivers. Additionally, greater understanding of caregiver’s experiences with this population will facilitate the development of robust evidence-based guidelines for health services when working with HF, COPD and CAD.

## Conclusions

This mixed methods systematic review provides a holistic synthesis of caregiver experiences of people with HF, COPD or CAD. It demonstrates there are a number of implications when an individual becomes a caregiver for those with chronic cardiopulmonary disease. Caregivers reframe their identity and change their life course. Caregivers learn a multitude of skills and develop expertise in their new role. Their expertise is invaluable and should be acknowledged in healthcare interventions for these conditions. The quality of evidence was limited by assessment of caregiver experience at single time point. There is need for future studies that employ longitudinal designs examining the change in caregiver experience over time. Caregiving can be positive if caregivers have access to support, are well informed and part of the healthcare team. Understanding the experiences of caregivers for people with these conditions allows healthcare professionals and policy makers to reflect on our approach. Health services must consider caregivers in the design and delivery of interventions.

## Supplementary Material

Reviewer comments

Author's manuscript
