# Supplementary material for: ’Who Cares?' The experiences of caregivers of adults living with heart failure, chronic obstructive pulmonary disease and coronary artery disease: a mixed methods systematic review
Source: BMJ Open. 2018 Jul 11;8(7):e020927. doi: 10.1136/bmjopen-2017-020927 (PMC6082485; doi:10.1136/bmjopen-2017-020927)
Supplement: Supplementary file 1 [file bmjopen-2017-020927supp001.pdf]

Supplementary:

Table 1: List of search terms

Medline:

MeSH: Caregivers

“Caregivers”.ti,ab OR “care-giver”.ti,ab OR “carer*.ti,ab” OR “informal car*”.ti,ab

AND

MeSH: Quality of life

“Quality of life” OR “outcome measures” OR “caregiver outcomes” OR “care-giver outcomes” OR “time use” OR “time-use” OR “occupations” OR “occupational engagement” OR “self-efficacy” OR “self efficacy” OR “experience” OR “emotion*” OR “psychological health impact” OR “physical health impact” OR “confidence” OR “self-confidence” OR “satisfaction” OR “dissatisfaction” OR “activities of daily living” OR “resilience” OR “social adj2 (interaction or engagement or support) OR “Social adj2 (participation)” OR “caregiver adj2 (support or health professional or medical team or nurse or patient or relationship* or knowledge)” OR “information exchange” OR “coping strategies” OR “leisure activity” OR “conflict” OR “caregiver responsib*” OR care-giver responsib*” OR “caregiver expectation” OR “care-giver expectation” OR “caregiver role” OR “care-giver role” OR “role adjustment” OR “caregiver or care-giver adj2 (attitude to health)”

AND

MeSH: Heart Failure, Heart Diseases, Myocardial Ischaemia, Coronary Artery Disease, Pulmonary Disease, Chronic Obstructive, Pulmonary Heart Disease, Chronic Obstructive Pulmonary Disease, Acute Coronary Syndrome, Cardiovascular Diseases, Coronary Artery Disease, Coronary Artery Bypass, Coronary Disease, Coronary Aneurysm, Stroke, Myocardial Infarction

“Heart Failure”.ti,ab OR “cardiac failure”.ti,ab OR “myocardial failure”.ti,ab OR “left ventricular failure”.ti,ab OR “right ventricular failure”.ti,ab OR “cardiomyopathy”.ti,ab, OR “systolic failure”.ti,ab OR “diastolic failure”.ti,ab OR “Chronic Obstructive Pulmonary Disease”.ti,ab OR “COPD”.ti,ab OR “chronic obstructive lung disease”.ti,ab OR “pulmonary disease”.ti,ab OR “pulmonary disorder”.ti,ab OR “respiratory disease”.ti,ab OR “respiratory disorder”.ti,ab OR “dyspnea”.ti,ab OR “dyspnoea”.ti,ab OR “Stroke”.ti,ab OR “stroke disability”.ti,ab OR “stroke disease”.ti,ab OR “stroke disorder”.ti,ab OR “CVA”.ti,ab OR “cerebrovascular accident”.ti,ab OR “transient ischaemic attack”.ti,ab OR “transient ischemic attack”.ti,ab OR “haemorrhage”.ti,ab OR “hemorrhage”.ti,ab OR “cerebral haemorrhage”.ti,ab OR “cerebral hemorrhage”.ti,ab OR “aneurysm”.ti,ab OR “atrial fibrillation”.ti,ab OR “Coronary Artery Disease”.ti,ab OR “acute coronary syndrome”.ti,ab OR “atherosclerosis”.ti,ab OR “arteriosclerosis”.ti,ab OR “ischaemic heart disease”,ti.ab OR “ischemic heart disease”.ti,ab OR “myocardial infarction”,ti.ab OR “coronary revascularisation”.ti,ab OR “coronary revascularization”.ti,ab OR “angina”.ti,ab OR “CABG”.ti,ab OR “coronary artery bypass graft”.ti,ab OR “stable angina”.ti,ab OR “angina pectoris”.ti,ab

CINAHL:

MeSH: Caregivers, caregiver burden, caregiver supports, caregiver role strain, risk for caregiver role strain, family caregiver status, caregiving endurance potential, caregiver-patient relationship, caregiver well-being, caregiver support, caregiver stressors, caregiver physical health, caregiver performance, caregiver performance: direct care, caregiver performance indirect care, caregiver lifestyle disruption, caregiver emotional health, caregiver strain index, caregiver role strain.

"Caregiver".ti,ab OR "care-giver".ti,ab OR "carer*.ti,ab" OR "carer".ti,ab OR "carers".ti,ab OR "informal care*".ti,ab

AND

MeSH: Quality of life

"Quality of life" OR "outcome measures" OR "caregiver outcomes" OR "time use" OR "occupations" OR "occupational engagement" OR "self-efficacy" OR "self efficacy" OR "experience" OR "emotion*" OR "psychological health impact" OR "physical health impact" OR "confidence" OR "self-confidence" OR "satisfaction" OR "dissatisfaction" OR "activities of daily living" OR "resilience" OR "social adj2 (interaction or engagement or support) OR "Social participation" OR "caregiver adj2 (support or health professional or medical team or nurse or patient or relationship* or knowledge)" OR "information exchange" OR "coping strategies" OR "leisure activity" OR "conflict" OR "caregiver responsib*" OR care-giver responsib*" OR "caregiver expectation" OR "care-giver expectation" OR "caregiver role" OR "care-giver role" OR "role adjustment" OR "caregiver or care-giver adj2 (adjustment)" OR "caregiver or care-giver adj2 (acceptance) OR "caregiver or care-giver adj2 (readiness)" OR "caregiver or care-giver adj2 (attitude to health)"

AND

MeSH: Heart Failure, Treatment Failure, Pulmonary Disease, Chronic Obstructive Pulmonary Disease, Chronic Obstructive Pulmonary Disease, Acute Coronary Syndrome, Coronary Arteriosclerosis, Coronary Artery Bypass, Coronary Disease, Coronary Aneurysm, Stroke OR Coronary Artery Disease

"Heart Failure".ti,ab OR "cardiac failure".ti,ab OR "myocardial failure".ti,ab OR "left ventricular failure".ti,ab OR "right ventricular failure".ti,ab OR "cardiomyopathy".ti,ab, OR "systolic failure".ti,ab OR "diastolic failure".ti,ab OR "COPD".ti,ab OR "chronic obstructive lung disease".ti,ab OR "pulmonary disease".ti,ab OR "pulmonary disorder".ti,ab OR "respiratory disease".ti,ab OR "respiratory disorder".ti,ab OR "dyspnea".ti,ab OR "dyspnoea".ti,ab OR "Stroke".ti,ab OR "stroke disability".ti,ab OR "stroke disease".ti,ab OR "stroke disorder".ti,ab OR "CVA".ti,ab OR "cerebrovascular accident".ti,ab OR "transient ischaemic attack".ti,ab OR "transient ischemic attack".ti,ab OR "haemorrhage".ti,ab OR "hemorrhage".ti,ab OR "cerebral haemorrhage".ti,ab OR "cerebral hemorrhage".ti,ab OR "aneurysm".ti,ab OR "atrial fibrillation".ti,ab OR "Coronary Artery Disease".ti,ab OR "acute coronary syndrome".ti,ab OR "atherosclerosis".ti,ab OR "arteriosclerosis".ti,ab OR "ischaemic heart disease",ti.ab OR "ischemic heart disease".ti,ab OR "myocardial infarction",ti.ab OR "coronary revascularisation".ti,ab OR "coronary revascularization".ti,ab OR "angina".ti,ab OR "CABG".ti,ab OR "coronary artery bypass graft".ti,ab OR "stable angina".ti,ab OR "angina pectoris".ti,ab

EMBASE:

MeSH: Caregivers

“Caregivers”.ti,ab OR “care-givers”.ti,ab OR “Caregiver”.ti,ab OR “care-giver”.ti,ab OR “carer*.ti,ab” OR “informal car*”.ti,ab

AND

MeSH: Quality of life

“Quality of life” OR “outcome measures” OR “caregiver outcomes” OR “care-giver outcomes” OR “time use” OR “occupations” OR “occupational engagement” OR (“self-efficacy” OR “self efficacy”) OR “experience” OR “emotion*” OR “psychological health impact” OR “physical health impact” OR (“confidence” OR “self-confidence”) OR “satisfaction” OR “dissatisfaction” OR “activities of daily living” OR “resilience” OR “social adj2 (interaction or engagement or support) OR “Social participation” OR “caregiver OR care-giver adj2 (support or health professional or medical team or nurse or patient or relationship* or knowledge)” OR “information exchange” OR “coping strategies” OR “leisure activity” OR “conflict” OR “caregiver responsib*” OR care-giver responsib*” OR “caregiver expectation” OR “care-giver expectation” OR “caregiver role” OR “care-giver role” OR “role adjustment” OR “caregiver or care-giver adj2 (adjustment or acceptance or readiness)” OR “caregiver or care-giver adj2 (attitude to health)”

AND

MeSH: Heart Failure, Chronic Obstructive Pulmonary Disease, Bronchitis, Chronic Obstructive, Disease exacerbation, Pulmonary Disease, Cardiovascular Disease, Stroke, Cerebrovascular Accident, Coronary Artery Disease, Ischemic Heart Disease, Coronary Artery Disease, Acute Coronary Syndrome, Heart Infarction, Coronary Aneurysm, Coronary Artery Aneurysm, Coronary Artery Bypass, Coronary Artery Bypass Graft, Coronary arteriosclerosis, Coronary Artery Atherosclerosis

“heart failure”,ti,ab OR “cardiac failure”.ti,ab OR “myocardial failure”.ti,ab OR “left ventricular failure”.ti,ab OR “right ventricular failure”.ti,ab OR “cardiomyopathy”.ti,ab, OR “systolic failure”.ti,ab OR “diastolic failure”.ti,ab OR “chronic obstructive pulmonary disease” OR “COPD”.ti,ab OR “chronic obstructive lung disease”.ti,ab OR “pulmonary disease”.ti,ab OR “pulmonary disorder”.ti,ab OR “respiratory disease”.ti,ab OR “respiratory disorder”.ti,ab OR “stroke disorder”.ti,ab OR “CVA”.ti,ab OR “cerebrovascular accident”.ti,ab OR “transient ischaemic attack”.ti,ab OR “transient ischemic attack”.ti,ab OR “haemorrhage”.ti,ab OR “hemorrhage”.ti,ab OR “cerebral haemorrhage”.ti,ab OR “cerebral hemorrhage”.ti,ab OR “aneurysm”.ti,ab OR “atrial fibrillation”.ti,ab OR “Coronary Artery Disease”.ti,ab OR “acute coronary syndrome”.ti,ab OR “atherosclerosis”.ti,ab OR “arteriosclerosis”.ti,ab OR “ischaemic heart disease”,ti.ab OR “ischemic heart disease”.ti,ab OR “myocardial infarction”,ti.ab OR “coronary revascularisation”.ti,ab OR “coronary revascularization”.ti,ab OR “angina”.ti,ab OR “CABG”.ti,ab OR “coronary artery bypass graft”.ti,ab OR “stable angina”.ti,ab OR “angina pectoris”.ti,ab

PsycInfo:

MeSH: Caregivers

“caregiver”.ti,ab OR “care-giver”.ti,ab OR “carer*.ti,ab” OR “informal car*”.ti,ab

AND

MeSH: Quality of life, Occupations, Well-being, Activities of daily living, Mental Health, Health Behaviour, Emotional Responses, Life satisfaction, Role satisfaction, Resilience, Life Experience, Communication, Strategies, Anxiety, Coping Behaviour, Distress, Recreation, Leisure Time, Participation, Adjustment, Couples

“Quality of life” OR “outcome measures” OR (“caregiver outcomes” OR “care-giver outcomes”) OR (“time-use” OR “time use”) OR “occupations” OR “occupational engagement” OR (“self-efficacy” OR “self efficacy”) OR “experience” OR “emotion*” OR “psychological health impact” OR “physical health impact” OR (“confidence” OR “self-confidence” OR self confidence) OR “satisfaction” OR “dissatisfaction” OR “activities of daily living” OR “resilience” OR “social adj2 (interaction or engagement or support or participation) OR “caregiver OR care-giver adj2 (support or health professional or medical team)” OR “information exchange” OR “coping strategies” OR “leisure activity” OR “conflict” OR “caregiver responsib*” OR care-giver responsib*” OR “caregiver expectation” OR “care-giver expectation” OR “caregiver role” OR “care-giver role” OR “role adjustment” OR “caregiver or care-giver adj2 (adjustment or acceptance or readiness)” OR “caregiver or care-giver adj2 (attitude to health)”

AND

MeSH: Heart Failure, Health Behaviour, Heart Disorders, Cardiovascular Disorders, Self-care skills, Intervention, Treatment Outcomes, Chronic Obstructive Pulmonary Disease, Pulmonary Heart Disease, Stroke, Treatment Outcomes, Intervention, Cerebrovascular Accidents, Coronary Artery Disease, Myocardial Infarctions, Ischemia, Aneurysms

“heart failure”,ti,ab OR “cardiac failure”.ti,ab OR “myocardial failure”.ti,ab OR “left ventricular failure”.ti,ab OR “right ventricular failure”.ti,ab OR “cardiomyopathy”.ti,ab, OR “systolic failure”.ti,ab OR “diastolic failure”.ti,ab OR “chronic obstructive pulmonary disease” OR “COPD”.ti,ab OR “chronic obstructive lung disease”.ti,ab OR “pulmonary disease”.ti,ab OR “pulmonary disorder”.ti,ab OR “respiratory disease”.ti,ab OR “respiratory disorder”.ti,ab OR “dyspnea”.ti,ab OR “dyspnoea”,ti,ab OR “stroke disorder”.ti,ab OR “CVA”.ti,ab OR “cerebrovascular accident”.ti,ab OR “transient ischaemic attack”.ti,ab OR “transient ischemic attack”.ti,ab OR “haemorrhage”.ti,ab OR “hemorrhage”.ti,ab OR “cerebral haemorrhage”.ti,ab OR “cerebral hemorrhage”.ti,ab OR “aneurysm”.ti,ab OR “atrial fibrillation”.ti,ab OR “Coronary Artery Disease”.ti,ab OR “acute coronary syndrome”.ti,ab OR “atherosclerosis”.ti,ab OR “arteriosclerosis”.ti,ab OR “ischaemic heart disease”,ti.ab OR “ischemic heart disease”.ti,ab OR “myocardial infarction”,ti.ab OR “coronary revascularisation”.ti,ab OR “coronary revascularization”.ti,ab OR “angina”.ti,ab OR “CABG”.ti,ab OR “coronary artery bypass graft”.ti,ab OR “stable angina”.ti,ab OR “angina pectoris”.ti,ab

Web of Science

TI = Title

TS = Topic

TI & TS = “caregiver” OR “care-giver” OR “carer*” OR “informal care*”

AND

TS = “Quality of life” OR “outcome measures” OR (“caregiver outcomes” OR “care-giver outcomes”) OR (“time-use” OR “time use”) OR “occupations” OR “occupational engagement” OR (“self-efficacy” OR “self efficacy”) OR “experience” OR “emotion*” OR “psychological health impact” OR “physical health impact” OR (“confidence” OR “self-confidence” OR self confidence) OR “satisfaction” OR “dissatisfaction” OR “activities of daily living” OR “resilience” OR “social adj2 (interaction or engagement or support or participation) OR “caregiver OR care-giver adj2 (support or health professional or medical team)” OR “information exchange” OR “coping strategies” OR “leisure activity” OR “conflict” OR “caregiver responsib*” OR care-giver responsib*” OR “caregiver expectation” OR “care-giver expectation” OR “caregiver role” OR “care-giver role” OR “role adjustment” OR “caregiver or care-giver adj2 (adjustment or acceptance or readiness)” OR “caregiver or care-giver adj2 (attitude to health)”

AND

TI & TS = “heart failure” OR “cardiac failure” OR “myocardial failure” OR “left ventricular failure” OR “right ventricular failure” OR “cardiomyopathy” OR “systolic failure” OR “diastolic failure” OR “chronic obstructive pulmonary disease” OR “COPD” OR “chronic obstructive lung disease” OR “pulmonary disease” OR “pulmonary disorder” OR “respiratory disease” OR “respiratory disorder” OR “dyspnea” OR “dyspnoea” OR “stroke disorder” OR “CVA” OR “cerebrovascular accident” OR “transient ischaemic attack” OR “transient ischemic attack” OR “haemorrhage” OR “hemorrhage” OR “cerebral haemorrhage” OR “cerebral hemorrhage” OR “aneurysm” OR “atrial fibrillation” OR “Coronary Artery Disease” OR “acute coronary syndrome” OR “atherosclerosis” OR “arteriosclerosis” OR “ischaemic heart disease” OR “ischemic heart disease” OR “myocardial infarction” OR “coronary revascularisation” OR “coronary revascularization” OR “angina” OR “CABG” OR “coronary artery bypass graft” OR “stable angina” OR “angina pectoris”

ProQuest: Global Dissertations & Theses/ Applied Social Sciences Index and Abstracts (ASSIA)

“caregiver”.ti,ab OR “care-giver”.ti,ab OR “carer*.ti,ab” OR “informal car*”.ti,ab

AND

“Quality of life” OR “outcome measures” OR (“caregiver outcomes” OR “care-giver outcomes”) OR (“time-use” OR “time use”) OR “occupations” OR “occupational engagement” OR (“self-efficacy” OR “self efficacy”) OR “experience” OR “emotion*” OR “psychological health impact” OR “physical health impact” OR (“confidence” OR “self-confidence” OR self confidence) OR “satisfaction” OR “dissatisfaction” OR “activities of daily living” OR “resilience” OR “social adj2 (interaction or engagement or support or participation) OR “caregiver OR care-giver adj2 (support or health professional or medical team)” OR “information exchange” OR “coping strategies” OR “leisure activity” OR “conflict” OR “caregiver responsib*” OR care-giver responsib*” OR “caregiver expectation” OR “care-giver expectation” OR “caregiver role” OR “care-giver role” OR “role adjustment” OR “caregiver or care-giver adj2 (adjustment or acceptance or readiness)” OR “caregiver or care-giver adj2 (attitude to health)”

AND

“heart failure”,ti,ab OR “cardiac failure”.ti,ab OR “myocardial failure”.ti,ab OR “left ventricular failure”.ti,ab OR “right ventricular failure”.ti,ab OR “cardiomyopathy”.ti,ab, OR “systolic failure”.ti,ab OR “diastolic failure”.ti,ab OR “chronic obstructive pulmonary disease” OR “COPD”.ti,ab OR “chronic obstructive lung disease”.ti,ab OR “pulmonary disease”.ti,ab OR “pulmonary disorder”.ti,ab OR “respiratory disease”.ti,ab OR “respiratory disorder”.ti,ab OR “dyspnea”.ti,ab OR “dyspnoea”,ti,ab OR “stroke disorder”.ti,ab OR “CVA”.ti,ab OR “cerebrovascular accident”.ti,ab OR “transient ischaemic attack”.ti,ab OR “transient ischemic attack”.ti,ab OR “haemorrhage”.ti,ab OR “hemorrhage”.ti,ab OR “cerebral haemorrhage”.ti,ab OR “cerebral hemorrhage”.ti,ab OR “aneurysm”.ti,ab OR “atrial fibrillation”.ti,ab OR “Coronary Artery Disease”.ti,ab OR “acute coronary syndrome”.ti,ab OR “atherosclerosis”.ti,ab OR “arteriosclerosis”.ti,ab OR “ischaemic heart disease”,ti.ab OR “ischemic heart disease”.ti,ab OR “myocardial infarction”,ti.ab OR “coronary revascularisation”.ti,ab OR “coronary revascularization”.ti,ab OR “angina”.ti,ab OR “CABG”.ti,ab OR “coronary artery bypass graft”.ti,ab OR “stable angina”.ti,ab OR “angina pectoris”.ti,ab

Ethos – British Library

CAREGIVER or CARE-GIVER.ti,ab

Searches initially included Heart Failure, Chronic Obstructive Pulmonary Disease, Stroke and Coronary Artery Disease as “AND” terms, however these yielded no results

Table 2 (a)

Caregiver experience - Concepts/Mapping Table – Qualitative Papers

MENTAL HEALTH = 1 **ROLE = 2** **LIFESTYLE CHANGE = 3**
SUPPORT = 4 **KNOWLEDGE/INFORMATION = 5**
RELATIONSHIPS = 6

Concepts only in Qualitative Papers: **EXPERT BY EXPERIENCE = 7** **VIGILANCE = 8**
TIME = 9 **SHARED CARE = 10**

	**1**	**2**	**3**	**4**	**5**	**6**	**7**	**8**	**9**	**10**
Andersen (2015) **(HF)**	✓		✓	✓	✓					✓
Baker (2010) **(HF)**	✓	✓	✓	✓		✓		✓		✓
Bove (2016) **(COPD)**	✓	✓	✓	✓	✓	✓	✓	✓	✓	✓
Burke (2014) **(HF)**	✓	✓	✓	✓	✓	✓	✓	✓		✓
Clark (2008) **(HF)**		✓	✓	✓	✓	✓	✓	✓	✓	✓
Figueiredo (2015) **(COPD)**	✓	✓	✓	✓	✓	✓	✓	✓	✓	
• Halm (2016) **(CAD)**	✓	✓	✓	✓	✓	✓	✓	✓	✓	✓
• Halm (2017) **(CAD)**	✓	✓	✓	✓	✓	✓	✓	✓	✓	✓
Hynes (2012) **(COPD)**	✓	✓	✓	✓	✓	✓	✓	✓	✓	✓
Imes (2011) **(HF)**	✓	✓	✓	✓	✓	✓		✓	✓	✓
Kitko (2010) **(HF)**	✓	✓	✓	✓	✓	✓	✓	✓	✓	✓
Liljeroos (2014) **(HF)**	✓		✓	✓	✓	✓		✓	✓	✓
Lindqvist (2013) **(COPD)**	✓	✓	✓	✓	✓	✓		✓	✓	✓
Luttik (2007) **(HF)**	✓	✓	✓	✓	✓	✓	✓	✓	✓	✓
Marcuccilli (2011) **(HF)**	✓		✓	✓		✓	✓	✓	✓	✓
Marcuccilli (2014) **(HF)**	✓	✓	✓	✓	✓	✓		✓	✓	
Pattenden (2007) **(HF)**	✓	✓	✓	✓		✓		✓		
Rolley (2010) **(CAD)**	✓	✓	✓	✓	✓	✓		✓		
Spence (2008) **(COPD)**	✓	✓	✓	✓	✓	✓	✓	✓	✓	✓

Strom (2015)<br>**(HF)**	✓	✓	✓	✓		✓	✓	✓		✓
Wallin (2013)<br>**(CAD)**	✓	✓	✓	✓	✓	✓			✓	
Wingham (2015)<br>**(HF)**	✓	✓	✓	✓	✓	✓	✓	✓	✓	✓
**Heart Failure**	**12**	**10**	**13**	**13**	**9**	**12**	**7**	**12**	**8**	**11**
**COPD**	**5**	**5**	**5**	**5**	**5**	**5**	**4**	**5**	**5**	**4**
**CAD**	**3**	**3**	**3**	**4**	**3**	**3**	**1**	**2**	**2**	**1**
**Total**	**20**	**18**	**21**	**21**	**17**	**20**	**12**	**19**	**15**	**16**

• = same study: counted as one

Table 2 (b)

Caregiver experience - Concepts/Mapping – Quantitative Papers

MENTAL HEALTH IMPACT = 1 **ROLE = 2** **LIFESTYLE CHANGE = 3**
SUPPORT = 4 **KNOWLEDGE/INFORMATION = 5**
RELATIONSHIPS = 6

	**1**	**2**	**3**	**4**	**5**	**6**
Agren (2010) **(HF)**	✓					✓
Al-Rawashdeh (2017) **(HF)**	✓		✓			✓
Badr (2017) **(COPD)**	✓		✓			✓
Bakas (2006) **(HF)**	✓	✓	✓			
Chung (2016) **(HF)**	✓	✓	✓			
Cossette (1993) **(COPD)**	✓	✓		✓		
Evangelista (2002) **(HF)**	✓					✓
Figueiredo (2013) **(COPD)**	✓		✓			✓
Figueiredo (2014) **(COPD)**	✓					✓
Grigorovich (2017) **(HF)**	✓		✓	✓		
• Halm (2006) **(CAD)**	✓			✓		✓
• Halm (2007) **(CAD)**	✓			✓		✓
Hess (2009) **(HF)**		✓				
Hooley (2005) **(HF)**	✓					✓
Hwang (2011) **(HF)**	✓	✓		✓		✓
Karmilovich (1994) **(HF)**	✓	✓			✓	✓
Kneeshaw (1999) **(CAD)**		✓	✓	✓	✓	✓
Loftus (2004) **(HF)**	✓	✓		✓		✓
Lum (2014) **(HF)**						✓
Luttik (2009) **(HF)**						✓

Miravitlles (2015) **(COPD)**			✓			✓
Nakken (2017) **(COPD)**	✓		✓	✓		✓
Park (2013) **(CAD)**		✓	✓			✓
Pi-Ming Yeh (2012) **(HF)**	✓			✓		
Pressler (2013) **(HF)**	✓	✓	✓			✓
Riegner (1996) **(COPD)**		✓		✓		✓
• Saunders (2008) **(HF)**	✓			✓		✓
• Saunders (2009) **(HF)**	✓			✓		✓
Saunders (2010) **(HF)**	✓	✓				✓
Schwarz (2003) **(HF)**	✓			✓		
Scott (2000) **(HF)**	✓	✓	✓	✓		
Takata (2008) **(COPD)**	✓	✓	✓	✓	✓	
Vellone (2015) **(HF)**	✓				✓	✓
Woolfe (2007) **(COPD)**	✓		✓	✓	✓	
**Heart Failure**	**15**	**9**	**6**	**7**	**2**	**13**
**COPD**	**5**	**3**	**6**	**5**	**2**	**6**
**CAD**	**4**	**2**	**2**	**3**	**1**	**3**
**Mixed Method Study**	**1**					
**Total**	**25**	**14**	**14**	**15**	**5**	**22**

• = same study: counted as one

Table 2 (c)

Caregiver experience - Concepts/Mapping – Mixed Method Papers

MENTAL HEALTH IMPACT = 1 **ROLE = 2** **LIFESTYLE CHANGE = 3**
SUPPORT = 4 **KNOWLEDGE/INFORMATION = 5**
RELATIONSHIPS = 6

	**1**	**2**	**3**	**4**	**5**	**6**
Näsström (2017) **(HF)**	✓			✓	✓	✓
**TOTAL**	**1**			**1**	**1**	1
