# Supplementary material for: ’Who Cares?' The experiences of caregivers of adults living with heart failure, chronic obstructive pulmonary disease and coronary artery disease: a mixed methods systematic review
Source: BMJ Open. 2018 Jul 11;8(7):e020927. doi: 10.1136/bmjopen-2017-020927 (PMC6082485; doi:10.1136/bmjopen-2017-020927)
Supplement: Supplementary file 2 [file bmjopen-2017-020927supp002.pdf]

Table 1

Individual Study Results

First Author/Year	Diagnosis	Outcome Measure	Result/Findings
Ågren (2010)	HF<br>(NYHA II-IV)	Caregiver Burden Scale<br>Short Form (SF)-36<br>Beck Depression Inventory<br>Control Attitude Scale<br>Knowledge Questionnaire<br>Charlson Co-morbidity Index	68% of partners = low level burden<br>30% of partners = medium burden<br>2% of partners = high burden<br>Regression analysis showed poor mental health ($p=0.001$) and lower perceived control ($p=0.001$) in partners and poor patient physical health ($p=0.001$) predicts higher caregiver burden
Al-Rawashdeh (2017)	HF<br>(NYHA I-IV)	Beck Depression Inventory<br>Minnesota Living with HF (modified for caregivers)<br>Patient Health Questionnaire<br>Short-Form 12 Health Survey	Patients whose spouse has higher sleep disturbance have poorer mental well-being ($\beta = -2.19$, $p <0.05$)<br>Caregiver sleep disturbance significantly correlated with their own physical ($\beta = -4.94$, $p< 0.001$) and mental well-being scores ($\beta = -6.62$, $p< 0.001$)
Andersen (2015)	HF	n/a	1) Involvement, willingness to assume responsibility and desire for knowledge<br>2) Unclear Responsibility and Insufficient Flow of Information<br>3) Available and Competent Supporters
Badr (2017)	COPD	Patient Health Questionnaire<br>COPD Severity Index<br>Activities of Daily Living scale<br>Lubben Social Network Scale<br>Zarit Burden Interview<br>Checklist created for this study re: co-morbidities (patients & caregivers)<br>Smoking status<br>Self-management behaviours – pts.	30% of patients and 20% of caregivers = mild depression<br>30% of patients and 8% of caregivers = moderate to severe depression.<br>Depression levels of patients and caregivers were significantly correlated ($r=0.28$, $P= 0.01$)

First Author/Year	Diagnosis	Outcome Measure	Result/Findings
Bakas (2006)	HF<br>(NYHA II-IV)	Control Attitudes Scale<br>Oberst Caregiving Burden Scale<br>Bakas Caregiving Outcomes Scale<br>Medical Outcomes Study<br>General Health	Caregivers reported their lives had changed for the worse due to providing care (M= 3.7, SD =1.5, 1 worst, 7 best<br>Performing household tasks and managing patient behaviours were most difficult, and the caregiver's emotional<br>and financial well-being, time for social activities, and general health had deteriorated. (ρ -0.46, $p < 0.05$)
Baker (2010)	HF<br>(LVAD in situ)	n/a	1) Commitment
Bove (2016)	COPD<br>(GOLD C&D)	n/a	1) Undefined and unpredictable responsibility
Burke (2014)	HF<br>(NYHA II-IV)	n/a	1) Health Manager/Care Plan Enforcer<br>2) Advocate for Improved Quality of Life<br>3) Expert in the Lived Experience of HF<br>4) Expressions of Role Conflict and Role Strain
Chung (2016)	HF<br>(NYHA II-IV)	Beck Depression Inventory-II<br>Zarit Burden Interview<br>Oberst Caregiving Burden Scale<br>Bakas Caregiving Outcome Scale<br>Medical Outcome Study	42% reported severe burden<br>Caregivers of patients with depressive symptoms had a higher level of burden (25±13 vs 13.5±12 on the ZBI; p<0.001),<br>Caregivers reported worse mental quality of life (46±10 vs 51±10 on the -12v2; p=0.026) than those of patients without depressive symptoms
Clark (2008)	HF<br>(NYHA II – IV)	n/a	1) The sharing of caring<br>2) The basis of care: formal and informal forms of knowledge about CHF<br>3) The activities of informal care: visible and invisible
Cossette (1993)	COPD<br>(GOLD III-V)	The SCL-90 scale<br>The subjective Stress Scale<br>Caregiving tasks index<br>The family/friend support index	Nearly 40% of wives used psychotropic drugs<br>Difficult emotional support significantly predicted somatization (β= 0.26, p ≤ 0.05), depression (β= 0.34, p ≤ 0.01), obsession compulsion (β= 0.43, p ≤ 0.01), anxiety (β= 0.26, p ≤ 0.05), and hostility (β= 0.37, p ≤ 0.01)

First Author/Year	Diagnosis	Outcome Measure	Result/Findings
Evangelista (2002)	HF<br>(NYHA I – IV)	SF-12<br>(Mental Component Summary Scale)	Caregiver emotional wellbeing associated with patient's emotional wellbeing<br>Caregivers' mental health score: Adjusted R2 = 0 .536, F = 40.299, p = .000<br>Caregivers reported higher mental wellbeing than patients (P < .001, p = 0.018 (patients), p = 0.118).
Figueiredo (2013)	COPD<br>(GOLD I-IV)	Carers Assessment of Managing Index (Portuguese version)<br>International Classification of Functioning Disability and Health	Better physical health perception was associated with an increasing use of problem-solving strategies (r_s = -0.313; p = 0.029)
Figueiredo (2014)	COPD<br>(GOLD I-IV)	International Classification of Functioning Disability and Health<br>Hospital Anxiety and Depression Scale (Portuguese version)<br>Carers' Assessment of Difficulties Index (Portuguese version)	61.9% = caregivers of early COPD patients observed to be anxious<br>70.4% = caregivers of advanced COPD patients observed to be anxious<br>Depression significantly more frequent in caregivers of advanced COPD patients (p = 0.030)<br>Self-rated physical (p = 0.035) and mental health (p = 0.011) worse in caregivers of advanced COPD patients
Figueiredo (2015)	COPD<br>(Mod – Severe)	n/a	1) Meaning<br>2) Challenges and constraints<br>3) Fears and concerns about the future<br>4) Needs<br>5) Positive aspects
Grigorovich (2017)	HF<br>(NYHA II-IV)	Center for Epidemiological Studies-Depression (CES-D)<br>Positive Affect Scale<br>Caregiver Impact Scale<br>Caregiver Assistance Scale<br>Pearlin Mastery Scale<br>Medical Outcomes Study Social, Support Survey, Personal Gain Scale, Brain Impairment Behaviour, MLWHFQ	Caregivers depression remained stable over1 year (mean -0.17 ± SE 0.11, p > 0.1<br>Caregivers depression associated with participation restriction (p< 0.05)<br>Caregivers positive affect remained stable over 1year (0.10 ± 0.10, p > 0.1)<br>Positive affect associated (p<0.05) with caregivers feelings of personal gain and social support

First Author/Year	Diagnosis	Outcome Measure	Result/Findings
*Halm et al. (2006)	CAD	Caregiving Burden Scale<br>Bakas Caregiving Outcomes Scale<br>SF-12 (spouse proxy ratings)<br>Karnofsky Performance Scale<br>Caregiver Competence Scale<br>State-Trait Anxiety Inventory<br>Center for Epidemiologic Studies Depression Scale<br>Mutuality Scale<br>Expressive Support Scale	Higher burden scores were associated with patient's gender (female) (β = .21, p = .02), increased personal gain (β = .29, p = .004), increased caregiver competence (β = .20, p = .05).<br>Lower burden was associated with better health status of the patient (β= -.22, p = .02), higher caregiver mental HRQL (β = -.38, p = .01).<br>caregiver burden was not significantly different at 3, 6, or 12 months post-surgery
*Halm et al. (2007)	CAD	(as above Halm, 2006)	Controlling for caregiver and patient characteristics, caregivers who perceived less mastery (β = 0.45, p = .001) had higher depressive symptoms
*Halm (2016)	CAD	n/a	1) Knowing what I am supposed to be doing<br>2) Managing multiple medications<br>3) Assisting with mobility<br>4) Dealing with unmonitorable symptoms<br>5) Managing poor appetite<br>6) Keeping spirits up<br>7) Navigating memory loss & confusion<br>8) Surviving moodiness<br>9) Dealing with financial matters
*Halm (2017)	CAD	n/a	1) Caregiving work – comfort, monitoring, support & functional
Hess (2009)	HF	Short-Form Test of Functional Health Literacy<br>Self- Care Index of Heart Failure<br>Family Caregiver Medication Administration Hassles (FCMAHS)	Analyses are the individual results for caregiver health literacy and medication medication administration hassles as the study was insufficiently powered<br>80% of caregivers had adequate health literacy<br>Average FCMAHS score was 27.80 (S.D. = 14.74), with a range of 10-44. Higher scores (out of 100) indicate greater medication administration hassle.

First Author/Year	Diagnosis	Outcome Measure	Result/Findings
Hooley (2005)	HF	Minnesota Living with<br>Heat Failure Questionnaire<br>Beck Depression Inventory II<br>Zarit Caregiver Burden Interview	18% of caregivers had high likelihood of clinical depression<br>Significant correlation between: caregiver burden and caregiver depression scores (r=0.61; p<0.0001), and caregiver burden and patient depression scores (r=0.28; p<0.05)
Hwang (2011)	HF<br>(NYHA I – IV)	Dutch Objective Burden Inventory (English version)<br>Medical Outcome Study Social Support Survey<br>Control Attitudes Scale-Revised<br>Caregiver Reaction Assessment<br>Short Form 36 Health Survey<br>Charlson Co-morbidity Index	Lack of family support for caregivers correlated with the patient's NYHA class, caregivers' relationship to the patient, p=0.003), caregivers' perceived control p <.001) and social support (p <.001)<br>impact of caregiving on caregivers' health was associated with time since the patient's last hospital discharge, (p= .008 caregivers' perceived control, (p = .03) and social support (p <.001)
Hynes (2012)	COPD<br>(NYHA III or IV)	n/a	1) Then and Now<br>2) Awareness of the disease and constant state of anxiety<br>3) The Caring Role<br>4) Perceived illness effects: striving for normalisation and symptom burden<br>5) Support<br>6) Contact with healthcare services
Imes (2011)	HF<br>(NYHA III-IV)	n/a	1) My experience of HF in My Loved One<br>2) Experience with Healthcare providers<br>3) Patient's experience of HF as Perceived by the Partner
Karmilovich (1994)	HF<br>(NYHA III OR IV)	Caregiver Demands Scale<br>(physical demands/role alteration)<br>Brief Symptom Inventory	Significant positive correlation between the number of helping behaviours and the level of stress (r = 0.32, p=0.04)<br>Positive correlation between perceived difficulty performing caring activity and level of stress (r = 0.43, p=0.01)<br>Burden score Mean = 15.27 (SD 11.3)

First Author/Year	Diagnosis	Outcome Measure	Result/Findings
Kitko (2010)	HF	n/a	1) The work of providing care<br>2) Work of living with the illness<br>3) The Work of navigating the system<br>4) The work of maintaining self<br>5) The work of managing the household<br>6) The work of vigilance<br>7) The work of normalcy
Kneeshaw (1999)	CAD	Mutuality Scale<br>Preparedness for Caregiving Scale<br>Recovery Inventory<br>OARS activities of daily living	Significant decrease between the mutuality scale means at hospital discharge and at 3 months ($p < .05$)<br>Preparedness for caring - most common concern of the caregivers was fear of the unknown, especially an emergency
Liljeroos (2014)	HF	n/a	1) Dyads perceive a need for continuous guidance throughout difference phases of the illness trajectory<br>2) Dyads perceive a need to share burden and support with each other and other dyads
Lindqvist (2013)	COPD<br>(Mild to severe)	n/a	(1) Unchanged life situation where no support was needed<br>(2) Socially restricted life and changed roles where support is needed<br>(3) Changes in health where support is needed<br>(4) Changes in the couple's relationship and their need for support.
Loftus (2004)	HF<br>(NYHA II-IV)	Self-reported health status questionnaire<br>State-Trait Anxiety Inventory<br>Pearlin scale of Mastery<br>Rosenberg Self-Esteem Scale<br>Social Support Scale<br>Dyadic Adjustment Scale<br>Caregiver Distress Scale<br>Sense of Competence Questionnaire<br>Centre for Epidemiological Studies depression Scale	Severity of heart failure and level of patient disability predictive of caregiver distress (personal cost, ANCOVA, $F=3.94$, $p<.05$; depression, ANCOVA, $F=7.15$, $p<.05$) independent of caregiver neuroticism<br>Satisfaction with social support was a better predictor of distress than a global measure of social support

First Author/Year	Diagnosis	Outcome Measure	Result/Findings
Lum (2014)	HF<br>(NYHA II-IV)	Zarit Burden Inventory<br>Benefit Finding Scale<br>Patient Health Questionnaire	Higher relationship quality associated with less caregiver burden and more caregiver benefit - ($r = -0.54$, 95% CI: [-0.81, -0.10], $P = 0.02$)
Luttik (2007)	HF	n/a	1) Changes in life<br>2) Anxiety<br>3) Changes in relationship<br>4) Sexuality<br>5) Coping/Support
Luttik (2009)	HF<br>(NYHA II-IV)	Medical Outcome Study<br>General Health Survey (RAND-36)<br>Dutch Objective Burden Inventory<br>Cantril's Ladder	Partners of people with CHF reported lower general health than partners of healthy persons (F (1,598) = 4.066, $P < 0.05$)<br>Interaction effect between group (partners of people with CHF vs. individuals living with a healthy partner) and gender for general well-being, $F(1,581) = 4.526$, $P < 0.05$, and for perceived health change, $F(1,604) = 4.283$, $P < 0.05$
Marcuccilli (2011)	HF –LVAD<br>in situ	n/a	1) Caregiving – a "24/7" Responsibility<br>2) Coping Strategies<br>3) Caregiving satisfaction
Marcuccilli (2014)	HF-LVAD	n/a	1) Advanced Heart Failure is a Life Changing Event<br>2) Self-doubt about LVAD Caregiving Improved with Time<br>3) Lifestyle Adjustments come with Time<br>4) Persistent Worry and Stress<br>5) Caregiving is not a Burden – it's part of life
Miravitlles (2015)	COPD	Survey on Disabilities,<br>Personal Autonomy, and<br>Dependency Situations<br>(National Institute of Statistics in Spain)	35% of caregivers had health problems<br>83% of caregivers had social/leisure problems<br>38% of those of working age had profession related problems

First Author/Year	Diagnosis	Outcome Measure	Result/Findings
Nakken (2017)	COPD	Charlson comorbidity index<br>Dutch relationship questionnaire<br>Care dependency scale<br>Utrecht Coping List<br>Hospital Anxiety and Depression Scale<br>EuroQol-5 Dimensions<br>Medical Outcome Study Social Support<br>Family Appraisal of Caregiving	Depression levels (P=0.261) social support (P=0.222 to 0.897) and caregiver burden (P=0.410 to 0.720) were comparable for male and female caregivers<br>Female caregivers had more symptoms of anxiety (P<0.0001) and worse health status (P=0.001<br>Lower levels of social support were associated with higher levels of caregiver strain<br>No differences in caregiver burden between gender (P=0.401 to 0.724)
Näsström (2017)	HF	Charlson comorbidity index<br>Relatives' perception of quality of care<br>Dutch Objective Burden Inventory<br>EuroQol<br>The Patient Health Questionnaire	1) Adapting to the caring needs and illness trajectory<br>2) Coping with caregiving demands<br>3) Interacting with healthcare providers<br>4) Need for knowledge to comprehend the health situation<br>More information was associated with better perceived health (rho 0.56, P < 0
Park (2013)	CAD	Caregiving Burden Scale	Subscale scores of caregiving demand and caregiving difficulty were 31.1 and 22.9<br>Demanding caregiving activities perceived by caregivers were: providing transportation, additional household tasks, providing emotional support, and two tied for fourth: monitoring symptoms and additional tasks outside the home
Pattenden (2007)	HF	n/a	1) Symptoms, multiple medications, co-morbidities<br>2) Confusion, anxiety and depression<br>3) Adapting life to heart failure<br>4) Changing roles for carers
Pi-Ming Yeh (2012)	HF	JAREL Spiritual Well-being Scale<br>Carers Assessments of Managing Index<br>Care Continuity Scale<br>Caregiver Reaction Assessment<br>Caregivers Esteem Subscale<br>Lack of Family Support Subscale	People expressed medium levels of ADL dependency (Mean = 9.56, SD = 3.93)<br>Higher scores of the older people's ADL dependence (β = 0.47, P < 0.001), lower scores of quality of relationship (β = -0.39, P < 0.01) and higher scores of lack of family support (β = 0.41, P < 0.001) were found to predict significantly greater family caregiver burden

First Author/Year	Diagnosis	Outcome Measure	Result/Findings
Pressler (2013)	HF<br>(NYHA I-IV)	Oberst Caregiving Burden Scale<br>Control Attitudes Scale<br>Patient Health Questionniare-8<br>Brief Symptom Inventory Anxiety Subscale<br>Bakas Caregiving Outcomes Scale<br>Medical Outcomes Study Short Form-12<br>Charlson Comorbidity Index<br>Family Assessment Device	Caregivers perception of life changed - neutral on average and improved from baseline to 4 (P=0.054) and 8 (P= 0.030) months<br>Poor physical and emotional HRQOL at baseline<br>Caregivers of patients with high HF symptoms spent more time on caregiving tasks at 4mo.(P = 0.001) & 8 mo.(P=0.003), perceived greater difficulty with caregiving tasks (baseline P=0.031 & 4 mo. P <0.001) and reported higher bassline anxiety (P=0.019) and poorer physical HRQOL at baseline (P < 0.001) and 4 mo. (P=0.008)
Riegner (1996)	COPD	Caregiver Strain Index<br>Social Support Questionnaire – Short Form<br>Situational Humor Response Questionnaire<br>Coping Humor Scale<br>Life Situation Survey	Role Strain significantly associated with quality of life (β = 0.15, F=11.5, p <.001)<br>For caregivers; social support satisfaction made a significant contribution to quality of life with β= 0.26, F=22.90, p.<.0001, and R^2 of .26
Rolley (2010)	CAD	n/a	1) A gendered approach to health, illness and caring<br>2) Shock, disbelief and the process of adjustment following PCI<br>3) Challenges and changes of the carer-patient relationship<br>4) The needs of the carer: support and information
*Saunders (2009)	HF	CES-D 10<br>Caregiver Reaction Assessment<br>Quality of Life Index	Caregiver burden is associated with lower CG HRQOL; increased burden in support (r=–0.50, p < .01.), finances (r=–0.52 p < .01), schedule (r=–0.62, p < .01) and health (r=–.71, p < .01) = decreased HRQOL
*Saunders (2008)	HF	(as above Saunders, 2009)	76% of caregivers had health problems in past month<br>Highest to lowest burden ranked: schedule (M = 3.20, SD = 1.09), finances (M = 2.39, SD = 1.18), family (M = 2.38, SD = 1.10), and health (M = 2.21, SD = .98).
Saunders (2010)	HF	Centre for Epidemiological Studies Short Depression Scale<br>Quality of Life Index (QLI)	Caregiver depression not difference between employed and unemployed caregivers (t= 1.0, p < 0.05)<br>Employed caregivers perceived higher well-being except for psychological/spiritual domain, well-being (t =-2.9, p<0.01), health/function (t =-2.3, p<0.05), socioeconomic (t = -2.7, p<0.05), family (t =-3.4, P<0.01)

First Author/Year	Diagnosis	Outcome Measure	Result/Findings
Schwarz (2003)	HF	Perceived Stress Scale<br>Center for Epidemiological Stress<br>Studies Depression Scale<br>Inventory of Socially Supportive Behaviours Scale<br>Clinical Assays Gamma Coat<br>Cortisol	The effects model with salivary cortisol was not significant (F=1.9, p=.15).<br>as measured by the PSS, was the only significant variable (t=5.80, p=.000)<br>No significant associations between salivary cortisol and the PSS (F=1.059, p=.384)
Scott (2000)	HF	Caregiver Preparedness Scale<br>Minnesota Living with Health Failure Questionnaire<br>Caregiver Reaction Assessment<br>The Mental Health Inventory-5<br>Quality of Life Index	Caregivers reported being least prepared for stress associated with role (M = 2.11)<br>89% of caregiver scores for mental health were less than age norm<br>55% of the caregivers have to eliminate things from their schedule/interrupt current activities to provide care<br>39% of the caregivers reported constant fatigue
Spence (2008)	COPD (Adv.)	n/a	1) Impact of family caregiving<br>2) Unmet Support Needs<br>3) Carers' perceptions of patients
Strom (2015)	HF	n/a	1) Being on the alert<br>2) Being a forced volunteer
Takata (2008)	COPD	Zarit Burden Interview<br>(Japanese version)<br>Barthel Index	53.3% of caregivers were depressed<br>Caregivers providing longer term care were more burdened<br>HB caregivers want to use social services such as Nursing Home 90.9% v 34.8% (p<0.05)
Vellone (2015)	HF<br>(NYHA I-IV)	Caregiver Contribution to<br>Self-care of heart failure Index	54.2% of caregivers were unable to recognize the signs and symptoms of a heart failure exacerbation quickly or very quickly
Wallin (2013)	CAD	n/a	1) Difficulties managing a changed life situation<br>2) Feeling like I come second<br>3) Feeling new hope for the future
Woolfe (2007)	COPD	Family Caregiver<br>Needs Survey	Information is the most important need for caregivers (M= 6.37, SD = 0.8<br>Important but poorly met needs = information (27.7), psychological (26.5) and personal (25.3)

First Author/Year	Diagnosis	Outcome Measure	Result/Findings
Wingham (2015)	HF	n/a	1) Providing support<br>2) Transition to becoming a caregiver<br>3) Engaging help

HF = Heart Failure, COPD = Chronic Obstructive Pulmonary Disease, LVAD = Left Ventricular Assist Device, LVAD DT = Left Ventricular Assist Device Destination Therapy, CAD = Coronary Artery Disease, n/a = not applicable, PCI = Percutaneous coronary intervention, MLWHFQ = Minnesota Living with Heart Failure Questionnaire

*** = same study**
