# Supplementary material for: ’Who Cares?' The experiences of caregivers of adults living with heart failure, chronic obstructive pulmonary disease and coronary artery disease: a mixed methods systematic review
Source: BMJ Open. 2018 Jul 11;8(7):e020927. doi: 10.1136/bmjopen-2017-020927 (PMC6082485; doi:10.1136/bmjopen-2017-020927)
Supplement: Author's manuscript [file bmjopen-2017-020927.draft_revisions.pdf]

BMJ Open is committed to open peer review. As part of this commitment we make the peer review history of every article we publish publicly available.

When an article is published we post the peer reviewers' comments and the authors' responses online. We also post the versions of the paper that were used during peer review. These are the versions that the peer review comments apply to.

The versions of the paper that follow are the versions that were submitted during the peer review process. They are not the versions of record or the final published versions. They should not be cited or distributed as the published version of this manuscript.

BMJ Open is an open access journal and the full, final, typeset and author-corrected version of record of the manuscript is available on our site with no access controls, subscription charges or pay-per-view fees (http://bmjopen.bmj.com).

If you have any questions on BMJ Open's open peer review process please email info.bmjopen@bmj.com

BMJ Open

"Who Cares?" The Experiences of Caregivers of Adults Living with Heart Failure, Chronic Obstructive Pulmonary Disease and Coronary Artery Disease – A Mixed Methods Systematic Review

Journal:	*BMJ Open*
Manuscript ID	bmjopen-2017-020927
Article Type:	Research
Date Submitted by the Author:	01-Dec-2017
Complete List of Authors:	Noonan, Miriam; University of Exeter Medical School, European Centre for Environment and Human Health<br>Wingham, Jennifer; Royal Cornwall Hospitals NHS Trust, Research, Development and Innovation; University of Exeter Medical School,<br>Taylor, Rod; University of Exeter, Peninsula Medical School
Keywords:	Caregivers, Heart failure < CARDIOLOGY, Coronary heart disease < CARDIOLOGY, Chronic obstructive pulmonary disease, mixed methods systematic review

SCHOLARONE™ Manuscripts

For peer review only

“Who Cares?” The Experiences of Caregivers of Adults Living with Heart Failure, Chronic Obstructive Pulmonary Disease and Coronary Artery Disease – A Mixed Methods Systematic Review

M Noonan[1], J Wingham[2,3] , RS Taylor[4]

Author Affiliations

1. Miriam Noonan University of Exeter Medical School, European Centre for Environment and Human Health, Knowledge Spa, Royal Cornwall Hospital, Truro, Cornwall, UK TR13HD, mn354@exeter.ac.uk

2. Dr. Jennifer Wingham Royal Cornwall Hospitals NHS Trust, Research, Development and Innovation, F37, Knowledge Spa, Royal Cornwall Hospital, TRURO, Cornwall, UK TR1 3HD,
3. University of Exeter Medical School,
Exeter, UK EX2 4SG

4. Professor Rod S. Taylor, University of Exeter, Peninsula Medical School, Exeter, Cornwall, UK

Correspondence to
Miriam C. Noonan; mn354@exeter.ac.uk

(Word Count, 4,290)

For peer review only - http://bmjopen.bmj.com/site/about/guidelines.xhtml

ABSTRACT

Objective To assess the experiences of unpaid caregivers providing care to people with heart failure (HF) or chronic obstructive pulmonary disease (COPD) or coronary artery disease (CAD).

Design Mixed methods systematic review including qualitative and quantitative studies.

Data Sources Databases searched: Medline Ebsco, PsychInfo, CINAHL Plus with Full Text, Embase, Web of Science, Ethos: The British Library and ProQuest. Grey literature identified using: Global Dissertations and Theses and Applied Sciences Index and hand searches and citation checking of included references. Search time frame: 1st January 1990 to 30th August 2017.

Eligibility criteria for selecting studies Inclusion was limited to English language studies in unpaid adult caregivers (>18 years), providing care for patients with HF, COPD and CAD. Studies that considered caregivers for any other diagnoses and studies undertaken in low and middle income countries were excluded. Quality assessment of included studies was conducted by two authors.

Data analysis/synthesis - A data based convergent synthesis was conducted

Results Searches returned 8,024 titles and abstracts. 54 studies - 21 qualitative, 32 quantitative, and 1 mixed were included. This totalled 26,453 caregivers who were primarily female (63 %), with average age of 62.2 years. Narrative synthesis yielded six concepts related to caregiver experience: (1) mental health (2) caregiver role (3) lifestyle change (4) support for caregivers (5) knowledge and (6) relationships. There was a discordance between paradigms regarding emerging concepts. Four concepts emerged from qualitative papers which were not present in quantitative papers (1) expert by experience (2) vigilance (3) shared care and (4) time.

Conclusion Caregiving is life altering and complex with significant health implications. Health professionals should support caregivers who in turn can facilitate the recipient to manage their long term condition. Further longitudinal research exploring the evolution of caregiver experiences over time of patients with chronic cardiopulmonary conditions is required.

For peer review only - http://bmjopen.bmj.com/site/about/guidelines.xhtml

Strengths and limitations of this study

- This mixed methods systematic review provides the opportunity for a broadened and deeper understanding of the literature on the experiences of unpaid caregivers' providing care to people with heart failure, chronic obstructive pulmonary disease, and coronary artery disease.
- This review provides an integration of qualitative and quantitative predictors of caregiver experiences.
- To maximise applicability we included studies from higher income countries only.
- Quality of evidence limited by assessment of caregiver experience at single point of time and there is need for future studies that employ longitudinal or repeated measures design.

Systematic review registration The study protocol was registered on PROSPERO (CRD42016053412).

For peer review only

For peer review only - http://bmjopen.bmj.com/site/about/guidelines.xhtml

INTRODUCTION

Unpaid caregiving is a global occurrence; there are approximately 43.5 million in the United States, 2.86 million in Australia and 6.5 million in the United Kingdom (UK) unpaid caregivers.[1] From 2001 to 2011 the number of unpaid caregivers in the UK grew at a faster rate than population growth.[2] The annual value of care provided by family members or a friend to an individual with a chronic illness is estimated to be £132billion,[3] almost equivalent to the total NHS spend of £134billion.[3]

The Institute of Public Care examining a Caregivers' Life (2017) described caregivers as becoming the 'Skilled Helper' performing a series of roles. A longitudinal study on caregiving by Seltzer & Wailing (2000), identify a dynamic process of transitions to becoming a caregiver in what they call the "caregiving career." Considering these transitions and the dynamic process of caring it is imperative for caregivers to receive a carer needs assessment as legally stipulated by the 2014 Care Act.[6] Additionally, the National Institute for Health and Care Excellence (NICE) clinical guidelines for heart failure (HF) (CG108) and chronic obstructive pulmonary disease (COPD) (CG101) both recommend that family members or caregivers are provided with support and included in discussions about care.

Cardio-pulmonary disease is a primary cause of illness. Cardiovascular disease is responsible for 45% of all deaths in Europe[9] and the cause of 1 in 4 deaths in the United States.[10] By the year 2020, COPD is projected to be in the global top five of diagnoses causing years lost through early mortality or disability-adjusted life years.[11] COPD and cardiovascular disease frequently co-exist.[12, 13] There are systematic reviews examining needs and experiences in HF caregiving[14, 15, 16] and COPD caregiving.[17, 18] However, we know of no systematic review that synthesises quantitative and qualitative studies to bring together caregivers' experiences of people with HF, COPD and coronary artery disease (CAD).

Using a mixed methods systematic review methodology that includes both qualitative and quantitative literature, this study aims to understand the experiences of adult caregivers when supporting people with HF, COPD and CAD.

METHODS

We conducted and reported this systematic review in accordance with the PRISMA (Preferred Reporting Items for Systematic Reviews and Meta-Analyses) statement.[19]

Study design

This study employed a mixed methods systematic review assessing both qualitative and quantitative studies.[20] The rationale for using a mixed methods review approach was multifaceted. Firstly, to

For peer review only - http://bmjopen.bmj.com/site/about/guidelines.xhtml

gain a qualitative assessment of the quantitative predictors of experiences of caregivers. Secondly, to develop a more holistic perspective of what caregiver's experience. Third, we wanted to assess the degree of convergence between qualitative and quantitative experiences.

Search Strategy

Our search strategy was designed in conjunction with an experienced Health Services Librarian and Information Specialists. Search terms included condition specific terms, i.e., "heart failure", "COPD" and "coronary artery disease", carer specific, plus experience related terms including, "experience", "quality of life" and "routine". (See supplementary table 1 for complete list of search terms.) Databases searched included: Medline Ebsco, PsychInfo, CINAHL Plus with Full Text, Embase, Web of Science, Ethos: The British Library and ProQuest. Grey literature was identified using Global Dissertations and Theses and Applied Sciences Index and hand searches and citation checking of included references. To ensure the contemporary nature of the evidence considered, the search time frame was January 1990 to August 2017. A single researcher (MN) initially screened titles and abstracts. Selection of full papers was performed by two researchers (MN and either JW or RST).

Study Selection

Quantitative and qualitative methods studies were included if they addressed 'caregiver experience' which was defined as encompassing the daily activities of caregivers and the impact of these activities on their lives. Inclusion was limited to English language studies in unpaid adult caregivers (>18 years), providing care for patients with HF, COPD and CAD. As we sought to inform the practice of UK and other high income countries we excluded studies undertaken in low and middle income countries (http://iccmoot.com/wp-content/uploads/2017/07/World-Bank-List-of-Economies.pdf).[21]

Data extraction

Data extracted from retained studies included: study design, population, study description, method, findings, discussion and author's conclusions. Caregiver quotes were extracted from qualitative studies. For quantitative studies, data extraction also included details of attrition, data analysis, and authors stated study limitations.

Study quality assessment

Qualitative studies were appraised utilising the Critical Appraisal (CASP) Tool for qualitative studies (http://docs.wixstatic.com/ugd/dded87_25658615020e427da194a325e7773d42.pdf).[22] In absence of an existing quality tool that could be used to appraise the quantitative studies addressing the

For peer review only - http://bmjopen.bmj.com/site/about/guidelines.xhtml

specific question of this study, a quality assessment tool was developed based on what were deemed to be the appropriate core biases. i.e. (1) was the sample recruited appropriately? (2) Was the level of attrition acceptable? (3) Was a validated quantitative outcome(s) used? (4) Were the methods of data analysis appropriate? A score of 1 was attributed to each study based on these four questions. Based on their quality assessment, studies were scored and ranked; 1 or 2 “low quality”, 3 “medium quality” and 4 or 5 “high quality”. Only high quality qualitative studies were included for synthesis.[23] It is difficult to apply a set of checklists when appraising qualitative research, although it is imperative to have criteria to appraise qualitative papers. Dixon-Woods (2001) reports; to synthesise qualitative data exclusion of studies of insufficient quality is important. This contributes to a synthesis of evidence which will facilitate obtaining a summary of evidence. Data Extraction and quality appraisal was first conducted by a single researcher (MN) and then checked by one of two researchers (JW or RST).

Data analysis and synthesis

The methods of mixed methods data synthesis is an emerging one and no single approach has yet been universally accepted.[25] In this study a data based convergent design[26] was chosen as it represents the most common type of synthesis used for mixed method reviews.[27] This requires the transformation of one methodology into another. Due to the heterogeneity of the quantitative methods employed across papers a meta-analysis was not appropriate. Instead, applying a narrative profile formation, quantitative data was converted into qualitative data.[28] Table 1 provides an overview of this. This resulted in two qualitative data sets[29] that were compared and concepts emerged. A mapping table was completed in order to provide an audit trail of how the overall concepts across all papers were derived (see supplementary file 2a and 2b). Initial synthesis was conducted by a single researcher (MN) and corroborated by two experienced researchers in quantitative (RST) and qualitative (JW) research.

For peer review only - http://bmjopen.bmj.com/site/about/guidelines.xhtml

Table 1

Overview of Narrative Profile Formation

Profile	Explanation	Emerged from Quantitative Studies
Modal Profile	Description of the group i.e. most frequently occurring characteristics	Primarily female, Caucasian co-habiting caregivers living an altered life course
Average Profile	The mean number of attributes of the individuals or situation	Engaging in a multitude of ADL tasks, to assist the recipient has a significant impact on caregiver mental health e.g. "(r = 0.32, p=0.04); significant positive correlation between the number of helping behaviours and the level of stress, positive correlation between perceived difficulty performing cg activity and level of stress (r = 0.43, p=0.01)"
Comparative Profile	Differences/similarities in comparing profiles	The quality of relationship between cg. and recipient influenced cg's perception of their role e.g. "Higher relationship quality was associated with less caregiver burden and more caregiver benefit - (r = -0.54, 95% CI: [-0.81, -0.10], P = 0.02)". & "emotional well-being of caregivers is associated with the emotional well-being of HF patients" (Factors Associated With Patients' Emotional Well-being = Caregivers' mental health score: Adjusted R2 = 0 .536, F = 40.299, p = .000)".
Holisitc Profile	Overall impressions of investigator	Cg's supported, informed and involved by healthcare teams have more positive experiences e.g. "Partners scored 94% of the max score for received information, 92% of the max score for participation in care, 83% of the max score for contact and 80% of the max score for overall satisfaction with participation in care."

For peer review only - http://bmjopen.bmj.com/site/about/guidelines.xhtml

RESULTS

Study selection

The study selection process is summarised in a PRISMA Flow diagram shown in Figure 1. Following removal of duplicates the search strategy yielded a total of 8,024 title and abstracts. Of these 241 full papers were reviewed, of which 57 papers (54 studies) were included for synthesis. A detailed summary of included studies is provided in Table 2.

PRISMA Flow Diagram Figure 1

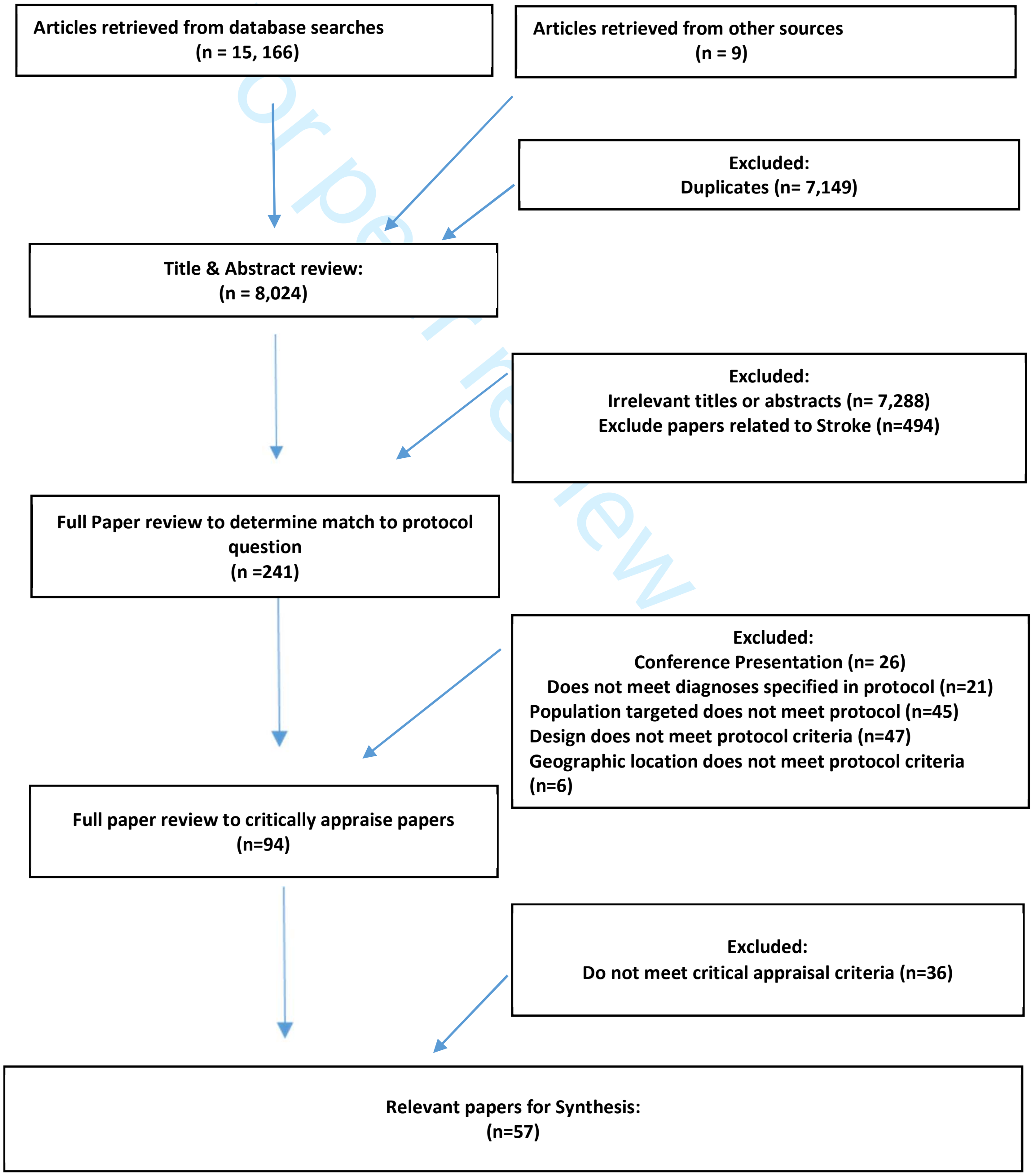

For peer review only - http://bmjopen.bmj.com/site/about/guidelines.xhtml

Table 2

Summary of included studies

First Author/Year	Diagnosis	Primary Focus	Methods	Country	Data Collection Method	Sampling	Participants (Carers)	Mean Age (Carers)
Agren (2010)	HF (NYHA II-IV)	Levels and predictors of Cg. burden	QUANT (Correlational)	SWEDEN	Cross Sectional	Purposive	135 (101F, 34M)	69
Al-Rawashdeh (2017)	HF (NYHA I-IV)	Sleep Disturbance and QOL in Dyads	QUANT	USA	Cross Sectional	Purposive	78 Dyads (58F, 20M)	N/S
Andersen (2013)	HF	Knowledge and exp. of cg's Cg's competence and support needed	QUAL	NORWAY	Interviews	Convenience	19 (17F, 2M)	63
Badr (2017)	COPD	Predictors of pt. & cg depression	QUANT	USA	Cohort Study	Purposive	89 (68F, 21M)	54.8
Bakas (2006)	HF (NYHA II-IV)	Perceived control and mgt. of HF by Cg's Difficulty of cg. tasks	QUANT (Descriptive Correlational)	USA	Cross Sectional	Convenience	21 (20F, 1M)	59.6
Baker (2010)	HF (LVAD in situ)	Exp. of cg's of LVAD pt's at home	QUAL (Descriptive)	USA	Interviews (Phenomenological)	Convenience	6 (5F, 1M)	51
Bove (2016)	COPD (GOLD C&D)	Exp. of Cg Role	QUAL	Denmark	Focus Groups	Purposive	22 (13F, 9M)	69.4
Burke (2014)	HF (NYHA II-IV)	Cg's perception of their role compared with HCP's perception	QUAL (Inductive)	USA	Interviews	Purposive	20 (18F, 1M, 1N/S)	59

For peer review only - http://bmjopen.bmj.com/site/about/guidelines.xhtml

Summary of included studies

First Author/Year	Diagnosis	Primary Focus	Methods	Country	Data Collection Method	Sampling	Participants (Carers)	Mean Age (Carers)
Chung (2016)	HF (NYHA II-IV)	Cg. outcomes when caring for depressed and non-depressed HF pt.'s	QUANT	USA	Cross Sectional	Convenience	102 Dyads (79F, 23M)	56.7
Clark (2007)	HF (NYHA II – IV)	Complexity of caregiving	QUAL	SCOTLAND	Interviews	Convenience	30 (23F, 7M)	68F, 67M
Cossette (1993)	COPD (GOLD III-V)	Relationship between cg tasks and Mental Health of cg's Influence of social support	QUANT	CANADA	Cross Sectional	Convenience	89 (F)	65
Evangelista (2002)	HF (NYHA I – IV)	Emotional wellbeing of HF pts and carers. Associated factors and gender differences in emotional wellbeing	QUANT (Descriptive) (Correlational)	USA	Cross Sectional	Convenience	103 Dyads (73F, 30M)	59.4
Figueiredo (2013)	COPD (GOLD I-IV)	Coping strategies of family cg's Impact on health	QUANT (Correlational)	PORTUGAL	Cross Sectional	Convenience	158 (120F, 38M)	58.3(early COPD) 60.8 (Adv. COPD)
Figueiredo (2014)	COPD (GOLD I-IV)	Subjective burden of family cg's of early and advanced COPD pts. Predictor variables of same	QUANT (Correlational)	PORTUGAL	Cross Sectional	Convenience	167 (125F, 42M)	58.3(early COPD) 60.5 (Adv. COPD)
Figueiredo (2015)	COPD (Mod – Severe)	Exp. of husbands and sons providing care to a family member	QUAL	PORTUGAL	Interviews	Purposive	12 (M)	70.9(Husbands) 43.4 (Sons)

For peer review only - http://bmjopen.bmj.com/site/about/guidelines.xhtml

Summary of included studies

First Author/Year	Diagnosis	Primary Focus	Methods	Country	Data Collection Method	Sampling	Participants (Carers)	Mean Age (Carers)
Grigorovich (2017)	HF (NYHA II-IV)	Emotional wellbeing of cg's	QUANT	CANADA	Longitudinal (repeated measures)	Convenience	50 (31F, 19M)	58
*Halm et al. (2006)<br>*Halm et al. (2007)	CAD	Cg burden in CABG pts.<br>Cg HRQOL outcomes<br>12 months post CABG	QUANT	USA	Cross Sectional	Convenience	166(136F, 30M)	64.7
*Halm (2016)<br>*Halm (2017)	CAD	Understand concerns exp. and needs of Cg's<br>3 mths. post CABG<br>Gender differences	QUAL	USA	Interviews	Purposive	32(16F, 16M)	60.6 (M<70)<br>61.5 (F<70)<br>75.9(M>70), 73.6(F>70)
Hess (2009)	HF	Association between Pt. literacy and self-care<br>Association between Cg. literacy and medication administration<br>Demographic variable of health literacy	QUANT (Correlational)	USA	Cross Sectional	Convenience	5 (F)	65
Hooley (2005)	HF (NYHA III or IV)	Cg burden, disease severity and depression in pts. and cg's.	QUANT	CANADA	Cohort Study	Convenience	50 (40F, 10M)	61
Hwang (2011)	HF (NYHA I – IV)	Perceived health and impact on family cg's<br>Factors associated with impact of caring<br>Effect of social support	QUANT	USA	Cross Sectional	Convenience	76 Dyads (54F, 22M)	53.4

For peer review only - http://bmjopen.bmj.com/site/about/guidelines.xhtml

Summary of included studies

First Author/Year	Diagnosis	Primary Focus	Methods	Country	Data Collection Method	Sampling	Participants (Carers)	Mean Age (Carers)
Hynes (2012)	COPD	Exp. of cg's providing care	QUAL	IRELAND	Interviews (Phenomenological)	Convenience	11(9F, 2M)	N/S
Imes (2011)	HF (NYHA III-IV)	Cg's view of HF Understanding of advanced directives Gg's coping/mgt. of HF	QUAL (Descriptive)	USA	Interviews	Convenience	14(11F, 3M)	64.8
Karmilovich (1994)	HF (NYHA III OR IV)	Components of caring. Assess stress and cg burden	QUANT (Correlational)	USA	Descriptive survey	Purposive	41 (30F, 11M)	56.7
Kitko (2010)	HF	Type of work involved in caregiving Generate middle range Theory of spousal caring	QUAL	USA	Interviews	Convenience	20(14F, 6M)	67
Kneeshaw (1999)	CAD	Cg mutuality and preparedness post CABG surgery Relationship with Recovery outcomes of elderly women post CABG	QUANT	USA	Cohort Study	Convenience	49 (32F, 17M)	50.1
Liljeroos (2014)	HF	Perceived caring needs in dyads	QUAL	SWEDEN	Focus Groups	Convenience	19Dyads (7F, 12M)	70
Lindqvist (2012)	COPD (Mild to severe)	Conceptions of daily life in female carers	QUAL (Descriptive)	SWEDEN	Interviews	Purposive	21(F)	72

For peer review only

For peer review only - http://bmjopen.bmj.com/site/about/guidelines.xhtml

Summary of included studies

First Author/Year	Diagnosis	Primary Focus	Methods	Country	Data Collection Method	Sampling	Participants (Carers)	Mean Age (Carers)
Loftus (2004)	HF (NYHA II-IV)	Outcomes of caregiving in HF	QUANT (Correlational)	UK	Cross Sectional	Convenience	53 (41F, 12M)	66.7
Lum (2014)	HF (NYHA II-IV)	Relationship quality. Impact of this on cg Influence of depressive symptoms	QUANT (Correlational)	USA	Cross Sectional	Purposive	19 (7F, 12M)	59
Luttik (2007)	HF	Exp. of cg's Cg wellbeing factors	QUAL	NETHERLANDS	Interviews	Convenience	13(10F, 3M)	66
Luttik (2009)	HF (NYHA II-IV)	QOL in cg's vs QOL in people with healthy partners	QUANT	NETHERLANDS	Cross Sectional	Purposive	607 (total) (303 cg's)	67(cg's)
Marcuccilli (2011)	HF –LVAD in situ	Life adjustments of cg's caring for LVAD pt.	QUAL	USA	Interviews (Phenomenological)	Convenience	5 (5F)	56.6
Marcuccilli (2014)	HF-LVAD as DT	Exp. of caring for family member with an LVAD-DT	QUAL	USA	Interviews (Phenomenological)	Purposive	7(6F, 1M)	65
Miravitlles (2015)	COPD	Burden of cg's	QUANT	SPAIN	Survey	Representative	22,795	56.5
Nakken (2017)	COPD	Gender diffs in cg's and their perception of pts.	QUANT (Correlational)	Netherlands	Cross Sectional	Convenience	188 Dyads (103F, 85M)	65.4F 65.1M
Näsström (2017)	HF	Cg's participation & perspective of home care services	MIXED METHOD	Sweden	Interviews	Purposive Repeated Measures	15 (11F,4M)	77

For peer review only - http://bmjopen.bmj.com/site/about/guidelines.xhtml

Summary of included studies

First Author/Year	Diagnosis	Primary Focus	Methods	Country	Data Collection Method	Sampling	Participants (Carers)	Mean Age (Carers)
Park (2013)	CAD	Difficulty of cg tasks for older cg's of CABG pt.'s	QUANT	USA	Cross Sectional	Convenience	35(29F, 6M)	60
Pattenden (2007)	HF	Impact and exp. of living with HF for pts and carers<br>Understand needs of this population	QUAL	UK	Interviews	Purposive	20(18F, 2M)	67.8
Pi-Ming Yeh (2011)	HF	Older pt. ADL dependency<br>Influences of factors on cg burden<br>Resiliency model	QUANT (Correlational)	USA	Cross Sectional	Purposive	50 (35F, 15M)	60.3
Pressler (2013)	HF (NYHA I-IV)	Changes in Cg. burden and HRQOL<br>Differences in cg's perceptions between NYHA classes<br>Time spent on cg tasks	QUANT	USA	Prospective Cohort Study	Convenience	65(48F, 17M)	59.7
Riegner (1996)	COPD	Exp. of QOL, support, role strain and humour in cg's and pt.'s. with COPD	QUANT (Correlational)	USA	Cross Sectional	Convenience	83(Dyads) (50F, 33M)	63.2

For peer review only

For peer review only - http://bmjopen.bmj.com/site/about/guidelines.xhtml

Summary of included studies

First Author/Year	Diagnosis	Primary Focus	Methods	Country	Data Collection Method	Sampling	Participants (Carers)	Mean Age (Carers)
Rolley (2010)	CAD	Exp. of carers of pt.'s undergoing PCI	QUAL	AUSTRALIA	Focus Groups	Convenience	18 (F)	N/S
*Saunders (2009)<br>*Saunders (2008)	HF<br>(NYHA I-IV)	Cg burden, HRQOL, Depression, illness severity<br>Cg burden and cg characteristics	QUANT (Correlational)	USA	Cross Sectional	Purposive	50 (42 F, 8M)	58.1
Saunders (2010)	HF	Compare employed and unemployed cg.'s<br>Cg outcomes and pt. characteristics	QUANT	USA	Cross Sectional	Convenience	41 (37F, 4M)	59(Unemp.)<br>52(Emp.)
Schwarz (2003)	HF	Impact of support for cg's<br>Assess cortisol levels, high in stressed cg.'s	QUANT	USA	Cross Sectional<br>Saliva Sample	Convenience	75 (55F, 20M)	63
Scott (2000)	HF	HRQOL of cg's<br>Cg readiness to care<br>HRQOL of HF pts.<br>Biopsychosocial exp. of caring	QUANT	USA	Cross Sectional	Purposive	18 (16F, 2M)	63
Spence (2008)	COPD (Adv.)	Needs and exp. of family cg's	QUAL (Descriptive)	N.IRELAND	Interviews	Purposive	7 (6F, 1M)	N/S

For peer review only - http://bmjopen.bmj.com/site/about/guidelines.xhtml

Summary of included studies

First Author/Year	Diagnosis	Primary Focus	Methods	Country	Data Collection Method	Sampling	Participants (Carers)	Mean Age (Carers)
Strom (2015)	HF	NOK's exp. and responsibilities when caring	QUAL	NORWAY	Interviews	Convenience	19 (17F, 2M)	Median 63
Takata (2008)	COPD (Long term O2 Therapy)	Cg burden	QUANT	JAPAN	Cross Sectional	Convenience	45 Dyads (37F, 8M)	68
Vellone (2015)	HF (NYHA I-IV)	Cg self-efficacy and contribution to self-care impact of pt. illness and cg characteristics on cg role	QUANT (Correlations)	ITALY	Cross Sectional	Convenience	515 Dyads (270F, 245M)	56.6
Wallin (2013)	CAD	Cg's needs for support and info. Impact on daily life	QUAL (Descriptive)	SWEDEN	Interviews	Purposive	20 (14F, 6M)	55
Woolfe (2007)	COPD	Cg needs Cg wellbeing	QUANT (Descriptive)	AUSTRALIA	Cross Sectional	Convenience	63(39F, 24M)	N/S
Wingham (2015)	HF	Needs of cg's Inform intervention	QUAL	UK	Interviews Focus Groups	Purposive	22 (16F, 6M)	67

***Primary Focus refers to the aims of the papers as identified by the authors.**
The data extracted from each paper as part of this systematic review may reflect a different focus.
Data extracted in this review pertains specifically to carers experiences.
Exp/exp = Experience
Cg. = Caregiver
***= Same study**

For peer review only - http://bmjopen.bmj.com/site/about/guidelines.xhtml

Study characteristics

Of the 54 studies, 21 were qualitative, 32 quantitative and 1 mixed methods. Of these, 34 focused on HF, 14 COPD and 6 CAD. Across all studies the total number of caregiver participants was 26,453. The majority of caregivers included in studies were female (63%), with an average age of 62 years. A summary of study characteristics is provided in Table 3.

Table 3

Study Characteristics

**Summary of Study Characteristics**	**(n = 54 Studies)**	
**Aetiology of patients**		
CAD	6	
HF	34	
COPD	14	
**Caregiver Participants**		**Patients ***
Median age (range)	62 (43-77)	68 (36-93)
Median % of female (range)	63% (5–270)	38% (1-229)
**Continents of publication**		
Europe	22	
North America	29	
Australasia	3	
**Date of publication**	**(n= 57 Publications)**	
1990-1995	2	
1996-2001	2	
2002-2007	10	
2008-2013	24	
2014-2017	19	
**Type of study**		
Qualitative	21	
Quantitative	32	
Mixed	1	

CAD – Coronary Artery Disease
HF – Heart Failure
COPD – Chronic Obstructive Pulmonary Disease

***Patient Data based on data collected in 34 studies**

For peer review only - http://bmjopen.bmj.com/site/about/guidelines.xhtml

Quality assessment

As suggested by Dixon-Woods (2001) regarding exclusion of studies of insufficient quality, qualitative papers were appraised and the following rationale was agreed by the research team. High quality studies were included and if low or medium quality studies produced a different insight or value to those deemed high quality they too were included. All 21 qualitative studies were classified as high quality. Quantitative studies were quality appraised classified as follows; 3 high quality, 12 medium quality and 17 low quality. See Table 4(a) and 4(b) for quality appraisal tables.

For peer review only

For peer review only - http://bmjopen.bmj.com/site/about/guidelines.xhtml

Table 4(a)

Quality Appraisal – Qualitative Papers

	**Design**	**Recruitment**	**Data Collection**	**Data Analysis**	**Findings**	**Total**
Andersen (2013)	1	1	1	0	1	**4 [H]**
Baker (2010)	1	0	1	1	1	**4 [H]**
Bove (2016)	1	1	1	1	1	**5 [H]**
Burke (2014)	1	1	1	1	1	**5 [H]**
Clark (2007)	1	1	1	1	0	**4 [H]**
Figueiredo (2015)	0	1	1	1	1	**4 [H]**
•Halm (2016)	1	1	1	1	1	5 [H]
•Halm (2017)	1	1	1	1	1	**5 [H]**
Hynes (2012)	1	1	1	0	1	**4 [H]**
Imes (2011)	1	1	1	1	1	**5 [H]**
Kitko (2010)	1	1	1	1	1	**5 [H]**
Liljeroos (2014)	1	1	1	1	1	**5 [H]**
Lindqvist (2012)	1	1	1	1	1	**5 [H]**
Luttik (2007)	1	1	1	1	1	**5 [H]**
Marcuccilli (2011)	1	0	1	1	1	**4 [H]**
Marcuccilli (2014)	1	1	1	1	1	**5 [H]**
*Näsström (2017)	1	1	1	1	1	**5 [H]**
Pattenden (2007)	1	1	1	1	0	**4 [H]**
Rolley (2010)	1	1	1	1	0	**4 [H]**
Spence (2008)	1	0	1	1	1	**4 [H]**
Strom (2015)	1	1	1	1	1	**5 [H]**
Wallin (2013)	1	0	1	1	1	**4 [H]**
Wingham (2015)	1	1	1	1	1	**5 [H]**

Quality criteria applied as per CASP Qualitative Checklist:
http://docs.wixstatic.com/ugd/dded87_29c5b002d99342f788c6ac670e49f274.pdf
H = High Quality. 4/ 5 out of 5 quality criteria achieved
M= Medium Quality. 3 out of 5 quality criteria achieved
L= Low Quality. 1 or 2 out of 5 quality criteria achieved
•= same study ***Mixed methods study – qualitative component**

For peer review only - http://bmjopen.bmj.com/site/about/guidelines.xhtml

Table 4(b)

Quality Appraisal – Quantitative Papers

First Author	Study design	Participant sampling	Participant attrition	Outcome Measures	Data Analysis	Overall score
Agren (2010)	CS	Purp [+1]	0% [+1]	Non-V	MV [+1}	**3 [M]**
Al-Rawashdeh (2017)	CS	Purp [+1]	NS	V [+1]	MV [+1]	**3 [M]**
Badr (2017)	CS	Con	15.5% [+1]	Non-V	MV [+1]	**2 [L]**
Bakas (2006)	CS	Con	NS	V [+1]	MV [+1]	**2 [L]**
Chung (2016)	CS	Con	NS	V [+1]	UV	**1 [L]**
Cossette (1993)	CS	Con	NS	V [+1]	MV [+1]	**2 [L]**
Evangelista (2002)	CS	Con	20% [+1]	V [+1]	MV [+1]	**3 [M]**
Figueiredo (2013)	CS	Con	17% [+1]	V [+1]	MV [+1]	**3 [M]**
Figueiredo (2014)	CS	Con	11% [+1]	Non-V	MV [+1]	**2 [L]**
Grigorovich (2017)	LS [+1]	Con	NS	V [+1]	MV [+1]	**3 [M]**
• Halm (2006)	CS	Con	64%	V [+1]	MV [+1]	**2 [L]**
• Halm (2007)	CS	Con	64%	V [+1]	MV [+1]	**2 [L]**
Hess (2009)	CS	Con	NS	V [+1]	MV [+1]	**2 [L]**
Hooley (2005)	CS	Con	0% [+1]	V [+1]	UV	**2 [L]**
Hwang (2011)	CS	Con	35%	V [+1]	MV [+1]	**2 [L]**
Karmilovich (1994)	CS	Purp [+1]	24%	V [+1]	MV [+1]	**3 [M]**
Kneeshaw (1999)	LS [+1]	Con	32.7%	V [+1]	MV [+1]	**3 [M]**
Loftus (2004)	LS [+1]	Con	36%	V [+1]	MV [+1]	**3 [M]**
Lum (2014)	CS	Purp [+1]	5% [+1]	V [+1]	MV [+1]	**4 [H]**
Luttik (2009)	CS	Purp [+1]	31%	Non-V	MV [+1]	**3 [M]**
Miravitlles (2015)	CS	Rand [+1]	0% [+1]	Non-V	MV [+1]	**3 [M]**
Nakken (2017)	CS	Con	58%	Non-V	MV [+1]	**1 [L]**
*Näsström (2017)	LS [+1]	Purp [+1]	7% [+1]	V [+1]	MV [+1]	**5 [H]**
Park (2013)	CS	Con	NS	V [+1]	UV	**1 [L]**

For peer review only - http://bmjopen.bmj.com/site/about/guidelines.xhtml

Pi-Ming Yeh (2011)	CS	Purp [+1]	39%	V [+1]	MV [+1]	**4 [H]**
Pressler (2013)	LS [+1]	Con	16% [+1]	V [+1]	MV [+1]	**4 [H]**
Riegner (1996)	CS	Con	71.8%	V [+1]	MV [+1]	**2 [L]**
•Saunders (2008)	CS	Purp [+1]	36%	V [+1]	MV [+1]	**3 [M]**
•Saunders (2009)	CS	Purp [+1]	36%	V [+1]	MV [+1]	**3 [M]**
Saunders (2010)	CS	Con	NS	V [+1]	UV	**1 [L]**
Schwarz (2003)	CS	Con	NS	V [+1]	MV [+1]	**2 [L]**
Scott (2000)	CS	Purp [+1]	10% [+1]	Non-V	MV [+1]	**3 [M]**
Takata (2008)	CS	Con	NS	V [+1]	UV	**1 [L]**
Vellone (2015)	CS	Con	NS	V [+1]	MV [+1]	**2 [L]**
Woolfe (2007)	CS	Con	37%	V [+1]	UV	**1 [L]**

Study design - CS: cross-sectional design; LS: longitudinal design
Participant sampling – Purp: purposive; Rand: random; Cons: consecutive; Con: convenience; NS; not stated/unclear
Attrition – 20% or less = +1; NS: Not Stated
Outcome measures – V: all validated outcomes; non-V: some or all non-validated; NS: not stated/unclear
Data analysis – MV: multivariate; UV: univariate
•= same study ***Mixed method study – quantitative component**

Overall quality rating
H = High Quality. 4/ 5 out of 5 quality criteria achieved
M= Medium Quality. 3 out of 5 quality criteria achieved
L= Low Quality. 1 or 2 out of 5 quality criteria achieved

For peer review only - http://bmjopen.bmj.com/site/about/guidelines.xhtml

Findings

Six concepts relating to caregiver experience were identified across both quantitative and qualitative papers, (1) mental health, (2) caregiver role, (3) lifestyle change, (4) support for caregivers, (5) knowledge and (6) relationships. There were four additional concepts that were identified only from the qualitative papers (6) expert by experience, (7) vigilance, (8) time and (9) shared-care (See Figure 2). The concepts are reflected in caregiver quotes in Table 5.

For peer review only

For peer review only - http://bmjopen.bmj.com/site/about/guidelines.xhtml

Figure 2

Conceptual Experiences of Caregivers

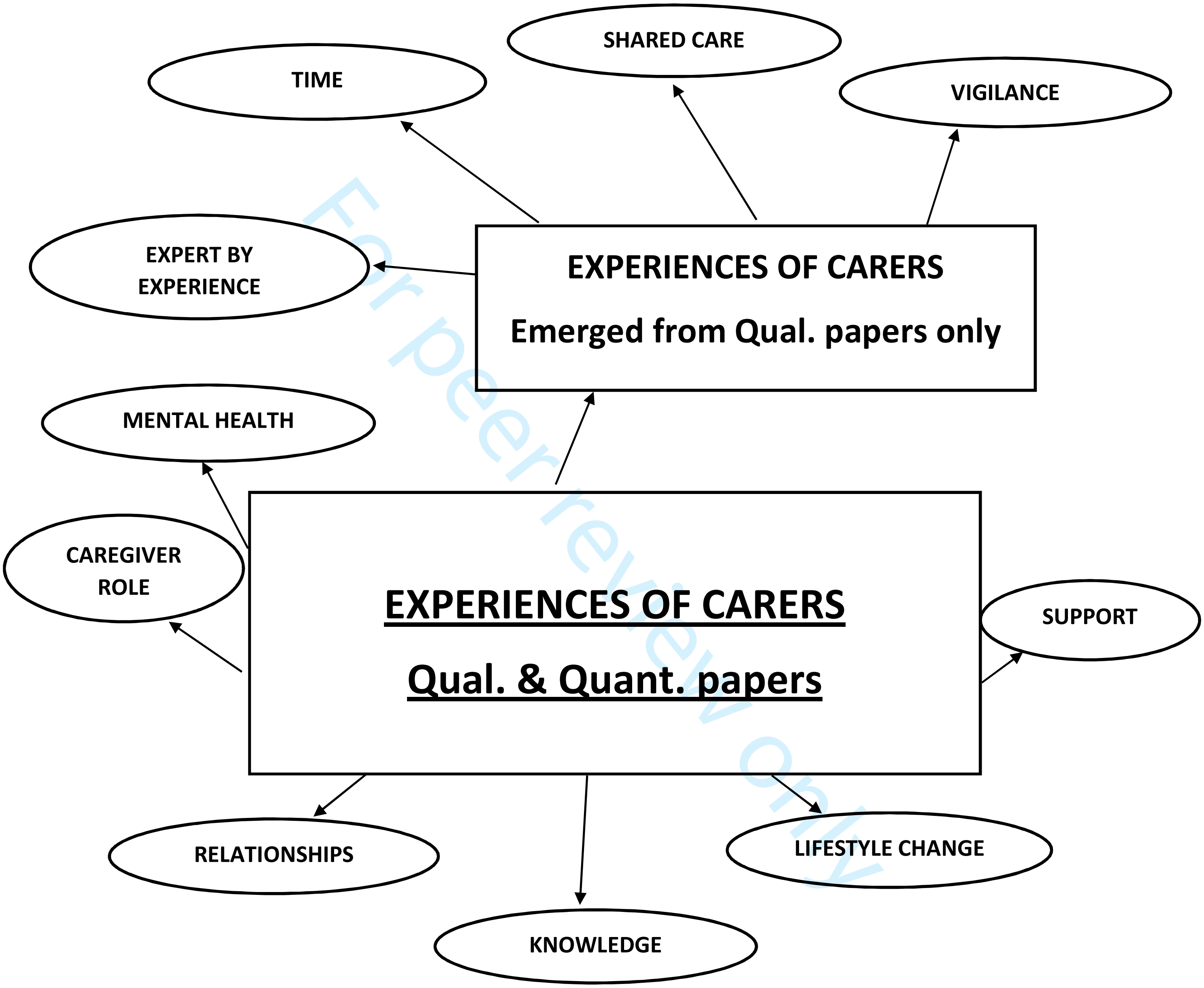

For peer review only - http://bmjopen.bmj.com/site/about/guidelines.xhtml

Table 5

Illustrative Quotes of caregiver experience – by concept

**Mental Health**	*"The mental strain is difficult. I feel so trapped"*<br><br>*""You fall into a huge hole, then the world gets so tiny, it all gets so narrow that it is almost unbearable"*<br><br>*"I feel like sleeping beauty. The hawthorn hedge has closed around me, and I cannot do anything about it."*
**Role**	*"I can sum my role up in three words, I am a cheerleader, drill sergeant, and negotiator"*
**Lifestyle Change**	*"Our life has come down. The two of us used to go out dancing. We loved dancing and then it all stopped."*
**Knowledge**	*"I wish I had had more education on the 'what ifs'. When I was leaving the hospital nobody really said, "OK now this is what's going to happen and this is what you're going to have to do." If there would've been any kind of complications I would've been totally in the dark. I didn't know all the things I needed to know."*
**Relationships**	*"I just love him and I find that every day when I see him, what else could I do to try and make him a wee bit … better? It's very satisfying to know that he appreciates what I do and it's nice to know that you are helping someone."*<br><br>*"'It's like having another child sometimes because you are sort of responsible and I feel he is my responsibility. I feel that he is not anybody else's responsibility…"*
**Support**	*"And then I really felt alone in it all. Because everybody would call and come over and ask, how is John? Hardly anyone asked "how are you doing"?*<br><br>*"Doctors (do) not realize that 1 day your life is jut normal and then this comes and smashes everything to bits, you know- and there are so many questions."*<br><br>*"I would be lost without, our heart failure nurse, and, all the other input we've had from all the other professionals, like the podiatrist and GP. . . .. . . You can do it, but in partnership with everybody else."*

For peer review only - http://bmjopen.bmj.com/site/about/guidelines.xhtml

**Vigilance**	***"Every morning I put my ear to his chest and listen to his heart, that is how we first discovered he was in atrial fibrillation so now I do it every morning before I leave. I monitor him very closely and there are days in which I do not feel comfortable leaving for work so those days I work at home. I call everyday from work and we have our routine, if I am not aware of anything he had planned for the day, I then immediately call my neighbor to check on him"***
**Shared Care**	***"There were days I thought to myself, where are we going from here? But we mastered it together and tried to do things at his pace."***
**Time**	***"At first it was overwhelming. I didn't think I could do it. When they first told me I was like, "I can't do that," you know. And then they explained to me, like, yes you can. It's like getting a new baby. You know, you learn how to take care of them step by step and then it's just part of the routine. And that's really the way it was"***
**Expert by Experience**	***"It's so frustrating when she goes into hospital and the nurses and the doctors say it's her condition, you know. I'm like I'm with her twenty-four hours a day, I know how breathless she is without infection and I know how breathless she is with an infection there's a major difference"***<br><br>***"I see him every day, they are just little subtle changes, they are not showing up in the numbers the doctors are concerned with but I see it"***

1. Mental Health

A total of 26 quantitative[30-56] 20 qualitative studies[57-77] and one mixed method[78] addressed mental health. It was researched across all diagnoses. This encompassed depression and burden. Qualitatively caregivers described an internal and external conflict of emotions, recognising a psychological change within themselves and the care recipient. Maintaining hope and positivity, versus managing worries, fears and anxieties was predominant.[57-76] Quantitatively Pressler's (2013) HF study; identified caregivers had moderately poor health at baseline and at 8 months but they had fewer depressive symptoms over time. Burden arose due to greater responsibilities.[59, 62,67,74,75] Pi-Ming Yeh (2011), noted the quality of relationship and lack of family support significantly predicted greater family carer burden. Näsström (2017) reported, carer burden was concerned with the future and their fears of potential demands. Ability to be resilient was a factor. Those with greater

For peer review only - http://bmjopen.bmj.com/site/about/guidelines.xhtml

resiliency appeared to adjust and cope better with the illness trajectory.[58, 59, 69, 70] Carers highlighted the mental adjustment after an acute event.[70] Living through an acute event was long lasting and some described post traumatic symptoms.[73]

2. Caregiver Role

Qualitative descriptions of the carer role describe it as complex and requiring much co-ordination. [74, 76, 77, 78] Caregivers describe significant role change such as increasing domestic tasks. [57, 60, 63, 65,69 -72, 75] Role loss is prevalent [58, 59, 64] and caregivers need to reframe their identity.[66, 73] Societal expectation regarding the relationship and gender influences caregivers adjusting to their roles.[59, 61, 62, 72, 77] Caring can be positive and rewarding. Caregivers learn about themselves and strengths they have. [59, 68-70, 73, 74, 76] 14 quantitative studies discuss role change specifically focusing on the tasks involved in the role of being a caregiver. [33-35, 43-45, 47, 51, 53, 54, 79, 80, 84, 85] Pressler (2013) was the only high quality quantitative study. It describes the tasks involved; domestic, emotional support, managing dietary needs and transport. Pressler also reports caregivers of persons with greater HF symptoms experience more difficulty with their role.

3. Lifestyle Changes

Fourteen quantitative studies addressed this concept. [47, 31, 32-34, 37, 39, 46, 53, 54, 56, 79, 80, 83, 84] Caregivers described leisure, social and work related problems. [33,34,83] Caring interrupted their schedule thus eliminating things from their routine.[33, 34, 53] In contrast Pressler (2013) reports that caregivers perceptions of how their lives had changed as a result of providing care was neutral and improved from baseline to 4 and 8 months. Qualitatively, caregivers had to become adaptable in their new role.[66,73,86] There was less personal time, greater difficulty engaging with leisure and hobbies either alone or with the care recipient [69,61-64,69,70,75,76] Caregivers described daily "ups & downs" and the need to constantly adjust their routines dependent on the presentation of the care recipient. [57,58,60,65,67,68,71,72,74,76]

4. Support for caregivers

This includes healthcare, family and social support. The weight of perceived external expectations and the necessity of being pro-active in obtaining support from family, friends and healthcare professionals and maintaining a social role was described across all diagnoses.[34,36-40,42-44, 47-51, 55, 85] 15 quantitative studies address this. [35, 39-41, 43, 45-50, 52-54, 56, 79, 78, 85] Pi-Ming Yeh's (2014) study was recognised as being of high quality. It explored the level of care recipient need and ADL dependence and its impact on caregivers. Lack of family support was a significant issue. Additionally, caregivers

For peer review only - http://bmjopen.bmj.com/site/about/guidelines.xhtml

felt abandoned by healthcare teams. At hospital discharge they had to provide care without advice or medical support.[60, 66, 71] Positive interactions with healthcare teams were reported. Namely access to healthcare professionals via telephone or home support.[57, 58, 70, 78] Näsström (2017) examined caregivers' experience of home care services and the support they provided to caregivers. They were satisfied with the service in terms of information received, participation in care and contact.

5. Knowledge

This concept includes caregivers understanding of the diagnosis and their need for knowledge throughout the duration of the illness. [57,61,64,68,76,78,86] Caregivers had to source information themselves. They report what they were told by health professionals was often inadequate. [65, 67, 77] Timing and format of information was significant. Caregivers had experience of receiving information verbally or by leaflets in hospital but describe being left alone to provide care in the long term [59, 62,63,72,75] Caregivers had difficulty understanding how to navigate the care system. [66, 73] They had to make decisions without full knowledge of the consequences of their decision making, especially at times of acute exacerbations.[59] The quantitative piece of Näsström's (2017) mixed methods study was appraised as high quality. Correlating with qualitative studies it reported receiving sufficient information was central to managing HF and was associated with better perceived health of caregivers.

6. Relationships

This concept pertains to the caregiver's relationship with the person they are caring for. 23 quantitative studies across all diagnoses explore this concept. [30-32, 364-38,40-46, 47,49-51,55, 78, 79,81-853] HF studies reported caring for individuals with more symptoms resulted in poorer perceived experiences.[47,81] Caregivers with higher relationship quality experienced less burden and more benefit from the relationship. Qualitative papers report the relationship prior to diagnosis influenced the current relationship. Perspective of the relationship was either a sense of duty [59,73,74,77,78] or feeling this was a valuable second chance.[60,68,75,76] Caregivers reported difficulty communicating about the condition leading to isolation, stress and conflict between caregiver and care recipient.[65,67] The relationship requires negotiating between caregiver and care recipient.[63,86] Repeatedly caregivers prioritised the care recipient over their own needs. [58, 66,70,75,77]

7. Expert by Experience

Caregivers learnt new skills in their caregiving role. They became "experts by experience" discovering through "doing" and observing health professionals.[60,62,76] Essentially developing "proto-

For peer review only - http://bmjopen.bmj.com/site/about/guidelines.xhtml

professional skills"; making decisions on medication administration, [59,73,78,86] judging the care recipients level of functioning each day [72] and decision making in times of exacerbations.[64] Caregivers developed expertise in observing the nuances of change in the care recipient often not perceived by healthcare teams or other family members such as; skin colour or irritability.[66,68,74]

8. Vigilance

Vigilance was recurring in the caregivers' narrative across all diagnoses. Caregivers were always on the alert observing the care recipient. [60,61,64,66,67,70-72,74,77] They lay awake at night listening for their partners' breath.[63,65,68,86] This impacted on caregivers' health creating constant fatigue, worry and stress.[59,73] Caregivers recognised that the need for vigilance came from themselves and their insecurities. [58, 69, 76]

9. Time

Time explores how caregivers adjusted to living with the illness. Caregivers had to adapt to a new life, referring to "then", how life used to be and "now" their current way of life.[63, 64,68,69,76] The duration of caregiving and severity of the illness influenced carer's ability to adjust.[60, 67, 69] Caregivers live day by day [77] and viewed the future, with either hope or uncertainty about what lay ahead. [59, 64,66,72,75]

10. Shared Care

Shared care is how caregiver and care recipient work together to manage the illness. Taking joint responsibility to administer medication [62, 74] and attending appointments together.[67] The presence of illness was described as a process they adjusted to together. [69, 73] Caregivers referred to themselves and the care recipient as "we", when discussing how they deal with the illness.[57,65,68] At times this mutual perspective between carer and care recipient served to isolate them from the world, they described the illness as *"taking a life of its own….it's like this third person"*, (Hynes, 2012, p. 1071)

DISCUSSION

This mixed methods systematic review demonstrates the similarities and differences in caregiver experiences across three common cardio-respiratory conditions. It also highlighted the differences in experiences obtained from qualitative and quantitative research. Commonly occurring experiences for caregivers included the exacerbation of their physical and mental health due to the role. Caregivers did not attend to their own needs and the trajectory of their lives changed with disruption to daily routine across all conditions. Caregivers had predominantly negative experiences

For peer review only - http://bmjopen.bmj.com/site/about/guidelines.xhtml

of support. Family support when available was valued. All caregivers described not knowing who to contact for advice or support when providing care at home. Those who reported on positive experiences of support gave examples where they had a nurse specialist to contact or where there was a home care team in place. All caregivers emphasised their need to be vigilant. This was either overt, putting one's head on the chest of the recipient to check breathing or covert; observing them throughout the day. All caregivers worked together with the recipient in illness management, this included counting medication together and using the term "we" and "it" when describing how they worked together against the illness. Time stood still for caregivers as they learnt to live with the illnesses. They expressed their fears for the future and what the passing of time would mean for them and the person for whom they were providing care. This concept of time was experienced by all caregivers. Each of the caregivers described their expertise by experience; for HF and COPD caregivers this was recognising subtle cues that may indicate a change in health such as skin colour or mood. CAD caregivers' expertise was more practical observing nurses administering wound dressings and developing expertise as a result of doing this task. There were differences in caregiver experience for each of the diagnoses and these are discussed below.

Heart Failure

HF caregivers experienced an "ebb and flow" in caring, a constant underlying worry, fear and anxiety which at times of change or illness heightened. HF caregivers experienced long lasting and sustained change in their lives. Additionally, they viewed obtaining knowledge as necessary throughout all stages of the illness. Sourcing information and communication with health professionals was often difficult. In spousal relationships they predominantly viewed the care recipient as another child or as a "duty."

Chronic Obstructive Pulmonary Disease

COPD caregivers experienced a prolonged impact on their mental health similar to HF caregivers. Severity of illness was influential on their experiences of burden. Role change was long lasting and profound for many and they expressed concerns with perceived lack of knowledge. In times of exacerbations COPD caregivers experienced anxiety and fear of their loved one dying due to not being able to breathe. COPD caregivers highlighted the loss of social roles as a result of trying to maintain their loved ones dignity. The coughing and spitting associated with COPD often left the care recipient embarrassed. The caregiver tried to avoid situations where this would happen. In spousal relationships changes in the relationship dynamic resulted in caregivers describing losing the intimate love they had for their partner.

For peer review only - http://bmjopen.bmj.com/site/about/guidelines.xhtml

Coronary Artery Disease

Caregivers of CAD patients experienced intense role change on discharge from hospital and in the acute phase of illness. They initially engaged with a high volume of tasks which reduced over time. CAD caregivers experienced post-traumatic type symptoms if they witnessed the recipient experience an acute event. Caregivers described being unable to talk about this and reliving the event in their heads. This anxiety did ease over time for many. Caregivers felt unprepared at hospital discharge and highlighted not realising how much their routine would change in order to provide care. Regarding the relationship dynamic between caregiver and recipient; they frequently reported viewing the experience as a second chance and had a renewed sense of love and appreciation for the relationship.

The findings of this systematic review correlate with Kang et al.'s (2011) systematic review of qualitative studies regarding caregiver experiences in Heart Failure. They identify that caregiving resulted in a multitude of changes in caregiver's lives regardless of age, gender and ethnicity. Doherty et al. (2016) emphasise the importance of addressing both patient and caregiver needs in order to maintain wellbeing for both. Additionally they highlight the importance of psychosocial support for caregivers which has been demonstrated in this review by the prevalence of mental health needs in caregivers. This also concurs with findings by AasbØ et al. (2016) reporting caregivers feeling the need to be in "emotional control" and Wingham et al. (2017) describing the "enduring anguish" experienced by caregivers. These descriptions emphasise the profound mental health impact of caregiving. Caregivers in Giancomini et al.'s (2012) qualitative review of living and dying with COPD reported increasing isolation in addition to having their own health issues. They described feeling pressured to balance their variety of caregiving roles; all similar experiences to the caregivers in this synthesis across the three diagnoses. Regarding support, Nakken et al. (2015) emphasise the need to include caregivers of COPD patients in clinical appointments to ensure they are not isolated in providing care and to allow for knowledge exchange. Similarly, Buck et al. (2015) recognise that caregivers are valuable providers of care and the inclusion of caregivers is necessary when developing collaborative models of care. This corresponds with the findings of this review.

Strengths and limitations

This mixed methods systematic review demonstrates the complexity of what it means to be a caregiver and should inform clinical care and the development of interventions. A mixed methods review can be contentious[92] as it requires synthesis of differing paradigms of quantitative and

For peer review only - http://bmjopen.bmj.com/site/about/guidelines.xhtml

quantitative research. In this review it required transformation of quantitative data into qualitative data.[15] We aimed to present a convergence of caregiver experiences by conducting a mixed methods synthesis. However, it demonstrated a divergence between the two paradigms in four of the emerging concepts. This highlights the challenge of synthesising multiple methods. Due to the importance and prevalence in the qualitative studies of the four concepts it was agreed that they were essential to include in this review. They contribute valuable knowledge to understanding caregiver experience. The four qualitative concepts may be difficult to assess with a set of outcome measures, however it is worth exploring how to capture them quantitatively in order to inform healthcare intervention. We believe this mixed methods synthesis is, to our knowledge the first to combine caregiver experiences in HF, COPD and CAD. It establishes a comprehensive assessment of the knowledge base of caregiver experiences in common cardiorespiratory conditions. It examines the differences and similarities in caregiver experiences across these diagnoses.

This review has three potential limitations; first, study inclusion was limited to English language studies from high income countries. We excluded caregivers of nursing home residents. Our findings are therefore likely to have limited applicability to other settings. Second, qualitative synthesis is an interpretation of other researcher's interpretations. However, we sought to minimise individual interpretative bias by seeking confirmation of the results by a second researcher. Third, the vast majority of included qualitative and quantitative studies were cross-sectional in design and therefore consider caregiver experience only at a single point of time.

Implications for practice and future research

In order to fully address the needs of caregivers identified in this review, future research should focus on the involvement of caregivers in the design and delivery of interventions for patients with cardiopulmonary disease. In addition to targeting patients, interventions need to consider targeting caregiver. Considering the experiences of CAD caregivers best practice guidelines for engaging with this population must be developed. Additionally, further research should also explore the impact of caring in COPD and CAD regarding caregivers' mental health. Consideration needs to be given as to whether quantitative research tools to explore caregiver expertise, view of the future, experience of shared care and vigilance can be developed in order to capture these important caregiver experiences. Additionally, longitudinal studies with repeated assessment need to be conducted in order to assess how stable are caregiver experiences or whether they are liable to much change over time. In this review only four out of thirty-two quantitative studies examined caregiver's experiences longitudinally. Qualitative research clearly demonstrates the caregiving experience is constantly evolving and this needs to be reflected in quantitative research. The quality of quantitative studies in

For peer review only - http://bmjopen.bmj.com/site/about/guidelines.xhtml

COPD and CAD were medium or low quality. This indicates the need for more empirically robust studies examining the experiences of caregivers in particular their mental health needs.

CONCLUSIONS

This mixed methods systematic review provides a holistic synthesis of experiences of unpaid caregivers of people with HF, COPD and CAD. It demonstrates that there are a number of implications when an individual becomes a caregiver for those with chronic cardiopulmonary disease. This includes re-framing one's identity and a change of life course. Caregivers learn and develop a multitude of skills and become experts in their new role. This expertise is invaluable and needs to be acknowledged in healthcare interventions for these conditions. Caring can be a positive experience if caregivers have access to social support, are well informed and are included as part of the healthcare team. Understanding the experiences of caregivers for people with these conditions allows healthcare professionals and policy makers to reflect on our approach. It is essential for health services to involve caregivers in the design and delivery of interventions if caregivers are to successfully manage their role and facilitate those with long term chronic illness to manage their conditions and live well in their own homes. The quality of evidence was limited by assessment of caregiver experience at single point of time. There is a need for future studies that employ longitudinal designs and examine the change in caregiver experience over time.

Contributors: MN, JW, RST: conceived and designed the review and quality appraised the included studies. MN wrote the initial draft of the manuscript. JW AND RST provided feedback and edits to manuscript drafts.

Funding

This study was supported by a University of Exeter Postgraduate Studentship Grant

Competing Interests

None declared

Data Sharing

Any requests for additional information about the study can be accessed via contacting the primary author.

Acknowledgements

We would like to thank the PenCLARHC Evidence Synthesis Team at University of Exeter Medical School**.** Katy Oaks and Catriona Organ, Librarians at Royal Cornwall Hospital Library, Knowledge Spa, Truro. Dr. Ruth Garside, Senior Lecturer in Evidence Synthesis, European Centre for Environment and Human Health for their assistance throughout the process of this review.

For peer review only - http://bmjopen.bmj.com/site/about/guidelines.xhtml

References:

1. International Alliance of Carer Organizations. *Global carer facts*. Available from: http://www.internationalcarers.org/carer-facts/global-carer-stats/(accessed 1st October 2017).
2. White, C. 2011 Census Analysis: Unpaid care in England and Wales, 2011 and comparison with 2001. *Office of National Statistics.* 2013. Available from: https://www.ons.gov.uk/peoplepopulationandcommunity/healthandsocialcare/healthcaresystem/articles/2011censusanalysisunpaidcareinenglandandwales2011andcomparisonwith2001/2013-02-15 (accessed 10th October 2017).
3. Carers UK. *State of Caring.* Available from: www.carersuk.org/ (accessed 25th July 2017).
4. Institute of Public Care. *A Carer's Life: Implications and Considerations for Commissioning.* 2017. Available from: http://ipcbrookes.ac.uk (accessed 30th September 2017).
5. Seltzer, MM, Wailing Li, L. The dynamics of caregiving: transitions during a three-year prospective study. *The Gerontologist.* 2000;40(2):165–178. Available from: https://doi.org/10.1093/geront/40.2.165.
6. The Care Act. Chapter 23. *The Care Act UK: The Stationery Office.* 2014. Available from: http://www.legislation.gov.uk/ukpga/2014/23/contents.enacted/data.htm (accessed 11th October 2016).
7. National Institute for Health and Clinical Excellence. *Chronic heart failure in adults: management.* 2010. NICE guideline (CG108).
8. National Institute for Health and Clinical Excellence. *Chronic obstructive pulmonary disease in over 16s: diagnosis and management.* 2010. NICE guideline (CG101).
9. Wilkins, E, Wilson, L, Wickramasinghe K, Bhatnagar, P, Leal, J, Luengo-Fernandez, R, Townsend, N. European Cardiovascular Disease Statistics 2017. *European Heart Network*, Brussels. Available from: http://www.ehnheart.org/images/CVD-statistics-report-August-2017.pdf (accessed 25th August 2017).
10. Center for Disease Control and Prevention. *Heart Disease Facts and Statistics*. Available from: www.cdc.gov (accessed 9th November 2016).
11. Raherison, C, Girodet, PO. Epidemiology of COPD. *European Respiratory Review*. 2009;18: 213-221. Available from: doi:10.1183/09059180.00003609.
12. Falk, JA, Kadiev, S, Criner, GJ, Scharf, SM, Minai, OA, Diaz, P. Cardiac disease in chronic obstructive pulmonary disease. *Proceedings of the American Thoracic Society*. 2008;5(4): 543-548. Available from: doi:10.1513/pats.200708-142ET.
13. Müllerova, H, Mapel, DW. Cardiovascular comorbidity in COPD systematic literature review. *CHEST.* 2013;144(4):1163–1178. Available from: doi:10.1378/chest.12-2847.
14. Kang, X, Li, Z, Nolan, T. Informal caregivers' experiences of caring for patients with chronic heart failure systematic review and metasynthesis of qualitative studies. *Journal of Cardiovascular Nursing.* 2011;26(5):386-394. Available from: doi: 10.1097/JCN.0b013e3182076a69.
15. Doherty, L, Fitzsimons, D, McIlfatrick, S. Carers' needs in advanced heart failure: a systematic narrative review. European *Journal* of Cardiovascular Nursing. 2016;15(4):203-212. Available from: doi: 10.1177/1474515115585237.

For peer review only - http://bmjopen.bmj.com/site/about/guidelines.xhtml

16. Ängerund, KH, Boman K, Brännström M. Areas for quality improvements in heart failure care: quality of care from the family members' perspective. *Scandinavian Journal of Caring Sciences.* 2017: 1471-6712. Available from: doi:10.1111/scs.12468.
17. Giacomini, M, DeJean, D, Simeonov, D, Smith, A. Experiences of living and dying with COPD: a systematic review and synthesis of the qualitative empirical literature. *Ontario* Health *Technology* Assessment Series. 2012;12(13):1-47.
18. Mansfield, E, Bryant, J, Regan, T, Waller, A, Boyes, A, Sanson-Fisher, R. Burden and unmet needs of caregivers of chronic obstructive pulmonary disease patients: a systematic review of the volume and focus of research output. *Journal of Chronic Obstructive Pulmonary Disease. 2016;* 13(5):662-667. Available from: doi:10.3109/15412555.2016.1151488.
19. Moher, D, Liberati, A, Tetzlaff, J, Altman, DG, The PRISMA Group. Preferred reporting items for systematic reviews and meta-analyses: The PRISMA statement. *PLoS Med.* 2009;6(7). Available from: doi:10.1371/journal.pmed.1000097.
20. Johnson, RB, Onwuegbuzie, AJ, Turner, LA. Toward a definition of mixed methods research. *Journal of Mixed Methods Research,* 2007;1:112–133. Available from: doi:10.1177/1558689806298224.
21. World Bank Data. Available from: http://www.worldbank.org/ (accessed 10th June 2017).
22. Critical Appraisal Skills Progamme. *Qualitative research checklist.* Available from: http://docs.wixstatic.com/ugd/dded87_25658615020e427da194a325e7773d42.pdf (accessed 10th December 2016).
23. Hannes K. Critical appraisal of qualitative research. In: Noyes J, Booth A, Hannes K, Harden A, Harris J, Lewin S, Lockwood C. (eds.) *Supplementary guidance for inclusion of qualitative research in cochrane systematic reviews of interventions*. Cochrane Collaboration Qualitative Methods Group. Available from: *http://methods.cochrane.org/qi/supplemental-handbook-guidance* (accessed 12th January 2017).
24. Dixon-Woods, M, Fitzpatrick, R, Roberts, K. Including qualitative research in systematic reviews: opportunities and problems. *Journal of Evaluation in Clinical Practice*. 2001;7(2):125-133. Available from: doi:10.1046/j.1365-2753.2001.00257.x.
25. Sandelowski, M, Voils, CI, Barroso, J. Defining and Designing Mixed Research Synthesis Studies. *Research in the Schools : A Nationally Refereed Journal Sponsored by the Mid-South Educational Research Association and the University of Alabama.* 2006;13(1):1-15.
26. Pluye, P, Hong, QN. *Annual Review of Public Health*. 2014. Available from: www.annualreviews.org (accessed September 27th 2016).
27. Hong, QN, Pluye, P, Bujold, M, Wassef, M. Convergent and sequential synthesis designs: implications for conducting and reporting systematic reviews of qualitative and quantitative evidence. *Systematic Reviews*. 2017;6(61). Available from: doi:10.1186/s13643-017-0454-2.
28. Tashakkori, A, Teddlie, C. Mixed Methodology: Combining Qualitative and Quantitative Approaches. *Applied Social Research Methods Series*. 1998;46. Sage Publications, Thousand Oaks, CA.
29. Frantzen, KK, Fetters, MD (2015). Meta-integration for synthesizing data in a systematic mixed studies review: insights from research on autism spectrum disorder. *Qual Quant.* 2015;50:2251–2277. Available from: doi:10.1007/s11135-015-0261-6.
30. Ågren, S, Evangelista, L, Strömberg, A. Do partners of patients with chronic heart failure experience caregiver burden? *European Journal of Cardiovascular Nursing*, 2010;9(4):254–262. Available from: doi:10.1016/j.ejcnurse.2010.03.001.
31. Al-Rawashdeh, SY, Lennie, TA, Chung, ML. The association of sleep disturbances with quality of life in heart failure patient–caregiver dyads. *Western Journal of Nursing Research*, 2017;39(4):492–506. Available from: doi: 10.1177/0193945916672647.

For peer review only - http://bmjopen.bmj.com/site/about/guidelines.xhtml

32. Badr, H, Federman, AD, Wolf, M, Revenson, TA Wisnivesky, JP. Depression in individuals with chronic obstructive pulmonary disease and their informal caregivers. *Aging & Mental Health. 2017;*21(9): 975-982. Available from: doi:10.1080/13607863.2016.1186153.
33. Bakas, T, Pressler, SJ, Johnson, E, Nauser, J, Shaneyfelt, T. Family caregiving in heart failure. *Nursing Research.* 2006;55(3):180-188.
34. Chung, ML, Lennie, TA, Mudd-Martin, G, Dunbar, SB, Pressler, SJ, Moser, DK. Depressive symptoms in patients with heart failure negatively affect family caregiver outcomes and quality of life. *European Journal of Cardiovascular Nursing. 2016;*15 (1):30-38. Available from: doi:10.1177/1474515114535329.
35. Cossette, S, Levesque, L. Caregiving tasks as predictors of mental health of wife caregivers of men with chronic obstructive pulmonary disease. *Research in Nursing & Health*, 1993;16:251-263.
36. Evangelista, l, Dracup, K, Doering, L, Westalke, C, Fonarow, GC Hamilton, M. Emotional well-being of heart failure patients and their caregivers. *Journal of Cardiac Failure*. 2002;8(5): p.300-305. Available from: doi:10.1054/jcaf.2002.128005.
37. Figueiredo, D., Gabriel, R., Jácome, C. & Marques, A. Caring for people with early and advanced chronic obstructive pulmonary disease: how do family carers cope? *Journal of Clinical Nursing.* 2013;23:211–220. Available from: doi: 10.1111/jocn.12363.
38. Figueiredo, D, Gabriel, R, Jácome, C, Cruz, J, Marques, A. Caring for relatives with chronic obstructive pulmonary disease: how does the disease severity impact on family carers? *Aging & Mental Health.* 2014;18(3):385-393. Available from: doi:10.1080/13607863.2013.837146.
39. Grigorovich, A, Lee, A, Ross, H, Woodend, K, Forde, S, Cameron, JI. A longitudinal view of factors that influence the emotional well-being of family caregivers to individuals with heart failure. *Aging & Mental Health.* 2016;21(8):844-850. Available from: doi:10.1080/13607863.2016.1168361.
40. Halm, MA, Jacobson-Treat, D, Lindquist, R. Correlates of caregiver burden after coronary artery bypass surgery. *Nursing Research*. 2006;55(6):426-436.
41. Halm, MA, Bakas, TB. Factors associated with caregiver depressive symptoms, outcomes, and perceived physical health after coronary artery bypass surgery. *Journal of Cardiovascular Nursing*. 2007;22(6):508-515. Available from: doi:10.1097/01.JCN.0000297388.21626.6c.
42. Hooley, PJD, Butler, G, Howlett, JG. The relationship of quality of life, depression, and caregiver burden in outpatients with congestive heart failure. *Congestive Heart Failure.* 2005;11(6):303-310. Available from: doi:10.1111/j.1527-5299.2005.03620.x.
43. Hwang, B, Fleischmann, KE, Howie-Esquivel, J, Stotts, NA, Dracup, K. Caregiving for patients with heart failure: impact on patients' families. *American Journal of Critical Care.* 2011;20(6):431-441. Available from: doi: 10.4037/ajcc2011472.
44. Karmilovich, SE. Burden and stress associated with spousal caregiving for individuals with heart failure. *Progress in Cardiovascular Nursing.* 1994;9(1):33-38.
45. Loftus, L. The influence of illness and psychological factors on patient and caregiver outcomes in heart failure. PhD [dissertation]. Liverpool: University of Liverpool; 2004.
46. Nakken, N, Janssen, DJA, van Vilet, M, de Vries, GJ, Clappers-Gielen, GAL, Michels, AJ, Spruit, MA. Gender differences in partners of patients with COPD and their perceptions about the patients. *International Journal of COPD*. 2017;12: 95–104. Available from: doi:https://doi.org/10.2147/COPD.S118871.
47. Pressler, SJ, Gradus-Pizlo, I, Chubinski, SD, Smith, G, Wheeler, S, Sloan, R, Jung, M. Family caregivers of patients with heart failure: a longitudinal study. *Journal of Cardiovascular Nursing.* 2013;28(5):417-428. Available from: doi:10.1097/JCN.0b013e3182563877.

For peer review only - http://bmjopen.bmj.com/site/about/guidelines.xhtml

48. Yeh, PM, Bull M. Use of the resiliency model of family stress, adjustment and adaptation in the analysis of family caregiver reaction among families of older people with congestive heart failure. *International Journal of Older People Nursing.* 2012;7:117–126. Available from: doi:10.1111/j.1748-3743.2011.00275.
49. Saunders, MM. Factors associated with caregiver burden in heart failure family caregivers. *Western journal of nursing research.* 2008;30(8):943-959. Available from: doi: 10.1177/0193945908319990.
50. Saunders, MM. Indicators of health-related quality of life in heart failure family caregivers. *Journal of Community Health Nursing.* 2009;26:173–182. Available from: doi: 10.1080/07370010903259196.
51. Saunders, MM. Working and caregiving a comparison of employed and unemployed caregivers of older heart failure patients. *Holistic Nursing Practice*. 2010;24(1):16-22. Available from: doi:10.1097/HNP.0b013e3181c8e469.
52. Schwarz, KA, Dunphy, G. An examination of perceived stress in family caregivers of older adults with heart failure. *Experimental Aging Research*. 2003;29(2):221-235. Available from doi:10.1080/03610730303717.
53. Scott, LD. Caregiving and care receiving among a technologically dependent heart failure population. *Advances in Nursing Science.* 2000;23(2):82-97
54. Takata, S, Washio, M, Moriwaki, A, Tsuda, T, Nakayama, H, Iwanaga, T, Inoue, H. Burden among caregivers of patients with chronic obstructive pulmonary disease with long-term oxygen therapy. *International Medical Journal.* 2008;15(1):53-57.
55. Vellone, E, D'Agostino, F, Buck, HG, Fida, R, Spatola, CF, Petruzzo, A, Riegel, B. The key role of caregiver confidence in the caregiver's contribution to self-care in adults with heart failure. *European Journal of Cardiovascular Nursing*. 2015;14(5):372–381. Available from: doi:10.1177/1474515114547649.
56. Woolfe, P, McMillan, M, Conway, J. The needs of caregivers of people with COPD: a study. *Australian Journal of Primary Health.* 2007;13(1):28-35. Available from: doi: 10.2147/COPD.S76091.
57. Andersen, KL, Korneliussen, K, Fagermoen, MS. Family caregivers to a patient with chronic heart failure living at home: «co-workers» in a blurred health care system. *Journal of Cardiovascular Nursing*. 2013;20(3):162-9.
58. Baker, K, Flattery, M, Salyer, J, Haugh, KH, Maltby, M. Caregiving for patients requiring left ventricular assistance device support. *Heart & Lung.* 2010;39(3):196-200. Available from: doi:10.1016/j.hrtlng.2009.07.007.
59. Bove, DG, Zakrisson, AB, Midtgaard, J, Lomborg, K, Overgaard, D. Undefined and unpredictable responsibility: a focus group study of the experiences of informal caregiver spouses of patients with severe COPD. *Journal of Clinical Nursing*. 2016;25:483–493. Available from: doi:10.1111/jocn.13076 483.
60. Burke, RE, Jones, J, Ho, MJ, Bekelman, D. Caregivers' perceived roles in caring for patients with heart failure: what do clinicians need to know? *Journal of Cardiac Failure,* 2014;20 (10):731-738. Available from: doi.org/10.1016/j.cardfail.2014.07.011.
61. Figueiredo, D, Jácome, C, Gabriel, R, Marques, A. Family care in chronic obstructive pulmonary disease: what happens when the carer is a man? *Scandinavian Journal of Caring Sciences.* 2016;30:721-730. Available from: doi: 10.1111/scs.12298.
62. Halm, MA. Specific needs, concerns, strategies and advice of caregivers after coronary artery bypass surgery. *Heart & Lung*. 2016;45:416-422. Available from: doi.org/10.1016/j.hrtlng.2016.07.001.

For peer review only - http://bmjopen.bmj.com/site/about/guidelines.xhtml

63. Halm, MA. Age and gender influences on the needs, concerns and strategies of CABG caregivers. *Heart & Lung*. 2017;46:159-165. Available from: doi.org/10.1016/j.hrtlng.2017.01.012.
64. Hynes, G, Stokes, A, McCarron, M. Informal care-giving in advanced chronic obstructive pulmonary disease: lay knowledge and experience. *Journal of Clinical Nursing*. 2012;21:1068-1077. Available from: doi: 10.1111/j.1365-2702.2011.03944.
65. Imes, C, Dougherty, CM, Pyper, G, Sullivan, MD. Care of Patients with Heart Failure Descriptive study of partners' experiences of living with severe heart failure. *Heart & Lung*. 2011;40:208-216. Available from: doi:10.1016/j.hrtlng.2010.12.007.
66. Kitko, LA. The work of spousal caregiving in heart failure. PhD [dissertation]. State College, PA: Pennsylvania State University;2010.
67. Liljeroos, M, Ågren, S, Jaarsma, T, Strömberg, A. Perceived caring needs in patient-partner dyads affected by heart failure: a qualitative study. *Journal of Clinical Nursing.* 2014;23:2928–2938. Available from: doi: 10.1111/jocn.12588.
68. Luttik, MLL, Jaarsma, T. Living with heart failure: partner perspectives. *Journal of Cardiovascular Nursing.* 2007;22(2):131-137. Available from: doi:10.1097/00005082-200703000-00010.
69. Marcucilli, L, Casida, J. From insiders' perspectives: adjusting to caregiving for patients with left ventricular assist devices. *Progress in Transplantation.* 2011;21(2):137-43. Available from: doi:10.7182/prtr.21.2.dq53164u1464u683.
70. Marcucilli, L, Casida, J, Bakas, T, Pagani, FD. Family caregivers' inside perspectives: caring for an adult with a left ventricular assist device as a destination therapy. *Progress in Transplantation*. 2014;24(4):332-340. Available from: doi:10.7182/pit2014684.
71. Pattenden, JF, Roberts, H Lewin, HJP. Living with heart failure; patient and carer perspectives. *European Journal of Cardiovascular Nursing.* 2007;6:273-279. Available from: doi:10.1016/j.ejcnurse.2007.01.097.
72. Rolley, J, Smith, J, DiGiacomo, M, Salamonson, Y, Davidson, P. The caregiving role following percutaneous coronary intervention. *Journal of Clinical Nursing*. 2010;20:227–235. Available from: doi: 10.1111/j.1365-2702.2009.03104.
73. Spence, A, Hasson, F, Waldron, M, Kernohan, G, McLaughlin, D, Cochrane, B, Watson, B. Active carers: living with chronic obstructive pulmonary disease. *International Journal of Palliative Nursing*. 2008;14(8):368-372. Available from: doi:10.12968/ijpn.2008.14.8.30771.
74. Ström, A, Andersen, KL, Korneliussen, K, Fagermoen, MS. Being "on the alert" and "a forced volunteer": a qualitative study of the invisible care provided by the next of kin of patients with chronic heart failure. *Journal of Multidisciplinary Healthcare.* 2015;8:271–277. Available from: doi.org/10.2147/JMDH.S82239.
75. Wallin, E, Larsson, IM, Rubertsson, S, Kristoferzon, ML. Relatives' experiences of everyday life six months after hypothermia treatment of a significant other's cardiac arrest. *Journal of Clinical Nursing.* 2013;22:1639–1646. Available from: doi: 10.1111/jocn.12112.
76. Wingham, J, Frost, J, Britten, N, Jolly, K, Greaves, C, Abraham, C, Dalal, H. Needs of caregivers in heart failure management: a qualitative study. *Chronic Illness*. 2015;11(4):304-319. Available from: doi:10.1177/1742395315574765.
77. Lindqvist, G, Albin, B, Heikkila, K Hjelm, K. Conceptions of daily life in women living with a man suffering from chronic obstructive pulmonary disease. *Primary Health Care Research & Development.* 2012;14:40–51. Available from: doi.org/10.1017/S146342361200031X.
78. Näsström, L, Luttik, ML, Idvall, E, Strömberg, A. Exploring partners' perspectives on participation in heart failure home care: a mixed-method design. *Journal of Advanced Nursing.* 2017;73(5):1208–1219. Available from: doi: 10.1111/jan.13216.

For peer review only - http://bmjopen.bmj.com/site/about/guidelines.xhtml

79. Kneeshaw, MF, Considine, RM, Jennings, J. Mutuality and preparedness of family caregivers for elderly women after bypass surgery. *Applied Nursing Research*. 1999;12(3):128-135.
80. Hess, AM. *The health literacy, self-care, and medication hassles of patients with heart failure and their caregivers.* Masters [dissertation]. West Lafayette, IN: Purdue University;2009.
81. Lum, HD, Lo, D, Hooker, S, Bekelman, D.B. Caregiving in heart failure: relationship quality is associated with caregiver benefit finding and caregiver burden. *Heart & Lung*. 2014;43:306-310. Available from: doi.org/10.1016/j.hrtlng.2014.05.002.
82. Luttik, ML, Jaarsma, T, Lesman, I, Sanderman, R, Hagedoorn, M. Quality of life in partners of people with congestive heart failure: gender and involvement in care. *Journal of Advanced Nursing.* 2009;65(7):1442–1451. Available from: doi: 10.1111/j.1365-2648.2009.05004.
83. Miravitlles, M, María, L, Oliva-Moreno, J, Hidalgo-Vega, Á. Caregivers' burden in patients with COPD. *International Journal of COPD.* 2015;10(1):347—356. Available from: doi.org/10.2147/COPD.S76091.
84. Park, EO, Yates, BC, Schumacher, KL, Meza, J, Kosloski, K, Pullen, C. Caregiving demand and difficulty in older adult spousal caregivers after coronary artery bypass surgery. *Geriatric Nursing*. 2013;34:383-387. Available from: doi.org/10.1016/j.gerinurse.2013.05.007.
85. Riegner, J. *The relationship of role strain, perceived social support, and humor to quality of life among couples experiencing the life-limiting illness of chronic obstructive pulmonary disease.* PhD [dissertation]. Tallahassee, Florida: Florida State University;1996.
86. Clark, AM, Reid, ME, Morrisson, CE, Capewell, S, Murdoch, DL, McMurray, JJ. The complex nature of informal care in home-based heart failure management. *Journal of Advanced Nursing*. 2008;61(4):373–383. Available from: doi:10.1111/j.1365-2648.2007.04527.x.
87. AasbØ, G, Nyheim Solbrække, K, Kristvik, E, Werner, A. Between disruption and continuity: challenges in maintaining the "biographical we" when caring for a partner with a severe chronic illness. *Sociology of health and illness*. 2016;38(5):782-796. Available from: doi:10.1111/1467-9566.12396.
88. Wingham, J, Frost, J Britten, N. Behind the smile: qualitative study of caregivers' anguish and management responses while caring for someone living with heart failure. *BMJ Open*. 2017;7:e014126. Available from: doi:10.1136/bmjopen-2016-014126.
89. Buck, HG, Harkness, K, Wion, R, Carroll, SL, Cosman, T, Kaasalainen, S, Arthur, HM. Caregiver's contributions to heart failure self-care: a systematic review. *European Journal of Cardiovascular Nursing*. 2015;14(1):79-89. Available from: doi:10.1177/1474515113518434.
90. Nakken, N, Janssen, DJA, van den Bogaart, EHA, Wouters, EFM, Franssen, FME, Vercoulen, JH, Spruit, MA. *European Respiratory Review.* 2015;24(137):498-504. Available from: doi: 10.1183/16000617.00010114.
91. Dixon-Woods, M, Agarwal, S, Jones, D, Young, B, Sutton, A. Synthesising qualitative and quantitative evidence: a review of possible methods. *Journal of Health Services Research*. 2005;10(1):45-53

For peer review only - http://bmjopen.bmj.com/site/about/guidelines.xhtml

For peer review only

For peer review only - http://bmjopen.bmj.com/site/about/guidelines.xhtml

For peer review only

For peer review only - http://bmjopen.bmj.com/site/about/guidelines.xhtml

Supplementary:

Table 1: List of search terms

Medline:

MeSH: Caregivers

“Caregivers”.ti,ab OR “care-giver”.ti,ab OR “carer*.ti,ab” OR “informal car*”.ti,ab

AND

MeSH: Quality of life

“Quality of life” OR “outcome measures” OR “caregiver outcomes” OR “care-giver outcomes” OR “time use” OR “time-use” OR “occupations” OR “occupational engagement” OR “self-efficacy” OR “self efficacy” OR “experience” OR “emotion*” OR “psychological health impact” OR “physical health impact” OR “confidence” OR “self-confidence” OR “satisfaction” OR “dissatisfaction” OR “activities of daily living” OR “resilience” OR “social adj2 (interaction or engagement or support) OR “Social adj2 (participation)” OR “caregiver adj2 (support or health professional or medical team or nurse or patient or relationship* or knowledge)” OR “information exchange” OR “coping strategies” OR “leisure activity” OR “conflict” OR “caregiver responsib*” OR care-giver responsib*” OR “caregiver expectation” OR “care-giver expectation” OR “caregiver role” OR “care-giver role” OR “role adjustment” OR “caregiver or care-giver adj2 (attitude to health)”

AND

MeSH: Heart Failure, Heart Diseases, Myocardial Ischaemia, Coronary Artery Disease, Pulmonary Disease, Chronic Obstructive, Pulmonary Heart Disease, Chronic Obstructive Pulmonary Disease, Acute Coronary Syndrome, Cardiovascular Diseases, Coronary Artery Disease, Coronary Artery Bypass, Coronary Disease, Coronary Aneurysm, Stroke, Myocardial Infarction

“Heart Failure”.ti,ab OR “cardiac failure”.ti,ab OR “myocardial failure”.ti,ab OR “left ventricular failure”.ti,ab OR “right ventricular failure”.ti,ab OR “cardiomyopathy”.ti,ab, OR “systolic failure”.ti,ab OR “diastolic failure”.ti,ab OR “Chronic Obstructive Pulmonary Disease”.ti,ab OR “COPD”.ti,ab OR “chronic obstructive lung disease”.ti,ab OR “pulmonary disease”.ti,ab OR “pulmonary disorder”.ti,ab OR “respiratory disease”.ti,ab OR “respiratory disorder”.ti,ab OR “dyspnea”.ti,ab OR “dyspnoea”.ti,ab OR “Stroke”.ti,ab OR “stroke disability”.ti,ab OR “stroke disease”.ti,ab OR “stroke disorder”.ti,ab OR “CVA”.ti,ab OR “cerebrovascular accident”.ti,ab OR “transient ischaemic attack”.ti,ab OR “transient ischemic attack”.ti,ab OR “haemorrhage”.ti,ab OR “hemorrhage”.ti,ab OR “cerebral haemorrhage”.ti,ab OR “cerebral hemorrhage”.ti,ab OR “aneurysm”.ti,ab OR “atrial fibrillation”.ti,ab OR “Coronary Artery Disease”.ti,ab OR “acute coronary syndrome”.ti,ab OR “atherosclerosis”.ti,ab OR “arteriosclerosis”.ti,ab OR “ischaemic heart disease”,ti.ab OR “ischemic heart disease”.ti,ab OR “myocardial infarction”,ti.ab OR “coronary revascularisation”.ti,ab OR “coronary revascularization”.ti,ab OR “angina”.ti,ab OR “CABG”.ti,ab OR “coronary artery bypass graft”.ti,ab OR “stable angina”.ti,ab OR “angina pectoris”.ti,ab

CINAHL:

MeSH: Caregivers, caregiver burden, caregiver supports, caregiver role strain, risk for caregiver role strain, family caregiver status, caregiving endurance potential, caregiver-patient relationship, caregiver well-being, caregiver support, caregiver stressors, caregiver physical health, caregiver performance, caregiver performance: direct care, caregiver performance indirect care, caregiver lifestyle disruption, caregiver emotional health, caregiver strain index, caregiver role strain.

“Caregiver”.ti,ab OR “care-giver”.ti,ab OR “carer*.ti,ab” OR “carer”.ti,ab OR “carers”.ti,ab OR “informal care*”.ti,ab

AND

MeSH: Quality of life

“Quality of life” OR “outcome measures” OR “caregiver outcomes” OR “time use” OR “occupations” OR “occupational engagement” OR “self-efficacy” OR “self efficacy” OR “experience” OR “emotion*” OR “psychological health impact” OR “physical health impact” OR “confidence” OR “self-confidence” OR “satisfaction” OR “dissatisfaction” OR “activities of daily living” OR “resilience” OR “social adj2 (interaction or engagement or support) OR “Social participation” OR “caregiver adj2 (support or health professional or medical team or nurse or patient or relationship* or knowledge)” OR “information exchange” OR “coping strategies” OR “leisure activity” OR “conflict” OR “caregiver responsib*” OR care-giver responsib*” OR “caregiver expectation” OR “care-giver expectation” OR “caregiver role” OR “care-giver role” OR “role adjustment” OR “caregiver or care-giver adj2 (adjustment)” OR “caregiver or care-giver adj2 (acceptance) OR “caregiver or care-giver adj2 (readiness)” OR “caregiver or care-giver adj2 (attitude to health)”

AND

MeSH: Heart Failure, Treatment Failure, Pulmonary Disease, Chronic Obstructive Pulmonary Disease, Chronic Obstructive Pulmonary Disease, Acute Coronary Syndrome, Coronary Arteriosclerosis, Coronary Artery Bypass, Coronary Disease, Coronary Aneurysm, Stroke OR Coronary Artery Disease

“Heart Failure”.ti,ab OR “cardiac failure”.ti,ab OR “myocardial failure”.ti,ab OR “left ventricular failure”.ti,ab OR “right ventricular failure”.ti,ab OR “cardiomyopathy”.ti,ab, OR “systolic failure”.ti,ab OR “diastolic failure”.ti,ab OR “COPD”.ti,ab OR “chronic obstructive lung disease”.ti,ab OR “pulmonary disease”.ti,ab OR “pulmonary disorder”.ti,ab OR “respiratory disease”.ti,ab OR “respiratory disorder”.ti,ab OR “dyspnea”.ti,ab OR “dyspnoea”.ti,ab OR “Stroke”.ti,ab OR “stroke disability”.ti,ab OR “stroke disease”.ti,ab OR “stroke disorder”.ti,ab OR “CVA”.ti,ab OR “cerebrovascular accident”.ti,ab OR “transient ischaemic attack”.ti,ab OR “transient ischemic attack”.ti,ab OR “haemorrhage”.ti,ab OR “hemorrhage”.ti,ab OR “cerebral haemorrhage”.ti,ab OR “cerebral hemorrhage”.ti,ab OR “aneurysm”.ti,ab OR “atrial fibrillation”.ti,ab OR “Coronary Artery Disease”.ti,ab OR “acute coronary syndrome”.ti,ab OR “atherosclerosis”.ti,ab OR “arteriosclerosis”.ti,ab OR “ischaemic heart disease”,ti.ab OR “ischemic heart disease”.ti,ab OR “myocardial infarction”,ti.ab OR “coronary revascularisation”.ti,ab OR “coronary revascularization”.ti,ab OR “angina”.ti,ab OR “CABG”.ti,ab OR “coronary artery bypass graft”.ti,ab OR “stable angina”.ti,ab OR “angina pectoris”.ti,ab

For peer review only

EMBASE:

MeSH: Caregivers

“Caregivers”.ti,ab OR “care-givers”.ti,ab OR “Caregiver”.ti,ab OR “care-giver”.ti,ab OR “carer*.ti,ab” OR “informal car*”.ti,ab

AND

MeSH: Quality of life

“Quality of life” OR “outcome measures” OR “caregiver outcomes” OR “care-giver outcomes” OR “time use” OR “occupations” OR “occupational engagement” OR (“self-efficacy” OR “self efficacy”) OR “experience” OR “emotion*” OR “psychological health impact” OR “physical health impact” OR (“confidence” OR “self-confidence”) OR “satisfaction” OR “dissatisfaction” OR “activities of daily living” OR “resilience” OR “social adj2 (interaction or engagement or support) OR “Social participation” OR “caregiver OR care-giver adj2 (support or health professional or medical team or nurse or patient or relationship* or knowledge)” OR “information exchange” OR “coping strategies” OR “leisure activity” OR “conflict” OR “caregiver responsib*” OR care-giver responsib*” OR “caregiver expectation” OR “care-giver expectation” OR “caregiver role” OR “care-giver role” OR “role adjustment” OR “caregiver or care-giver adj2 (adjustment or acceptance or readiness)” OR “caregiver or care-giver adj2 (attitude to health)”

AND

MeSH: Heart Failure, Chronic Obstructive Pulmonary Disease, Bronchitis, Chronic Obstructive, Disease exacerbation, Pulmonary Disease, Cardiovascular Disease, Stroke, Cerebrovascular Accident, Coronary Artery Disease, Ischemic Heart Disease, Coronary Artery Disease, Acute Coronary Syndrome, Heart Infarction, Coronary Aneurysm, Coronary Artery Aneurysm, Coronary Artery Bypass, Coronary Artery Bypass Graft, Coronary arteriosclerosis, Coronary Artery Atherosclerosis

“heart failure”,ti,ab OR “cardiac failure”.ti,ab OR “myocardial failure”.ti,ab OR “left ventricular failure”.ti,ab OR “right ventricular failure”.ti,ab OR “cardiomyopathy”.ti,ab, OR “systolic failure”.ti,ab OR “diastolic failure”.ti,ab OR “chronic obstructive pulmonary disease” OR “COPD”.ti,ab OR “chronic obstructive lung disease”.ti,ab OR “pulmonary disease”.ti,ab OR “pulmonary disorder”.ti,ab OR “respiratory disease”.ti,ab OR “respiratory disorder”.ti,ab OR “stroke disorder”.ti,ab OR “CVA”.ti,ab OR “cerebrovascular accident”.ti,ab OR “transient ischaemic attack”.ti,ab OR “transient ischemic attack”.ti,ab OR “haemorrhage”.ti,ab OR “hemorrhage”.ti,ab OR “cerebral haemorrhage”.ti,ab OR “cerebral hemorrhage”.ti,ab OR “aneurysm”.ti,ab OR “atrial fibrillation”.ti,ab OR “Coronary Artery Disease”.ti,ab OR “acute coronary syndrome”.ti,ab OR “atherosclerosis”.ti,ab OR “arteriosclerosis”.ti,ab OR “ischaemic heart disease”,ti.ab OR “ischemic heart disease”.ti,ab OR “myocardial infarction”,ti.ab OR “coronary revascularisation”.ti,ab OR “coronary revascularization”.ti,ab OR “angina”.ti,ab OR “CABG”.ti,ab OR “coronary artery bypass graft”.ti,ab OR “stable angina”.ti,ab OR “angina pectoris”.ti,ab

For peer review only

PsycInfo:

MeSH: Caregivers

“caregiver”.ti,ab OR “care-giver”.ti,ab OR “carer*.ti,ab” OR “informal car*”.ti,ab

AND

MeSH: Quality of life, Occupations, Well-being, Activities of daily living, Mental Health, Health Behaviour, Emotional Responses, Life satisfaction, Role satisfaction, Resilience, Life Experience, Communication, Strategies, Anxiety, Coping Behaviour, Distress, Recreation, Leisure Time, Participation, Adjustment, Couples

“Quality of life” OR “outcome measures” OR (“caregiver outcomes” OR “care-giver outcomes”) OR (“time-use” OR “time use”) OR “occupations” OR “occupational engagement” OR (“self-efficacy” OR “self efficacy”) OR “experience” OR “emotion*” OR “psychological health impact” OR “physical health impact” OR (“confidence” OR “self-confidence” OR self confidence) OR “satisfaction” OR “dissatisfaction” OR “activities of daily living” OR “resilience” OR “social adj2 (interaction or engagement or support or participation) OR “caregiver OR care-giver adj2 (support or health professional or medical team)” OR “information exchange” OR “coping strategies” OR “leisure activity” OR “conflict” OR “caregiver responsib*” OR care-giver responsib*” OR “caregiver expectation” OR “care-giver expectation” OR “caregiver role” OR “care-giver role” OR “role adjustment” OR “caregiver or care-giver adj2 (adjustment or acceptance or readiness)” OR “caregiver or care-giver adj2 (attitude to health)”

AND

MeSH: Heart Failure, Health Behaviour, Heart Disorders, Cardiovascular Disorders, Self-care skills, Intervention, Treatment Outcomes, Chronic Obstructive Pulmonary Disease, Pulmonary Heart Disease, Stroke, Treatment Outcomes, Intervention, Cerebrovascular Accidents, Coronary Artery Disease, Myocardial Infarctions, Ischemia, Aneurysms

“heart failure”,ti,ab OR “cardiac failure”.ti,ab OR “myocardial failure”.ti,ab OR “left ventricular failure”.ti,ab OR “right ventricular failure”.ti,ab OR “cardiomyopathy”.ti,ab, OR “systolic failure”.ti,ab OR “diastolic failure”.ti,ab OR “chronic obstructive pulmonary disease” OR “COPD”.ti,ab OR “chronic obstructive lung disease”.ti,ab OR “pulmonary disease”.ti,ab OR “pulmonary disorder”.ti,ab OR “respiratory disease”.ti,ab OR “respiratory disorder”.ti,ab OR “dyspnea”.ti,ab OR “dyspnoea”,ti,ab OR “stroke disorder”.ti,ab OR “CVA”.ti,ab OR “cerebrovascular accident”.ti,ab OR “transient ischaemic attack”.ti,ab OR “transient ischemic attack”.ti,ab OR “haemorrhage”.ti,ab OR “hemorrhage”.ti,ab OR “cerebral haemorrhage”.ti,ab OR “cerebral hemorrhage”.ti,ab OR “aneurysm”.ti,ab OR “atrial fibrillation”.ti,ab OR “Coronary Artery Disease”.ti,ab OR “acute coronary syndrome”.ti,ab OR “atherosclerosis”.ti,ab OR “arteriosclerosis”.ti,ab OR “ischaemic heart disease”,ti.ab OR “ischemic heart disease”.ti,ab OR “myocardial infarction”,ti.ab OR “coronary revascularisation”.ti,ab OR “coronary revascularization”.ti,ab OR “angina”.ti,ab OR “CABG”.ti,ab OR “coronary artery bypass graft”.ti,ab OR “stable angina”.ti,ab OR “angina pectoris”.ti,ab

For peer review only

Web of Science

TI = Title

TS = Topic

TI & TS = “caregiver” OR “care-giver” OR “carer*” OR “informal care*”

AND

TS = “Quality of life” OR “outcome measures” OR (“caregiver outcomes” OR “care-giver outcomes”) OR (“time-use” OR “time use”) OR “occupations” OR “occupational engagement” OR (“self-efficacy” OR “self efficacy”) OR “experience” OR “emotion*” OR “psychological health impact” OR “physical health impact” OR (“confidence” OR “self-confidence” OR self confidence) OR “satisfaction” OR “dissatisfaction” OR “activities of daily living” OR “resilience” OR “social adj2 (interaction or engagement or support or participation) OR “caregiver OR care-giver adj2 (support or health professional or medical team)” OR “information exchange” OR “coping strategies” OR “leisure activity” OR “conflict” OR “caregiver responsib*” OR care-giver responsib*” OR “caregiver expectation” OR “care-giver expectation” OR “caregiver role” OR “care-giver role” OR “role adjustment” OR “caregiver or care-giver adj2 (adjustment or acceptance or readiness)” OR “caregiver or care-giver adj2 (attitude to health)”

AND

TI & TS = “heart failure” OR “cardiac failure” OR “myocardial failure” OR “left ventricular failure” OR “right ventricular failure” OR “cardiomyopathy” OR “systolic failure” OR “diastolic failure” OR “chronic obstructive pulmonary disease” OR “COPD” OR “chronic obstructive lung disease” OR “pulmonary disease” OR “pulmonary disorder” OR “respiratory disease” OR “respiratory disorder” OR “dyspnea” OR “dyspnoea” OR “stroke disorder” OR “CVA” OR “cerebrovascular accident” OR “transient ischaemic attack” OR “transient ischemic attack” OR “haemorrhage” OR “hemorrhage” OR “cerebral haemorrhage” OR “cerebral hemorrhage” OR “aneurysm” OR “atrial fibrillation” OR “Coronary Artery Disease” OR “acute coronary syndrome” OR “atherosclerosis” OR “arteriosclerosis” OR “ischaemic heart disease” OR “ischemic heart disease” OR “myocardial infarction” OR “coronary revascularisation” OR “coronary revascularization” OR “angina” OR “CABG” OR “coronary artery bypass graft” OR “stable angina” OR “angina pectoris”

For peer review only

ProQuest: Global Dissertations & Theses/ Applied Social Sciences Index and Abstracts (ASSIA)

“caregiver”.ti,ab OR “care-giver”.ti,ab OR “carer*.ti,ab” OR “informal car*”.ti,ab

AND

“Quality of life” OR “outcome measures” OR (“caregiver outcomes” OR “care-giver outcomes”) OR (“time-use” OR “time use”) OR “occupations” OR “occupational engagement” OR (“self-efficacy” OR “self efficacy”) OR “experience” OR “emotion*” OR “psychological health impact” OR “physical health impact” OR (“confidence” OR “self-confidence” OR self confidence) OR “satisfaction” OR “dissatisfaction” OR “activities of daily living” OR “resilience” OR “social adj2 (interaction or engagement or support or participation) OR “caregiver OR care-giver adj2 (support or health professional or medical team)” OR “information exchange” OR “coping strategies” OR “leisure activity” OR “conflict” OR “caregiver responsib*” OR care-giver responsib*” OR “caregiver expectation” OR “care-giver expectation” OR “caregiver role” OR “care-giver role” OR “role adjustment” OR “caregiver or care-giver adj2 (adjustment or acceptance or readiness)” OR “caregiver or care-giver adj2 (attitude to health)”

AND

“heart failure”,ti,ab OR “cardiac failure”.ti,ab OR “myocardial failure”.ti,ab OR “left ventricular failure”.ti,ab OR “right ventricular failure”.ti,ab OR “cardiomyopathy”.ti,ab, OR “systolic failure”.ti,ab OR “diastolic failure”.ti,ab OR “chronic obstructive pulmonary disease” OR “COPD”.ti,ab OR “chronic obstructive lung disease”.ti,ab OR “pulmonary disease”.ti,ab OR “pulmonary disorder”.ti,ab OR “respiratory disease”.ti,ab OR “respiratory disorder”.ti,ab OR “dyspnea”.ti,ab OR “dyspnoea”,ti,ab OR “stroke disorder”.ti,ab OR “CVA”.ti,ab OR “cerebrovascular accident”.ti,ab OR “transient ischaemic attack”.ti,ab OR “transient ischemic attack”.ti,ab OR “haemorrhage”.ti,ab OR “hemorrhage”.ti,ab OR “cerebral haemorrhage”.ti,ab OR “cerebral hemorrhage”.ti,ab OR “aneurysm”.ti,ab OR “atrial fibrillation”.ti,ab OR “Coronary Artery Disease”.ti,ab OR “acute coronary syndrome”.ti,ab OR “atherosclerosis”.ti,ab OR “arteriosclerosis”.ti,ab OR “ischaemic heart disease”,ti.ab OR “ischemic heart disease”.ti,ab OR “myocardial infarction”,ti.ab OR “coronary revascularisation”.ti,ab OR “coronary revascularization”.ti,ab OR “angina”.ti,ab OR “CABG”.ti,ab OR “coronary artery bypass graft”.ti,ab OR “stable angina”.ti,ab OR “angina pectoris”.ti,ab

For peer review only

Ethos – British Library

CAREGIVER or CARE-GIVER.ti,ab

Searches initially included Heart Failure, Chronic Obstructive Pulmonary Disease, Stroke and Coronary Artery Disease as "AND" terms, however these yielded no results

For peer review only

Table 2 (a)

Caregiver experience - Concepts/Mapping Table – Qualitative Papers

MENTAL HEALTH = 1 **ROLE = 2** **LIFESTYLE CHANGE = 3**
SUPPORT = 4 **KNOWLEDGE/INFORMATION = 5**
RELATIONSHIPS = 6

Concepts only in Qualitative Papers: **EXPERT BY EXPERIENCE = 7** **VIGILANCE = 8**
TIME = 9 **SHARED CARE = 10**

	**1**	**2**	**3**	**4**	**5**	**6**	**7**	**8**	**9**	**10**
Andersen (2013) **(HF)**	✓		✓	✓	✓					✓
Baker (2010) **(HF)**	✓	✓	✓	✓		✓		✓		✓
Bove (2016) **(COPD)**	✓	✓	✓	✓	✓	✓	✓	✓	✓	✓
Burke (2014) **(HF)**	✓	✓	✓	✓	✓	✓	✓	✓		✓
Clark (2007) **(HF)**		✓	✓	✓	✓	✓	✓	✓	✓	✓
Figueiredo (2015) **(COPD)**	✓	✓	✓	✓	✓	✓	✓	✓	✓	
• Halm (2016) **(CAD)**	✓	✓	✓	✓	✓	✓	✓	✓	✓	✓
• Halm (2017) **(CAD)**	✓	✓	✓	✓	✓	✓	✓	✓	✓	✓
Hynes (2012) **(COPD)**	✓	✓	✓	✓	✓	✓	✓	✓	✓	✓
Imes (2011) **(HF)**	✓	✓	✓	✓	✓	✓		✓	✓	✓
Kitko (2010) **(HF)**	✓	✓	✓	✓	✓	✓	✓	✓	✓	✓
Liljeroos (2014) **(HF)**	✓		✓	✓	✓	✓		✓	✓	✓
Lindqvist (2012) **(COPD)**	✓	✓	✓	✓	✓	✓		✓	✓	✓
Luttik (2007) **(HF)**	✓	✓	✓	✓	✓	✓	✓	✓	✓	✓
Marcuccilli (2011) **(HF)**	✓		✓	✓		✓	✓	✓	✓	✓
Marcuccilli (2014) **(HF)**	✓	✓	✓	✓	✓	✓		✓	✓	
Näsström (2017) **(HF)**		✓	✓	✓	✓	✓	✓	✓	✓	✓
Pattenden (2007) **(HF)**	✓	✓	✓	✓		✓		✓		
Rolley (2010) **(CAD)**	✓	✓	✓	✓	✓	✓		✓		

Spence (2008) **(COPD)**	✓	✓	✓	✓	✓	✓	✓	✓	✓	✓
Strom (2015) **(HF)**	✓	✓	✓	✓		✓	✓	✓		✓
Wallin (2013) **(CAD)**	✓	✓	✓	✓	✓	✓			✓	
Wingham (2015) **(HF)**	✓	✓	✓	✓	✓	✓	✓	✓	✓	✓
**Heart Failure**	**12**	**11**	**14**	**14**	**10**	**13**	**8**	**13**	**9**	**12**
**COPD**	**5**	**5**	**5**	**5**	**5**	**5**	**4**	**5**	**5**	**4**
**CAD**	**4**	**4**	**4**	**4**	**4**	**4**	**2**	**3**	**3**	**2**
**Total**	**21**	**20**	**23**	**23**	**19**	**22**	**14**	**21**	**17**	**18**

• = same study

For peer review only

Table 2 (b)

Caregiver experience - Concepts/Mapping – Quantitative Papers

MENTAL HEALTH IMPACT = 1 **ROLE = 2** **LIFESTYLE CHANGE = 3**
SUPPORT = 4 **KNOWLEDGE/INFORMATION = 5**
RELATIONSHIPS = 6

	**1**	**2**	**3**	**4**	**5**	**6**
Agren (2010) **(HF)**	✓					✓
Al-Rawashdeh (2017) **(HF)**	✓		✓			✓
Badr (2017) **(COPD)**	✓		✓			✓
Bakas (2006) **(HF)**	✓	✓	✓			
Chung (2016) **(HF)**	✓	✓	✓			
Cossette (1993) **(COPD)**	✓	✓		✓		
Evangelista (2002) **(HF)**	✓					✓
Figueiredo (2013) **(COPD)**	✓		✓			✓
Figueiredo (2014) **(COPD)**	✓					✓
Grigorovich (2017) **(HF)**	✓		✓	✓		
• Halm (2006) **(CAD)**	✓			✓		✓
• Halm (2007) **(CAD)**	✓			✓		✓
Hess (2009) **(HF)**		✓				
Hooley (2005) **(HF)**	✓					✓
Hwang (2011) **(HF)**	✓	✓		✓		✓
Karmilovich (1994) **(HF)**	✓	✓			✓	✓
Kneeshaw (1999) **(CAD)**		✓	✓	✓	✓	✓
Loftus (2004) **(HF)**	✓	✓		✓		✓
Lum (2014) **(HF)**						✓
Luttik (2009) **(HF)**						✓

Miravitlles (2015) **(COPD)**			✓			✓
Nakken (2017) **(COPD)**	✓		✓	✓		✓
Näsström (2017) **(HF)**	✓			✓	✓	✓
Park (2013) **(CAD)**		✓	✓			✓
Pi-Ming Yeh (2011) **(HF)**	✓			✓		
Pressler (2013) **(HF)**	✓	✓	✓			✓
Riegner (1996) **(COPD)**		✓		✓		✓
• Saunders (2008) **(HF)**	✓			✓		✓
• Saunders (2009) **(HF)**	✓			✓		✓
Saunders (2010) **(HF)**	✓	✓				✓
Schwarz (2003) **(HF)**	✓			✓		
Scott (2000) **(HF)**	✓	✓	✓	✓		
Takata (2008) **(COPD)**	✓	✓	✓	✓	✓	
Vellone (2015) **(HF)**	✓				✓	✓
Woolfe (2007) **(COPD)**	✓		✓	✓	✓	
**Heart Failure**	**19**	**9**	**6**	**9**	**3**	**15**
**COPD**	**5**	**3**	**6**	**5**	**2**	**6**
**CAD**	**4**	**2**	**2**	**3**	**1**	**4**
**Total**	**28**	**14**	**14**	**17**	**6**	**25**

• = same study

For peer review only

PRISMA 2009 Checklist

Section/topic	#	Checklist item	Reported on page #
**TITLE**			
Title	1	Identify the report as a systematic review, meta-analysis, or both.	1
**ABSTRACT**			
Structured summary	2	Provide a structured summary including, as applicable: background; objectives; data sources; study eligibility criteria, participants, and interventions; study appraisal and synthesis methods; results; limitations; conclusions and implications of key findings; systematic review registration number.	2
**INTRODUCTION**			
Rationale	3	Describe the rationale for the review in the context of what is already known.	4
Objectives	4	Provide an explicit statement of questions being addressed with reference to participants, interventions, comparisons, outcomes, and study design (PICOS).	4,5
**METHODS**			
Protocol and registration	5	Indicate if a review protocol exists, if and where it can be accessed (e.g., Web address), and, if available, provide registration information including registration number.	2
Eligibility criteria	6	Specify study characteristics (e.g., PICOS, length of follow-up) and report characteristics (e.g., years considered, language, publication status) used as criteria for eligibility, giving rationale.	5
Information sources	7	Describe all information sources (e.g., databases with dates of coverage, contact with study authors to identify additional studies) in the search and date last searched.	5
Search	8	Present full electronic search strategy for at least one database, including any limits used, such that it could be repeated.	5
Study selection	9	State the process for selecting studies (i.e., screening, eligibility, included in systematic review, and, if applicable, included in the meta-analysis).	5
Data collection process	10	Describe method of data extraction from reports (e.g., piloted forms, independently, in duplicate) and any processes for obtaining and confirming data from investigators.	5
Data items	11	List and define all variables for which data were sought (e.g., PICOS, funding sources) and any assumptions and simplifications made.	5,6
Risk of bias in individual studies	12	Describe methods used for assessing risk of bias of individual studies (including specification of whether this was done at the study or outcome level), and how this information is to be used in any data synthesis.	5,6,18,19,20
Summary measures	13	State the principal summary measures (e.g., risk ratio, difference in means).	5,6
Synthesis of results	14	Describe the methods of handling data and combining results of studies, if done, including measures of consistency (e.g., I^2) for each meta-analysis.	6

For peer review only

PRISMA 2009 Checklist

Section/topic	#	Checklist item	Reported on page #
Risk of bias across studies	15	Specify any assessment of risk of bias that may affect the cumulative evidence (e.g., publication bias, selective reporting within studies).	5,6,18,19,20
Additional analyses	16	Describe methods of additional analyses (e.g., sensitivity or subgroup analyses, meta-regression), if done, indicating which were pre-specified.	6,7
**RESULTS**			
Study selection	17	Give numbers of studies screened, assessed for eligibility, and included in the review, with reasons for exclusions at each stage, ideally with a flow diagram.	8
Study characteristics	18	For each study, present characteristics for which data were extracted (e.g., study size, PICOS, follow-up period) and provide the citations.	9
Risk of bias within studies	19	Present data on risk of bias of each study and, if available, any outcome level assessment (see item 12).	5,6,18,19,20
Results of individual studies	20	For all outcomes considered (benefits or harms), present, for each study: (a) simple summary data for each intervention group (b) effect estimates and confidence intervals, ideally with a forest plot.	9, 17
Synthesis of results	21	Present results of each meta-analysis done, including confidence intervals and measures of consistency.	22-28
Risk of bias across studies	22	Present results of any assessment of risk of bias across studies (see Item 15).	5,6,18,19,20
Additional analysis	23	Give results of additional analyses, if done (e.g., sensitivity or subgroup analyses, meta-regression [see Item 16]).	
**DISCUSSION**			
Summary of evidence	24	Summarize the main findings including the strength of evidence for each main outcome; consider their relevance to key groups (e.g., healthcare providers, users, and policy makers).	28,29,30,31,
Limitations	25	Discuss limitations at study and outcome level (e.g., risk of bias), and at review-level (e.g., incomplete retrieval of identified research, reporting bias).	30,31
Conclusions	26	Provide a general interpretation of the results in the context of other evidence, and implications for future research.	32
**FUNDING**			
Funding	27	Describe sources of funding for the systematic review and other support (e.g., supply of data); role of funders for the systematic review.	32

From: Moher D, Liberati A, Tetzlaff J, Altman DG, The PRISMA Group (2009). Preferred Reporting Items for Systematic Reviews and Meta-Analyses: The PRISMA Statement. PLoS Med 6(7): e1000097. doi:10.1371/journal.pmed1000097

For more information, visit: **www.prisma-statement.org**.

For peer review only - http://bmjopen.bmj.com/site/about/guidelines.xhtml

BMJ Open

"Who Cares?" The Experiences of Caregivers of Adults Living with Heart Failure, Chronic Obstructive Pulmonary Disease and Coronary Artery Disease – A Mixed Methods Systematic Review

Journal:	*BMJ Open*
Manuscript ID	bmjopen-2017-020927.R1
Article Type:	Research
Date Submitted by the Author:	16-Feb-2018
Complete List of Authors:	Noonan, Miriam; University of Exeter Medical School, European Centre for Environment and Human Health<br>Wingham, Jennifer; Royal Cornwall Hospitals NHS Trust, Research, Development and Innovation; University of Exeter Medical School,<br>Taylor, Rod; University of Exeter, Peninsula Medical School
**Primary Subject Heading**:	Patient-centred medicine
Secondary Subject Heading:	Evidence based practice, Cardiovascular medicine, Respiratory medicine, Research methods, Health services research
Keywords:	Caregivers, Heart failure < CARDIOLOGY, Coronary heart disease < CARDIOLOGY, Chronic obstructive pulmonary disease, mixed methods systematic review

SCHOLARONE™ Manuscripts

For peer review only

For peer review only - http://bmjopen.bmj.com/site/about/guidelines.xhtml

"Who Cares?" The Experiences of Caregivers of Adults Living with Heart Failure, Chronic Obstructive Pulmonary Disease and Coronary Artery Disease – A Mixed Methods Systematic Review

M Noonan[1], J Wingham[2,3] , RS Taylor[4]

Author Affiliations

1. Miriam Noonan University of Exeter Medical School, European Centre for Environment and Human Health, Knowledge Spa, Royal Cornwall Hospital, Truro, Cornwall, UK TR13HD, mn354@exeter.ac.uk

2. Dr. Jennifer Wingham Royal Cornwall Hospitals NHS Trust, Research, Development and Innovation, F37, Knowledge Spa, Royal Cornwall Hospital, TRURO, Cornwall, UK TR1 3HD,
3. University of Exeter Medical School,
Exeter, UK EX2 4SG

4. Professor Rod S. Taylor, University of Exeter, Peninsula Medical School, Exeter, Cornwall, UK

Correspondence to
Miriam C. Noonan; **mn354@exeter.ac.uk**

(Word Count 4,035)

ABSTRACT

For peer review only - http://bmjopen.bmj.com/site/about/guidelines.xhtml

Objective To assess the experiences of unpaid caregivers providing care to people with heart failure (HF) or chronic obstructive pulmonary disease (COPD) or coronary artery disease (CAD).

Design Mixed methods systematic review including qualitative and quantitative studies.

Data Sources Databases searched: Medline Ebsco, PsycInfo, CINAHL Plus with Full Text, Embase, Web of Science, Ethos: The British Library and ProQuest. Grey literature identified using: Global Dissertations and Theses and Applied Sciences Index and hand searches and citation checking of included references. Search time frame: 1st January 1990 to 30th August 2017.

Eligibility criteria for selecting studies Inclusion was limited to English language studies in unpaid adult caregivers (>18 years), providing care for patients with HF, COPD or CAD. Studies that considered caregivers for any other diagnoses and studies undertaken in low and middle income countries were excluded. Quality assessment of included studies was conducted by two authors.

Data analysis/synthesis - A results based convergent synthesis was conducted

Results Searches returned 8,026 titles and abstracts. 54 studies - 21 qualitative, 32 quantitative, and 1 mixed were included. This totalled 26,453 caregivers who were primarily female (63 %), with average age of 62.2 years. Narrative synthesis yielded six concepts related to caregiver experience: (1) mental health (2) caregiver role (3) lifestyle change (4) support for caregivers (5) knowledge and (6) relationships. There was a discordance between paradigms regarding emerging concepts. Four concepts emerged from qualitative papers which were not present in quantitative papers (1) expert by experience (2) vigilance (3) shared care and (4) time.

Conclusion Caregiving is life altering and complex with significant health implications. Health professionals should support caregivers who in turn can facilitate the recipient to manage their long term condition. Further longitudinal research exploring the evolution of caregiver experiences over time of patients with chronic cardiopulmonary conditions is required.

For peer review only

For peer review only - http://bmjopen.bmj.com/site/about/guidelines.xhtml

Strengths and limitations of this study

- This mixed methods systematic review provides the opportunity for a broadened and deeper understanding of the literature on the experiences of unpaid caregivers' providing care to people with heart failure, chronic obstructive pulmonary disease, and coronary artery disease.
- This review provides an integration of qualitative and quantitative predictors of caregiver experiences.
- To maximise applicability we included studies from higher income countries only.
- Quality of evidence limited by assessment of caregiver experience at single point of time and there is need for future studies that employ longitudinal or repeated measures design.

Systematic review registration The study protocol was registered on PROSPERO (CRD42016053412).

For peer review only

For peer review only - http://bmjopen.bmj.com/site/about/guidelines.xhtml

INTRODUCTION

A caregiver is anyone providing unpaid care, to a friend or family member who is unable to care for themselves.[1] This may be emotional support; someone to talk to, or practical support; dressing wounds, mobility assistance or medication checking.[2] Globally there are 43.5million caregivers in the United States, 2.86 million in Australia and 6.5million in the UK.[3] Between 2001 to 2011 the number of unpaid caregivers in the UK grew at a faster rate than population growth.[4] The annual value of unpaid care provided to an individual with a chronic illness is estimated to be £132billion.[5]

Focus groups examining a caregivers' life conducted by "The Institute of Public Care" (2017), based at Oxford Brookes University; described caregivers as the 'Skilled Helper' performing a series of roles.[6] Seltzer & Wailing (2000) describe a dynamic process of transitions to being a caregiver.[7] These transitions comprise participating in the role before identifying as a caregiver, acceptance of the role, engaging in it with awareness and sometimes moving beyond the caregiving role when the patient moving to paid care settings or bereavement occurs. This process is not linear and people move through the different transitions at varying rates. Acknowledging this, it is imperative for caregivers to receive a caregiver needs assessment as legally stipulated by the 2014 Care Act. [8] Additionally, the National Institute for Health and Care Excellence (NICE) clinical guidelines for heart failure (HF) (CG108)[9] and chronic obstructive pulmonary disease (COPD) (CG101)[10] both recommend that family members or caregivers are provided with support and included in discussions about care.

Cardio-pulmonary disease is a primary cause of illness. Cardiovascular disease is responsible for 45% deaths in Europe[11] and 1 in 4 deaths in the United States.[12] By 2020, COPD is projected to be in the global top five of diagnoses causing years lost through early mortality or disability-adjusted life years.[13]Caregivers of HF patients have a multitude of unmet needs due to fluctuations in the trajectory of HF.[14] COPD has frequent unplanned hospital admissions and a high morbidity rate.[15] Caregivers experience depressed mood, greater anxiety and increased subjective burden when their support needs are not met.[16,17] The unpredictability of HF and COPD leads to caregivers constantly adjusting their role, creating a need to continuously re-asses what caregiver needs are.[18,19] Spousal caregivers of myocardial infarct patients experience increased levels of stress, lifestyle impact and emotional distress.[20] Caring for coronary artery bypass graft patients in tasks such as monitoring and provision of emotional support increased caregiver burden to a level described as moderate.[21] COPD and cardiovascular disease are both increasing in prevalence and frequently co-exist. [22, 23] We know of no systematic review that synthesises quantitative and qualitative studies to combine caregivers' experiences of people with HF, COPD or coronary artery disease (CAD).

For peer review only - http://bmjopen.bmj.com/site/about/guidelines.xhtml

Using a mixed methods systematic review methodology including both qualitative and quantitative literature, this study aims to understand the experiences of adult caregivers when supporting people with HF, COPD or CAD.

METHODS

We conducted and reported this systematic review in accordance with the PRISMA (Preferred Reporting Items for Systematic Reviews and Meta-Analyses) statement.[24]

Study design

This study employed a mixed methods systematic review assessing both qualitative and quantitative studies.[25] The rationale for using a mixed methods review approach was multifaceted. Firstly, to gain a qualitative assessment of the quantitative predictors of experiences of caregivers. Secondly, to develop a holistic perspective of what caregiver's experience. Third, we wanted to assess the degree of convergence between qualitative and quantitative experiences.

Search Strategy

Our search strategy was designed in conjunction with a Health Services Librarian and Information Specialists. Search terms included condition specific terms, i.e., "heart failure", "COPD" and "coronary artery disease", caregiver specific, plus experience related terms, "experience", "quality of life" "activities of daily living", "occupational engagement", "time use", "self-efficacy", "coping strategies", "leisure activity", "information exchange" and "caregiver expectation"". (See supplementary table 1 for complete list of search terms.) Databases searched included: Medline Ebsco, PsycInfo, CINAHL Plus with Full Text, Embase, Web of Science, Ethos: The British Library and ProQuest. Grey literature was identified using Global Dissertations and Theses and Applied Sciences Index and hand searches and citation checking of included references. To ensure the contemporary nature of the evidence considered, the search time frame was January 1990 to August 2017. A single researcher (MN) initially screened titles and abstracts. Selection of full papers was performed by two researchers (MN and either JW or RST).

Study Selection

Studies were included if they addressed 'caregiver experience' which was defined as encompassing the daily activities of caregivers and the impact of these activities on their lives. Studies were cross-checked by a single researcher (MN) with the eligibility criteria. These were English language studies involving unpaid adult caregivers (>18 years), providing care for patients with HF, COPD or CAD living in the community and not residential settings with paid care staff. Qualitative, quantitative and grey literature studies were all included in the search strategy. Conference papers were excluded.

For peer review only - http://bmjopen.bmj.com/site/about/guidelines.xhtml

Outcomes of interest included psychological and physical outcomes reported, occupational engagement and routine. As we sought to inform the practice of UK and other high income countries we excluded studies undertaken in low and middle income countries. [26]

Data extraction

Data extracted from retained studies included: study design, sample and recruitment, study description, method, findings, discussion and author's conclusions and limitations. Caregiver quotes were extracted from qualitative studies. For quantitative studies, data extraction also included details of attrition and data analysis.

Study quality assessment

Qualitative studies were appraised utilising the Critical Appraisal (CASP) Tool.[27] Only high quality qualitative studies were included for synthesis.[28] It is difficult to apply checklists when appraising qualitative research, although it is imperative to have criteria to appraise qualitative papers. When synthesising qualitative data exclusion of studies of insufficient quality is important.[29] This will facilitate a synthesis of evidence. In absence of an existing quality tool that could be used to appraise quantitative studies addressing the specific question of this study, a quality assessment tool was developed by the research team based on what were deemed to be the appropriate core biases i.e. (1) was the sample recruited appropriately? (2) Was the level of attrition acceptable? (3) Was a validated quantitative outcome(s) used? (4) Were the methods of data analysis appropriate? A score of 1 was attributed to each study based on these four questions. Based on their quality assessment, studies were scored and ranked; 1 or 2 "low quality", 3 "medium quality" and 4 or 5 "high quality". . Data Extraction and quality appraisal was first conducted by a single researcher (MN) and checked by one of two researchers (JW or RST).

Data analysis and synthesis

The methodology of mixed methods data synthesis is an emerging one and no single approach has yet been universally accepted.[30] In this study a results based convergent design was chosen.[31,32] This requires transformation of one method into another. Due to the heterogeneity of the quantitative methods a meta-analysis was not appropriate. Instead, applying a narrative profile formation, quantitative data was converted into qualitative data.[33] Extracted data from quantitative and qualitative studies were imported into separate spreadsheets. A meta-ethnographic approach was utilised to synthesise qualitative studies.[34] A narrative formation approach[35] was utilised to synthesise the quantitative data into a qualitative data set. Narrative formation is a verbal description via the use of profiles of each of the studies. The five profiles are modal, average, holistic, comparative and normative.[35] Table 1 provides an example of this approach. This resulted in

For peer review only - http://bmjopen.bmj.com/site/about/guidelines.xhtml

two qualitative data sets[36] from which concepts emerged. A mapping table was completed in order to provide an audit trail of how the overall concepts across all papers were derived (see supplementary file 2a, 2b and 2c). Initial synthesis was conducted by a single researcher (MN) and corroborated by two experienced researchers in quantitative (RST) and qualitative (JW) research.

Table 1

Narrative Formation

Profile	Explanation	Study	Extracted from study	Emerging from study	Concept
**Modal**	This is a narrative description of the group being studied	Chung 2012	102 dyads, predominantly spousal caregivers. Group is caregivers of patients with HF. Comparing depressed and non-depressed HF pts and the caregiver experience as a result of this.		Role – spouse as a caregiver
**Average**	This is a detailed narrative description based on the mean (average) attributes of the individuals/situations being studied		Caregiver Mean age 56.7, 78% female, white (94%).<br>41% of pts NYHA III-IV<br>42% caregivers reported severe burden (the Zarit Burden Interview (ZBI)≥17)”<br><br>“Caregivers of patients with depressive symptoms had a higher level of burden (25±13 vs 13.5±12 on the ZBI; p<0.001), spent more time caregiving	Impact of patient mental health on caregiver<br><br>Lifestyle change – restrictions<br><br>Time spent caregiving = higher burden<br><br>Relationship between patient and caregiver i.e. patient mental health has an impacting role on caregiver subjective	Mental Health Routine/Lifestyle

For peer review only - http://bmjopen.bmj.com/site/about/guidelines.xhtml

			(37±12 vs 30±11 on the Oberst Caregiving Burden Scale; p=0.004), and reported worse mental quality of life (46±10 vs 51±10 on the SF-12v2; p=0.026) than those of patients without depressive symptoms."	experience	
**Comparative**	This is a description comparing studies and comparing individuals being studied.		Caregivers who provided care for depressed patients reported higher burden related to social life limitations, poor perceived control, stress of family obligations, and patients' dependency	Patient Illness severity impacting on caregiver	Relationship – patient illness and caregiver response to this subjective and objective
**Normative**	A comparison of the study individuals with the general population			Greater impact on caregivers lives when patients were depressed	
**Holistic (also called inferential or summative)**	A description of the overall perception of the investigator			Female, white caregivers, experienced greater levels of burden, loss of roles and greater distress when patients	

For peer review only - http://bmjopen.bmj.com/site/about/guidelines.xhtml

				were depressed. This could be due to the increased need for practical and emotional support, feeling they need to be constantly present	

For peer review only - http://bmjopen.bmj.com/site/about/guidelines.xhtml
For peer review only

RESULTS

Study selection

Study selection process is summarised in a PRISMA Flow diagram shown in Figure 1. Following removal of duplicates the search strategy yielded a total of 8,026 title and abstracts. Of these 242 full papers were reviewed, of which 57 papers (54 studies) were included for synthesis. A detailed summary of included studies is provided in Table 2.

For peer review only

For peer review only - http://bmjopen.bmj.com/site/about/guidelines.xhtml

PRISMA Flow Diagram Figure 1

For peer review only

For peer review only - http://bmjopen.bmj.com/site/about/guidelines.xhtml

Table 2

Summary of included studies

First Author/Year	Diagnosis	Aims	Key Conclusions	Methods	Country	Data Collection Sampling	Participants Caregiver (Time caring)	Mean Age (Caregiver /Recipient)
Ågren (2010)	HF (NYHA II-IV)	Describe the levels and Identify independent predictors of cg. burden. in partners of pts with HF.	A partner-centred approach is essential to educate and support partners of pts. with HF and to improve the life situation for patient–partner dyads	QUANT (Correlational)	SWEDEN	Cross Sectional Purposive	135 (101F, 34M) (N/S)	69/71
Al-Rawashdeh (2017)	HF (NYHA I-IV)	To examine whether individuals' sleep disturbance predicted their own, & their partners' QoL in HF i.e. sleep cg. dyads pt–spousal	Pts & their spousal cgs. have interdependent relationships that influence each other's outcomes	QUANT	USA	Cross Sectional Purposive	78 Dyads (58F, 20M) (N/S)	62.2./59.5
Andersen (2015)	HF	Obtain knowledge on experiences and views and the desire for knowledge of family cgs of HF pts and competence and support the cgs need to manage the situation	Experience of responsibility Need for more knowledge Mixed experience of health services	QUAL	NORWAY	Interviews Convenience	19 (17F, 2M) (N/S)	63/N/ S
Badr (2017)	COPD	Individual-level predictors of pt and cgs depression in COPD as well as how dyad members effect each other's depression	Similar factors predict pt and cg depression in COPD. Women are at increased risk for developing depression when their partners are depressed	QUANT	USA	Cohort Study Purposive	89 (68F, 21M) (N/S)	54.8/67

For peer review only - http://bmjopen.bmj.com/site/about/guidelines.xhtml

Summary of included studies

First Author/Year	Diagnosis	Aims	Key Conclusions	Methods	Country	Data Collection Sampling	Participants Caregiver (Time caring)	Mean Age (Caregiver /Recipient)
Bakas (2006)	HF (NYHA II-IV)	Examine relationships among age, perceived control over managing HF perceived difficulty with tasks, perceived outcomes, and perceived mental and general health among cgs of persons with HF	Poorer perceived mental health associated with younger age, poorer perception of control greater perceived task difficulty and poorer cg perceived outcomes,	QUANT (Descriptive Correlational)	USA	Cross Sectional Convenience	21 (20F, 1M) (N/S)	59.6/62.7
Baker (2010)	HF (LVAD in situ)	To describe experiences of cgs of pts who received LVAD therapy as a bridge to transplantation	Cgs. successfully incorporated the role of cg for pts with a complex therapeutic regimen into their daily lives	QUAL (Phenomenological) (Descriptive)	USA	Interviews Convenience	6 (5F, 1M) (26-372 days)	51/N/S
Bove (2016)	COPD (GOLD C&D)	Explore how spouses of pts with severe COPD experience their role	Cgs. wanted to provide meaningful care for their partners, but sought knowledge and support from health professionals	QUAL	DENMARK	Focus Groups Purposive	22(13F, 9M) (N/S)	69.4/N/S
Burke (2014)	HF (NYHA II-IV)	Understand what roles cgs perceive and desire for themselves, and to compare and contrast these roles with those they perceive to be desired by the health care system	Disconnect between health professionals and cgs. There is a need to formalise cg role and involve cgs in disease management interventions	QUAL (Inductive)	USA	Interviews Purposive	20 (18F, 1M, 1N/S) (<1 - > 8 hours per week)	59/64

For peer review only - http://bmjopen.bmj.com/site/about/guidelines.xhtml

Summary of included studies

First Author/Year	Diagnosis	Aims	Key Conclusions	Methods	Country	Data Collection Sampling	Participants Caregiver (Time caring)	Mean Age (Caregiver /Recipient)
Chung (2016)	HF (NYHA II-IV)	Examine differences in cg outcomes between cgs who care for HF pts with and without depressive symptoms	Cgs of pts with depressive symptoms had significantly higher levels of cg burden and worse quality of life compared to those caring for pts without depressive symptoms	QUANT	USA	Cross Sectional Convenience	102 Dyads (79F, 23M) (N/S)	56.7/61.4
Clark (2008)	HF (NYHA II – IV)	To examined the complexity of caregiving for pts with HF	Caring for HF is not solely task orientated but informed and sophisticated like professional care	QUAL	SCOTLAND	Interviews Convenience	30 (23F, 7M) (N/S)	68F, 67M/N/S
Cossette (1993)	COPD (GOLD III-V)	Examine relationship between type, number and disturbance of caring tasks and impact on mental health of cg's Examine influence of social support	The relationship between mental health and task varied according to category The number of supervision tasks is a predictor of mental health Caregiving tasks can explain use of psychotropic drugs	QUANT	CANADA	Cross Sectional Convenience	89 (F) (Mean = 13yrs)	65/68.6
Evangelista (2002)	HF (NYHA I – IV)	Describe emotional wellbeing of HF pts and cgs Identify factors associated with of pts emotional wellbeing Determine gender differences in emotional wellbeing of pts and cgs	Emotional wellbeing of cgs is associated with emotional wellbeing	QUANT (Descriptive) (Correlational)	USA	Cross Sectional Convenience	103 Dyads (73F, 30M) (N/S)	59.4/57.6

For peer review only - http://bmjopen.bmj.com/site/about/guidelines.xhtml

Summary of included studies

First Author/Year	Diagnosis	Aims	Key Conclusions	Methods	Country	Data Collection Sampling	Participants Caregiver (Time caring)	Mean Age (Caregiver /Recipient)
Figueiredo (2013)	COPD (GOLD I-IV)	Examine coping strategies of family cg's of pts with early and advanced COPD and how those relate to their subjective health	Cgs of people with advanced and early COPD cope differently with caring demands Problem focused coping strategies helped both groups of cgs	QUANT (Correlational)	PORTUGAL	Cross Sectional Convenience	158 (120F, 38M) (> 4 years)	58.4(early COPD) 60.8 (Adv. COPD) /69.4
Figueiredo (2014)	COPD (GOLD I-IV)	To analyse subjective burden of family cg's of early and advanced COPD pts. and its predictor variables	Caregiving overall subjective burden is associated with poorer self-rated health and psychological well-being.	QUANT (Correlational)	PORTUGAL	Cross Sectional Convenience	167 (125F, 42M) (> 4 years)	58.3(early COPD) 60.5 (Adv. COPD) /69.3
Figueiredo (2015)	COPD (Mod – Severe)	Obtain knowledge on experience of husbands &sons providing care to a family member	Men are committed and dedicated cgs Differences between husbands & sons were present related to: "meaning", challenges and constraints" "fears and concerns about the future" "needs" and "positive aspects of caring"	QUAL	PORTUGAL	Interviews Purposive	12 (M) (> 4 years)	70.9(Husbands) 43.4 (Sons)/72.1
Grigorovich (2017)	HF (NYHA II-IV)	To examine changes in cgs wellbeing over time. Identify pt and cg factors associated with positive and higher negative outcomes	Cg negative emotion remained stable over time. Depression associated with participation restriction for cgs More personal gain and support resulted in positive affect	QUANT	CANADA	Longitudinal (repeated measures) Convenience	50 (31F, 19M) (Mean = 18 months)	58/61.6

For peer review only

For peer review only - http://bmjopen.bmj.com/site/about/guidelines.xhtml

Summary of included studies

First Author/Year	Diagnosis	Aims	Key Conclusions	Methods	Country	Data Collection Sampling	Participants Caregiver (Time caring)	Mean Age (Caregiver /Recipient)
*Halm et al. (2006)<br>*Halm et al. (2007)	CAD	To determine cg burden after CABG surgery 12 months	Cg burden not significantly different post CABG at 3,6 or 12 months.<br>Lower burden associated with better pt health and higher cg mental health	QUANT	USA	Cross Sectional<br>Convenience	166(136F, 30M)<br>(≤12 months)	64.7/66.8
*Halm (2016)<br>*Halm (2017)	CAD	To describe the concerns needs, strategies and advice of CABG cg's during the first 3 months post- surgery<br>To explore cg burden by age and gender	There is a need for a dedicated cg program to prepare partners for their role	QUAL	USA	Interviews<br>Purposive	32(16F, 16M)<br>(N/S)	60.6 (M<70) /60.1<br>61.5 (F<70) /62.5<br>75.9(M>70) /74.4<br>73.6(F>70) /77.6
Hess (2009)	HF	To examine the association between cg. literacy and medication administration	Most caregivers had adequate health literacy (80%).<br>No conclusions regarding this medication administration as inadequate number of caregivers recruited	QUANT (Correlational)	USA	Cross Sectional<br>Convenience	5 (F)<br>(N/S)	65/72.8
Hooley (2005)	HF<br>(NYHA III or IV)	To explore if greater cg burden is associated with increasing disease burden and depressive symptoms in pts and cg	Cgs experience high cg burden and depressive symptoms which are related to pt QOL disease burden and cg burden<br>Medication and six month outcomes are associated with these factors	QUANT	CANADA	Cohort Study<br>Convenience	50<br>(40F, 10M)<br>(N/S)	61/72

For peer review only - http://bmjopen.bmj.com/site/about/guidelines.xhtml

Summary of included studies

First Author/Year	Diagnosis	Aims	Key Conclusions	Methods	Country	Data Collection Sampling	Participants Caregiver (Time caring)	Mean Age (Caregiver /Recipient)
Hwang (2011)	HF (NYHA I – IV)	To identify factors associated with the impact of caregiving	There is a need for interventions to increases cg's sense of control and social support	QUANT	USA	Cross Sectional Convenience	76 Dyads (54F, 22M) (Mean = 53.4 months)	53.4/53.8
Hynes (2012)	COPD	To explore the experiences of cg's providing care to a family member with COPD	Cgs experience of illness burden included symptom, cultural and lifeworld meanings. Relationships between formal health care and healthcare professionals were difficult	QUAL (Phenomenological)	IRELAND	Interviews Convenience	11(9F, 2M) (1 – 15 years)	N/S/N/S
Imes (2011)	HF (NYHA III-IV)	To describe cg's experience of living with HF	The severity of the disease limited the partners lifestyle Partners were unprepared for cg role	QUAL (Descriptive)	USA	Interviews Convenience	14(11F, 3M) (N/S)	64.8/68
Karmilovich (1994)	HF (NYHA III OR IV)	To examine cg demands and components of caring. Assess stress and correlation with cg burden .Assess relationship of demographic variables to cg burden and stress	The most significant burden experienced may relate to the emotional component. There is a need for education about the disease Women reported more burden than men	QUANT (Correlational)	USA	Descriptive survey Purposive	41 (30F, 11M) (N/S)	56.7/N/S
Kitko (2010)	HF	To gain a deeper understanding of the type of work in spousal caregiving	Cg' require more than a brief contact with healthcare providers The needs of the cg escalate in tandem with pt	QUAL	USA	Interviews Convenience	20(14F, 6M) (2 months – 9 years)	67/70

For peer review only - http://bmjopen.bmj.com/site/about/guidelines.xhtml

Summary of included studies

First Author/Year	Diagnosis	Aims	Key Conclusions	Methods	Country	Data Collection Sampling	Participants Caregiver (Time caring)	Mean Age (Caregiver /Recipient)
Kneeshaw (1999)	CAD	To examine cg mutuality and preparedness for caring post CABG surgery. To examine if these cg factors influence recovery outcomes of pts	Mutuality Scale (MS) and Preparedness for Caregiving (PCS) at discharge indicated positive feelings to role and preparedness and positive correlation with recovery At 3 months indicators of stress present in cg-pt relationship	QUANT	USA	Longitudinal Convenience	49 (32F, 17M) (N/S)	50.1/72.6
Liljeroos (2014)	HF	To understand perceived caring needs in dyads and understand areas of. support for cgs	Two themes; dyads need continuous guidance Dyads need to share burden and support with each other and others	QUAL	SWEDEN	Focus Groups Convenience	19Dyads (N/S)	70/72 (7F, 12M)
Lindqvist (2013)	COPD (Mild to severe)	To describe conceptions of daily life for women caring for men	Four themes: unchanged life socially restricted life, changed roles changes in health changes in the relationship and support is needed	QUAL (Phenomenological)	SWEDEN	Interviews Purposive	21(F) (N/S)	72/N/S
Loftus (2004)	HF (NYHA II-IV)	Investigate outcomes of caregiving in late stage HF	Caregiving is an interpersonal dynamic process dependent on interpersonal style of cg and pt.	QUANT (Correlational)	UK	Longitudinal Convenience	53 (41F, 12M) (6.66 hours per day)	66.7/76.3

For peer review only - http://bmjopen.bmj.com/site/about/guidelines.xhtml

Summary of included studies

First Author/Year	Diagnosis	Aims	Key Conclusions	Methods	Country	Data Collection Sampling	Participants Caregiver (Time caring)	Mean Age (Caregiver /Recipient)
Lum (2014)	HF (NYHA II-IV)	Measure of relationship quality and cg benefit, burden and depressive symptoms	Higher relationship quality was associated with less caregiver burden and more caregiver benefit	QUANT (Correlational)	USA	Cross Sectional Purposive	19 (7F, 12M) (<1 - >8 hours per week)	59/69
Luttik (2007)	HF	Experience and needs of cgs wellbeing factors	Several life changes occur; changes in life changes in relationship, coping and support	QUAL	NETHERLANDS	Interviews Convenience	13(10F, 3M) (N/S)	66/88.6
Luttik (2009)	HF (NYHA II-IV)	Investigate QOL in cg's of HF pts versus QOL in people with healthy partners	Performing cg tasks was negatively associated with QOL of female cgs	QUANT	NETHERLANDS	Cross Sectional Purposive	303 (N/S)	67/69
Marcuccilli (2011)	HF –LVAD in situ	Explore life adjustments of cg's caring for long term LVAD pts.	Burden of caring can be overwhelming but dissipated over time Support is vital for cgs in coping and adjusting to cg role	QUAL (Phenomenological)	USA	Interviews Convenience	5 (5F) (N/S)	56.6/N/S
Marcuccilli (2014)	HF-LVAD as DT	Explore experience of caring for family member with an LVAD-DT	Fear of death and hope for life. LVADs restored hope	QUAL (Phenomenological)	USA	Interviews Purposive	7(6F, 1M) (18 – 24 hours per day)	65/N/S

For peer review only

For peer review only - http://bmjopen.bmj.com/site/about/guidelines.xhtml

Summary of included studies

First Author/Year	Diagnosis	Aims	Key Conclusions	Methods	Country	Data Collection Sampling	Participants Caregiver (Time caring)	Mean Age (Caregiver /Recipient)
Miravitlles (2015)	COPD	Analyse burden of cg's	Higher burden among cg's caring for pts with greater dependency	QUANT	SPAIN	Survey Representative	22,795 (Mean = 12.7 hours daily, severe COPD)	56.5/72
Nakken (2017)	COPD	Investigate differences in male and female cg's and their perception of pts. symptoms	Female partners had more anxiety male and female<br><br>Coping styles differed between genders. Female cgs viewed male pts as less dependent	QUANT (Correlational)	NETHERLANDS	Cross Sectional Convenience	188 Dyads (103F, 85M) (N/S)	65.4/63.3F 65.1/68.7M
Näsström (2017)	HF	Cg's participation & perspective of home care services	Cg's were satisfied with most aspects of participation, information and contact	MIXED METHOD	SWEDEN	Interviews Purposive	15 (11F, 4M) (N/S)	77/N/S
Park (2013)	CAD	Difficulty and demands of cg tasks for older cg's of CABG pt.'s	Cg's reported more demands than difficulties Difficult tasks were household, transportation, managing behaviour problems and tasks outside the home	QUANT	USA	Cross Sectional Convenience	35(29F, 6M) (Mean = 19days)	60/N/S
Pattenden (2007)	HF	Explore how pts and cgs cope with daily life with HF	Significant psychological and physical health impact for cgs	QUAL	UK	Interviews Purposive	20(18F, 2M) (N/S)	67.8/N/S

For peer review only - http://bmjopen.bmj.com/site/about/guidelines.xhtml

Summary of included studies

First Author/Year	Diagnosis	Aims	Key Conclusions	Methods	Country	Data Collection Sampling	Participants Caregiver (Time caring)	Mean Age (Caregiver /Recipient)
Pi-Ming Yeh (2012)	HF	Explore burden on family cg's of older HF pts	Pt ADL, support quality of relationship continuity of care, coping and spiritual wellbeing all predicted cg burden	QUANT (Correlational)	USA	Cross Sectional Purposive	50 (35F, 15M) (< 6 months - > 1 year)	60.3/77.6
Pressler (2013)	HF (NYHA I-IV)	Examine changes in cg. burden and HRQOL Determine different perceptions between cg's of low HF pts and Estimate time spent on cg tasks	Cg's of pts with more HF symptoms had poorer perception of cg experience cgs of high HF pts Cgs had fewer depressive symptoms over time	QUANT (Repeated Measures)	USA	Longitudinal Convenience	65(48F, 17M) (Mean = 9.3 years)	59.7/69
Riegner (1996)	COPD	To understand QOL and its Association with role strain, humour and support in cg's and pt.'s.	Low role strain and high support equals greater QOL	QUANT (Correlational)	USA	Cross Sectional Convenience	83(Dyads) (50F, 33M) (N/S)	63.2/65.6
Rolley (2010)	CAD	Describe experience of cgs of pt.'s undergoing Percuatenous Coronary Intervention	Findings indicate need to include cg's in care planning and decision making	QUAL	AUSTRALIA	Focus Groups Convenience	18 (F) (N/S)	N/S/N/S
*Saunders (2009) *Saunders (2008)	HF (NYHA I-IV)	To determine indicators of cg HRQOL	Nurses should conduct carer assessments screening for depression and financial impact of caring	QUANT (Correlational)	USA	Cross Sectional Purposive	50 (42 F, 8M) (Mean = 5.9 years)	58.1/77.6

For peer review only - http://bmjopen.bmj.com/site/about/guidelines.xhtml

Summary of included studies

First Author/Year	Diagnosis	Aims	Key Conclusions	Methods	Country	Data Collection Sampling	Participants Caregiver (Time caring)	Mean Age (Caregiver /Recipient)
Saunders (2010)	HF	Compare employed and unemployed cg.'s on depression and wellbeing	Employed cg's were likely to be sharing the role Employed cg's need to be supported This study supports the dual role of caregiving and employment	QUANT	USA	Cross Sectional Convenience	41 (37F, 4M) (2.9 – 6 years)	59(Unemp.)/78 52(Emp.)/77
Schwarz (2003)	HF	Evaluate support on stress for cg's.	Social support did not moderate effect of stress on depression	QUANT	USA	Cross Sectional Convenience	75 (55F, 20M) (Mean = 6 years)	63/N/S
Scott (2000)	HF	HRQOL of cg's and pts receiving community-based inotropic infusions	Cg esteem and perceived control affected pt HRQOL	QUANT	USA	Cross Sectional Purposive	18 (16F, 2M)	63/69.3
Spence (2008)	COPD (Adv.)	Needs and experiences of family cg's	Cgs reported restricted ADLs and emotional distress	QUAL (Descriptive)	N.IRELAND	Interviews Purposive	7 (6F, 1M) (1 – 4 years)	N/S/N/S
Strom (2015)	HF	Next of kin's experience and responsibilities when caring	Two themes: "being on the alert" "forced volunteer" indicate caring is a challenging experience	QUAL	NORWAY	Interviews Convenience	19 (17F, 2M) (N/S)	Median 63/NS
Takata (2008)	COPD (Long term O2 Therapy)	Explore cg burden	High burdened cg's spend more time on physical tasks and unlikely to be able to go out 8 hours or more per day	QUANT	JAPAN	Cross Sectional Convenience	45 Dyads (37F, 8M) (N/S)	68/75.2

For peer review only - http://bmjopen.bmj.com/site/about/guidelines.xhtml

For peer review only

Summary of included studies

First Author/Year	Diagnosis	Aims	Key Conclusions	Methods	Country	Data Collection Sampling	Participants Caregiver (Time caring)	Mean Age (Caregiver /Recipient)
Vellone (2015)	HF (NYHA I-IV)	Examine cg self-efficacy and contribution to pt self-care	Self-efficacy building activities are needed for cgs.	QUANT (Correlation)	ITALY	Cross Sectional Convenience	515 Dyads (270F, 245M)	56.6/75.6
Wallin (2013)	CAD	To describe cg's need for support and information and impact after a cardiac event	Everyday life was still affected 6 months after the cardiac event	QUAL (Descriptive)	SWEDEN	Interviews Purposive	20 (14F, 6M) (N/S)	55/N/S
Woolfe (2007)	COPD	Identify needs of cg's and how this impacts cg wellbeing	Information is the most important need for cg's. Cg need scores on Mean Barrier Needs indicate cg role is stressful	QUANT (Descriptive)	AUSTRALIA	Cross Sectional Convenience	63(39F, 24M) (N/S)	N/S/N/S
Wingham (2015)	HF	Identify needs of cg's to inform development of a caregiver resource for use in a home- based self-management intervention	If cg needs are met this study indicates cg's can successfully support self-management	QUAL	UK	Interviews (I) Focus Groups (FG) Purposive	22 (16F, 6M) (6 months – 8 years)	(I)67/N/S (FG)62 /N/S

For peer review only - http://bmjopen.bmj.com/site/about/guidelines.xhtml

Study characteristics

Of the 54 studies, 21 were qualitative, 32 quantitative and 1 mixed methods. 34 focused on HF, 14 COPD and 6 CAD. The total number of caregiver participants was 26,453. Caregivers were primarily female (63%), with a median age of 62 years. Patient median age was 69. A summary of study characteristics is provided in Table 3.

For peer review only

For peer review only - http://bmjopen.bmj.com/site/about/guidelines.xhtml

Table 3

Study Characteristics

Summary of Study Characteristics	n = 54 Studies	
**Aetiology of patients**		
CAD n (%)	6 (11)	
HF n (%)	34 (63)	
COPD n (%)	14 (26)	
**Caregiver Participants***		**Patients ***
Median age (range)	62 (43-77)	69 (36-93)
Median % of female (range)	63% (5–270)	38% (1-229)
**Relationship between patient and caregiver (n = 26,008 caregivers)**		
Spousal/Partner n (%)	2,321 (9%)	
Son/Daughter n (%)	610 (2%)	
Sibling n (%)	22 (0.07%)	
Parent n (%)	10 (0.03%)	
Friend/Relative n (%)	228 (0.9%)	
Not stated	22,961 (88%)	
**Type of study**		
Qualitative n, (%)	21 (39)	
Quantitative n, (%)	32 (59)	
Mixed n, (%)	1 (2)	
**Study Design**		
Cross-Sectional n, (%)	24 (44)	
Longitudinal n, (%)	4 (7)	
Cohort n, (%)	2 (4)	
Quant. (survey) n, (%)	2 (4)	
Qual. (Interview/ Focus Group) n, (%)	16 (31)	
Phenomenological n, (%)	5 (8)	
Inductive n, (%)	1 (2)	
**Continents of publication**		
Europe n, (%)	22 (41)	
North America n, (%)	29 (54)	
Australasia n, (%)	3 (5)	
**Date of publication**	**n= 57 Publications●**	

For peer review only - http://bmjopen.bmj.com/site/about/guidelines.xhtml

1990-1995	2
1996-2001	3
2002-2007	10
2008-2013	22
2014-2017	20

For peer review only

For peer review only - http://bmjopen.bmj.com/site/about/guidelines.xhtml

Quality assessment

Studies of insufficient quality were excluded, qualitative papers were appraised and only high quality qualitative studies were included.[29] All 21 qualitative studies were classified as high quality. Quantitative studies were quality appraised classified as follows; 3 high quality, 12 medium quality and 17 low quality. See Table 4(a) and 4(b) for quality appraisal tables.

For peer review only

For peer review only - http://bmjopen.bmj.com/site/about/guidelines.xhtml

Table 4(a)

Quality Appraisal – Qualitative Papers

	Design	Recruitment	Data Collection	Data Analysis	Findings	Total
Andersen (2015)	1	1	1	0	1	**4 [H]**
Baker (2010)	1	0	1	1	1	**4 [H]**
Bove (2016)	1	1	1	1	1	**5 [H]**
Burke (2014)	1	1	1	1	1	**5 [H]**
Clark (2008)	1	1	1	1	0	**4 [H]**
Figueiredo (2015)	0	1	1	1	1	**4 [H]**
•Halm (2016)	1	1	1	1	1	5 [H]
•Halm (2017)	1	1	1	1	1	**5 [H]**
Hynes (2012)	1	1	1	0	1	**4 [H]**
Imes (2011)	1	1	1	1	1	**5 [H]**
Kitko (2010)	1	1	1	1	1	**5 [H]**
Liljeroos (2014)	1	1	1	1	1	**5 [H]**
Lindqvist (2013)	1	1	1	1	1	**5 [H]**
Luttik (2007)	1	1	1	1	1	**5 [H]**
Marcuccilli (2011)	1	0	1	1	1	**4 [H]**
Marcuccilli (2014)	1	1	1	1	1	**5 [H]**
*Näsström (2017)	1	1	1	1	1	**5 [H]**
Pattenden (2007)	1	1	1	1	0	**4 [H]**
Rolley (2010)	1	1	1	1	0	**4 [H]**
Spence (2008)	1	0	1	1	1	**4 [H]**
Strom (2015)	1	1	1	1	1	**5 [H]**
Wallin (2013)	1	0	1	1	1	**4 [H]**
Wingham (2015)	1	1	1	1	1	**5 [H]**

For peer review only - http://bmjopen.bmj.com/site/about/guidelines.xhtml

Table 4(b)

Quality Appraisal – Quantitative Papers

First Author	Study design	Participant sampling	Participant attrition	Outcome Measures	Data Analysis	Overall score
Ågren (2010)	CS	Purp [+1]	0% [+1]	Non-V	MV [+1}	**3 [M]**
Al-Rawashdeh (2017)	CS	Purp [+1]	NS	V [+1]	MV [+1]	**3 [M]**
Badr (2017)	CS	Con	15.5% [+1]	Non-V	MV [+1]	**2 [L]**
Bakas (2006)	CS	Con	NS	V [+1]	MV [+1]	**2 [L]**
Chung (2016)	CS	Con	NS	V [+1]	UV	**1 [L]**
Cossette (1993)	CS	Con	NS	V [+1]	MV [+1]	**2 [L]**
Evangelista (2002)	CS	Con	20% [+1]	V [+1]	MV [+1]	**3 [M]**
Figueiredo (2013)	CS	Con	17% [+1]	V [+1]	MV [+1]	**3 [M]**
Figueiredo (2014)	CS	Con	11% [+1]	Non-V	MV [+1]	**2 [L]**
Grigorovich (2017)	LS [+1]	Con	NS	V [+1]	MV [+1]	**3 [M]**
• Halm (2006)	CS	Con	64%	V [+1]	MV [+1]	**2 [L]**
• Halm (2007)	CS	Con	64%	V [+1]	MV [+1]	**2 [L]**
Hess (2009)	CS	Con	NS	V [+1]	MV [+1]	**2 [L]**
Hooley (2005)	CS	Con	0% [+1]	V [+1]	UV	**2 [L]**
Hwang (2011)	CS	Con	35%	V [+1]	MV [+1]	**2 [L]**
Karmilovich (1994)	CS	Purp [+1]	24%	V [+1]	MV [+1]	**3 [M]**
Kneeshaw (1999)	LS [+1]	Con	32.7%	V [+1]	MV [+1]	**3 [M]**
Loftus (2004)	LS [+1]	Con	36%	V [+1]	MV [+1]	**3 [M]**
Lum (2014)	CS	Purp [+1]	5% [+1]	V [+1]	MV [+1]	**4 [H]**
Luttik (2009)	CS	Purp [+1]	31%	Non-V	MV [+1]	**3 [M]**
Miravitlles (2015)	CS	Rand [+1]	0% [+1]	Non-V	MV [+1]	**3 [M]**
Nakken (2017)	CS	Con	58%	Non-V	MV [+1]	**1 [L]**
*Näsström (2017)	LS [+1]	Purp [+1]	7% [+1]	V [+1]	MV [+1]	**5 [H]**
Park (2013)	CS	Con	NS	V [+1]	UV	**1 [L]**

For peer review only - http://bmjopen.bmj.com/site/about/guidelines.xhtml

Pi-Ming Yeh (2012)	CS	Purp [+1]	39%	V [+1]	MV [+1]	**4 [H]**
Pressler (2013)	LS [+1]	Con	16% [+1]	V [+1]	MV [+1]	**4 [H]**
Riegner (1996)	CS	Con	71.8%	V [+1]	MV [+1]	**2 [L]**
• Saunders (2008)	CS	Purp [+1]	36%	V [+1]	MV [+1]	**3 [M]**
• Saunders (2009)	CS	Purp [+1]	36%	V [+1]	MV [+1]	**3 [M]**
Saunders (2010)	CS	Con	NS	V [+1]	UV	**1 [L]**
Schwarz (2003)	CS	Con	NS	V [+1]	MV [+1]	**2 [L]**
Scott (2000)	CS	Purp [+1]	10% [+1]	Non-V	MV [+1]	**3 [M]**
Takata (2008)	CS	Con	NS	V [+1]	UV	**1 [L]**
Vellone (2015)	CS	Con	NS	V [+1]	MV [+1]	**2 [L]**
Woolfe (2007)	CS	Con	37%	V [+1]	UV	**1 [L]**

For peer review only

For peer review only - http://bmjopen.bmj.com/site/about/guidelines.xhtml

Findings

Six concepts relating to caregiver experience were identified, (1) mental health, (2) caregiver role, (3) lifestyle change, (4) support for caregivers, (5) knowledge and (6) relationships. Four additional concepts were identified from qualitative papers only (6) expert by experience, (7) vigilance, (8) time and (9) shared-care (See Figure 2). The concepts are reflected in caregiver quotes in Table 5.

For peer review only

For peer review only - http://bmjopen.bmj.com/site/about/guidelines.xhtml

Figure 2

Conceptual Experiences of Caregivers

For peer review only

For peer review only - http://bmjopen.bmj.com/site/about/guidelines.xhtml

Table 5

Illustrative Quotes of caregiver experience – by concept

**Mental Health**	*"The mental strain is difficult. I feel so trapped"*<br><br>*"“You fall into a huge hole, then the world gets so tiny, it all gets so narrow that it is almost unbearable”*<br><br>*“I feel like sleeping beauty. The hawthorn hedge has closed around me, and I cannot do anything about it.”*
**Role**	*“I can sum my role up in three words, I am a cheerleader, drill sergeant, and negotiator”*
**Lifestyle Change**	*“Our life has come down. The two of us used to go out dancing. We loved dancing and then it all stopped.”*
**Knowledge**	*“I wish I had had more education on the ‘what ifs’. When I was leaving the hospital nobody really said, “OK now this is what’s going to happen and this is what you’re going to have to do.” If there would’ve been any kind of complications I would’ve been totally in the dark. I didn’t know all the things I needed to know."*
**Relationships**	*“I just love him and I find that every day when I see him, what else could I do to try and make him a wee bit … better? It’s very satisfying to know that he appreciates what I do and it’s nice to know that you are helping someone.”*<br><br>*“‘It’s like having another child sometimes because you are sort of responsible and I feel he is my responsibility. I feel that he is not anybody else’s responsibility…”*
**Support**	*“And then I really felt alone in it all. Because everybody would call and come over and ask, how is John? Hardly anyone asked “how are you doing”?*<br><br>*“Doctors (do) not realize that 1 day your life is jut normal and then this comes and smashes everything to bits, you know- and there are so many questions.”*<br><br>*“I would be lost without, our heart failure nurse, and, all the other input we’ve had from all the other professionals, like the podiatrist and GP. . . .. . . You can do it, but in partnership with everybody else.”*

For peer review only - http://bmjopen.bmj.com/site/about/guidelines.xhtml

**Vigilance**	***"Every morning I put my ear to his chest and listen to his heart, that is how we first discovered he was in atrial fibrillation so now I do it every morning before I leave. I monitor him very closely and there are days in which I do not feel comfortable leaving for work so those days I work at home. I call everyday from work and we have our routine, if I am not aware of anything he had planned for the day, I then immediately call my neighbour to check on him"***
**Shared Care**	***"There were days I thought to myself, where are we going from here? But we mastered it together and tried to do things at his pace."***
**Time**	***"At first it was overwhelming. I didn't think I could do it. When they first told me I was like, "I can't do that," you know. And then they explained to me, like, yes you can. It's like getting a new baby. You know, you learn how to take care of them step by step and then it's just part of the routine. And that's really the way it was"***
**Expert by Experience**	***"It's so frustrating when she goes into hospital and the nurses and the doctors say it's her condition, you know. I'm like I'm with her twenty-four hours a day, I know how breathless she is without infection and I know how breathless she is with an infection there's a major difference"***<br><br>***"I see him every day, they are just little subtle changes, they are not showing up in the numbers the doctors are concerned with but I see it"***

For peer review only

1. Mental Health

Twenty-five quantitative, [37-63] twenty qualitative [64-84] and one mixed method[85] study addressed mental health. This encompassed depression and burden. Caregivers described an internal and external conflict of emotions, recognising a psychological change within themselves and the care recipient. Maintaining hope and positivity, versus managing worries, fears and anxieties was predominant.[64-83] Pressler's (2013) HF study; identified caregivers had moderately poor health at baseline and at 8 months but they had fewer depressive symptoms over time.[55] Burden arose due to greater responsibilities.[66, 69,74,82,83] Pi-Ming Yeh (2012), noted the quality of relationship and lack of family support significantly predicted greater family caregiver burden.[54] Näsström (2017) reported, caregiver burden was concerned with the future and their fears of potential demands.[85] Those with greater resiliency appeared to adjust and cope better with the illness trajectory.[65, 66, 77, 78] Caregivers

For peer review only - http://bmjopen.bmj.com/site/about/guidelines.xhtml

described mental adjustment after an acute event.[78] Living through an acute event was long lasting and some experienced post traumatic symptoms.[81]

2. Caregiver Role

This is addressed in eighteen qualitative [65-73,75,76,78-84,86] and fourteen quantitative studies.[40-42, 50-52, 55, 58, 60, 61, 87, 88, 89,90] Caregiver role is complex and requires much co-ordination. [75, 82, 84] Caregivers describe significant role change such as increasing domestic tasks. [64, 67, 70, 72,77 -80, 83] Role loss is prevalent [65, 66, 71] and caregivers need to reframe their identity.[73, 81] Societal expectation regarding the relationship and gender, influences caregivers adjusting to their roles.[66, 68, 69, 75, 80] Caring can be positive and rewarding. Caregivers learn about themselves and strengths they have. [66, 76-78, 81, 82, 84] Pressler (2013), describes the tasks involved; domestic, emotional support, managing dietary needs and transport.[55] Pressler also reports caregivers of persons with greater HF symptoms experience more difficulty with their role.[55]

3. Lifestyle Changes

Fourteen quantitative [38-41, 44, 46, 53,55, 60, 61, 63, 88,89, 91] and twenty-one qualitative[64-84,86] studies addressed lifestyle changes. Caregivers experienced leisure, social and work related problems. [40,41,91] Caring interrupted and eliminated tasks from their routine.[40, 41, 60] Contrastingly, Pressler (2013) reported caregivers perceptions of how their lives changed as a result of caregiving was neutral and improved from baseline to 4 and 8 months.[55] Caregivers became adaptable in their new role.[73,81,86] There was less personal time for leisure and hobbies either alone or with the care recipient[68-71,77,78,83,84] Caregivers described daily "ups & downs" and had to adjust their routines dependent on the presentation of the care recipient. [64,65,67,72,74,76,79,80,82,84]

4. Support for caregivers

Fifteen quantitative[42, 46-48, 50, 52-54,56,57,59-61, 63,88,90], twenty-one qualitative[64-84,86] and one mixed method[85] study examine support. This includes healthcare, family and social support. The weight of perceived external expectations, the necessity of being pro-active in obtaining support and maintaining a social role was described across all diagnoses.[41,46,47,49-51, 54-58, 62, 90] Pi-Ming Yeh (2012) identified lack of family support as a significant issue.[54] Caregivers felt abandoned by healthcare teams. After hospital discharge they had to provide care without advice or medical support.[67, 73, 79] Positive interactions were reported. Namely access to healthcare professionals via telephone or home support.[64, 65, 78, 85]

For peer review only - http://bmjopen.bmj.com/site/about/guidelines.xhtml

5. Knowledge

This was addressed in seventeen qualitative[64,66-76,78,80,81,83,84,86] five quantitative[51,61-63,88] and one mixed method[85] study. This describes caregivers understanding of the diagnosis and need for knowledge throughout the duration of illness. [64,68,71,76,84,85,86] Caregivers report information from health professionals was often inadequate. [72, 74, 75] Timing and format of information was significant. Caregivers received information verbally or by leaflets in hospital but describe being left alone to provide care in the long term [66, 69,70,80,83] Caregivers had difficulty understanding how to navigate the care system. [73, 81] They had to make decisions without full knowledge of the consequences of their decision making, particularly during acute exacerbations.[66] The quantitative element of Näsström's (2017) mixed methods study correlated with qualitative studies; receipt of sufficient information was central to managing HF and was associated with better perceived health of caregivers.[85]

6. Relationships

Twenty qualitative,[65-84,86] twenty-two quantitative[37-39, 43-45,47-53,55-58,62,88,91,92,93] and one mixed method[85] study examined relationships. In HF studies caring for individuals with more symptoms resulted in poorer perceived experiences.[55,92] Higher relationship quality resulted in less burden and more benefit from the relationship. The relationship prior to diagnosis influenced the current relationship. Perspective of the relationship was either a sense of duty [66,75,81,82,85] or this was a valuable second chance.[67,76,83,84] Caregivers reported difficulty communicating about the condition leading to isolation, stress and conflict between caregiver and care recipient.[72,74] The relationship requires negotiation.[70,86] Caregivers prioritised the care recipient over their own needs. [65, 73,75,78,83]

7. Expert by Experience

Twelve qualitative studies[66-71,73,76,77,81,82,84,86] addressed this concept. Caregivers learnt new skills. They became "experts by experience" discovering through "doing" and observing health professionals.[67,69,84] They developed "proto-professional skills"; in medication administration [66,81,86] judging care recipients level of functioning [80] and decision making in times of exacerbations.[71] Caregivers observed the nuances of change in the care recipient often not perceived by healthcare teams or other family members such as skin colour or irritability.[73,76,82]

8. Vigilance

Vigilance was recurring in caregivers' narrative across all diagnoses and was present in nineteen qualitative studies.[65-81,82,84,86] Caregivers were always on the alert observing the care recipient.

For peer review only - http://bmjopen.bmj.com/site/about/guidelines.xhtml

[67,68,71,73-75,78-80,82] They lay awake at night listening for their partners' breath.[70,72,76,86] This impacted on caregivers' health creating constant fatigue, worry and stress.[66,81] Caregivers recognised that the need for vigilance came from themselves and their insecurities. [65, 77, 84]

9. Time

Time explores how caregivers adjusted to living with the illness and was present in fifteen qualitative studies.[66,68-78,81,83,84,86] Caregivers adapted to a new life, referring to "then", how life was and "now" their current life.[70, 71,76,77,84] The duration of caregiving and severity of illness influenced caregiver's ability to adjust.[67, 74, 77] Caregivers lived day by day [84] and viewed the future, with hope or uncertainty about what lay ahead. [66, 71,73,80,83]

10. Shared Care

Shared care was present in sixteen qualitative studies.[64-67,69-77,81,82,84,86] This demonstrates caregiver and care recipient working together managing the illness. Jointly administering medication [69, 82] and attending appointments.[74] The presence of illness was a process they adjusted to together. [77, 81] Caregivers referred to themselves and the care recipient as "we", when discussing dealing with the illness.[64,72,76] The mutual perspective between caregiver and care recipient served to isolate them from the world, the illness was *"taking a life of its own…it's like this third person"*, (Hynes, 2012, p. 1071)

There were differences in caregiver experience for each of the diagnoses and these are discussed below.

Heart Failure

HF caregivers experienced an "ebb and flow" in caring, an underlying worry, fear and anxiety which at times of change or illness heightened. Lifestyle changes were long lasting and sustained. Obtaining knowledge was necessary throughout all stages of the illness. Sourcing information and communication with health professionals was often difficult. In spousal relationships they predominantly viewed the care recipient as another child or as a "duty."

Chronic Obstructive Pulmonary Disease

COPD caregivers experienced a prolonged impact on their mental health similar to HF caregivers. Severity of illness was influential on their experience of burden. Role change was long lasting and profound for many. They expressed concerns with perceived lack of knowledge. During

For peer review only - http://bmjopen.bmj.com/site/about/guidelines.xhtml

exacerbations COPD caregivers experienced anxiety and fear of their loved one dying. COPD caregivers highlighted the loss of social roles while trying to maintain their loved ones dignity. The coughing and spitting associated with COPD often left the care recipient embarrassed. The caregiver tried to avoid situations where this would happen. The dynamics of spousal relationships changed, caregivers described losing the intimate love they had for their partner.

Coronary Artery Disease

Caregivers of CAD patients experienced intense role change on discharge from hospital and in the acute phase of illness. They initially engaged with a high volume of tasks which reduced over time. CAD caregivers experienced post-traumatic type symptoms if they witnessed the recipient experience an acute event. Caregivers described being unable to talk about this and reliving the event in their heads. Anxiety did ease over time for many. Caregivers felt unprepared at hospital discharge and highlighted not realising how much their routine would be disrupted. Caregivers reported viewing the experience as a second chance and had a renewed sense of love and appreciation for the relationship.

DISCUSSION

This mixed methods systematic review demonstrates the similarities and differences in caregiver experiences across three common cardio-respiratory conditions. It highlighted the differences in experiences obtained from qualitative and quantitative research. Commonly occurring experiences included the exacerbation of caregiver physical and mental health due to the role. This correlates with Kang et al.'s (2011) systematic review of HF caregivers identifying that caregiving resulted in a multitude of changes in caregiver's lives regardless of age, gender and ethnicity.[94] Addressing both patient and caregiver needs in order to maintain wellbeing for both is important[19] and recognises the value of "shared-care" between patient and caregiver. The prevalence of mental health needs in this review demonstrates the need for psychosocial support for caregivers. This concurs with AasbØ et al. (2016), identifying caregivers need to be in "emotional control"[95] and Wingham et al. (2017) describing the "enduring anguish" experienced by caregivers.[96] Lawton et al. (1991) attribute caregiver well-being to the commitment of the caregiver to the role and dealing with competing demands which can increase burden and negatively impact affect. Spousal caregivers may be more ready to accept the role of caregiving than adult children who may view it as an imposition on an already established lifestyle.[97] In this review societal expectations had an impact on how caregivers adjust to their role. Additionally, the quality of the relationship prior to becoming a caregiver had an influence on the caregiver subjective experience of burden.

For peer review only - http://bmjopen.bmj.com/site/about/guidelines.xhtml

Caregivers had predominantly negative experiences of support and described uncertainty of how to obtain this. Caregivers' need greater support and knowledge transfer to conduct their role.[98] They should be included in clinical appointments[99] to ensure they are not isolated in providing care and to allow for knowledge exchange. Caregivers in Giacomini et al.'s (2012) review of living and dying with COPD reported increasing isolation in addition to their own health issues.[18] They described pressure balancing their variety of roles; similar experiences to the caregivers in this synthesis across all diagnoses. Caregivers emphasised their need to be vigilant. This falls into five categories as defined by Feeney Mahoney's (2003) study of Alzheimer's caregivers; "watchful supervision", "protective intervening", "anticipating", "on duty" and "being there". Caregivers in this synthesis described overt vigilance, putting one's head on the chest of the recipient to check breathing or covert vigilance; observing them throughout the day.[100] Healthcare professionals must be aware of these levels of vigilance and the constant presence of them to support caregivers in their role.

Caregivers are valuable providers of care. Caregiver's needs should be assessed systematically and in a formalised manner in healthcare settings.[16] When developing collaborative models of care the inclusion of caregivers is imperative.[101]

Strengths and limitations

This review demonstrates the complexity of what it means to be a caregiver and should inform clinical care development of interventions. A mixed methods review can be contentious[102] due to the synthesis of differing paradigms. In this review it required transformation of quantitative data into qualitative data.[15,103] We aimed to present a convergence of caregiver experiences by conducting a mixed methods synthesis. However, it demonstrated four differing concepts between the two paradigms. This highlights the challenge of synthesising multiple methods. It is worth exploring how the four additional qualitative concepts could be captured quantitatively in order to inform healthcare intervention. This mixed methods synthesis is, to our knowledge the first to combine caregiver experiences in HF, COPD or CAD. It examines the differences and similarities in experiences, establishing a comprehensive assessment of the knowledge base of caregiver experiences in common cardiorespiratory conditions.

There are limitations to this review; at study level qualitative and quantitative studies were primarily cross-sectional in design, therefore considering caregiver experience only at a single point of time. Only high quality qualitative studies were retained for synthesis. Limitations at systematic review level included English language studies from high income countries and excluding caregivers of

For peer review only - http://bmjopen.bmj.com/site/about/guidelines.xhtml

nursing home residents. Our findings may have limited applicability to other settings. Converting quantitative data into a qualitative data set risks the quantitative data set being oversimplified. This was managed with regular research team meetings to review each stage of this process. Finally, qualitative synthesis is an interpretation of other researcher's interpretations. Again, to minimise individual interpretative bias a second researcher was utilised to seek confirmation of the results.

Implications for practice and future research

There are a number implications following this review. It has demonstrated there are similarities and differences in the caregiver experience in HF, COPD or CAD. The impact on caregiver's lives of those with HF and COPD appears longer lasting and more turbulent than caring for CAD patients. CAD caregiver's experience of hospital during exacerbations increased distress at discharge. This review reflects the complexity of the caregiver's role. The mixed method approach indicted differences in what is being investigated. This is important in demonstrating an understanding of the caregiver experience when dealing with complex conditions. Future research should focus on the involvement of caregivers in the design and delivery of interventions for patients with cardiopulmonary disease. In addition to targeting patients, interventions need to consider caregivers. Best practice interventions for CAD caregivers in the discharge process from hospital to home must be formalised. Further research should also explore mental health in COPD and CAD caregivers.

Consideration needs to be given as to whether quantitative research tools to explore caregiver expertise, view of the future, experience of shared care and vigilance can be developed to capture these caregiver experiences. Longitudinal studies with repeated assessment need to be conducted to assess how stable are caregiver experiences or whether they are liable to much change over time. In this review only four out of thirty-two quantitative studies examined caregiver's experiences longitudinally. Qualitative research demonstrates the nuanced expertise of the caregiving experience, it is important for researchers to consider how to reflect this in quantitative investigation. The quality of quantitative studies in COPD and CAD were medium or low. There is a need for more empirically robust studies examining the experiences of these caregivers.

CONCLUSIONS

This mixed methods systematic review provides a holistic synthesis of caregiver experiences of people with HF, COPD or CAD. It demonstrates there are a number of implications when an individual becomes a caregiver for those with chronic cardiopulmonary disease. Caregivers re-frame their identity and change their life course. Caregivers learn a multitude of skills and develop

For peer review only - http://bmjopen.bmj.com/site/about/guidelines.xhtml

expertise in their new role. Their expertise is invaluable and should be acknowledged in healthcare interventions for these conditions. The quality of evidence was limited by assessment of caregiver experience at single time point. There is need for future studies that employ longitudinal designs examining the change in caregiver experience over time. Caregiving can be positive if caregivers have access to support, are well informed and part of the healthcare team. Understanding the experiences of caregivers for people with these conditions allows healthcare professionals and policy makers to reflect on our approach. Health services must consider caregivers in the design and delivery of interventions.

Contributors: MN, JW, RST: conceived and designed the review and quality appraised the included studies. MN wrote the initial draft of the manuscript. JW AND RST provided feedback and edits to manuscript drafts.

Funding

This study was supported by a University of Exeter Postgraduate Studentship Grant

Competing Interests

None declared

Data Sharing

Any requests for additional information about the study can be accessed via contacting the primary author.

Acknowledgements

We would like to thank the PenCLARHC Evidence Synthesis Team at University of Exeter Medical School**.** Katy Oaks and Catriona Organ, Librarians at Royal Cornwall Hospital Library, Knowledge Spa, Truro. Dr. Ruth Garside, Senior Lecturer in Evidence Synthesis, European Centre for Environment and Human Health for their assistance throughout the process of this review.

References:

1. Carers Trust (2015). Available from: https://carers.org/what-carer (Accessed 26th June 2017).
2. Wolff, J, Spillman, BC, Freedman, VA, Kasper, JD. A national profile of family and unpaid caregivers who assist older adults with health care activities. *JAMA Intern Med*. 2016; 176(3):372-379. Available from doi:10.1001/jamainternmed.2015.7664.
3. International Alliance of Carer Organizations. *Global carer facts*. Available from: http://www.internationalcarers.org/carer-facts/global-carer-stats/(Accessed 1st October 2017).
4. White, C. 2011 Census Analysis: Unpaid care in England and Wales, 2011 and comparison with 2001. *Office of National Statistics.* 2013. Available from: https://www.ons.gov.uk/peoplepopulationandcommunity/healthandsocialcare/healthcaresystem/articles/2011censusanalysisunpaidcareinenglandandwales2011andcomparisonwith2001/2013-02-15 (Accessed 10th October 2017).

For peer review only - http://bmjopen.bmj.com/site/about/guidelines.xhtml

5. Buckner, L, Yeandle, S. Valuing Carers 2015. The rising value of carers' support. *Carers UK 2015.* Available from http://www.carersuk.org/for-professionals/policy/policy-library/valuing-carers-2015 (Accessed 7th July 2017).
6. The Institute of Public Care. *A Carer's Life: Implications and Considerations for Commissioning.* 2017. Available from: http://ipc.brookes.ac.uk/publications/A_carers_life.html (Accessed 30th September 2017).
7. Seltzer, MM, Wailing Li, L. The dynamics of caregiving: transitions during a three-year prospective study. *The Gerontologist.* 2000; 40(2):165–178. Available from doi.org/10.1093/geront/40.2.165.
8. The Care Act. Chapter 23. *The Care Act UK: The Stationery Office.* 2014. Available from: http://www.legislation.gov.uk/ukpga/2014/23/contents.enacted/data.htm (Accessed 11th October 2016).
9. National Institute for Health and Clinical Excellence. *Chronic heart failure in adults: management.* 2010. NICE guideline (CG108).
10. National Institute for Health and Clinical Excellence. *Chronic obstructive pulmonary disease in over 16s: diagnosis and management.* 2010. NICE guideline (CG101).
11. Wilkins, E, Wilson, L, Wickramasinghe K, Bhatnagar, P, Leal, J, Luengo-Fernandez, R, Townsend, N. European Cardiovascular Disease Statistics 2017. *European Heart Network*, Brussels. Available from: http://www.ehnheart.org/images/CVD-statistics-report-August-2017.pdf (Accessed 25th August 2017).
12. Center for Disease Control and Prevention. *Heart Disease Facts and Statistics*. Available from: www.cdc.gov (Accessed 9th November 2016).
13. Raherison, C, Girodet, PO. Epidemiology of COPD. *European Respiratory Review*. 2009;18: 213-221. Available from: doi:10.1183/09059180.00003609.
14. Nicholas Dionne-Odom, J., Hooker, S.A., Bekelman, D. et al. Heart Fail Rev (2017) 22: 543. Available from: https://doi.org/10.1007/s10741-017-9597-4.
15. Foster, F, Piggott, R, Teece, Beech, R. Patients with COPD tell their stories about living with the long-term condition: an innovative and powerful way to impact primary health care professionals' attitudes and behaviour? *Education for Primary Care*, 2016, 27:4, 314-319. Available from doi:10.1080/14739879.2016.1181699.
16. Farquhar, M. Assessing carer needs in chronic obstructive pulmonary disease. *Chronic Respiratory Disesae*. 2017, 1-10. Available from doi:10.1177/1479972317719086.
17. Mansfield, E, Bryant, J, Regan, T, Waller, A, Boyes, A, Sanson-Fisher, R. Burden and unmet needs of caregivers of chronic obstructive pulmonary disease patients: a systematic review of the volume and focus of research output. *Journal of Chronic Obstructive Pulmonary Disease. 2016;* 13(5):662-667. Available from: doi:10.3109/15412555.2016.1151488.
18. Giacomini, M, DeJean, D, Simeonov, D, Smith, A. Experiences of living and dying with COPD: a systematic review and synthesis of the qualitative empirical literature. *Ontario* Health *Technology* Assessment Series. 2012;12(13):1-47.
19. Doherty, L, Fitzsimons, D, McIlfatrick, S. Carers' needs in advanced heart failure: a systematic narrative review. European *Journal* of Cardiovascular Nursing. 2016; 15(4):203-212. Available from: doi: 10.1177/1474515115585237.
20. Stewart, M, Davidson, K, Meade, D, Hirth, A, Makrides, L. Myocardial infarction: survivors' and spouses' stress, coping, and support. *Journal of Advanced Nursing*. 2000; 31: 1351–1360. Available from: doi:10.1046/j.1365-2648.2000.01454.x
21. Stolarik A, Lindsay P, Sherrad H, Woodend K. Determination of the burden of care in families of cardiac surgery patients. Progress in Cardiovascular Nursing. 2000; 15:4–10

For peer review only - http://bmjopen.bmj.com/site/about/guidelines.xhtml

22. Falk, JA, Kadiev, S, Criner, GJ, Scharf, SM, Minai, OA, Diaz, P. Cardiac disease in chronic obstructive pulmonary disease. *Proceedings of the American Thoracic Society*. 2008;5(4): 543-548. Available from: doi:10.1513/pats.200708-142ET.
23. Müllerova, H, Mapel, DW. Cardiovascular comorbidity in COPD systematic literature review. *CHEST.* 2013;144(4):1163–1178. Available from: doi:10.1378/chest.12-2847.
24. Moher, D, Liberati, A, Tetzlaff, J, Altman, DG, The PRISMA Group. Preferred reporting items for systematic reviews and meta-analyses: The PRISMA statement. *PLoS Med.* 2009;6(7). Available from: doi:10.1371/journal.pmed.1000097.
25. Johnson, RB, Onwuegbuzie, AJ, Turner, LA. Toward a definition of mixed methods research. *Journal of Mixed Methods Research,* 2007;1:112–133. Available from: doi:10.1177/1558689806298224.
26. World Bank Data. Available from: http://www.worldbank.org/ (Accessed 10th June 2017).
27. Critical Appraisal Skills Programme. *Qualitative research checklist.* Available from: http://docs.wixstatic.com/ugd/dded87_25658615020e427da194a325e7773d42.pdf (Accessed 10th December 2016).
28. Hannes K. Critical appraisal of qualitative research. In: Noyes J, Booth A, Hannes K, Harden A, Harris J, Lewin S, Lockwood C. (eds.) *Supplementary guidance for inclusion of qualitative research in cochrane systematic reviews of interventions*. Cochrane Collaboration Qualitative Methods Group. Available from: *http://methods.cochrane.org/qi/supplemental-handbook-guidance* (Accessed 12th January 2017).
29. Dixon-Woods, M, Fitzpatrick, R, Roberts, K. Including qualitative research in systematic reviews: opportunities and problems. *Journal of Evaluation in Clinical Practice*. 2001; 7(2):125-133. Available from: doi:10.1046/j.1365-2753.2001.00257.x.
30. Sandelowski, M, Voils, CI, Barroso, J. Defining and Designing Mixed Research Synthesis Studies. *Research in the Schools : A Nationally Refereed Journal Sponsored by the Mid-South Educational Research Association and the University of Alabama.* 2006; 13(1):1-15.
31. Pluye, P, Hong, QN. *Annual Review of Public Health*. 2014. Available from: www.annualreviews.org (Accessed September 27th 2016).
32. Hong, QN, Pluye, P, Bujold, M, Wassef, M. Convergent and sequential synthesis designs: implications for conducting and reporting systematic reviews of qualitative and quantitative evidence. *Systematic Reviews*. 2017; 6(61). Available from: doi:10.1186/s13643-017-0454-2.
33. Tashakkori, A, Teddlie, C. Mixed Methodology: Combining Qualitative and Quantitative Approaches. *Applied Social Research Methods Series*. 1998; 46. SAGE Publications, Thousand Oaks, CA.
34. Noblit, GW. & Hare, RD. Meta-ethnography: synthesizing qualitative studies. 1988. SAGE Publications, Thousand Oaks, CA.
35. Tashakkori, A, Teddlie, C. Foundations of Mixed Methods Research: integrating quantitative and qualitative approaches in the social and behavioural sciences. 2009. SAGE Publications Thousand Oaks, CA.
36. Frantzen, KK, Fetters, MD. Meta-integration for synthesizing data in a systematic mixed studies review: insights from research on autism spectrum disorder. *Qual Quant.* 2015;50:2251–2277. Available from: doi:10.1007/s11135-015-0261-6.
37. Ågren, S, Evangelista, L, Strömberg, A. Do partners of patients with chronic heart failure experience caregiver burden? *European Journal of Cardiovascular Nursing*, 2010;9(4):254–262. Available from: doi:10.1016/j.ejcnurse.2010.03.001.
38. Al-Rawashdeh, SY, Lennie, TA, Chung, ML. The association of sleep disturbances with quality of life in heart failure patient–caregiver dyads. *Western Journal of Nursing Research*, 2017;39(4):492–506. Available from: doi: 10.1177/0193945916672647.

For peer review only - http://bmjopen.bmj.com/site/about/guidelines.xhtml

39. Badr, H, Federman, AD, Wolf, M, Revenson, TA Wisnivesky, JP. Depression in individuals with chronic obstructive pulmonary disease and their informal caregivers. *Aging & Mental Health. 2017;*21(9): 975-982. Available from: doi:10.1080/13607863.2016.1186153.
40. Bakas, T, Pressler, SJ, Johnson, E, Nauser, J, Shaneyfelt, T. Family caregiving in heart failure. *Nursing Research.* 2006;55(3):180-188.
41. Chung, ML, Lennie, TA, Mudd-Martin, G, Dunbar, SB, Pressler, SJ, Moser, DK. Depressive symptoms in patients with heart failure negatively affect family caregiver outcomes and quality of life. *European Journal of Cardiovascular Nursing. 2016;*15 (1):30-38. Available from: doi:10.1177/1474515114535329.
42. Cossette, S, Levesque, L. Caregiving tasks as predictors of mental health of wife caregivers of men with chronic obstructive pulmonary disease. *Research in Nursing & Health*, 1993;16:251-263.
43. Evangelista, l, Dracup, K, Doering, L, Westalke, C, Fonarow, GC Hamilton, M. Emotional well-being of heart failure patients and their caregivers. *Journal of Cardiac Failure*. 2002;8(5): p.300-305. Available from: doi:10.1054/jcaf.2002.128005.
44. Figueiredo, D., Gabriel, R., Jácome, C. & Marques, A. Caring for people with early and advanced chronic obstructive pulmonary disease: how do family carers cope? *Journal of Clinical Nursing.* 2013;23:211–220. Available from: doi: 10.1111/jocn.12363.
45. Figueiredo, D, Gabriel, R, Jácome, C, Cruz, J, Marques, A. Caring for relatives with chronic obstructive pulmonary disease: how does the disease severity impact on family carers? *Aging & Mental Health.* 2014;18(3):385-393. Available from: doi:10.1080/13607863.2013.837146.
46. Grigorovich, A, Lee, A, Ross, H, Woodend, K, Forde, S, Cameron, JI. A longitudinal view of factors that influence the emotional well-being of family caregivers to individuals with heart failure. *Aging & Mental Health*. 2017;21(8):844-850. Available from: doi:10.1080/13607863.2016.1168361.
47. Halm, MA, Jacobson-Treat, D, Lindquist, R. Correlates of caregiver burden after coronary artery bypass surgery. *Nursing Research*. 2006;55(6):426-436.
48. Halm, MA, Bakas, TB. Factors associated with caregiver depressive symptoms, outcomes, and perceived physical health after coronary artery bypass surgery. *Journal of Cardiovascular Nursing*. 2007;22(6):508-515. Available from: doi:10.1097/01.JCN.0000297388.21626.6c.
49. Hooley, PJD, Butler, G, Howlett, JG. The relationship of quality of life, depression, and caregiver burden in outpatients with congestive heart failure. *Congestive Heart Failure.* 2005;11(6):303-310. Available from: doi:10.1111/j.1527-5299.2005.03620.x.
50. Hwang, B, Fleischmann, KE, Howie-Esquivel, J, Stotts, NA, Dracup, K. Caregiving for patients with heart failure: impact on patients' families. *American Journal of Critical Care.* 2011;20(6):431-441. Available from: doi: 10.4037/ajcc2011472.
51. Karmilovich, SE. Burden and stress associated with spousal caregiving for individuals with heart failure. *Progress in Cardiovascular Nursing.* 1994;9(1):33-38.
52. Loftus, L. The influence of illness and psychological factors on patient and caregiver outcomes in heart failure. PhD [dissertation]. Liverpool: University of Liverpool; 2004.
53. Nakken, N, Janssen, DJA, van Vilet, M, de Vries, GJ, Clappers-Gielen, GAL, Michels, AJ, Spruit, MA. Gender differences in partners of patients with COPD and their perceptions about the patients. *International Journal of COPD*. 2017;12: 95–104. Available from: doi:https://doi.org/10.2147/COPD.S118871.
54. Ping-Meh, Y, Bull M. Use of the resiliency model of family stress, adjustment and adaptation in the analysis of family caregiver reaction among families of older people with congestive heart failure. *International Journal of Older People Nursing.* 2012;7:117–126. Available from: doi:10.1111/j.1748-3743.2011.00275.

For peer review only - http://bmjopen.bmj.com/site/about/guidelines.xhtml

55. Pressler, SJ, Gradus-Pizlo, I, Chubinski, SD, Smith, G, Wheeler, S, Sloan, R, Jung, M. Family caregivers of patients with heart failure: a longitudinal study. *Journal of Cardiovascular Nursing.* 2013;28(5):417-428. Available from: doi:10.1097/JCN.0b013e3182563877.
56. Saunders, MM. Factors associated with caregiver burden in heart failure family caregivers. *Western journal of nursing research.* 2008;30(8):943-959. Available from: doi: 10.1177/0193945908319990.
57. Saunders, MM. Indicators of health-related quality of life in heart failure family caregivers. *Journal of Community Health Nursing.* 2009;26:173–182. Available from: doi: 10.1080/07370010903259196.
58. Saunders, MM. Working and caregiving a comparison of employed and unemployed caregivers of older heart failure patients. *Holistic Nursing Practice*. 2010;24(1):16-22. Available from: doi:10.1097/HNP.0b013e3181c8e469.
59. Schwarz, KA, Dunphy, G. An examination of perceived stress in family caregivers of older adults with heart failure. *Experimental Aging Research*. 2003;29(2):221-235. Available from doi:10.1080/03610730303717.
60. Scott, LD. Caregiving and care receiving among a technologically dependent heart failure population. *Advances in Nursing Science.* 2000;23(2):82-97
61. Takata, S, Washio, M, Moriwaki, A, Tsuda, T, Nakayama, H, Iwanaga, T, Inoue, H. Burden among caregivers of patients with chronic obstructive pulmonary disease with long-term oxygen therapy. *International Medical Journal.* 2008;15(1):53-57.
62. Vellone, E, D'Agostino, F, Buck, HG, Fida, R, Spatola, CF, Petruzzo, A, Riegel, B. The key role of caregiver confidence in the caregiver's contribution to self-care in adults with heart failure. *European Journal of Cardiovascular Nursing*. 2015;14(5):372–381. Available from: doi:10.1177/1474515114547649.
63. Woolfe, P, McMillan, M, Conway, J. The needs of caregivers of people with COPD: a study. *Australian Journal of Primary Health.* 2007;13(1):28-35. Available from: doi: 10.2147/COPD.S76091.
64. Andersen, KL, Korneliussen, K, Fagermoen, MS. Family caregivers to a patient with chronic heart failure living at home: «co-workers» in a blurred health care system. *Journal of Cardiovascular Nursing*. 2015;20(3):162-9.
65. Baker, K, Flattery, M, Salyer, J, Haugh, KH, Maltby, M. Caregiving for patients requiring left ventricular assistance device support. *Heart & Lung.* 2010;39(3):196-200. Available from: doi:10.1016/j.hrtlng.2009.07.007.
66. Bove, DG, Zakrisson, AB, Midtgaard, J, Lomborg, K, Overgaard, D. Undefined and unpredictable responsibility: a focus group study of the experiences of informal caregiver spouses of patients with severe COPD. *Journal of Clinical Nursing*. 2016;25:483–493. Available from: doi:10.1111/jocn.13076 483.
67. Burke, RE, Jones, J, Ho, MJ, Bekelman, D. Caregivers' perceived roles in caring for patients with heart failure: what do clinicians need to know? *Journal of Cardiac Failure,* 2014;20 (10):731-738. Available from: doi.org/10.1016/j.cardfail.2014.07.011.
68. Figueiredo, D, Jácome, C, Gabriel, R, Marques, A. Family care in chronic obstructive pulmonary disease: what happens when the carer is a man? *Scandinavian Journal of Caring Sciences.* 2015;30:721-730. Available from: doi: 10.1111/scs.12298.
69. Halm, MA. Specific needs, concerns, strategies and advice of caregivers after coronary artery bypass surgery. *Heart & Lung*. 2016;45:416-422. Available from: doi.org/10.1016/j.hrtlng.2016.07.001.

For peer review only - http://bmjopen.bmj.com/site/about/guidelines.xhtml

70. Halm, MA. Age and gender influences on the needs, concerns and strategies of CABG caregivers. *Heart & Lung*. 2017;46:159-165. Available from: doi.org/10.1016/j.hrtlng.2017.01.012.
71. Hynes, G, Stokes, A, McCarron, M. Informal care-giving in advanced chronic obstructive pulmonary disease: lay knowledge and experience. *Journal of Clinical Nursing*. 2012;21:1068-1077. Available from: doi: 10.1111/j.1365-2702.2011.03944.
72. Imes, C, Dougherty, CM, Pyper, G, Sullivan, MD. Care of Patients with Heart Failure Descriptive study of partners' experiences of living with severe heart failure. *Heart & Lung*. 2011;40:208-216. Available from: doi:10.1016/j.hrtlng.2010.12.007.
73. Kitko, LA. The work of spousal caregiving in heart failure. PhD [dissertation]. State College, PA: Pennsylvania State University;2010.
74. Liljeroos, M, Ågren, S, Jaarsma, T, Strömberg, A. Perceived caring needs in patient-partner dyads affected by heart failure: a qualitative study. *Journal of Clinical Nursing.* 2014;23:2928–2938. Available from: doi: 10.1111/jocn.12588.
75. Lindqvist, G, Albin, B, Heikkila, K Hjelm, K. Conceptions of daily life in women living with a man suffering from chronic obstructive pulmonary disease. Primary Health Care Research & Development. 2012;14:40–51. Available from: doi.org/10.1017/S146342361200031X.
76. Luttik, MLL, Jaarsma, T. Living with heart failure: partner perspectives. *Journal of Cardiovascular Nursing.* 2007;22(2):131-137. Available from: doi:10.1097/00005082-200703000-00010.
77. Marcucilli, L, Casida, J. From insiders' perspectives: adjusting to caregiving for patients with left ventricular assist devices. *Progress in Transplantation.* 2011;21(2):137-43. Available from: doi:10.7182/prtr.21.2.dq53164u1464u683.
78. Marcucilli, L, Casida, J, Bakas, T, Pagani, FD. Family caregivers' inside perspectives: caring for an adult with a left ventricular assist device as a destination therapy. *Progress in Transplantation*. 2014;24(4):332-340. Available from: doi:10.7182/pit2014684.
79. Pattenden, JF, Roberts, H Lewin, HJP. Living with heart failure; patient and carer perspectives. *European Journal of Cardiovascular Nursing.* 2007;6:273-279. Available from: doi:10.1016/j.ejcnurse.2007.01.097.
80. Rolley, J, Smith, J, DiGiacomo, M, Salamonson, Y, Davidson, P. The caregiving role following percutaneous coronary intervention. *Journal of Clinical Nursing*. 2010;20:227–235. Available from: doi: 10.1111/j.1365-2702.2009.03104.
81. Spence, A, Hasson, F, Waldron, M, Kernohan, G, McLaughlin, D, Cochrane, B, Watson, B. Active carers: living with chronic obstructive pulmonary disease. *International Journal of Palliative Nursing*. 2008;14(8):368-372. Available from: doi:10.12968/ijpn.2008.14.8.30771.
82. Ström, A, Andersen, KL, Korneliussen, K, Fagermoen, MS. Being "on the alert" and "a forced volunteer": a qualitative study of the invisible care provided by the next of kin of patients with chronic heart failure. *Journal of Multidisciplinary Healthcare.* 2015;8:271–277. Available from: doi.org/10.2147/JMDH.S82239.
83. Wallin, E, Larsson, IM, Rubertsson, S, Kristoferzon, ML. Relatives' experiences of everyday life six months after hypothermia treatment of a significant other's cardiac arrest. *Journal of Clinical Nursing.* 2013;22:1639–1646. Available from: doi: 10.1111/jocn.12112.
84. Wingham, J, Frost, J, Britten, N, Jolly, K, Greaves, C, Abraham, C, Dalal, H. Needs of caregivers in heart failure management: a qualitative study. *Chronic Illness*. 2015;11(4):304-319. Available from: doi:10.1177/1742395315574765.
85. Näsström, L, Luttik, ML, Idvall, E, Strömberg, A. Exploring partners' perspectives on participation in heart failure home care: a mixed-method design. *Journal of Advanced Nursing.* 2017;73(5):1208–1219. Available from: doi: 10.1111/jan.13216.

For peer review only - http://bmjopen.bmj.com/site/about/guidelines.xhtml

86. Clark, AM, Reid, ME, Morrisson, CE, Capewell, S, Murdoch, DL, McMurray, JJ. The complex nature of informal care in home-based heart failure management. Journal of Advanced Nursing. 2008;61(4):373–383. Available from: doi:10.1111/j.1365-2648.2007.04527.x.
87. Hess, AM. *The health literacy, self-care, and medication hassles of patients with heart failure and their caregivers.* Masters [dissertation]. West Lafayette, IN: Purdue University;2009.
88. Kneeshaw, MF, Considine, RM, Jennings, J. Mutuality and preparedness of family caregivers for elderly women after bypass surgery. Applied Nursing Research. 1999;12(3):128-135.
89. Park, EO, Yates, BC, Schumacher, KL, Meza, J, Kosloski, K, Pullen, C. Caregiving demand and difficulty in older adult spousal caregivers after coronary artery bypass surgery. Geriatric Nursing. 2013;34:383-387. Available from: doi.org/10.1016/j.gerinurse.2013.05.007.
90. Riegner, J. *The relationship of role strain, perceived social support, and humor to quality of life among couples experiencing the life-limiting illness of chronic obstructive pulmonary disease.* PhD [dissertation]. Tallahassee, Florida: Florida State University;1996.
91. Miravitlles, M, María, L, Oliva-Moreno, J, Hidalgo-Vega, Á. Caregivers' burden in patients with COPD. *International Journal of COPD.* 2015;10(1):347—356. Available from: doi.org/10.2147/COPD.S76091.
92. Lum, HD, Lo, D, Hooker, S, Bekelman, D.B. Caregiving in heart failure: relationship quality is associated with caregiver benefit finding and caregiver burden. Heart & Lung. 2014;43:306-310. Available from: doi.org/10.1016/j.hrtlng.2014.05.002.
93. Luttik, ML, Jaarsma, T, Lesman, I, Sanderman, R, Hagedoorn, M. Quality of life in partners of people with congestive heart failure: gender and involvement in care. *Journal of Advanced Nursing.* 2009;65(7):1442–1451. Available from: doi: 10.1111/j.1365-2648.2009.05004.
94. Kang, X, Li, Z, Nolan, MT. Informal caregivers' experiences of caring for patients with chronic heart failure Systematic review and metasynthesis of qualitative studies. 2011; 26(5),386-394.
95. AasbØ, G, Nyheim Solbrække, K, Kristvik, E, Werner, A. Between disruption and continuity: challenges in maintaining the "biographical we" when caring for a partner with a severe chronic illness. *Sociology of health and illness*. 2016; 38(5):782-796. Available from: doi:10.1111/1467-9566.12396.
96. Wingham, J, Frost, J Britten, N. Behind the smile: qualitative study of caregivers' anguish and management responses while caring for someone living with heart failure. *BMJ Open*. 2017; 7:e014126. Available from: doi:10.1136/bmjopen-2016-014126.
97. Lawton, MP, Moss, M, Kleban, MH, Glicksman, A, Rovine, M. A Two-factor model of caregiving appraisal and psychological well-being. *Journal of Gerontology*. 1991; 46(4): 181–P189. Available from: https://doi.org/10.1093/geronj/46.4.P181.
98. Lee, CS Bidwell, JT, Paturzo, M, Alvaro, R, Cocchieri, A, Jaarsma, T, Stromberg, A, Riegel, B, Vellone, E. Patterns of self-care and clinical events in a cohort of adults with heart failure: 1 year follow-up. *Heart & Lung.* 2018; 47(1): 40-46. Available from: doi: 10.1016/j.hrtlng.2017.09.004
99. Nakken, N, Janssen, DJA, van den Bogaart, EHA, Wouters, EFM, Franssen, FME, Vercoulen, JH, Spruit, MA. *European Respiratory Review.* 2015; 24(137):498-504. Available from: doi: 10.1183/16000617.00010114.
100. Feeney Mahoney, D. Vigilance: Evolution and Definition for Caregivers of Family Members with Alzheimer's Disease. Journal of Gerontological Nursing. 2003;29(8):24-30. Available from: https://doi.org/10.3928/0098-9134-20030801-07
101. Buck, HG, Harkness, K, Wion, R, Carroll, SL, Cosman, T, Kaasalainen, S, Arthur, HM. Caregiver's contributions to heart failure self-care: a systematic review. *European Journal of Cardiovascular Nursing*. 2015;14(1):79-89. Available from: doi:10.1177/1474515113518434.

For peer review only - http://bmjopen.bmj.com/site/about/guidelines.xhtml

102. Dixon-Woods, M, Agarwal, S, Jones, D, Young, B, Sutton, A. Synthesising qualitative and quantitative evidence: a review of possible methods. *Journal of Health Services Research*. 2005;10(1):45-53
103. Bazely, P. Integrating analyses in mixed methods research. SAGE Publications London. 2018

Figure Legend Table 2

***Primary Focus refers to the aims of the papers as identified by the authors. The data extracted from each paper as part of this systematic review may reflect a different focus. Data extracted in this review pertains specifically to caregivers experiences.**

HF = Heart Failure, COPD = Chronic Obstructive Pulmonary Disease, LVAD = Left Ventricular Assist Device, LVAD DT = Left Ventricular Assist Device Destination Therapy, CAD = Coronary Artery Disease, Cg/cg/cgs = Caregiver/s, Pt/Pts/pts = patient/s, N/S = Not stated, CABG = Coronary Artery Bypass Graft, QOL = Quality of Life, ADL = Activities of daily living, Unemp. = unemployed, Emp. = employed. *= Same study

Figure Legend Table 3

CAD – Coronary Artery Disease
HF – Heart Failure
COPD – Chronic Obstructive Pulmonary Disease

*** Caregiver Data based on data collected in 50 studies**
***Patient Data based on data collected in 35 studies**
● There were 57 publications, however there were 54 studies. The following studies utilised the same data but produced two publications: Halm, 2006 & 2007. Saunders, 2008 & 2009. Halm 2016 & 2017.

Figure Legend Table 4(a)
H = High Quality. 4/ 5 out of 5 quality criteria achieved
M= Medium Quality. 3 out of 5 quality criteria achieved
L= Low Quality. 1 or 2 out of 5 quality criteria achieved
•= same study ***Mixed methods study – qualitative component**

Figure Legend Table 4(b)
Study design - CS: cross-sectional design; LS: longitudinal design
Participant sampling – Purp: purposive; Rand: random; Cons: consecutive; Con: convenience; NS; not stated/unclear
Attrition – 20% or less = +1; NS: Not Stated
Outcome measures – V: all validated outcomes; non-V: some or all non-validated; NS: not stated/unclear
Data analysis – MV: multivariate; UV: univariate
•= same study ***Mixed method study – quantitative component**

Overall quality rating
H = High Quality. 4/ 5 out of 5 quality criteria achieved

For peer review only - http://bmjopen.bmj.com/site/about/guidelines.xhtml

M= Medium Quality. 3 out of 5 quality criteria achieved
L= Low Quality. 1 or 2 out of 5 quality criteria achieved

For peer review only

For peer review only - http://bmjopen.bmj.com/site/about/guidelines.xhtml

For peer review only

For peer review only - http://bmjopen.bmj.com/site/about/guidelines.xhtml

For peer review only

For peer review only - http://bmjopen.bmj.com/site/about/guidelines.xhtml

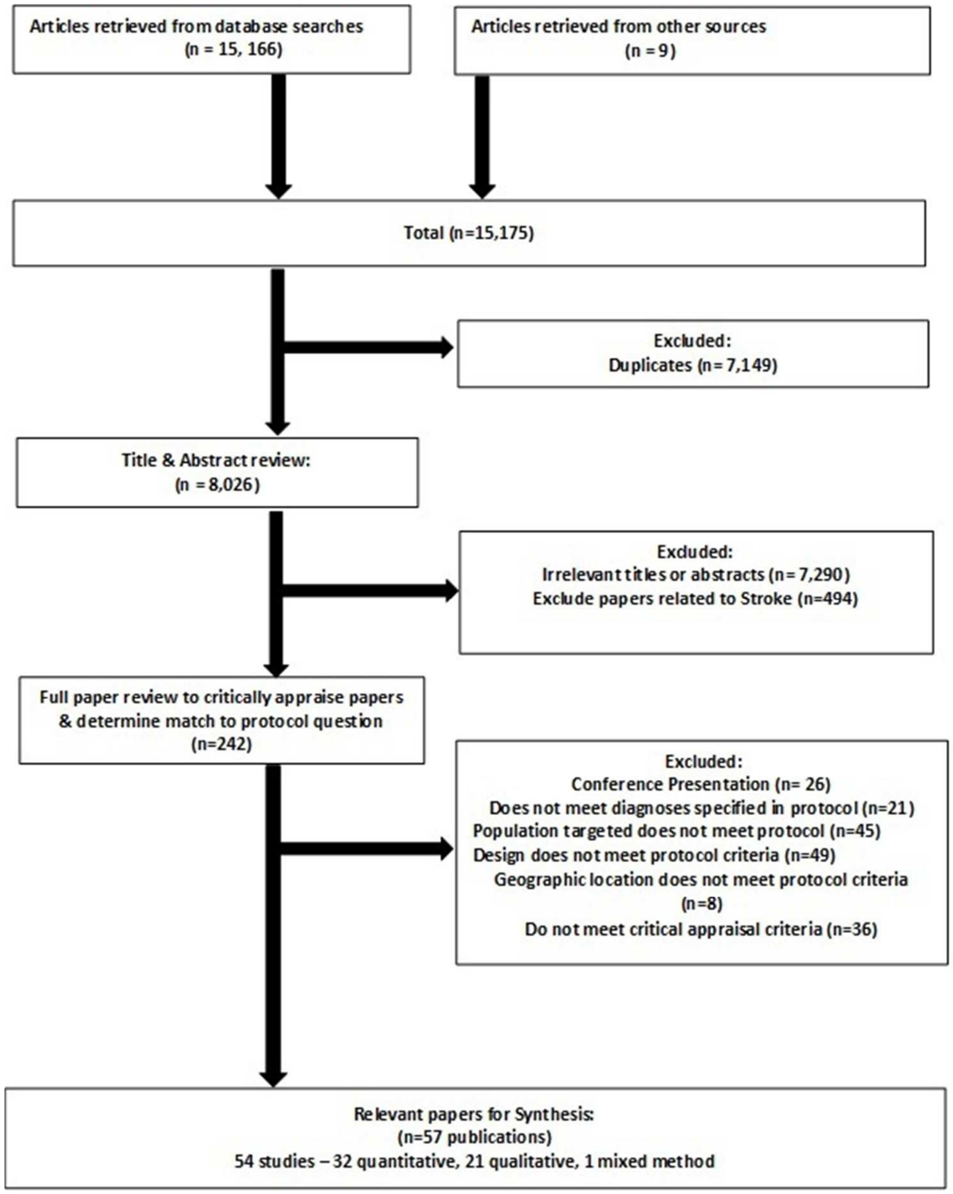

52x62mm (300 x 300 DPI)

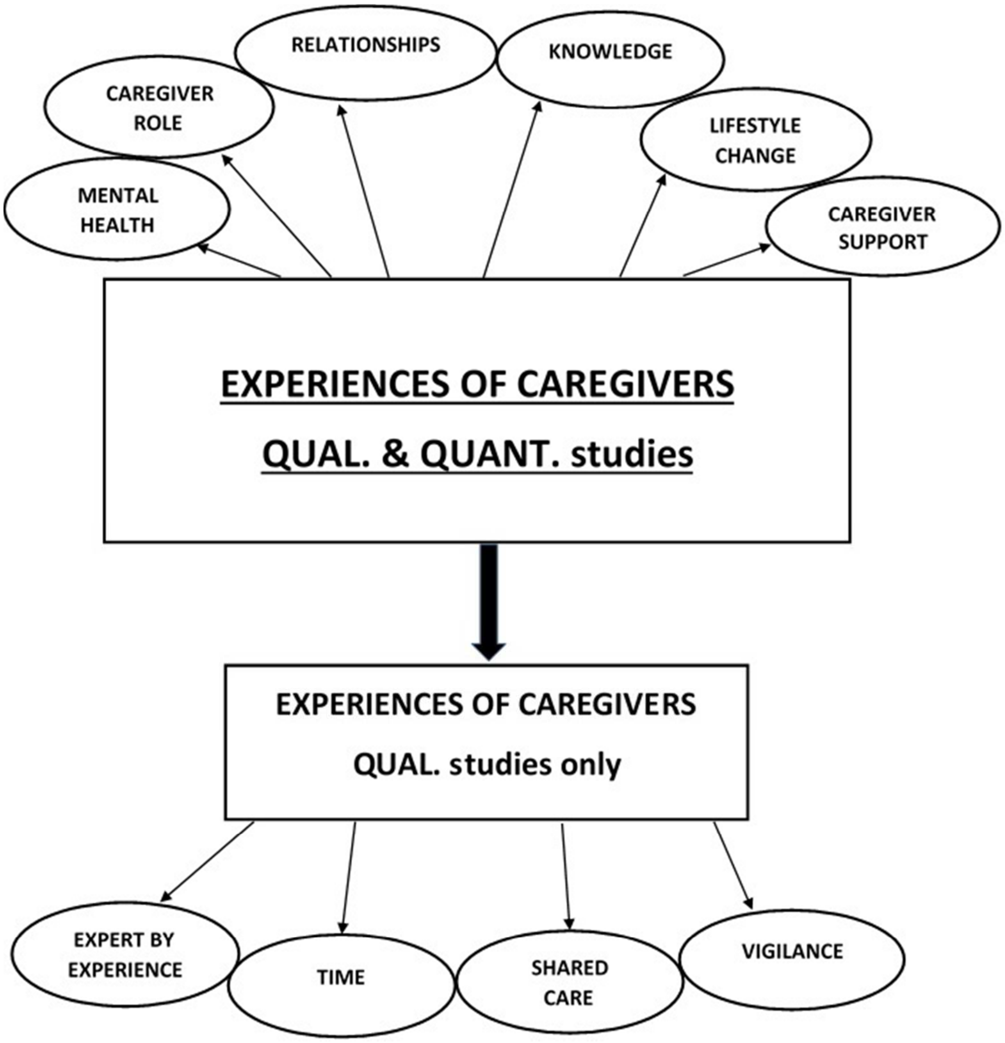

59x61mm (300 x 300 DPI)

Supplementary:

Table 1: List of search terms

Medline:

MeSH: Caregivers

“Caregivers”.ti,ab OR “care-giver”.ti,ab OR “carer*.ti,ab” OR “informal car*”.ti,ab

AND

MeSH: Quality of life

“Quality of life” OR “outcome measures” OR “caregiver outcomes” OR “care-giver outcomes” OR “time use” OR “time-use” OR “occupations” OR “occupational engagement” OR “self-efficacy” OR “self efficacy” OR “experience” OR “emotion*” OR “psychological health impact” OR “physical health impact” OR “confidence” OR “self-confidence” OR “satisfaction” OR “dissatisfaction” OR “activities of daily living” OR “resilience” OR “social adj2 (interaction or engagement or support) OR “Social adj2 (participation)” OR “caregiver adj2 (support or health professional or medical team or nurse or patient or relationship* or knowledge)” OR “information exchange” OR “coping strategies” OR “leisure activity” OR “conflict” OR “caregiver responsib*” OR care-giver responsib*” OR “caregiver expectation” OR “care-giver expectation” OR “caregiver role” OR “care-giver role” OR “role adjustment” OR “caregiver or care-giver adj2 (attitude to health)”

AND

MeSH: Heart Failure, Heart Diseases, Myocardial Ischaemia, Coronary Artery Disease, Pulmonary Disease, Chronic Obstructive, Pulmonary Heart Disease, Chronic Obstructive Pulmonary Disease, Acute Coronary Syndrome, Cardiovascular Diseases, Coronary Artery Disease, Coronary Artery Bypass, Coronary Disease, Coronary Aneurysm, Stroke, Myocardial Infarction

“Heart Failure”.ti,ab OR “cardiac failure”.ti,ab OR “myocardial failure”.ti,ab OR “left ventricular failure”.ti,ab OR “right ventricular failure”.ti,ab OR “cardiomyopathy”.ti,ab, OR “systolic failure”.ti,ab OR “diastolic failure”.ti,ab OR “Chronic Obstructive Pulmonary Disease”.ti,ab OR “COPD”.ti,ab OR “chronic obstructive lung disease”.ti,ab OR “pulmonary disease”.ti,ab OR “pulmonary disorder”.ti,ab OR “respiratory disease”.ti,ab OR “respiratory disorder”.ti,ab OR “dyspnea”.ti,ab OR “dyspnoea”.ti,ab OR “Stroke”.ti,ab OR “stroke disability”.ti,ab OR “stroke disease”.ti,ab OR “stroke disorder”.ti,ab OR “CVA”.ti,ab OR “cerebrovascular accident”.ti,ab OR “transient ischaemic attack”.ti,ab OR “transient ischemic attack”.ti,ab OR “haemorrhage”.ti,ab OR “hemorrhage”.ti,ab OR “cerebral haemorrhage”.ti,ab OR “cerebral hemorrhage”.ti,ab OR “aneurysm”.ti,ab OR “atrial fibrillation”.ti,ab OR “Coronary Artery Disease”.ti,ab OR “acute coronary syndrome”.ti,ab OR “atherosclerosis”.ti,ab OR “arteriosclerosis”.ti,ab OR “ischaemic heart disease”,ti.ab OR “ischemic heart disease”.ti,ab OR “myocardial infarction”,ti.ab OR “coronary revascularisation”.ti,ab OR “coronary revascularization”.ti,ab OR “angina”.ti,ab OR “CABG”.ti,ab OR “coronary artery bypass graft”.ti,ab OR “stable angina”.ti,ab OR “angina pectoris”.ti,ab

For peer review only

CINAHL:

MeSH: Caregivers, caregiver burden, caregiver supports, caregiver role strain, risk for caregiver role strain, family caregiver status, caregiving endurance potential, caregiver-patient relationship, caregiver well-being, caregiver support, caregiver stressors, caregiver physical health, caregiver performance, caregiver performance: direct care, caregiver performance indirect care, caregiver lifestyle disruption, caregiver emotional health, caregiver strain index, caregiver role strain.

"Caregiver".ti,ab OR "care-giver".ti,ab OR "carer*.ti,ab" OR "carer".ti,ab OR "carers".ti,ab OR "informal care*".ti,ab

AND

MeSH: Quality of life

"Quality of life" OR "outcome measures" OR "caregiver outcomes" OR "time use" OR "occupations" OR "occupational engagement" OR "self-efficacy" OR "self efficacy" OR "experience" OR "emotion*" OR "psychological health impact" OR "physical health impact" OR "confidence" OR "self-confidence" OR "satisfaction" OR "dissatisfaction" OR "activities of daily living" OR "resilience" OR "social adj2 (interaction or engagement or support) OR "Social participation" OR "caregiver adj2 (support or health professional or medical team or nurse or patient or relationship* or knowledge)" OR "information exchange" OR "coping strategies" OR "leisure activity" OR "conflict" OR "caregiver responsib*" OR care-giver responsib*" OR "caregiver expectation" OR "care-giver expectation" OR "caregiver role" OR "care-giver role" OR "role adjustment" OR "caregiver or care-giver adj2 (adjustment)" OR "caregiver or care-giver adj2 (acceptance) OR "caregiver or care-giver adj2 (readiness)" OR "caregiver or care-giver adj2 (attitude to health)"

AND

MeSH: Heart Failure, Treatment Failure, Pulmonary Disease, Chronic Obstructive Pulmonary Disease, Chronic Obstructive Pulmonary Disease, Acute Coronary Syndrome, Coronary Arteriosclerosis, Coronary Artery Bypass, Coronary Disease, Coronary Aneurysm, Stroke OR Coronary Artery Disease

"Heart Failure".ti,ab OR "cardiac failure".ti,ab OR "myocardial failure".ti,ab OR "left ventricular failure".ti,ab OR "right ventricular failure".ti,ab OR "cardiomyopathy".ti,ab, OR "systolic failure".ti,ab OR "diastolic failure".ti,ab OR "COPD".ti,ab OR "chronic obstructive lung disease".ti,ab OR "pulmonary disease".ti,ab OR "pulmonary disorder".ti,ab OR "respiratory disease".ti,ab OR "respiratory disorder".ti,ab OR "dyspnea".ti,ab OR "dyspnoea".ti,ab OR "Stroke".ti,ab OR "stroke disability".ti,ab OR "stroke disease".ti,ab OR "stroke disorder".ti,ab OR "CVA".ti,ab OR "cerebrovascular accident".ti,ab OR "transient ischaemic attack".ti,ab OR "transient ischemic attack".ti,ab OR "haemorrhage".ti,ab OR "hemorrhage".ti,ab OR "cerebral haemorrhage".ti,ab OR "cerebral hemorrhage".ti,ab OR "aneurysm".ti,ab OR "atrial fibrillation".ti,ab OR "Coronary Artery Disease".ti,ab OR "acute coronary syndrome".ti,ab OR "atherosclerosis".ti,ab OR "arteriosclerosis".ti,ab OR "ischaemic heart disease",ti.ab OR "ischemic heart disease".ti,ab OR "myocardial infarction",ti.ab OR "coronary revascularisation".ti,ab OR "coronary revascularization".ti,ab OR "angina".ti,ab OR "CABG".ti,ab OR "coronary artery bypass graft".ti,ab OR "stable angina".ti,ab OR "angina pectoris".ti,ab

For peer review only

EMBASE:

MeSH: Caregivers

“Caregivers”.ti,ab OR “care-givers”.ti,ab OR “Caregiver”.ti,ab OR “care-giver”.ti,ab OR “carer*.ti,ab” OR “informal car*”.ti,ab

AND

MeSH: Quality of life

“Quality of life” OR “outcome measures” OR “caregiver outcomes” OR “care-giver outcomes” OR “time use” OR “occupations” OR “occupational engagement” OR (“self-efficacy” OR “self efficacy”) OR “experience” OR “emotion*” OR “psychological health impact” OR “physical health impact” OR (“confidence” OR “self-confidence”) OR “satisfaction” OR “dissatisfaction” OR “activities of daily living” OR “resilience” OR “social adj2 (interaction or engagement or support) OR “Social participation” OR “caregiver OR care-giver adj2 (support or health professional or medical team or nurse or patient or relationship* or knowledge)” OR “information exchange” OR “coping strategies” OR “leisure activity” OR “conflict” OR “caregiver responsib*” OR care-giver responsib*” OR “caregiver expectation” OR “care-giver expectation” OR “caregiver role” OR “care-giver role” OR “role adjustment” OR “caregiver or care-giver adj2 (adjustment or acceptance or readiness)” OR “caregiver or care-giver adj2 (attitude to health)”

AND

MeSH: Heart Failure, Chronic Obstructive Pulmonary Disease, Bronchitis, Chronic Obstructive, Disease exacerbation, Pulmonary Disease, Cardiovascular Disease, Stroke, Cerebrovascular Accident, Coronary Artery Disease, Ischemic Heart Disease, Coronary Artery Disease, Acute Coronary Syndrome, Heart Infarction, Coronary Aneurysm, Coronary Artery Aneurysm, Coronary Artery Bypass, Coronary Artery Bypass Graft, Coronary arteriosclerosis, Coronary Artery Atherosclerosis

“heart failure”,ti,ab OR “cardiac failure”.ti,ab OR “myocardial failure”.ti,ab OR “left ventricular failure”.ti,ab OR “right ventricular failure”.ti,ab OR “cardiomyopathy”.ti,ab, OR “systolic failure”.ti,ab OR “diastolic failure”.ti,ab OR “chronic obstructive pulmonary disease” OR “COPD”.ti,ab OR “chronic obstructive lung disease”.ti,ab OR “pulmonary disease”.ti,ab OR “pulmonary disorder”.ti,ab OR “respiratory disease”.ti,ab OR “respiratory disorder”.ti,ab OR “stroke disorder”.ti,ab OR “CVA”.ti,ab OR “cerebrovascular accident”.ti,ab OR “transient ischaemic attack”.ti,ab OR “transient ischemic attack”.ti,ab OR “haemorrhage”.ti,ab OR “hemorrhage”.ti,ab OR “cerebral haemorrhage”.ti,ab OR “cerebral hemorrhage”.ti,ab OR “aneurysm”.ti,ab OR “atrial fibrillation”.ti,ab OR “Coronary Artery Disease”.ti,ab OR “acute coronary syndrome”.ti,ab OR “atherosclerosis”.ti,ab OR “arteriosclerosis”.ti,ab OR “ischaemic heart disease”,ti.ab OR “ischemic heart disease”.ti,ab OR “myocardial infarction”,ti.ab OR “coronary revascularisation”.ti,ab OR “coronary revascularization”.ti,ab OR “angina”.ti,ab OR “CABG”.ti,ab OR “coronary artery bypass graft”.ti,ab OR “stable angina”.ti,ab OR “angina pectoris”.ti,ab

For peer review only

PsycInfo:

MeSH: Caregivers

“caregiver”.ti,ab OR “care-giver”.ti,ab OR “carer*.ti,ab” OR “informal car*”.ti,ab

AND

MeSH: Quality of life, Occupations, Well-being, Activities of daily living, Mental Health, Health Behaviour, Emotional Responses, Life satisfaction, Role satisfaction, Resilience, Life Experience, Communication, Strategies, Anxiety, Coping Behaviour, Distress, Recreation, Leisure Time, Participation, Adjustment, Couples

“Quality of life” OR “outcome measures” OR (“caregiver outcomes” OR “care-giver outcomes”) OR (“time-use” OR “time use”) OR “occupations” OR “occupational engagement” OR (“self-efficacy” OR “self efficacy”) OR “experience” OR “emotion*” OR “psychological health impact” OR “physical health impact” OR (“confidence” OR “self-confidence” OR self confidence) OR “satisfaction” OR “dissatisfaction” OR “activities of daily living” OR “resilience” OR “social adj2 (interaction or engagement or support or participation) OR “caregiver OR care-giver adj2 (support or health professional or medical team)” OR “information exchange” OR “coping strategies” OR “leisure activity” OR “conflict” OR “caregiver responsib*” OR care-giver responsib*” OR “caregiver expectation” OR “care-giver expectation” OR “caregiver role” OR “care-giver role” OR “role adjustment” OR “caregiver or care-giver adj2 (adjustment or acceptance or readiness)” OR “caregiver or care-giver adj2 (attitude to health)”

AND

MeSH: Heart Failure, Health Behaviour, Heart Disorders, Cardiovascular Disorders, Self-care skills, Intervention, Treatment Outcomes, Chronic Obstructive Pulmonary Disease, Pulmonary Heart Disease, Stroke, Treatment Outcomes, Intervention, Cerebrovascular Accidents, Coronary Artery Disease, Myocardial Infarctions, Ischemia, Aneurysms

“heart failure”,ti,ab OR “cardiac failure”.ti,ab OR “myocardial failure”.ti,ab OR “left ventricular failure”.ti,ab OR “right ventricular failure”.ti,ab OR “cardiomyopathy”.ti,ab, OR “systolic failure”.ti,ab OR “diastolic failure”.ti,ab OR “chronic obstructive pulmonary disease” OR “COPD”.ti,ab OR “chronic obstructive lung disease”.ti,ab OR “pulmonary disease”.ti,ab OR “pulmonary disorder”.ti,ab OR “respiratory disease”.ti,ab OR “respiratory disorder”.ti,ab OR “dyspnea”.ti,ab OR “dyspnoea”,ti,ab OR “stroke disorder”.ti,ab OR “CVA”.ti,ab OR “cerebrovascular accident”.ti,ab OR “transient ischaemic attack”.ti,ab OR “transient ischemic attack”.ti,ab OR “haemorrhage”.ti,ab OR “hemorrhage”.ti,ab OR “cerebral haemorrhage”.ti,ab OR “cerebral hemorrhage”.ti,ab OR “aneurysm”.ti,ab OR “atrial fibrillation”.ti,ab OR “Coronary Artery Disease”.ti,ab OR “acute coronary syndrome”.ti,ab OR “atherosclerosis”.ti,ab OR “arteriosclerosis”.ti,ab OR “ischaemic heart disease”,ti.ab OR “ischemic heart disease”.ti,ab OR “myocardial infarction”,ti.ab OR “coronary revascularisation”.ti,ab OR “coronary revascularization”.ti,ab OR “angina”.ti,ab OR “CABG”.ti,ab OR “coronary artery bypass graft”.ti,ab OR “stable angina”.ti,ab OR “angina pectoris”.ti,ab

For peer review only

Web of Science

TI = Title

TS = Topic

TI & TS = “caregiver” OR “care-giver” OR “carer*” OR “informal care*”

AND

TS = “Quality of life” OR “outcome measures” OR (“caregiver outcomes” OR “care-giver outcomes”) OR (“time-use” OR “time use”) OR “occupations” OR “occupational engagement” OR (“self-efficacy” OR “self efficacy”) OR “experience” OR “emotion*” OR “psychological health impact” OR “physical health impact” OR (“confidence” OR “self-confidence” OR self confidence) OR “satisfaction” OR “dissatisfaction” OR “activities of daily living” OR “resilience” OR “social adj2 (interaction or engagement or support or participation) OR “caregiver OR care-giver adj2 (support or health professional or medical team)” OR “information exchange” OR “coping strategies” OR “leisure activity” OR “conflict” OR “caregiver responsib*” OR care-giver responsib*” OR “caregiver expectation” OR “care-giver expectation” OR “caregiver role” OR “care-giver role” OR “role adjustment” OR “caregiver or care-giver adj2 (adjustment or acceptance or readiness)” OR “caregiver or care-giver adj2 (attitude to health)”

AND

TI & TS = “heart failure” OR “cardiac failure” OR “myocardial failure” OR “left ventricular failure” OR “right ventricular failure” OR “cardiomyopathy” OR “systolic failure” OR “diastolic failure” OR “chronic obstructive pulmonary disease” OR “COPD” OR “chronic obstructive lung disease” OR “pulmonary disease” OR “pulmonary disorder” OR “respiratory disease” OR “respiratory disorder” OR “dyspnea” OR “dyspnoea” OR “stroke disorder” OR “CVA” OR “cerebrovascular accident” OR “transient ischaemic attack” OR “transient ischemic attack” OR “haemorrhage” OR “hemorrhage” OR “cerebral haemorrhage” OR “cerebral hemorrhage” OR “aneurysm” OR “atrial fibrillation” OR “Coronary Artery Disease” OR “acute coronary syndrome” OR “atherosclerosis” OR “arteriosclerosis” OR “ischaemic heart disease” OR “ischemic heart disease” OR “myocardial infarction” OR “coronary revascularisation” OR “coronary revascularization” OR “angina” OR “CABG” OR “coronary artery bypass graft” OR “stable angina” OR “angina pectoris”

For peer review only

ProQuest: Global Dissertations & Theses/ Applied Social Sciences Index and Abstracts (ASSIA)

“caregiver”.ti,ab OR “care-giver”.ti,ab OR “carer*.ti,ab” OR “informal car*”.ti,ab

AND

“Quality of life” OR “outcome measures” OR (“caregiver outcomes” OR “care-giver outcomes”) OR (“time-use” OR “time use”) OR “occupations” OR “occupational engagement” OR (“self-efficacy” OR “self efficacy”) OR “experience” OR “emotion*” OR “psychological health impact” OR “physical health impact” OR (“confidence” OR “self-confidence” OR self confidence) OR “satisfaction” OR “dissatisfaction” OR “activities of daily living” OR “resilience” OR “social adj2 (interaction or engagement or support or participation) OR “caregiver OR care-giver adj2 (support or health professional or medical team)” OR “information exchange” OR “coping strategies” OR “leisure activity” OR “conflict” OR “caregiver responsib*” OR care-giver responsib*” OR “caregiver expectation” OR “care-giver expectation” OR “caregiver role” OR “care-giver role” OR “role adjustment” OR “caregiver or care-giver adj2 (adjustment or acceptance or readiness)” OR “caregiver or care-giver adj2 (attitude to health)”

AND

“heart failure”,ti,ab OR “cardiac failure”.ti,ab OR “myocardial failure”.ti,ab OR “left ventricular failure”.ti,ab OR “right ventricular failure”.ti,ab OR “cardiomyopathy”.ti,ab, OR “systolic failure”.ti,ab OR “diastolic failure”.ti,ab OR “chronic obstructive pulmonary disease” OR “COPD”.ti,ab OR “chronic obstructive lung disease”.ti,ab OR “pulmonary disease”.ti,ab OR “pulmonary disorder”.ti,ab OR “respiratory disease”.ti,ab OR “respiratory disorder”.ti,ab OR “dyspnea”.ti,ab OR “dyspnoea”,ti,ab OR “stroke disorder”.ti,ab OR “CVA”.ti,ab OR “cerebrovascular accident”.ti,ab OR “transient ischaemic attack”.ti,ab OR “transient ischemic attack”.ti,ab OR “haemorrhage”.ti,ab OR “hemorrhage”.ti,ab OR “cerebral haemorrhage”.ti,ab OR “cerebral hemorrhage”.ti,ab OR “aneurysm”.ti,ab OR “atrial fibrillation”.ti,ab OR “Coronary Artery Disease”.ti,ab OR “acute coronary syndrome”.ti,ab OR “atherosclerosis”.ti,ab OR “arteriosclerosis”.ti,ab OR “ischaemic heart disease”,ti.ab OR “ischemic heart disease”.ti,ab OR “myocardial infarction”,ti.ab OR “coronary revascularisation”.ti,ab OR “coronary revascularization”.ti,ab OR “angina”.ti,ab OR “CABG”.ti,ab OR “coronary artery bypass graft”.ti,ab OR “stable angina”.ti,ab OR “angina pectoris”.ti,ab

For peer review only

Ethos – British Library

CAREGIVER or CARE-GIVER.ti,ab

Searches initially included Heart Failure, Chronic Obstructive Pulmonary Disease, Stroke and Coronary Artery Disease as "AND" terms, however these yielded no results

For peer review only

Table 2 (a)

Caregiver experience - Concepts/Mapping Table – Qualitative Papers

MENTAL HEALTH = 1 **ROLE = 2** **LIFESTYLE CHANGE = 3**
SUPPORT = 4 **KNOWLEDGE/INFORMATION = 5**
RELATIONSHIPS = 6

Concepts only in Qualitative Papers: **EXPERT BY EXPERIENCE = 7** **VIGILANCE = 8**
TIME = 9 **SHARED CARE = 10**

	**1**	**2**	**3**	**4**	**5**	**6**	**7**	**8**	**9**	**10**
Andersen (2015) **(HF)**	✓		✓	✓	✓					✓
Baker (2010) **(HF)**	✓	✓	✓	✓		✓		✓		✓
Bove (2016) **(COPD)**	✓	✓	✓	✓	✓	✓	✓	✓	✓	✓
Burke (2014) **(HF)**	✓	✓	✓	✓	✓	✓	✓	✓		✓
Clark (2008) **(HF)**		✓	✓	✓	✓	✓	✓	✓	✓	✓
Figueiredo (2015) **(COPD)**	✓	✓	✓	✓	✓	✓	✓	✓	✓	
• Halm (2016) **(CAD)**	✓	✓	✓	✓	✓	✓	✓	✓	✓	✓
• Halm (2017) **(CAD)**	✓	✓	✓	✓	✓	✓	✓	✓	✓	✓
Hynes (2012) **(COPD)**	✓	✓	✓	✓	✓	✓	✓	✓	✓	✓
Imes (2011) **(HF)**	✓	✓	✓	✓	✓	✓		✓	✓	✓
Kitko (2010) **(HF)**	✓	✓	✓	✓	✓	✓	✓	✓	✓	✓
Liljeroos (2014) **(HF)**	✓		✓	✓	✓	✓		✓	✓	✓
Lindqvist (2013) **(COPD)**	✓	✓	✓	✓	✓	✓		✓	✓	✓
Luttik (2007) **(HF)**	✓	✓	✓	✓	✓	✓	✓	✓	✓	✓
Marcuccilli (2011) **(HF)**	✓		✓	✓		✓	✓	✓	✓	✓
Marcuccilli (2014) **(HF)**	✓	✓	✓	✓	✓	✓		✓	✓	
Pattenden (2007) **(HF)**	✓	✓	✓	✓		✓		✓		
Rolley (2010) **(CAD)**	✓	✓	✓	✓	✓	✓		✓		
Spence (2008) **(COPD)**	✓	✓	✓	✓	✓	✓	✓	✓	✓	✓

Strom (2015) **(HF)**	✓	✓	✓	✓		✓	✓	✓		✓
Wallin (2013) **(CAD)**	✓	✓	✓	✓	✓	✓			✓	
Wingham (2015) **(HF)**	✓	✓	✓	✓	✓	✓	✓	✓	✓	✓
**Heart Failure**	**12**	**10**	**13**	**13**	**9**	**12**	**7**	**12**	**8**	**11**
**COPD**	**5**	**5**	**5**	**5**	**5**	**5**	**4**	**5**	**5**	**4**
**CAD**	**3**	**3**	**3**	**4**	**3**	**3**	**1**	**2**	**2**	**1**
**Total**	**20**	**18**	**21**	**21**	**17**	**20**	**12**	**19**	**15**	**16**

• = same study: counted as one

For peer review only

Table 2 (b)

Caregiver experience - Concepts/Mapping – Quantitative Papers

MENTAL HEALTH IMPACT = 1 **ROLE = 2** **LIFESTYLE CHANGE = 3**
SUPPORT = 4 **KNOWLEDGE/INFORMATION = 5**
RELATIONSHIPS = 6

	1	2	3	4	5	6
Agren (2010) **(HF)**	✓					✓
Al-Rawashdeh (2017) **(HF)**	✓		✓			✓
Badr (2017) **(COPD)**	✓		✓			✓
Bakas (2006) **(HF)**	✓	✓	✓			
Chung (2016) **(HF)**	✓	✓	✓			
Cossette (1993) **(COPD)**	✓	✓		✓		
Evangelista (2002) **(HF)**	✓					✓
Figueiredo (2013) **(COPD)**	✓		✓			✓
Figueiredo (2014) **(COPD)**	✓					✓
Grigorovich (2017) **(HF)**	✓		✓	✓		
• Halm (2006) **(CAD)**	✓			✓		✓
• Halm (2007) **(CAD)**	✓			✓		✓
Hess (2009) **(HF)**		✓				
Hooley (2005) **(HF)**	✓					✓
Hwang (2011) **(HF)**	✓	✓		✓		✓
Karmilovich (1994) **(HF)**	✓	✓			✓	✓
Kneeshaw (1999) **(CAD)**		✓	✓	✓	✓	✓
Loftus (2004) **(HF)**	✓	✓		✓		✓
Lum (2014) **(HF)**						✓
Luttik (2009) **(HF)**						✓

For peer review only

Miravitlles (2015) **(COPD)**			✓			✓
Nakken (2017) **(COPD)**	✓		✓	✓		✓
Park (2013) **(CAD)**		✓	✓			✓
Pi-Ming Yeh (2012) **(HF)**	✓			✓		
Pressler (2013) **(HF)**	✓	✓	✓			✓
Riegner (1996) **(COPD)**		✓		✓		✓
• Saunders (2008) **(HF)**	✓			✓		✓
• Saunders (2009) **(HF)**	✓			✓		✓
Saunders (2010) **(HF)**	✓	✓				✓
Schwarz (2003) **(HF)**	✓			✓		
Scott (2000) **(HF)**	✓	✓	✓	✓		
Takata (2008) **(COPD)**	✓	✓	✓	✓	✓	
Vellone (2015) **(HF)**	✓				✓	✓
Woolfe (2007) **(COPD)**	✓		✓	✓	✓	
**Heart Failure**	**15**	**9**	**6**	**7**	**2**	**13**
**COPD**	**5**	**3**	**6**	**5**	**2**	**6**
**CAD**	**4**	**2**	**2**	**3**	**1**	**3**
**Mixed Method Study**	**1**					
**Total**	**25**	**14**	**14**	**15**	**5**	**22**

• = same study: counted as one

For peer review only

Table 2 (c)

Caregiver experience - Concepts/Mapping – Mixed Method Papers

MENTAL HEALTH IMPACT = 1 **ROLE = 2** **LIFESTYLE CHANGE = 3**
SUPPORT = 4 **KNOWLEDGE/INFORMATION = 5**
RELATIONSHIPS = 6

	**1**	**2**	**3**	**4**	**5**	**6**
Näsström (2017) **(HF)**	✓			✓	✓	✓
**TOTAL**	**1**			**1**	**1**	1

For peer review only

PRISMA 2009 Checklist

Section/topic	#	Checklist item	Reported on page #
**TITLE**			
Title	1	Identify the report as a systematic review, meta-analysis, or both.	1
**ABSTRACT**			
Structured summary	2	Provide a structured summary including, as applicable: background; objectives; data sources; study eligibility criteria, participants, and interventions; study appraisal and synthesis methods; results; limitations; conclusions and implications of key findings; systematic review registration number.	2
**INTRODUCTION**			
Rationale	3	Describe the rationale for the review in the context of what is already known.	4,5
Objectives	4	Provide an explicit statement of questions being addressed with reference to participants, interventions, comparisons, outcomes, and study design (PICOS).	4,5,6
**METHODS**			
Protocol and registration	5	Indicate if a review protocol exists, if and where it can be accessed (e.g., Web address), and, if available, provide registration information including registration number.	3
Eligibility criteria	6	Specify study characteristics (e.g., PICOS, length of follow-up) and report characteristics (e.g., years considered, language, publication status) used as criteria for eligibility, giving rationale.	5
Information sources	7	Describe all information sources (e.g., databases with dates of coverage, contact with study authors to identify additional studies) in the search and date last searched.	5
Search	8	Present full electronic search strategy for at least one database, including any limits used, such that it could be repeated.	5
Study selection	9	State the process for selecting studies (i.e., screening, eligibility, included in systematic review, and, if applicable, included in the meta-analysis).	5,6
Data collection process	10	Describe method of data extraction from reports (e.g., piloted forms, independently, in duplicate) and any processes for obtaining and confirming data from investigators.	6
Data items	11	List and define all variables for which data were sought (e.g., PICOS, funding sources) and any assumptions and simplifications made.	5,6
Risk of bias in individual studies	12	Describe methods used for assessing risk of bias of individual studies (including specification of whether this was done at the study or outcome level), and how this information is to be used in any data synthesis.	5,6,7,28,29,30,31
Summary measures	13	State the principal summary measures (e.g., risk ratio, difference in means).	6,7
Synthesis of results	14	Describe the methods of handling data and combining results of studies, if done, including measures of consistency (e.g., I^2) for each meta-analysis.	6,7,8,9

For peer review only
For peer review only - http://bmjopen.bmj.com/site/about/guidelines.xhtml

PRISMA 2009 Checklist

Section/topic	#	Checklist item	Reported on page #
Risk of bias across studies	15	Specify any assessment of risk of bias that may affect the cumulative evidence (e.g., publication bias, selective reporting within studies).	5,6,7,28,29,30,31
Additional analyses	16	Describe methods of additional analyses (e.g., sensitivity or subgroup analyses, meta-regression), if done, indicating which were pre-specified.	6,7
**RESULTS**			
Study selection	17	Give numbers of studies screened, assessed for eligibility, and included in the review, with reasons for exclusions at each stage, ideally with a flow diagram.	11
Study characteristics	18	For each study, present characteristics for which data were extracted (e.g., study size, PICOS, follow-up period) and provide the citations.	12-24
Risk of bias within studies	19	Present data on risk of bias of each study and, if available, any outcome level assessment (see item 12).	5,6,28,29,30,31
Results of individual studies	20	For all outcomes considered (benefits or harms), present, for each study: (a) simple summary data for each intervention group (b) effect estimates and confidence intervals, ideally with a forest plot.	12-24, 26,27
Synthesis of results	21	Present results of each meta-analysis done, including confidence intervals and measures of consistency.	12-24 32,33
Risk of bias across studies	22	Present results of any assessment of risk of bias across studies (see Item 15).	5,6,28,29,30,31
Additional analysis	23	Give results of additional analyses, if done (e.g., sensitivity or subgroup analyses, meta-regression [see Item 16]).	32,33
**DISCUSSION**			
Summary of evidence	24	Summarize the main findings including the strength of evidence for each main outcome; consider their relevance to key groups (e.g., healthcare providers, users, and policy makers).	32,33,34,35,36,37,38
Limitations	25	Discuss limitations at study and outcome level (e.g., risk of bias), and at review-level (e.g., incomplete retrieval of identified research, reporting bias).	40,41
Conclusions	26	Provide a general interpretation of the results in the context of other evidence, and implications for future research.	41
**FUNDING**			
Funding	27	Describe sources of funding for the systematic review and other support (e.g., supply of data); role of funders for the systematic review.	42

From: Moher D, Liberati A, Tetzlaff J, Altman DG, The PRISMA Group (2009). Preferred Reporting Items for Systematic Reviews and Meta-Analyses: The PRISMA Statement. PLoS Med 6(7): e1000097. doi:10.1371/journal.pmed1000097

For more information, visit: **www.prisma-statement.org**.

For peer review only - http://bmjopen.bmj.com/site/about/guidelines.xhtml

“Who Cares?” The Experiences of Caregivers of Adults Living with Heart Failure, Chronic Obstructive Pulmonary Disease and Coronary Artery Disease – A Mixed Methods Systematic Review

Journal:	*BMJ Open*
Manuscript ID	bmjopen-2017-020927.R2
Article Type:	Research
Date Submitted by the Author:	09-Apr-2018
Complete List of Authors:	Noonan, Miriam; University of Exeter Medical School, European Centre for Environment and Human Health<br>Wingham, Jennifer; Royal Cornwall Hospitals NHS Trust, Research, Development and Innovation; University of Exeter Medical School,<br>Taylor, Rod; University of Exeter, Peninsula Medical School
**Primary Subject Heading**:	Patient-centred medicine
Secondary Subject Heading:	Evidence based practice, Cardiovascular medicine, Respiratory medicine, Research methods, Health services research
Keywords:	Caregivers, Heart failure < CARDIOLOGY, Coronary heart disease < CARDIOLOGY, Chronic obstructive pulmonary disease, Mixed methods systematic review

SCHOLARONE™
Manuscripts

"Who Cares?" The Experiences of Caregivers of Adults Living with Heart Failure, Chronic Obstructive Pulmonary Disease and Coronary Artery Disease – A Mixed Methods Systematic Review

M Noonan[1], J Wingham,[2,] RS Taylor[3]

Author Affiliations:

1. Miriam Noonan. University of Exeter Medical School, European Centre for Environment and Human Health, Knowledge Spa, Royal Cornwall Hospital, Truro, Cornwall, UK.

2. Dr. Jennifer Wingham. Royal Cornwall Hospitals NHS Trust, Research, Development and Innovation, F37, Knowledge Spa, Royal Cornwall Hospital, Truro, Cornwall & University of Exeter Medical School, Exeter, UK.

3. Professor Rod S. Taylor. University of Exeter Medical School, Exeter, UK.

Correspondence to
Miriam C. Noonan
University of Exeter Medical School
European Centre for Environment and Human Health,
Knowledge Spa, Royal Cornwall Hospital, Truro, Cornwall, UK TR13HD, UK
mn354@exeter.ac.uk

(Word Count 4,267)

For peer review only

For peer review only - http://bmjopen.bmj.com/site/about/guidelines.xhtml

ABSTRACT

Objective To assess the experiences of unpaid caregivers providing care to people with heart failure (HF) or chronic obstructive pulmonary disease (COPD) or coronary artery disease (CAD).

Design Mixed methods systematic review including qualitative and quantitative studies.

Data Sources Databases searched: Medline Ebsco, PsycInfo, CINAHL Plus with Full Text, Embase, Web of Science, Ethos: The British Library and ProQuest. Grey literature identified using: Global Dissertations and Theses and Applied Sciences Index and hand searches and citation checking of included references. Search time frame: 1st January 1990 to 30th August 2017.

Eligibility criteria for selecting studies Inclusion was limited to English language studies in unpaid adult caregivers (>18 years), providing care for patients with HF, COPD or CAD. Studies that considered caregivers for any other diagnoses and studies undertaken in low and middle income countries were excluded. Quality assessment of included studies was conducted by two authors.

Data analysis/synthesis - A results based convergent synthesis was conducted

Results Searches returned 8,026 titles and abstracts. 54 studies - 21 qualitative, 32 quantitative, and 1 mixed were included. This totalled 26,453 caregivers who were primarily female (63 %), with average age of 62.2 years. Narrative synthesis yielded six concepts related to caregiver experience: (1) mental health (2) caregiver role (3) lifestyle change (4) support for caregivers (5) knowledge and (6) relationships. There was a discordance between paradigms regarding emerging concepts. Four concepts emerged from qualitative papers which were not present in quantitative papers (1) expert by experience (2) vigilance (3) shared care and (4) time.

Conclusion Caregiving is life altering and complex with significant health implications. Health professionals should support caregivers who in turn can facilitate the recipient to manage their long term condition. Further longitudinal research exploring the evolution of caregiver experiences over time of patients with chronic cardiopulmonary conditions is required.

Systematic review registration - The study protocol was registered on PROSPERO: CRD42016053412.

For peer review only

For peer review only - http://bmjopen.bmj.com/site/about/guidelines.xhtml

Strengths and limitations of this study

- This mixed methods systematic review provides the opportunity for a broadened and deeper understanding of the qualitative and quantitative literature on the experiences of unpaid caregivers' providing care to people with heart failure, chronic obstructive pulmonary disease, and coronary artery disease.
- This review provides an integration of the type and extent of caregiver's experiences and predictors of caregiver experiences.
- To maximise applicability we included studies from higher income countries only.
- Quality of evidence limited by assessment of caregiver experience at single point of time and there is need for future studies that employ longitudinal or repeated measures design.

For peer review only

For peer review only - http://bmjopen.bmj.com/site/about/guidelines.xhtml

INTRODUCTION

A caregiver is anyone providing unpaid care, to a friend or family member who is unable to care for themselves.[1] This may be emotional support; someone to talk to, or practical support; dressing wounds, mobility assistance or medication checking.[2] There are 43.5million caregivers in the United States, 2.86 million in Australia and 6.5million in the UK.[3] Between 2001 to 2011 the number of unpaid caregivers in the UK grew at a faster rate than population growth.[4] The annual value of unpaid care provided to an individual with a chronic illness is estimated to be £132billion.[5]

Focus groups examining a caregivers' life conducted by "The Institute of Public Care" (2017), based at Oxford Brookes University; described caregivers as the 'Skilled Helper' performing a series of roles.[6] Seltzer & Wailing (2000) describe a dynamic process of transitions to being a caregiver.[7] These transitions comprise participating in the role before identifying as a caregiver, acceptance of the role, engaging in it with awareness and sometimes moving beyond the caregiving role when the patient moving to paid care settings or bereavement occurs. This process is not linear and people move through the different transitions at varying rates. Acknowledging this, it is imperative for caregivers to receive a caregiver needs assessment as legally stipulated by the 2014 Care Act. [8] Additionally, the National Institute for Health and Care Excellence (NICE) clinical guidelines for heart failure (HF) (CG108)[9] and chronic obstructive pulmonary disease (COPD) (CG101)[10] both recommend that family members or caregivers are provided with support and included in discussions about care.

Cardio-pulmonary disease is a primary cause of illness. Cardiovascular disease is responsible for 45% deaths in Europe[11] and 1 in 4 deaths in the United States.[12] By 2020, COPD is projected to be in the global top five of diagnoses causing years lost through early mortality or disability-adjusted life years.[13]Caregivers of HF patients have a multitude of unmet needs due to fluctuations in the trajectory of HF.[14] COPD has frequent unplanned hospital admissions and a high morbidity rate.[15] Caregivers experience depressed mood, greater anxiety and increased subjective burden when their support needs are not met.[16,17] The unpredictability of HF and COPD leads to caregivers constantly adjusting their role, creating a need to continuously re-asses what caregiver needs are.[18,19] Spousal caregivers of myocardial infarct patients experience increased levels of stress, lifestyle impact and emotional distress.[20] Caring for coronary artery bypass graft patients in tasks such as monitoring and provision of emotional support increased caregiver burden to a level described as moderate.[21] COPD and cardiovascular disease are both increasing in prevalence and frequently co-exist. [22, 23] We know of no systematic review that synthesises quantitative and qualitative studies to combine caregivers' experiences of people with HF, COPD or coronary artery disease (CAD).

For peer review only - http://bmjopen.bmj.com/site/about/guidelines.xhtml

Using a mixed methods systematic review methodology including both qualitative and quantitative literature, this study aims to understand the experiences of adult caregivers when supporting people with HF, COPD or CAD.

METHODS

We conducted and reported this systematic review in accordance with the PRISMA (Preferred Reporting Items for Systematic Reviews and Meta-Analyses) statement.[24]

Patient and Public Involvement

There was no patient and or public involvement in this systematic review.

Study design

This study employed a mixed methods systematic review assessing both qualitative and quantitative studies.[25] The rationale for using a mixed methods review approach was multifaceted. Firstly, to gain a qualitative assessment of the type and extent of caregiver's experiences. Second to assess the quantitative predictors of caregiver's experiences. Thirdly, to develop a holistic perspective of what caregiver's experience. Finally, we wanted to assess the degree of convergence between qualitative and quantitative experiences.

Search Strategy

Our search strategy was designed in conjunction with a Health Services Librarian and Information Specialists. Search terms included condition specific terms, i.e., "heart failure", "COPD" and "coronary artery disease", caregiver specific, plus experience related terms, "experience", "quality of life" "activities of daily living", "occupational engagement", "time use", "self-efficacy", "coping strategies", "leisure activity", "information exchange" and "caregiver expectation"". (See supplementary table 1 for complete list of search terms.) Databases searched included: Medline Ebsco, PsycInfo, CINAHL Plus with Full Text, Embase, Web of Science, Ethos: The British Library and ProQuest. Grey literature was identified using Global Dissertations and Theses and Applied Sciences Index and hand searches and citation checking of included references. To ensure the contemporary nature of the evidence considered, the search time frame was January 1990 to August 2017. A single researcher (MN) initially screened titles and abstracts. Selection of full papers was performed by two researchers (MN and either JW or RST) and cross checked with the eligibility criteria.

Study Selection

Studies were included if they addressed 'caregiver experience' which was defined as encompassing the daily activities of caregivers and the impact of these activities on their lives. These were English

For peer review only - http://bmjopen.bmj.com/site/about/guidelines.xhtml

language studies involving unpaid adult caregivers (>18 years), providing care for patients with HF, COPD or CAD living in the community and not residential settings with paid care staff. Qualitative, quantitative and grey literature studies were all included in the search strategy. Conference papers were excluded. Outcomes of interest included psychological and physical outcomes reported, occupational engagement and routine. As we sought to inform the practice of UK and other high income countries we excluded studies undertaken in low and middle income countries. [26]

Data extraction

Data extracted from retained studies included: study design, sample and recruitment, study description, method, findings, discussion and author's conclusions and limitations. Caregiver quotes were extracted from qualitative studies. For quantitative studies, data extraction also included details of attrition and data analysis.

Study quality assessment

Qualitative studies were appraised utilising the Critical Appraisal (CASP) Tool.[27] In absence of an existing quality tool that could be used to appraise quantitative studies addressing the specific question of this study, a quality assessment tool was developed by the research team based on what were deemed to be the appropriate core biases i.e. (1) Was the study design longitudinal (score of 1) or cross sectional (score of 0). (2) How was the sample recruited? Purposive (score of 1) or convenience (score of 0). (3) Was the level of attrition/response rate acceptable? Attrition of 20%/lower or response rate of 80% or above (score of 1) or attrition of more than 20% or response rate less than 80% (score of 0). (4) Was a validated quantitative outcome(s) used? Validated (score of 1), non-validated (score of 0). (5) Were the methods of data analysis appropriate? Multivariate (score of 1) or univariate (score of 0). Based on their quality assessment, scores were totalled and studies were ranked: 1 or 2 "low quality", 3 "medium quality" and 4 or 5 "high quality". Data extraction and quality appraisal was first conducted by a single researcher (MN) and checked by one of two researchers (JW or RST).

Data analysis and synthesis

The methodology of mixed methods data synthesis is an emerging one and no single approach has yet been universally accepted.[28] In this study a results based convergent design was chosen.[29,30] This requires transformation of one method into another. Due to the heterogeneity of the quantitative methods a meta-analysis was not appropriate. Instead, applying a narrative profile formation, quantitative data was converted into qualitative data.[31] Extracted data from quantitative and qualitative studies were imported into separate spreadsheets. A meta-ethnographic approach was utilised to synthesise qualitative studies.[32] A narrative formation approach[33] was utilised to

For peer review only - http://bmjopen.bmj.com/site/about/guidelines.xhtml

synthesise the quantitative data into a qualitative data set. Narrative formation is a verbal description via the use of profiles of each of the studies. The five profiles are modal, average, holistic, comparative and normative.[33] Table 1 provides an example of this approach. This resulted in two qualitative data sets[34] from which concepts emerged. A mapping table was completed in order to provide an audit trail of how the overall concepts across all papers were derived (see supplementary file 2a, 2b and 2c). Initial synthesis was conducted by a single researcher (MN) and corroborated by two experienced researchers in quantitative (RST) and qualitative (JW) research.

Table 1

Narrative Formation

Profile	Explanation of Profile	Author, Year	Modal narrative extracted from study	Emerging from study	Concept
**Modal**	This is a narrative description of the group being studied	Chung, 2016	102 dyads, predominantly spousal caregivers. Group is caregivers of patients with HF. Comparing depressed and non-depressed HF pts and the	This study considers whether caregiver experiences are different for caregivers when caring for depressed or non-depressed patients<br><br>Spouse as a caregiver, Impact of depression on the role of caregiver. How caregivers experience their role	Role (Role as a spousal caregiver)
**Average**	This is a detailed narrative description based on the mean (average) attributes of the individuals/ situations being studied		Caregiver Mean age 56.7, 78% female, white (94%). 41% of pts NYHA III-IV 42% caregivers reported severe	Impact of patient mental health on caregiver<br><br>Lifestyles are changed and restricted<br><br>Time spent caregiving	Mental Health Routine Lifestyle

For peer review only - http://bmjopen.bmj.com/site/about/guidelines.xhtml

			burden (the Zarit Burden Interview (ZBI)≥17)"<br><br>"Caregivers of patients with depressive symptoms had a higher level of burden (25±13 vs 13.5±12 on the ZBI; p<0.001), spent more time caregiving (37±12 vs 30±11 on the Oberst Caregiving Burden Scale; p=0.004), and reported worse mental quality of life (46±10 vs 51±10 on the SF-12v2; p=0.026) than those of patients without depressive symptoms."	resulted in higher burden being experienced<br><br>Patient mental health has an impact on caregiver subjective experience. The reelationship between patient and caregiver	
**Comparative**	This is a description comparing studies and comparing individuals being studied.		Caregivers who provided care for depressed patients reported higher burden related to social life limitations, poor	Patient illness severity impacts on caregiver<br><br>Life changes negatively impacted on caregivers relationships<br><br>Depressive symptoms of	Caregiver's Relationships

For peer review only - http://bmjopen.bmj.com/site/about/guidelines.xhtml

			perceived control, stress of family obligations, and patients' dependency than those caring for non-depressed patients<br><br>Caregivers of depressed patients reported more difficulty providing emotional support and managing behavioural problems	patients are associated with poor outcomes of caregivers<br><br>Caregiver's subjective and objective response to the patient's illness severity	
**Normative**	A comparison of the study individuals with the general population	Depression in HF is estimated to be 4-5% higher than in the "normal population"[35]	27% of patients in this study scored 14 or higher on Becks Depression Index (a score of 14 or higher is clinically significant for depression)<br><br>Family members caring for HF patients with depressive symptoms had significantly higher levels of caregiving burden and worse quality of life	Greater impact on caregivers lives when patients were depressed	Mental health<br><br>Role of caregivers

For peer review only

For peer review only - http://bmjopen.bmj.com/site/about/guidelines.xhtml

			compared to those caring for patients without depressive symptoms.<br><br>"Most difficult task for both sets of caregivers – providing emotional support (M=3.3, SD=1.2)"		
**Holistic (also called inferential or summative)**	A description of the overall perception of the investigator			Female, white caregivers, experienced greater levels of burden, loss of roles and greater distress when patients were depressed. This could be due to the increased need for practical and emotional support, feeling they need to be constantly present	

For peer review only

For peer review only - http://bmjopen.bmj.com/site/about/guidelines.xhtml

RESULTS

Study selection

Study selection process is summarised in a PRISMA Flow diagram shown in Figure 1. Following removal of duplicates the search strategy yielded a total of 8,026 title and abstracts. Of these 242 full papers were reviewed, of which 57 papers (54 studies) were included for synthesis. A detailed summary of included studies is provided in Table 2.

For peer review only

For peer review only - http://bmjopen.bmj.com/site/about/guidelines.xhtml

PRISMA Flow Diagram Figure 1

For peer review only

For peer review only - http://bmjopen.bmj.com/site/about/guidelines.xhtml

Table 2

Summary of included studies

First Author/Year	Diagnosis	Aims (as stated by authors)	Methods	Country	Data Collection Sampling	Participants Caregiver (Time caring)	Mean Age (Caregiver /Recipient)
Ågren (2010)	HF (NYHA II-IV)	Describe the levels and identify independent predictors of cg. burden. in partners of pts with HF.	QUANT (Correlational)	SWEDEN	Cross Sectional Purposive	135 (101F, 34M) (N/S)	69/71
Al-Rawashdeh (2017)	HF (NYHA I-IV)	To examine whether individuals' sleep disturbance predicted their own & their partners' QoL in HF	QUANT	USA	Cross Sectional Purposive	78 Dyads (58F, 20M) (N/S)	62.2./59.5
Andersen (2015)	HF	Obtain knowledge on experiences and views and the desire for knowledge of family cgs of HF pts, their competence and support required	QUAL	NORWAY	Interviews Convenience	19 (17F, 2M) (N/S)	63/N/ S
Badr (2017)	COPD	Individual-level predictors of pt and and cgs depression in COPD as well as how dyad members effect each other's depression	QUANT	USA	Cohort Study Purposive	89 (68F, 21M) (N/S)	54.8/67

For peer review only - http://bmjopen.bmj.com/site/about/guidelines.xhtml

Summary of included studies

First Author/Year	Diagnosis	Aims (as stated by authors)	Methods	Country	Data Collection Sampling	Participants Caregiver (Time caring)	Mean Age (Caregiver /Recipient)
Bakas (2006)	HF (NYHA II-IV)	Examine relationships among age, perceived control over managing HF perceived difficulty with tasks, perceived outcomes, and perceived mental and general health among cgs of persons with HF	QUANT (Descriptive Correlational)	USA	Cross Sectional Convenience	21 (20F, 1M) (N/S)	59.6/62.7
Baker (2010)	HF (LVAD in situ)	To describe experiences of cgs of pts who received LVAD therapy as a bridge to transplantation	QUAL (Phenomenological) (Descriptive)	USA	Interviews Convenience	6 (5F, 1M) (26-372 days)	51/N/S
Bove (2016)	COPD (GOLD C&D)	Explore how spouses of pts with severe COPD experience their role	QUAL	DENMARK	Focus Groups Purposive	22(13F, 9M) (N/S)	69.4/N/S
Burke (2014)	HF (NYHA II-IV)	Understand what roles cgs perceive and desire for themselves, and to compare and contrast these roles with those they perceive to be desired by the health care system	QUAL (Inductive)	USA	Interviews Purposive	20 (18F, 1M, 1N/S) (<1 - > 8 hours per week)	59/64

For peer review only - http://bmjopen.bmj.com/site/about/guidelines.xhtml

Summary of included studies

First Author/Year	Diagnosis	Aims (as stated by authors)	Methods	Country	Data Collection Sampling	Participants Caregiver (Time caring)	Mean Age (Caregiver /Recipient)
Chung (2016)	HF (NYHA II-IV)	Examine differences in cg outcomes between cgs who care for HF pts with and without depressive symptoms	QUANT	USA	Cross Sectional Convenience	102 Dyads (79F, 23M) (N/S)	56.7/61.4
Clark (2008)	HF (NYHA II – IV)	To examine the complexity of caregiving for pts with HF	QUAL	SCOTLAND	Interviews Convenience	30 (23F, 7M) (N/S)	68F, 67M/N/S
Cossette (1993)	COPD (GOLD III-V)	Examine relationship between type, number and disturbance of caring tasks and impact on mental health of cg's Examine influence of social support	QUANT	CANADA	Cross Sectional Convenience	89 (F) (Mean = 13yrs)	65/68.6
Evangelista (2002)	HF (NYHA I – IV)	Describe emotional wellbeing of HF pts and cgs Identify factors associated with emotional wellbeing of pts. Determine gender differences in emotional wellbeing of pts and cgs	QUANT (Descriptive) Correlational)	USA	Cross Sectional Convenience	103 Dyads (73F, 30M) (N/S)	59.4/57.6

For peer review only - http://bmjopen.bmj.com/site/about/guidelines.xhtml

Summary of included studies

First Author/Year	Diagnosis	Aims (as stated by authors)	Methods	Country	Data Collection Sampling	Participants Caregiver (Time caring)	Mean Age (Caregiver /Recipient)
Figueiredo (2013)	COPD (GOLD I-IV)	Examine coping strategies of family cg's of pts with early and advanced COPD and how those relate to their subjective health	QUANT (Correlational)	PORTUGAL	Cross Sectional Convenience	158 (120F, 38M) (> 4 years)	58.4(early COPD) 60.8 (Adv. COPD) /69.4
Figueiredo (2014)	COPD (GOLD I-IV)	To analyse subjective burden of family cg's of early and advanced COPD pts and its predictor variables	QUANT (Correlational)	PORTUGAL	Cross Sectional Convenience	167 (125F, 42M) (> 4 years)	58.3(early COPD) 60.5 (Adv. COPD) /69.3
Figueiredo (2015)	COPD (Mod – Severe)	Obtain knowledge on experience of husbands &sons providing care to a family member	QUAL	PORTUGAL	Interviews Purposive	12 (M) (> 4 years)	70.9(Husbands) 43.4 (Sons)/72.1
Grigorovich (2017)	HF (NYHA II-IV)	To examine changes in cg's wellbeing over time. Identify pt and cg factors associated with positive and negative outcomes	QUANT (Repeated measures)	CANADA	Longitudinal Convenience	50 (31F, 19M) (Mean = 18 months)	58/61.6

For peer review only - http://bmjopen.bmj.com/site/about/guidelines.xhtml

Summary of included studies

First Author/Year	Diagnosis	Aims (as stated by authors)	Methods	Country	Data Collection Sampling	Participants Caregiver (Time caring)	Mean Age (Caregiver /Recipient)
*Halm et al. (2006)<br>*Halm et al. (2007)	CAD	To determine cg burden after CABG surgery	QUANT	USA	Cross Sectional<br>Convenience	166(136F, 30M)<br>(≤12 months)	64.7/66.8
*Halm (2016)<br>*Halm (2017)	CAD	To describe the concerns needs, strategies and advice of CABG cg's during the first 3 months post- surgery<br>To explore cg burden by age and gender	QUAL	USA	Interviews<br>Purposive	32(16F, 16M)<br>(N/S)	60.6 (M<70) /60.1<br>61.5 (F<70) /62.5<br>75.9(M>70) /74.4<br>73.6(F>70) /77.6
Hess (2009)	HF	To examine the association between cg literacy and medication administration	QUANT<br>(Correlational)	USA	Cross Sectional<br>Convenience	5 (F)<br>(N/S)	65/72.8
Hooley (2005)	HF<br>(NYHA III or IV)	To explore if greater cg burden is associated with increasing disease burden and depressive symptoms in pts and cg	QUANT	CANADA	Cohort Study<br>Convenience	50<br>(40F, 10M)<br>(N/S)	61/72

For peer review only - http://bmjopen.bmj.com/site/about/guidelines.xhtml

Summary of included studies

First Author/Year	Diagnosis	Aims (as stated by authors)	Methods	Country	Data Collection Sampling	Participants Caregiver (Time caring)	Mean Age (Caregiver /Recipient)
Hwang (2011)	HF (NYHA I – IV)	To identify factors associated with the impact of caregiving	QUANT	USA	Cross Sectional Convenience	76 Dyads (54F, 22M) (Mean = 53.4 months)	53.4/53.8
Hynes (2012)	COPD	To explore the experiences of cg's providing care to a family member with COPD	QUAL (Phenomenological)	IRELAND	Interviews Convenience	11(9F, 2M) (1 – 15 years)	N/S/N/S
Imes (2011)	HF (NYHA III-IV)	To describe cg's experience of living with HF	QUAL (Descriptive)	USA	Interviews Convenience	14(11F, 3M) (N/S)	64.8/68
Karmilovich (1994)	HF (NYHA III OR IV)	To examine cg demands and components of caring. Assess stress and correlation with cg burden. Assess relationship of	QUANT (Correlational)	USA	Descriptive survey Purposive	41 (30F, 11M) (N/S)	56.7/N/S
Kitko (2010)	HF	To gain a deeper understanding the type of work in spousal caregiving	QUAL	USA	Interviews Convenience	20(14F, 6M) (2 months – 9 years)	67/70

For peer review only - http://bmjopen.bmj.com/site/about/guidelines.xhtml

Summary of included studies

First Author/Year	Diagnosis	Aims (as stated by authors)	Methods	Country	Data Collection Sampling	Participants Caregiver (Time caring)	Mean Age (Caregiver /Recipient)
Kneeshaw (1999)	CAD	To examine cg mutuality and preparedness for caring post CABG surgery.	QUANT	USA	Longitudinal Convenience	49 (32F, 17M) (N/S)	50.1/72.6
Liljeroos (2014)	HF	To understand perceived caring needs in dyads and understand areas of support for cgs	QUAL	SWEDEN	Focus Groups Convenience	19Dyads (7F, 12M) (N/S)	70/72
Lindqvist (2013)	COPD (Mild to severe)	To describe conceptions of daily life for women caring for men with COPD	QUAL (Phenomenological)	SWEDEN	Interviews Purposive	21(F) (N/S)	72/N/S
Loftus (2004)	HF (NYHA II-IV)	Investigate outcomes of caregiving in late stage HF	QUANT (Correlational)	UK	Longitudinal Convenience	53 (41F, 12M) (6.66 hours per day)	66.7/76.3
Lum (2014)	HF (NYHA II-IV)	Measure of relationship quality and cg benefit, burden and depressive	QUANT (Correlational)	USA	Cross Sectional Purposive	19 (7F, 12M) (<1 - >8 hours per week)	59/69

For peer review only - http://bmjopen.bmj.com/site/about/guidelines.xhtml

Summary of included studies

First Author/Year	Diagnosis	Aims (as stated by authors)	Methods	Country	Data Collection Sampling	Participants Caregiver (Time caring)	Mean Age (Caregiver /Recipient)
Luttik (2007)	HF	Experiences and needs of cgs wellbeing factors	QUAL	NETHERLANDS	Interviews Convenience	13(10F, 3M) (N/S)	66/88.6
Luttik (2009)	HF (NYHA II-IV)	Investigate QOL in cg's of HF pts versus QOL in people with healthy partners	QUANT	NETHERLANDS	Cross Sectional Purposive	303 (N/S)	67/6
Marcuccilli (2011)	HF –LVAD in situ	Explore life adjustments of cg's caring for long term LVAD pts.	QUAL (Phenomenological)	USA	Interviews Convenience	5 (5F) (N/S)	56.6/N/S
Marcuccilli (2014)	HF-LVAD as DT	Explore experience of caring for family member with	QUAL (Phenomenological)	USA	Interviews Purposive	7(6F, 1M) (18 – 24 hours per day)	65/N/S
Miravitlles (2015)	COPD	Analyse burden of cg's	QUANT	SPAIN	Survey Representative	22,795 (Mean = 12.7 hours daily, severe COPD)	56.5/72
Nakken (2017)	COPD	Investigate differences in male and female cg's and their perception of pts symptoms	QUANT (Correlational)	NETHERLANDS	Cross Sectional Convenience	188 Dyads (103F, 85M) (N/S)	65.4/63.3F 65.1/68.7M

For peer review only - http://bmjopen.bmj.com/site/about/guidelines.xhtml

Summary of included studies

First Author/Year	Diagnosis	Aims (as stated by authors)	Methods	Country	Data Collection Sampling	Participants Caregiver (Time caring)	Mean Age (Caregiver /Recipient)
Näsström (2017)	HF	Cg's participation & perspective of home care services	MIXED METHOD	SWEDEN	Interviews Purposive	15 (11F, 4M) (N/S)	77/N/S
Park (2013)	CAD	Difficulty and demands of cg tasks for older cg's of CABG pt.'s	QUANT	USA	Cross Sectional Convenience	35(29F, 6M) (Mean = 19days)	60/N/S
Pattenden (2007)	HF	Explore how pts and cgs cope with daily life with HF	QUAL	UK	Interviews Purposive	20(18F, 2M) (N/S)	67.8/N/S
Pi-Ming Yeh (2012)	HF	Explore burden on family cg's of older HF pts	QUANT (Correlational)	USA	Cross Sectional Purposive	50 (35F, 15M) (< 6 months - > 1 year)	60.3/77.6
Pressler (2013)	HF (NYHA I-IV)	Examine changes in cg burden and HRQOL Determine different perceptions between cg's of low HF pts and cgs of high HF pts Estimate time spent on cg tasks	QUANT (Repeated Measures)	USA	Longitudinal Convenience	65(48F, 17M) (Mean = 9.3 years)	59.7/69

For peer review only - http://bmjopen.bmj.com/site/about/guidelines.xhtml

Summary of included studies

First Author/Year	Diagnosis	Aims (as stated by authors)	Methods	Country	Data Collection Sampling	Participants Caregiver (Time caring)	Mean Age (Caregiver /Recipient)
Riegner (1996)	COPD	To understand QOL and its association with role strain, humour and support in cg's and pt's.	QUANT (Correlational)	USA	Cross Sectional Convenience	83(Dyads) (50F, 33M) (N/S)	63.2/65.6
Rolley (2010)	CAD	Describe experience of cgs of pt.'s undergoing percuatenous coronary intervention	QUAL	AUSTRALIA	Focus Groups Convenience	18 (F) (N/S)	N/S/N/S
*Saunders (2009)<br>*Saunders (2008)	HF (NYHA I-IV)	To determine indicators of cg HRQOL	QUANT (Correlational)	USA	Cross Sectional Purposive	50 (42 F, 8M) (Mean = 5.9 years)	58.1/77.6
Saunders (2010)	HF	Compare employed and unemployed cg's on depression and wellbeing	QUANT	USA	Cross Sectional Convenience	41 (37F, 4M) (2.9 – 6 years)	59(Unemp.)/78 52(Emp.)/77
Schwarz (2003)	HF	Evaluate support on stress for cg's.	QUANT	USA	Cross Sectional Convenience	75 (55F, 20M) (Mean = 6 years)	63/N/S
Scott (2000)	HF	HRQOL of cg's and pts receiving community-based inotropic infusions	QUANT	USA	Cross Sectional Purposive	18 (16F, 2M)	63/69.3

For peer review only

For peer review only - http://bmjopen.bmj.com/site/about/guidelines.xhtml

Summary of included studies

First Author/Year	Diagnosis	Aims (as stated by authors)	Methods	Country	Data Collection Sampling	Participants Caregiver (Time caring)	Mean Age (Caregiver /Recipient)
Spence (2008)	COPD (Adv.)	Needs and experiences of family cg's	QUAL (Descriptive)	N.IRELAND	Interviews Purposive	7 (6F, 1M) (1 – 4 years)	N/S/N/S
Strom (2015)	HF	Next of kin's experience and responsibilities when caring	QUAL	NORWAY	Interviews Convenience	19 (17F, 2M) (N/S)	Median 63/NS
Takata (2008)	COPD (Long term O2 Therapy)	Explore cg burden	QUANT	JAPAN	Cross Sectional Convenience	45 Dyads (37F, 8M) (N/S)	68/75.2
Vellone (2015)	HF (NYHA I-IV)	Examine cg self-efficacy and contribution to pt self-care	QUANT (Correlation)	ITALY	Cross Sectional Convenience	515 Dyads (270F, 245M)	56.6/75.6
Wallin (2013)	CAD	To describe cg's need for support and information and impact after a cardiac event	QUAL (Descriptive)	SWEDEN	Interviews Purposive	20 (14F, 6M) (N/S)	55/N/S
Woolfe (2007)	COPD	Identify needs of cg's and how this impacts cg wellbeing	QUANT (Descriptive)	AUSTRALIA	Cross Sectional Convenience	63(39F, 24M) (N/S)	N/S/N/S

For peer review only

For peer review only - http://bmjopen.bmj.com/site/about/guidelines.xhtml

Summary of included studies

First Author/Year	Diagnosis	Aims (as stated by authors)	Methods	Country	Data Collection Sampling	Participants Caregiver (Time caring)	Mean Age (Caregiver /Recipient)
Wingham (2015)	HF	Identify needs of cg's to inform development of a caregiver resource for use in a home- based self-management intervention	QUAL	UK	Interviews (I)<br>Focus Groups (FG)<br>Purposive	22 (16F, 6M)<br>(6 months – 8 years)	(I)67/N/S<br>(FG)62 /N/S

For peer review only - http://bmjopen.bmj.com/site/about/guidelines.xhtml

Study characteristics

Of the 54 studies, 21 were qualitative, 32 quantitative and 1 mixed methods. 34 focused on HF, 14 COPD and 6 CAD. The total number of caregiver participants was 26,453. Caregivers were primarily female (63%), with a median age of 62 years. Patient median age was 69. A summary of study characteristics is provided in Table 3.

For peer review only

For peer review only - http://bmjopen.bmj.com/site/about/guidelines.xhtml

Table 3

Study Characteristics

Summary of Study Characteristics	n = 54 Studies	
**Aetiology of patients**		
CAD n (%)	6 (11)	
HF n (%)	34 (63)	
COPD n (%)	14 (26)	
**Caregiver Participants***		**Patients ***
Median age (range)	62 (43-77)	69 (36-93)
Median % of female (range)	63% (5–270)	38% (1-229)
**Relationship between patient and caregiver (n = 26,008 caregivers)**		
Spousal/Partner n (%)	2,321 (9)	
Son/Daughter n (%)	610 (2)	
Sibling n (%)	22 (<1)	
Parent n (%)	10 (<1)	
Friend/Relative n (%)	228 (<1)	
Not stated	22,961 (88)	
**Type of study**		
Qualitative n (%)	21 (39)	
Quantitative n (%)	32 (59)	
Mixed n (%)	1 (2)	
**Study Design**		
Cross-Sectional n (%)	24 (44)	
Longitudinal n (%)	4 (7)	
Cohort n (%)	2 (4)	
Quant. (survey) n (%)	2 (4)	
Qual. (Interview/ Focus Group) n (%)	16 (31)	
Phenomenological n (%) 5 (8)		
Inductive n (%) 1 (2)		
**Continents of publication**		
Europe n (%)	22 (41)	
North America n (%)	29 (54)	
Australasia n (%)	3 (5)	
**Date of publication** **n= 57 Publications●**		

For peer review only - http://bmjopen.bmj.com/site/about/guidelines.xhtml

1990-1995	2
1996-2001	3
2002-2007	10
2008-2013	22
2014-2017	20

For peer review only

For peer review only - http://bmjopen.bmj.com/site/about/guidelines.xhtml

Quality assessment

Studies of insufficient quality were excluded, qualitative papers were appraised and only high quality qualitative studies were included.[36] A total of 21 out of 193 qualitative studies were classified as high quality. Quantitative studies were classified as follows; 3 high quality, 12 medium quality and 17 low quality. See Table 4(a) and 4(b) for quality appraisal tables. Given of the number of high quality qualitative studies and in accord with current guidelines for the synthesis of qualitative evidence, we limited inclusion to qualitative studies of high quality only.[36] In contrast, given the low number of number of high quality quantitative studies, to ensure comprehensiveness of our review, we included all quantitative studies, regardless of quality.

For peer review only

For peer review only - http://bmjopen.bmj.com/site/about/guidelines.xhtml

Table 4(a)

Quality Appraisal – Qualitative Papers

	Design	Recruitment	Data Collection	Data Analysis	Findings	Total
Andersen (2015)	1	1	1	0	1	**4 [H]**
Baker (2010)	1	0	1	1	1	**4 [H]**
Bove (2016)	1	1	1	1	1	**5 [H]**
Burke (2014)	1	1	1	1	1	**5 [H]**
Clark (2008)	1	1	1	1	0	**4 [H]**
Figueiredo (2015)	0	1	1	1	1	**4 [H]**
•Halm (2016)	1	1	1	1	1	**5 [H]**
•Halm (2017)	1	1	1	1	1	**5 [H]**
Hynes (2012)	1	1	1	0	1	**4 [H]**
Imes (2011)	1	1	1	1	1	**5 [H]**
Kitko (2010)	1	1	1	1	1	**5 [H]**
Liljeroos (2014)	1	1	1	1	1	**5 [H]**
Lindqvist (2013)	1	1	1	1	1	**5 [H]**
Luttik (2007)	1	1	1	1	1	**5 [H]**
Marcuccilli (2011)	1	0	1	1	1	**4 [H]**
Marcuccilli (2014)	1	1	1	1	1	**5 [H]**
*Näsström (2017)	1	1	1	1	1	**5 [H]**
Pattenden (2007)	1	1	1	1	0	**4 [H]**
Rolley (2010)	1	1	1	1	0	**4 [H]**
Spence (2008)	1	0	1	1	1	**4 [H]**
Strom (2015)	1	1	1	1	1	**5 [H]**
Wallin (2013)	1	0	1	1	1	**4 [H]**
Wingham (2015)	1	1	1	1	1	**5 [H]**

For peer review only - http://bmjopen.bmj.com/site/about/guidelines.xhtml

Table 4(b)

Quality Appraisal – Quantitative Papers

**First Author**	**Study design**	**Participant sampling**	**Participant attrition**	**Outcome Measures**	**Data Analysis**	Overall score
Ågren (2010)	CS	Purp [+1]	0% [+1]	Non-V	MV [+1}	**3 [M]**
Al-Rawashdeh (2017)	CS	Purp [+1]	NS	V [+1]	MV [+1]	**3 [M]**
Badr (2017)	CS	Con	15.5% [+1]	Non-V	MV [+1]	**2 [L]**
Bakas (2006)	CS	Con	NS	V [+1]	MV [+1]	**2 [L]**
Chung (2016)	CS	Con	NS	V [+1]	UV	**1 [L]**
Cossette (1993)	CS	Con	NS	V [+1]	MV [+1]	**2 [L]**
Evangelista (2002)	CS	Con	20% [+1]	V [+1]	MV [+1]	**3 [M]**
Figueiredo (2013)	CS	Con	17% [+1]	V [+1]	MV [+1]	**3 [M]**
Figueiredo (2014)	CS	Con	11% [+1]	Non-V	MV [+1]	**2 [L]**
Grigorovich (2017)	LS [+1]	Con	NS	V [+1]	MV [+1]	**3 [M]**
• Halm (2006)	CS	Con	64%	V [+1]	MV [+1]	**2 [L]**
• Halm (2007)	CS	Con	64%	V [+1]	MV [+1]	**2 [L]**
Hess (2009)	CS	Con	NS	V [+1]	MV [+1]	**2 [L]**
Hooley (2005)	CS	Con	0% [+1]	V [+1]	UV	**2 [L]**
Hwang (2011)	CS	Con	35%	V [+1]	MV [+1]	**2 [L]**
Karmilovich (1994)	CS	Purp [+1]	24%	V [+1]	MV [+1]	**3 [M]**
Kneeshaw (1999)	LS [+1]	Con	32.7%	V [+1]	MV [+1]	**3 [M]**
Loftus (2004)	LS [+1]	Con	36%	V [+1]	MV [+1]	**3 [M]**
Lum (2014)	CS	Purp [+1]	5% [+1]	V [+1]	MV [+1]	**4 [H]**
Luttik (2009)	CS	Purp [+1]	31%	Non-V	MV [+1]	**3 [M]**
Miravitlles (2015)	CS	Rand [+1]	0% [+1]	Non-V	MV [+1]	**3 [M]**
Nakken (2017)	CS	Con	58%	Non-V	MV [+1]	**1 [L]**
*Näsström (2017)	LS [+1]	Purp [+1]	7% [+1]	V [+1]	MV [+1]	**5 [H]**

For peer review only - http://bmjopen.bmj.com/site/about/guidelines.xhtml

Park (2013)	CS	Con	NS	V [+1]	UV	**1 [L]**
Pi-Ming Yeh (2012)	CS	Purp [+1]	39%	V [+1]	MV [+1]	**4 [H]**
Pressler (2013)	LS [+1]	Con	16% [+1]	V [+1]	MV [+1]	**4 [H]**
Riegner (1996)	CS	Con	71.8%	V [+1]	MV [+1]	**2 [L]**
• Saunders (2008)	CS	Purp [+1]	36%	V [+1]	MV [+1]	**3 [M]**
• Saunders (2009)	CS	Purp [+1]	36%	V [+1]	MV [+1]	**3 [M]**
Saunders (2010)	CS	Con	NS	V [+1]	UV	**1 [L]**
Schwarz (2003)	CS	Con	NS	V [+1]	MV [+1]	**2 [L]**
Scott (2000)	CS	Purp [+1]	10% [+1]	Non-V	MV [+1]	**3 [M]**
Takata (2008)	CS	Con	NS	V [+1]	UV	**1 [L]**
Vellone (2015)	CS	Con	NS	V [+1]	MV [+1]	**2 [L]**
Woolfe (2007)	CS	Con	37%	V [+1]	UV	**1 [L]**

For peer review only

For peer review only - http://bmjopen.bmj.com/site/about/guidelines.xhtml

Findings

Six concepts relating to caregiver experience were identified, (1) mental health, (2) caregiver role, (3) lifestyle change, (4) support for caregivers, (5) knowledge and (6) relationships. Four additional concepts were identified from qualitative papers only (6) expert by experience, (7) vigilance, (8) time and (9) shared-care (See Figure 2). The concepts are reflected in caregiver quotes in Table 5.

For peer review only

For peer review only - http://bmjopen.bmj.com/site/about/guidelines.xhtml

Figure 2

Conceptual Experiences of Caregivers

For peer review only

For peer review only - http://bmjopen.bmj.com/site/about/guidelines.xhtml

Table 5

Illustrative Quotes of caregiver experience – by concept

**Mental Health**	***"The mental strain is difficult. I feel so trapped"***<br><br>***""You fall into a huge hole, then the world gets so tiny, it all gets so narrow that it is almost unbearable"***<br><br>***"I feel like sleeping beauty. The hawthorn hedge has closed around me, and I cannot do anything about it."***
**Role**	***"I can sum my role up in three words, I am a cheerleader, drill sergeant, and negotiator"***
**Lifestyle Change**	***"Our life has come down. The two of us used to go out dancing. We loved dancing and then it all stopped."***
**Knowledge**	***"I wish I had had more education on the 'what ifs'. When I was leaving the hospital nobody really said, "OK now this is what's going to happen and this is what you're going to have to do." If there would've been any kind of complications I would've been totally in the dark. I didn't know all the things I needed to know."***
**Relationships**	***"I just love him and I find that every day when I see him, what else could I do to try and make him a wee bit … better? It's very satisfying to know that he appreciates what I do and it's nice to know that you are helping someone."***<br><br>***"'It's like having another child sometimes because you are sort of responsible and I feel he is my responsibility. I feel that he is not anybody else's responsibility…"***
**Support**	***"And then I really felt alone in it all. Because everybody would call and come over and ask, how is John? Hardly anyone asked "how are you doing"?***<br><br>***"Doctors (do) not realize that 1 day your life is jut normal and then this comes and smashes everything to bits, you know- and there are so many questions."***<br><br>***"I would be lost without, our heart failure nurse, and, all the other input we've had from all the other professionals, like the podiatrist and GP. . . .. . . You can do it, but in partnership with everybody else."***

For peer review only - http://bmjopen.bmj.com/site/about/guidelines.xhtml

**Vigilance**	***"Every morning I put my ear to his chest and listen to his heart, that is how we first discovered he was in atrial fibrillation so now I do it every morning before I leave. I monitor him very closely and there are days in which I do not feel comfortable leaving for work so those days I work at home. I call everyday from work and we have our routine, if I am not aware of anything he had planned for the day, I then immediately call my neighbour to check on him"***
**Shared Care**	***"There were days I thought to myself, where are we going from here? But we mastered it together and tried to do things at his pace."***
**Time**	***"At first it was overwhelming. I didn't think I could do it. When they first told me I was like, "I can't do that," you know. And then they explained to me, like, yes you can. It's like getting a new baby. You know, you learn how to take care of them step by step and then it's just part of the routine. And that's really the way it was"***
**Expert by Experience**	***"It's so frustrating when she goes into hospital and the nurses and the doctors say it's her condition, you know. I'm like I'm with her twenty-four hours a day, I know how breathless she is without infection and I know how breathless she is with an infection there's a major difference"***<br><br>***"I see him every day, they are just little subtle changes, they are not showing up in the numbers the doctors are concerned with but I see it"***

1. Mental Health

Twenty-five quantitative, [37-63] twenty qualitative [64-84] and one mixed method[85] study addressed mental health. This encompassed depression and burden. Caregivers described an internal and external conflict of emotions, recognising a psychological change within themselves and the care recipient. Maintaining hope and positivity, versus managing worries, fears and anxieties was predominant.[64-83] Pressler's (2013) HF study; identified caregivers had moderately poor health at baseline and at 8 months but they had fewer depressive symptoms over time.[55] Burden arose due to greater responsibilities.[66, 69,74,82,83] Pi-Ming Yeh (2012), noted the quality of relationship and lack of family support significantly predicted greater family caregiver burden.[54] Näsström (2017) reported, caregiver burden was concerned with the future and their fears of potential demands.[85] Those with greater resiliency appeared to adjust and cope better with the illness trajectory.[65, 66, 77, 78] Caregivers

For peer review only - http://bmjopen.bmj.com/site/about/guidelines.xhtml

described mental adjustment after an acute event.[78] Living through an acute event was long lasting and some experienced post traumatic symptoms.[81]

2. Caregiver Role

This is addressed in eighteen qualitative [65-73,75,76,78-84,86] and fourteen quantitative studies.[40-42, 50-52, 55, 58, 60, 61, 87, 88, 89,90] Caregiver role is complex and requires much co-ordination. [75, 82, 84] Caregivers describe significant role change such as increasing domestic tasks. [64, 67, 70, 72,77 -80, 83] Role loss is prevalent [65, 66, 71] and caregivers need to reframe their identity.[73, 81] Societal expectation regarding the relationship and gender, influences caregivers adjusting to their roles.[66, 68, 69, 75, 80] Caring can be positive and rewarding. Caregivers learn about themselves and strengths they have. [66, 76-78, 81, 82, 84] Pressler (2013), describes the tasks involved; domestic, emotional support, managing dietary needs and transport.[55] Pressler also reports caregivers of persons with greater HF symptoms experience more difficulty with their role.[55]

3. Lifestyle Changes

Fourteen quantitative [38-41, 44, 46, 53,55, 60, 61, 63, 88,89, 91] and twenty-one qualitative[64-84,86] studies addressed lifestyle changes. Caregivers experienced leisure, social and work related problems. [40,41,91] Caring interrupted and eliminated tasks from their routine.[40, 41, 60] Contrastingly, Pressler (2013) reported caregivers perceptions of how their lives changed as a result of caregiving was neutral and improved from baseline to 4 and 8 months.[55] Caregivers became adaptable in their new role.[73,81,86] There was less personal time for leisure and hobbies either alone or with the care recipient[68-71,77,78,83,84] Caregivers described daily "ups & downs" and had to adjust their routines dependent on the presentation of the care recipient. [64,65,67,72,74,76,79,80,82,84]

4. Support for caregivers

Fifteen quantitative[42, 46-48, 50, 52-54,56,57,59-61, 63,88,90], twenty-one qualitative[64-84,86] and one mixed method[85] study examine support. This includes healthcare, family and social support. The weight of perceived external expectations, the necessity of being pro-active in obtaining support and maintaining a social role was described across all diagnoses.[41,46,47,49-51, 54-58, 62, 90] Pi-Ming Yeh (2012) identified lack of family support as a significant issue.[54] Caregivers felt abandoned by healthcare teams. After hospital discharge they had to provide care without advice or medical support.[67, 73, 79] Positive interactions were reported. Namely access to healthcare professionals via telephone or home support.[64, 65, 78, 85]

For peer review only - http://bmjopen.bmj.com/site/about/guidelines.xhtml

5. Knowledge

This was addressed in seventeen qualitative[64,66-76,78,80,81,83,84,86] five quantitative[51,61-63,88] and one mixed method[85] study. This describes caregivers understanding of the diagnosis and need for knowledge throughout the duration of illness. [64,68,71,76,84,85,86] Caregivers report information from health professionals was often inadequate. [72, 74, 75] Timing and format of information was significant. Caregivers received information verbally or by leaflets in hospital but describe being left alone to provide care in the long term [66, 69,70,80,83] Caregivers had difficulty understanding how to navigate the care system. [73, 81] They had to make decisions without full knowledge of the consequences of their decision making, particularly during acute exacerbations.[66] The quantitative element of Näsström's (2017) mixed methods study correlated with qualitative studies; receipt of sufficient information was central to managing HF and was associated with better perceived health of caregivers.[85]

6. Relationships

Twenty qualitative,[65-84,86] twenty-two quantitative[37-39, 43-45,47-53,55-58,62,88,91,92,93] and one mixed method[85] study examined relationships. In HF studies caring for individuals with more symptoms resulted in poorer perceived experiences.[55,92] Higher relationship quality resulted in less burden and more benefit from the relationship. The relationship prior to diagnosis influenced the current relationship. Perspective of the relationship was either a sense of duty [66,75,81,82,85] or this was a valuable second chance.[67,76,83,84] Caregivers reported difficulty communicating about the condition leading to isolation, stress and conflict between caregiver and care recipient.[72,74] The relationship requires negotiation.[70,86] Caregivers prioritised the care recipient over their own needs. [65, 73,75,78,83]

7. Expert by Experience

Twelve qualitative studies[66-71,73,76,77,81,82,84,86] addressed this concept. Caregivers learnt new skills. They became "experts by experience" discovering through "doing" and observing health professionals.[67,69,84] They developed "proto-professional skills"; in medication administration [66,81,86] judging care recipients level of functioning [80] and decision making in times of exacerbations.[71] Caregivers observed the nuances of change in the care recipient often not perceived by healthcare teams or other family members such as skin colour or irritability.[73,76,82]

8. Vigilance

Vigilance was recurring in caregivers' narrative across all diagnoses and was present in nineteen qualitative studies.[65-81,82,84,86] Caregivers were always on the alert observing the care recipient.

For peer review only - http://bmjopen.bmj.com/site/about/guidelines.xhtml

[67,68,71,73-75,78-80,82] They lay awake at night listening for their partners' breath.[70,72,76,86] This impacted on caregivers' health creating constant fatigue, worry and stress.[66,81] Caregivers recognised that the need for vigilance came from themselves and their insecurities. [65, 77, 84]

9. Time

Time explores how caregivers adjusted to living with the illness and was present in fifteen qualitative studies.[66,68-78,81,83,84,86] Caregivers adapted to a new life, referring to "then", how life was and "now" their current life.[70, 71,76,77,84] The duration of caregiving and severity of illness influenced caregiver's ability to adjust.[67, 74, 77] Caregivers lived day by day [84] and viewed the future, with hope or uncertainty about what lay ahead. [66, 71,73,80,83]

10. Shared Care

Shared care was present in sixteen qualitative studies.[64-67,69-77,81,82,84,86] This demonstrates caregiver and care recipient working together managing the illness. Jointly administering medication [69, 82] and attending appointments.[74] The presence of illness was a process they adjusted to together. [7, 81] Caregivers referred to themselves and the care recipient as "we", when discussing dealing with the illness.[64,72,76] The mutual perspective between caregiver and care recipient served to isolate them from the world, the illness was *"taking a life of its own…it's like this third person"*, (Hynes, 2012, p. 1071)

There were differences in caregiver experience for each of the diagnoses and these are discussed below.

Heart Failure

HF caregivers experienced an "ebb and flow" in caring, an underlying worry, fear and anxiety which at times of change or illness heightened.[50,52, 60,72,78, 82, 84,86,93]Lifestyle changes were long lasting and sustained.[40, 60, 65, 72, 78,82, 84,86,93] Obtaining knowledge was necessary throughout all stages of the illness. [51, 64, 67, 74, 84, 86, 93]Sourcing information and communication with health professionals was often difficult. [64, 67, 72, 86, 93]In spousal relationships they predominantly viewed the care recipient as another child or as a "duty." [51, 52, 65, 67, 72, 74, 78, 85]

Chronic Obstructive Pulmonary Disease

COPD caregivers experienced a prolonged impact on their mental health similar to HF caregivers. [42, 45, 53,61, 66,71,81] Severity of illness was influential on their experience of burden.[39,44,45,61] Role change was

For peer review only - http://bmjopen.bmj.com/site/about/guidelines.xhtml

long lasting and profound for many.[71,81,66,68] They expressed concerns with perceived lack of knowledge.[63,66,71,75,81] During exacerbations COPD caregivers experienced anxiety and fear of their loved one dying.[66,68,71,75] COPD caregivers highlighted the loss of social roles while trying to maintain their loved ones dignity.[66,75,71,81,90,91] The coughing and spitting associated with COPD often left the care recipient embarrassed.[68,66,81] The caregiver tried to avoid situations where this would happen. The dynamics of spousal relationships changed, caregivers described losing the intimate love they had for their partner.[66,71,75, 81]

Coronary Artery Disease

Caregivers of CAD patients experienced intense role change on discharge from hospital and in the acute phase of illness.[48,69,80,83,88,89] They initially engaged with a high volume of tasks which reduced over time.[47,48,69,83,80,88,89] CAD caregivers experienced post-traumatic type symptoms if they witnessed the recipient experience an acute event.[80,83] Caregivers described being unable to talk about this and reliving the event in their heads. Anxiety did ease over time for many.[80,83] Caregivers felt unprepared at hospital discharge and highlighted not realising how much their routine would be disrupted.[69, 80,83] Caregivers reported viewing the experience as a second chance and had a renewed sense of love and appreciation for the relationship.[80,83]

DISCUSSION

This mixed methods systematic review demonstrates the similarities and differences in caregiver experiences across three common cardio-respiratory conditions. It highlighted the differences in experiences obtained from qualitative and quantitative research. Commonly occurring experiences included the exacerbation of caregiver physical and mental health due to the role. This correlates with Kang et al.'s (2011) systematic review of HF caregivers identifying that caregiving resulted in a multitude of changes in caregiver's lives regardless of age, gender and ethnicity.[94] Addressing both patient and caregiver needs in order to maintain wellbeing for both is important[19] and recognises the value of "shared-care" between patient and caregiver. The prevalence of mental health needs in this review demonstrates the need for psychosocial support for caregivers. This concurs with AasbØ et al. (2016), identifying caregivers need to be in "emotional control"[95] and Wingham et al. (2017) describing the "enduring anguish" experienced by caregivers.[96] Lawton et al. (1991) attribute caregiver well-being to the commitment of the caregiver to the role and dealing with competing demands which can increase burden and negatively impact affect. Spousal caregivers may be more ready to accept the role of caregiving than adult children who may view it as an imposition on an already established lifestyle.[97] In this review societal expectations had an impact on how caregivers

For peer review only - http://bmjopen.bmj.com/site/about/guidelines.xhtml

adjust to their role. Additionally, the quality of the relationship prior to becoming a caregiver had an influence on the caregiver subjective experience of burden.

Caregivers had predominantly negative experiences of support and described uncertainty of how to obtain this. Caregivers' need greater support and knowledge transfer to conduct their role.[98] They should be included in clinical appointments[99] to ensure they are not isolated in providing care and to allow for knowledge exchange. Caregivers in Giacomini et al.'s (2012) review of living and dying with COPD reported increasing isolation in addition to their own health issues.[18] They described pressure balancing their variety of roles; similar experiences to the caregivers in this synthesis across all diagnoses. Caregivers emphasised their need to be vigilant. This falls into five categories as defined by Feeney Mahoney's (2003) study of Alzheimer's caregivers; "watchful supervision", "protective intervening", "anticipating", "on duty" and "being there". Caregivers in this synthesis described overt vigilance, putting one's head on the chest of the recipient to check breathing or covert vigilance; observing them throughout the day.[100] Healthcare professionals must be aware of these levels of vigilance and the constant presence of them to support caregivers in their role.

Caregivers are valuable providers of care. Caregiver's needs should be assessed systematically and in a formalised manner in healthcare settings.[16] When developing collaborative models of care the inclusion of caregivers is imperative.[101]

Strengths and limitations

This review demonstrates the complexity of what it means to be a caregiver and should inform clinical care development of interventions. A mixed methods review can be contentious[102] due to the synthesis of differing paradigms. In this review it required transformation of quantitative data into qualitative data.[15,103] We aimed to present a convergence of caregiver experiences by conducting a mixed methods synthesis. However, it demonstrated four differing concepts between the two paradigms. This highlights the challenge of synthesising multiple methods. It is worth exploring how the four additional qualitative concepts could be captured quantitatively in order to inform healthcare intervention. This mixed methods synthesis is, to our knowledge the first to combine caregiver experiences in HF, COPD or CAD. It examines the differences and similarities in experiences, establishing a comprehensive assessment of the knowledge base of caregiver experiences in common cardiorespiratory conditions.

For peer review only - http://bmjopen.bmj.com/site/about/guidelines.xhtml

There are limitations to this study; both in our review methods and the nature of included studies. First, we acknowledge that the inclusion of lower quality quantitative studies may lead to risk of bias: the majority of quantitative studies utilised convenience sampling, had a high attrition and low response rate. Non-validated outcome measures were utilised in some quantitative studies with the majority of studies conducting univariate rather than multivariate analysis. However, given the limited number of high quality quantitative studies (4 studies) we believe this broader inclusion increased the generalisability of our review. Furthermore, we would note that the conclusions of this review were broadly the same with consideration of only the high quality quantitative studies. Second, studies were restricted to English language only, from high income countries and excluding caregivers of nursing home residents. This may limit the applicability of findings to other settings. Third, converting quantitative data into a qualitative data set risks the quantitative data set being oversimplified. This was managed with regular research team meetings to review each stage of this process. Fourth qualitative synthesis is an interpretation of other researcher's interpretations. To minimise individual interpretative bias a second researcher was utilised to seek confirmation of the results. Finally, included qualitative and quantitative studies were primarily cross-sectional in design, therefore considering caregiver experience only at a single point of time.

Implications for practice and future research

There are a number implications following this review. It has demonstrated there are similarities and differences in the caregiver experience in HF, COPD or CAD. The impact on caregiver's lives of those with HF and COPD appears longer lasting and more turbulent than caring for CAD patients. CAD caregiver's experience of hospital during exacerbations increased distress at discharge. This review reflects the complexity of the caregiver's role. The mixed method approach indicted differences in what is being investigated. This is important in demonstrating an understanding of the caregiver experience when dealing with complex conditions. Future research should focus on the involvement of caregivers in the design and delivery of interventions for patients with cardiopulmonary disease. In addition to targeting patients, interventions need to consider caregivers. Best practice interventions for CAD caregivers in the discharge process from hospital to home must be formalised. There appears to be a focus on the mental health of caregivers of those with HF, however, further research is needed to explore this in COPD and CAD caregivers.

Consideration needs to be given as to whether quantitative research tools to explore caregiver expertise, view of the future, experience of shared care and vigilance can be developed to capture

For peer review only - http://bmjopen.bmj.com/site/about/guidelines.xhtml

these caregiver experiences to inform the development of self-management interventions for patients and caregivers. Longitudinal studies with repeated assessment need to be conducted to assess how stable are caregiver experiences or whether they are liable to much change over time. In this review only four out of thirty-two quantitative studies examined caregiver's experiences longitudinally. Understanding whether there are caregiver changes over time will facilitate greater understanding of caregiver needs for health professionals when working with this population. Qualitative research demonstrates the nuanced expertise of the caregiving experience, it is important for researchers to consider how to reflect this in quantitative investigation so as to inform funders in order to develop and trial interventions in HF, COPD and CAD. The quality of quantitative studies in COPD and CAD were medium or low. There is a need for more empirically robust studies examining the experiences of these caregivers. Additionally, greater understanding of caregiver's experiences with this population will facilitate the development of robust evidence based guidelines for health services when working with HF, COPD and CAD.

CONCLUSIONS

This mixed methods systematic review provides a holistic synthesis of caregiver experiences of people with HF, COPD or CAD. It demonstrates there are a number of implications when an individual becomes a caregiver for those with chronic cardiopulmonary disease. Caregivers re-frame their identity and change their life course. Caregivers learn a multitude of skills and develop expertise in their new role. Their expertise is invaluable and should be acknowledged in healthcare interventions for these conditions. The quality of evidence was limited by assessment of caregiver experience at single time point. There is need for future studies that employ longitudinal designs examining the change in caregiver experience over time. Caregiving can be positive if caregivers have access to support, are well informed and part of the healthcare team. Understanding the experiences of caregivers for people with these conditions allows healthcare professionals and policy makers to reflect on our approach. Health services must consider caregivers in the design and delivery of interventions.

Contributors: MN, JW, RST: conceived and designed the review and quality appraised the included studies. MN wrote the initial draft of the manuscript. JW AND RST provided feedback and edits to manuscript drafts.

Funding

This study was supported by a University of Exeter Postgraduate Studentship Grant

Copyright/license for publication

For peer review only - http://bmjopen.bmj.com/site/about/guidelines.xhtml

"The Corresponding Author has the right to grant on behalf of all authors and does grant on behalf of all authors, a worldwide licence to the Publishers and its licensees in perpetuity, in all forms, formats and media (whether known now or created in the future), to i) publish, reproduce, distribute, display and store the Contribution, ii) translate the Contribution into other languages, create adaptations, reprints, include within collections and create summaries, extracts and/or, abstracts of the Contribution, iii) create any other derivative work(s) based on the Contribution, iv) to exploit all subsidiary rights in the Contribution, v) the inclusion of electronic links from the Contribution to third party material where-ever it may be located; and, vi) licence any third party to do any or all of the above."

Patient consent

Not applicable

Competing Interests

None declared

Data Sharing

Any requests for additional information about the study can be accessed via contacting the primary author.

Acknowledgements

We would like to thank the PenCLARHC Evidence Synthesis Team at University of Exeter Medical School**.** Katy Oaks and Catriona Organ, Librarians at Royal Cornwall Hospital Library, Knowledge Spa, Truro. Dr. Ruth Garside, Senior Lecturer in Evidence Synthesis, European Centre for Environment and Human Health for their assistance throughout the process of this review.

References:

1. Carers Trust (2015). Available from: https://carers.org/what-carer (Accessed 26th June 2017).
2. Wolff, J, Spillman, BC, Freedman, VA, Kasper, JD. A national profile of family and unpaid caregivers who assist older adults with health care activities. *JAMA Intern Med*. 2016; 176(3):372-379. Available from doi:10.1001/jamainternmed.2015.7664.
3. International Alliance of Carer Organizations. *Global carer facts*. Available from: http://www.internationalcarers.org/carer-facts/global-carer-stats/(Accessed 1st October 2017).
4. White, C. 2011 Census Analysis: Unpaid care in England and Wales, 2011 and comparison with 2001. *Office of National Statistics.* 2013. Available from: https://www.ons.gov.uk/peoplepopulationandcommunity/healthandsocialcare/healthcaresystem/articles/2011censusanalysisunpaidcareinenglandandwales2011andcomparisonwith2001/2013-02-15 (Accessed 10th October 2017).
5. Buckner, L, Yeandle, S. Valuing Carers 2015. The rising value of carers' support. *Carers UK 2015.* Available from *http://www.carersuk.org/for-professionals/policy/policy-library/valuing-carers-2015* (Accessed 7th July 2017).

For peer review only - http://bmjopen.bmj.com/site/about/guidelines.xhtml

6. The Institute of Public Care. *A Carer's Life: Implications and Considerations for Commissioning.* 2017. Available from: http://ipc.brookes.ac.uk/publications/A_carers_life.html (Accessed 30th September 2017).
7. Seltzer, MM, Wailing Li, L. The dynamics of caregiving: transitions during a three-year prospective study. *The Gerontologist.* 2000; 40(2):165–178. Available from doi.org/10.1093/geront/40.2.165.
8. The Care Act. Chapter 23. *The Care Act UK: The Stationery Office.* 2014. Available from: http://www.legislation.gov.uk/ukpga/2014/23/contents.enacted/data.htm (Accessed 11th October 2016).
9. National Institute for Health and Clinical Excellence. *Chronic heart failure in adults: management.* 2010. NICE guideline (CG108).
10. National Institute for Health and Clinical Excellence. *Chronic obstructive pulmonary disease in over 16s: diagnosis and management.* 2010. NICE guideline (CG101).
11. Wilkins, E, Wilson, L, Wickramasinghe K, Bhatnagar, P, Leal, J, Luengo-Fernandez, R, Townsend, N. European Cardiovascular Disease Statistics 2017. *European Heart Network*, Brussels. Available from: http://www.ehnheart.org/images/CVD-statistics-report-August-2017.pdf (Accessed 25th August 2017).
12. Center for Disease Control and Prevention. *Heart Disease Facts and Statistics*. Available from: www.cdc.gov (Accessed 9th November 2016).
13. Raherison, C, Girodet, PO. Epidemiology of COPD. *European Respiratory Review*. 2009;18: 213-221. Available from: doi:10.1183/09059180.00003609.
14. Nicholas Dionne-Odom, J., Hooker, S.A., Bekelman, D. et al. Heart Fail Rev (2017) 22: 543. Available from: https://doi.org/10.1007/s10741-017-9597-4.
15. Foster, F, Piggott, R, Teece, Beech, R. Patients with COPD tell their stories about living with the long-term condition: an innovative and powerful way to impact primary health care professionals' attitudes and behaviour? *Education for Primary Care*, 2016, 27:4, 314-319. Available from doi:10.1080/14739879.2016.1181699.
16. Farquhar, M. Assessing carer needs in chronic obstructive pulmonary disease. *Chronic Respiratory Disesae*. 2017, 1-10. Available from doi:10.1177/1479972317719086.
17. Mansfield, E, Bryant, J, Regan, T, Waller, A, Boyes, A, Sanson-Fisher, R. Burden and unmet needs of caregivers of chronic obstructive pulmonary disease patients: a systematic review of the volume and focus of research output. *Journal of Chronic Obstructive Pulmonary Disease. 2016;* 13(5):662-667. Available from: doi:10.3109/15412555.2016.1151488.
18. Giacomini, M, DeJean, D, Simeonov, D, Smith, A. Experiences of living and dying with COPD: a systematic review and synthesis of the qualitative empirical literature. *Ontario* Health *Technology* Assessment Series. 2012;12(13):1-47.
19. Doherty, L, Fitzsimons, D, McIlfatrick, S. Carers' needs in advanced heart failure: a systematic narrative review. European *Journal* of Cardiovascular Nursing. 2016; 15(4):203-212. Available from: doi: 10.1177/1474515115585237.
20. Stewart, M, Davidson, K, Meade, D, Hirth, A, Makrides, L. Myocardial infarction: survivors' and spouses' stress, coping, and support. *Journal of Advanced Nursing*. 2000; 31: 1351–1360. Available from: doi:10.1046/j.1365-2648.2000.01454.x
21. Stolarik A, Lindsay P, Sherrad H, Woodend K. Determination of the burden of care in families of cardiac surgery patients. Progress in Cardiovascular Nursing. 2000; 15:4–10
22. Falk, JA, Kadiev, S, Criner, GJ, Scharf, SM, Minai, OA, Diaz, P. Cardiac disease in chronic obstructive pulmonary disease. *Proceedings of the American Thoracic Society*. 2008;5(4): 543-548. Available from: doi:10.1513/pats.200708-142ET.

For peer review only - http://bmjopen.bmj.com/site/about/guidelines.xhtml

23. Müllerova, H, Mapel, DW. Cardiovascular comorbidity in COPD systematic literature review. *CHEST.* 2013;144(4):1163–1178. Available from: doi:10.1378/chest.12-2847.
24. Moher, D, Liberati, A, Tetzlaff, J, Altman, DG, The PRISMA Group. Preferred reporting items for systematic reviews and meta-analyses: The PRISMA statement. *PLoS Med.* 2009;6(7). Available from: doi:10.1371/journal.pmed.1000097.
25. Johnson, RB, Onwuegbuzie, AJ, Turner, LA. Toward a definition of mixed methods research. *Journal of Mixed Methods Research,* 2007;1:112–133. Available from: doi:10.1177/1558689806298224.
26. World Bank Data. Available from: http://www.worldbank.org/ (Accessed 10th June 2017).
27. Critical Appraisal Skills Programme. *Qualitative research checklist.* Available from: http://docs.wixstatic.com/ugd/dded87_25658615020e427da194a325e7773d42.pdf (Accessed 10th December 2016).
28. Sandelowski, M, Voils, CI, Barroso, J. Defining and Designing Mixed Research Synthesis Studies. *Research in the Schools : A Nationally Refereed Journal Sponsored by the Mid-South Educational Research Association and the University of Alabama.* 2006; 13(1):1-15.
29. Pluye, P, Hong, QN. *Annual Review of Public Health*. 2014. Available from: www.annualreviews.org (Accessed September 27th 2016).
30. Hong, QN, Pluye, P, Bujold, M, Wassef, M. Convergent and sequential synthesis designs: implications for conducting and reporting systematic reviews of qualitative and quantitative evidence. *Systematic Reviews*. 2017; 6(61). Available from: doi:10.1186/s13643-017-0454-2.
31. Tashakkori, A, Teddlie, C. Mixed Methodology: Combining Qualitative and Quantitative Approaches. *Applied Social Research Methods Series*. 1998; 46. SAGE Publications, Thousand Oaks, CA.
32. Noblit, GW. & Hare, RD. Meta-ethnography: synthesizing qualitative studies. 1988. SAGE Publications, Thousand Oaks, CA.
33. Tashakkori, A, Teddlie, C. Foundations of Mixed Methods Research: integrating quantitative and qualitative approaches in the social and behavioural sciences. 2009. SAGE Publications Thousand Oaks, CA.
34. Frantzen, KK, Fetters, MD. Meta-integration for synthesizing data in a systematic mixed studies review: insights from research on autism spectrum disorder. *Qual Quant.* 2015;50:2251–2277. Available from: doi:10.1007/s11135-015-0261-6.
35. Rutledge, T, Reis, VA, Linke, SE, Greenberg, BH, Mills, PJ. Depression in heart failure. A meta-analytic review of prevalence, intervention effects, and associations with clinical outcomes. *Journal of the American College of Cardiology,* 2006, 48(8):1527-1537. Available from doi:10.1016/j.jacc.2006.06.055.
36. Dixon-Woods, M, Fitzpatrick, R, Roberts, K. Including qualitative research in systematic reviews: opportunities and problems. Journal of Evaluation in Clinical Practice. 2001; 7(2):125-133. Available from: doi:10.1046/j.1365-2753.2001.00257.x.
37. Ågren, S, Evangelista, L, Strömberg, A. Do partners of patients with chronic heart failure experience caregiver burden? *European Journal of Cardiovascular Nursing*, 2010;9(4):254–262. Available from: doi:10.1016/j.ejcnurse.2010.03.001.
38. Al-Rawashdeh, SY, Lennie, TA, Chung, ML. The association of sleep disturbances with quality of life in heart failure patient–caregiver dyads. *Western Journal of Nursing Research*, 2017;39(4):492–506. Available from: doi: 10.1177/0193945916672647.
39. Badr, H, Federman, AD, Wolf, M, Revenson, TA Wisnivesky, JP. Depression in individuals with chronic obstructive pulmonary disease and their informal caregivers. *Aging & Mental Health. 2017;*21(9): 975-982. Available from: doi:10.1080/13607863.2016.1186153.

For peer review only - http://bmjopen.bmj.com/site/about/guidelines.xhtml

40. Bakas, T, Pressler, SJ, Johnson, E, Nauser, J, Shaneyfelt, T. Family caregiving in heart failure. *Nursing Research.* 2006;55(3):180-188.
41. Chung, ML, Lennie, TA, Mudd-Martin, G, Dunbar, SB, Pressler, SJ, Moser, DK. Depressive symptoms in patients with heart failure negatively affect family caregiver outcomes and quality of life. *European Journal of Cardiovascular Nursing. 2016;*15 (1):30-38. Available from: doi:10.1177/1474515114535329.
42. Cossette, S, Levesque, L. Caregiving tasks as predictors of mental health of wife caregivers of men with chronic obstructive pulmonary disease. *Research in Nursing & Health*, 1993;16:251-263.
43. Evangelista, l, Dracup, K, Doering, L, Westalke, C, Fonarow, GC Hamilton, M. Emotional well-being of heart failure patients and their caregivers. *Journal of Cardiac Failure*. 2002;8(5): p.300-305. Available from: doi:10.1054/jcaf.2002.128005.
44. Figueiredo, D., Gabriel, R., Jácome, C. & Marques, A. Caring for people with early and advanced chronic obstructive pulmonary disease: how do family carers cope? *Journal of Clinical Nursing.* 2013;23:211–220. Available from: doi: 10.1111/jocn.12363.
45. Figueiredo, D, Gabriel, R, Jácome, C, Cruz, J, Marques, A. Caring for relatives with chronic obstructive pulmonary disease: how does the disease severity impact on family carers? *Aging & Mental Health.* 2014;18(3):385-393. Available from: doi:10.1080/13607863.2013.837146.
46. Grigorovich, A, Lee, A, Ross, H, Woodend, K, Forde, S, Cameron, JI. A longitudinal view of factors that influence the emotional well-being of family caregivers to individuals with heart failure. *Aging & Mental Health*. 2017;21(8):844-850. Available from: doi:10.1080/13607863.2016.1168361.
47. Halm, MA, Jacobson-Treat, D, Lindquist, R. Correlates of caregiver burden after coronary artery bypass surgery. *Nursing Research*. 2006;55(6):426-436.
48. Halm, MA, Bakas, TB. Factors associated with caregiver depressive symptoms, outcomes, and perceived physical health after coronary artery bypass surgery. *Journal of Cardiovascular Nursing*. 2007;22(6):508-515. Available from: doi:10.1097/01.JCN.0000297388.21626.6c.
49. Hooley, PJD, Butler, G, Howlett, JG. The relationship of quality of life, depression, and caregiver burden in outpatients with congestive heart failure. *Congestive Heart Failure.* 2005;11(6):303-310. Available from: doi:10.1111/j.1527-5299.2005.03620.x.
50. Hwang, B, Fleischmann, KE, Howie-Esquivel, J, Stotts, NA, Dracup, K. Caregiving for patients with heart failure: impact on patients' families. *American Journal of Critical Care.* 2011;20(6):431-441. Available from: doi: 10.4037/ajcc2011472.
51. Karmilovich, SE. Burden and stress associated with spousal caregiving for individuals with heart failure. *Progress in Cardiovascular Nursing.* 1994;9(1):33-38.
52. Loftus, L. The influence of illness and psychological factors on patient and caregiver outcomes in heart failure. PhD [dissertation]. Liverpool: University of Liverpool; 2004.
53. Nakken, N, Janssen, DJA, van Vilet, M, de Vries, GJ, Clappers-Gielen, GAL, Michels, AJ, Spruit, MA. Gender differences in' partners of patients with COPD and their perceptions about the patients. *International Journal of COPD*. 2017;12: 95–104. Available from: doi:https://doi.org/10.2147/COPD.S118871.
54. Ping-Meh, Y, Bull M. Use of the resiliency model of family stress, adjustment and adaptation in the analysis of family caregiver reaction among families of older people with congestive heart failure. *International Journal of Older People Nursing.* 2012;7:117–126. Available from: doi:10.1111/j.1748-3743.2011.00275.
55. Pressler, SJ, Gradus-Pizlo, I, Chubinski, SD, Smith, G, Wheeler, S, Sloan, R, Jung, M. Family caregivers of patients with heart failure: a longitudinal study. *Journal of Cardiovascular Nursing.* 2013;28(5):417-428. Available from: doi:10.1097/JCN.0b013e3182563877.

For peer review only - http://bmjopen.bmj.com/site/about/guidelines.xhtml

56. Saunders, MM. Factors associated with caregiver burden in heart failure family caregivers. *Western journal of nursing research.* 2008;30(8):943-959. Available from: doi: 10.1177/0193945908319990.
57. Saunders, MM. Indicators of health-related quality of life in heart failure family caregivers. *Journal of Community Health Nursing.* 2009;26:173–182. Available from: doi: 10.1080/07370010903259196.
58. Saunders, MM. Working and caregiving a comparison of employed and unemployed caregivers of older heart failure patients. *Holistic Nursing Practice*. 2010;24(1):16-22. Available from: doi:10.1097/HNP.0b013e3181c8e469.
59. Schwarz, KA, Dunphy, G. An examination of perceived stress in family caregivers of older adults with heart failure. *Experimental Aging Research*. 2003;29(2):221-235. Available from doi:10.1080/03610730303717.
60. Scott, LD. Caregiving and care receiving among a technologically dependent heart failure population. *Advances in Nursing Science.* 2000;23(2):82-97
61. Takata, S, Washio, M, Moriwaki, A, Tsuda, T, Nakayama, H, Iwanaga, T, Inoue, H. Burden among caregivers of patients with chronic obstructive pulmonary disease with long-term oxygen therapy. *International Medical Journal.* 2008;15(1):53-57.
62. Vellone, E, D'Agostino, F, Buck, HG, Fida, R, Spatola, CF, Petruzzo, A, Riegel, B. The key role of caregiver confidence in the caregiver's contribution to self-care in adults with heart failure. *European Journal of Cardiovascular Nursing*. 2015;14(5):372–381. Available from: doi:10.1177/1474515114547649.
63. Woolfe, P, McMillan, M, Conway, J. The needs of caregivers of people with COPD: a study. *Australian Journal of Primary Health.* 2007;13(1):28-35. Available from: doi: 10.2147/COPD.S76091.
64. Andersen, KL, Korneliussen, K, Fagermoen, MS. Family caregivers to a patient with chronic heart failure living at home: «co-workers» in a blurred health care system. *Journal of Cardiovascular Nursing*. 2015;20(3):162-9.
65. Baker, K, Flattery, M, Salyer, J, Haugh, KH, Maltby, M. Caregiving for patients requiring left ventricular assistance device support. *Heart & Lung.* 2010;39(3):196-200. Available from: doi:10.1016/j.hrtlng.2009.07.007.
66. Bove, DG, Zakrisson, AB, Midtgaard, J, Lomborg, K, Overgaard, D. Undefined and unpredictable responsibility: a focus group study of the experiences of informal caregiver spouses of patients with severe COPD. *Journal of Clinical Nursing*. 2016;25:483–493. Available from: doi:10.1111/jocn.13076 483.
67. Burke, RE, Jones, J, Ho, MJ, Bekelman, D. Caregivers' perceived roles in caring for patients with heart failure: what do clinicians need to know? *Journal of Cardiac Failure,* 2014;20 (10):731-738. Available from: doi.org/10.1016/j.cardfail.2014.07.011.
68. Figueiredo, D, Jácome, C, Gabriel, R, Marques, A. Family care in chronic obstructive pulmonary disease: what happens when the carer is a man? *Scandinavian Journal of Caring Sciences.* 2015;30:721-730. Available from: doi: 10.1111/scs.12298.
69. Halm, MA. Specific needs, concerns, strategies and advice of caregivers after coronary artery bypass surgery. *Heart & Lung*. 2016;45:416-422. Available from: doi.org/10.1016/j.hrtlng.2016.07.001.
70. Halm, MA. Age and gender influences on the needs, concerns and strategies of CABG caregivers. *Heart & Lung*. 2017;46:159-165. Available from: doi.org/10.1016/j.hrtlng.2017.01.012.

For peer review only - http://bmjopen.bmj.com/site/about/guidelines.xhtml

71. Hynes, G, Stokes, A, McCarron, M. Informal care-giving in advanced chronic obstructive pulmonary disease: lay knowledge and experience. *Journal of Clinical Nursing*. 2012;21:1068-1077. Available from: doi: 10.1111/j.1365-2702.2011.03944.
72. Imes, C, Dougherty, CM, Pyper, G, Sullivan, MD. Care of Patients with Heart Failure Descriptive study of partners' experiences of living with severe heart failure. *Heart & Lung*. 2011;40:208-216. Available from: doi:10.1016/j.hrtlng.2010.12.007.
73. Kitko, LA. The work of spousal caregiving in heart failure. PhD [dissertation]. State College, PA: Pennsylvania State University;2010.
74. Liljeroos, M, Ågren, S, Jaarsma, T, Strömberg, A. Perceived caring needs in patient-partner dyads affected by heart failure: a qualitative study. *Journal of Clinical Nursing.* 2014;23:2928–2938. Available from: doi: 10.1111/jocn.12588.
75. Lindqvist, G, Albin, B, Heikkila, K Hjelm, K. Conceptions of daily life in women living with a man suffering from chronic obstructive pulmonary disease. Primary Health Care Research & Development. 2012;14:40–51. Available from: doi.org/10.1017/S146342361200031X.
76. Luttik, MLL, Jaarsma, T. Living with heart failure: partner perspectives. *Journal of Cardiovascular Nursing.* 2007;22(2):131-137. Available from: doi:10.1097/00005082-200703000-00010.
77. Marcucilli, L, Casida, J. From insiders' perspectives: adjusting to caregiving for patients with left ventricular assist devices. *Progress in Transplantation.* 2011;21(2):137-43. Available from: doi:10.7182/prtr.21.2.dq53164u1464u683.
78. Marcucilli, L, Casida, J, Bakas, T, Pagani, FD. Family caregivers' inside perspectives: caring for an adult with a left ventricular assist device as a destination therapy. *Progress in Transplantation*. 2014;24(4):332-340. Available from: doi:10.7182/pit2014684.
79. Pattenden, JF, Roberts, H Lewin, HJP. Living with heart failure; patient and carer perspectives. *European Journal of Cardiovascular Nursing.* 2007;6:273-279. Available from: doi:10.1016/j.ejcnurse.2007.01.097.
80. Rolley, J, Smith, J, DiGiacomo, M, Salamonson, Y, Davidson, P. The caregiving role following percutaneous coronary intervention. *Journal of Clinical Nursing*. 2010;20:227–235. Available from: doi: 10.1111/j.1365-2702.2009.03104.
81. Spence, A, Hasson, F, Waldron, M, Kernohan, G, McLaughlin, D, Cochrane, B, Watson, B. Active carers: living with chronic obstructive pulmonary disease. *International Journal of Palliative Nursing*. 2008;14(8):368-372. Available from: doi:10.12968/ijpn.2008.14.8.30771.
82. Ström, A, Andersen, KL, Korneliussen, K, Fagermoen, MS. Being "on the alert" and "a forced volunteer": a qualitative study of the invisible care provided by the next of kin of patients with chronic heart failure. *Journal of Multidisciplinary Healthcare.* 2015;8:271–277. Available from: doi.org/10.2147/JMDH.S82239.
83. Wallin, E, Larsson, IM, Rubertsson, S, Kristoferzon, ML. Relatives' experiences of everyday life six months after hypothermia treatment of a significant other's cardiac arrest. *Journal of Clinical Nursing.* 2013;22:1639–1646. Available from: doi: 10.1111/jocn.12112.
84. Wingham, J, Frost, J, Britten, N, Jolly, K, Greaves, C, Abraham, C, Dalal, H. Needs of caregivers in heart failure management: a qualitative study. *Chronic Illness*. 2015;11(4):304-319. Available from: doi:10.1177/1742395315574765.
85. Näsström, L, Luttik, ML, Idvall, E, Strömberg, A. Exploring partners' perspectives on participation in heart failure home care: a mixed-method design. *Journal of Advanced Nursing.* 2017;73(5):1208–1219. Available from: doi: 10.1111/jan.13216.
86. Clark, AM, Reid, ME, Morrisson, CE, Capewell, S, Murdoch, DL, McMurray, JJ. The complex nature of informal care in home-based heart failure management. Journal of Advanced Nursing. 2008;61(4):373–383. Available from: doi:10.1111/j.1365-2648.2007.04527.x.

For peer review only - http://bmjopen.bmj.com/site/about/guidelines.xhtml

87. Hess, AM. *The health literacy, self-care, and medication hassles of patients with heart failure and their caregivers.* Masters [dissertation]. West Lafayette, IN: Purdue University;2009.
88. Kneeshaw, MF, Considine, RM, Jennings, J. Mutuality and preparedness of family caregivers for elderly women after bypass surgery. Applied Nursing Research. 1999;12(3):128-135.
89. Park, EO, Yates, BC, Schumacher, KL, Meza, J, Kosloski, K, Pullen, C. Caregiving demand and difficulty in older adult spousal caregivers after coronary artery bypass surgery. Geriatric Nursing. 2013;34:383-387. Available from: doi.org/10.1016/j.gerinurse.2013.05.007.
90. Riegner, J. *The relationship of role strain, perceived social support, and humor to quality of life among couples experiencing the life-limiting illness of chronic obstructive pulmonary disease.* PhD [dissertation]. Tallahassee, Florida: Florida State University;1996.
91. Miravitlles, M, María, L, Oliva-Moreno, J, Hidalgo-Vega, Á. Caregivers' burden in patients with COPD. *International Journal of COPD.* 2015;10(1):347—356. Available from: doi.org/10.2147/COPD.S76091.
92. Lum, HD, Lo, D, Hooker, S, Bekelman, D.B. Caregiving in heart failure: relationship quality is associated with caregiver benefit finding and caregiver burden. Heart & Lung. 2014;43:306-310. Available from: doi.org/10.1016/j.hrtlng.2014.05.002.
93. Luttik, ML, Jaarsma, T, Lesman, I, Sanderman, R, Hagedoorn, M. Quality of life in partners of people with congestive heart failure: gender and involvement in care. *Journal of Advanced Nursing.* 2009;65(7):1442–1451. Available from: doi: 10.1111/j.1365-2648.2009.05004.
94. Kang, X, Li, Z, Nolan, MT. Informal caregivers' experiences of caring for patients with chronic heart failure Systematic review and metasynthesis of qualitative studies. 2011; 26(5),386-394.
95. AasbØ, G, Nyheim Solbrække, K, Kristvik, E, Werner, A. Between disruption and continuity: challenges in maintaining the "biographical we" when caring for a partner with a severe chronic illness. *Sociology of health and illness*. 2016; 38(5):782-796. Available from: doi:10.1111/1467-9566.12396.
96. Wingham, J, Frost, J Britten, N. Behind the smile: qualitative study of caregivers' anguish and management responses while caring for someone living with heart failure. *BMJ Open*. 2017; 7:e014126. Available from: doi:10.1136/bmjopen-2016-014126.
97. Lawton, MP, Moss, M, Kleban, MH, Glicksman, A, Rovine, M. A Two-factor model of caregiving appraisal and psychological well-being. *Journal of Gerontology*. 1991; 46(4): 181–P189. Available from: https://doi.org/10.1093/geronj/46.4.P181.
98. Lee, CS Bidwell, JT, Paturzo, M, Alvaro, R, Cocchieri, A, Jaarsma, T, Stromberg, A, Riegel, B, Vellone, E. Patterns of self-care and clinical events in a cohort of adults with heart failure: 1 year follow-up. *Heart & Lung.* 2018; 47(1): 40-46. Available from: doi: 10.1016/j.hrtlng.2017.09.004
99. Nakken, N, Janssen, DJA, van den Bogaart, EHA, Wouters, EFM, Franssen, FME, Vercoulen, JH, Spruit, MA. *European Respiratory Review.* 2015; 24(137):498-504. Available from: doi: 10.1183/16000617.00010114.
100. Feeney Mahoney, D. Vigilance: Evolution and Definition for Caregivers of Family Members with Alzheimer's Disease. Journal of Gerontological Nursing. 2003;29(8):24-30. Available from: https://doi.org/10.3928/0098-9134-20030801-07
101. Buck, HG, Harkness, K, Wion, R, Carroll, SL, Cosman, T, Kaasalainen, S, Arthur, HM. Caregiver's contributions to heart failure self-care: a systematic review. *European Journal of Cardiovascular Nursing*. 2015;14(1):79-89. Available from: doi:10.1177/1474515113518434.
102. Dixon-Woods, M, Agarwal, S, Jones, D, Young, B, Sutton, A. Synthesising qualitative and quantitative evidence: a review of possible methods. *Journal of Health Services Research*. 2005;10(1):45-53
103. Bazely, P. Integrating analyses in mixed methods research. SAGE Publications London. 2018

For peer review only - http://bmjopen.bmj.com/site/about/guidelines.xhtml

Figure Legend Table 2

***Primary Focus refers to the aims of the papers as identified by the authors. The data extracted from each paper as part of this systematic review may reflect a different focus. Data extracted in this review pertains specifically to caregivers experiences.**

HF = Heart Failure, COPD = Chronic Obstructive Pulmonary Disease, LVAD = Left Ventricular Assist Device, LVAD DT = Left Ventricular Assist Device Destination Therapy, CAD = Coronary Artery Disease, Cg/cg/cgs = Caregiver/s, Pt/Pts/pts = patient/s, N/S = Not stated, CABG = Coronary Artery Bypass Graft, QOL = Quality of Life, ADL = Activities of daily living, *= Same study

Figure Legend Table 3

CAD – Coronary Artery Disease
HF – Heart Failure
COPD – Chronic Obstructive Pulmonary Disease

*** Caregiver Data based on data collected in 50 studies**
***Patient Data based on data collected in 35 studies**
● There were 57 publications, however there were 54 studies. The following studies utilised the same data but produced two publications: Halm, 2006 & 2007. Saunders, 2008 & 2009. Halm 2016 & 2017.

Figure Legend Table 4(a)
H = High Quality. 4/ 5 out of 5 quality criteria achieved
M= Medium Quality. 3 out of 5 quality criteria achieved
L= Low Quality. 1 or 2 out of 5 quality criteria achieved
•= same study ***Mixed methods study – qualitative component**

Figure Legend Table 4(b)
Study design - CS: cross-sectional design; LS: longitudinal design
Participant sampling – Purp: purposive; Rand: random; Cons: consecutive; Con: convenience; NS; not stated/unclear
Attrition – 20% or less = +1; NS: Not Stated
Outcome measures – V: all validated outcomes; non-V: some or all non-validated; NS: not stated/unclear
Data analysis – MV: multivariate; UV: univariate
•= same study ***Mixed method study – quantitative component**

Overall quality rating
H = High Quality. 4/ 5 out of 5 quality criteria achieved
M= Medium Quality. 3 out of 5 quality criteria achieved
L= Low Quality. 1 or 2 out of 5 quality criteria achieved

For peer review only - http://bmjopen.bmj.com/site/about/guidelines.xhtml

For peer review only

For peer review only - http://bmjopen.bmj.com/site/about/guidelines.xhtml

For peer review only

For peer review only - http://bmjopen.bmj.com/site/about/guidelines.xhtml

For peer review only

For peer review only - http://bmjopen.bmj.com/site/about/guidelines.xhtml

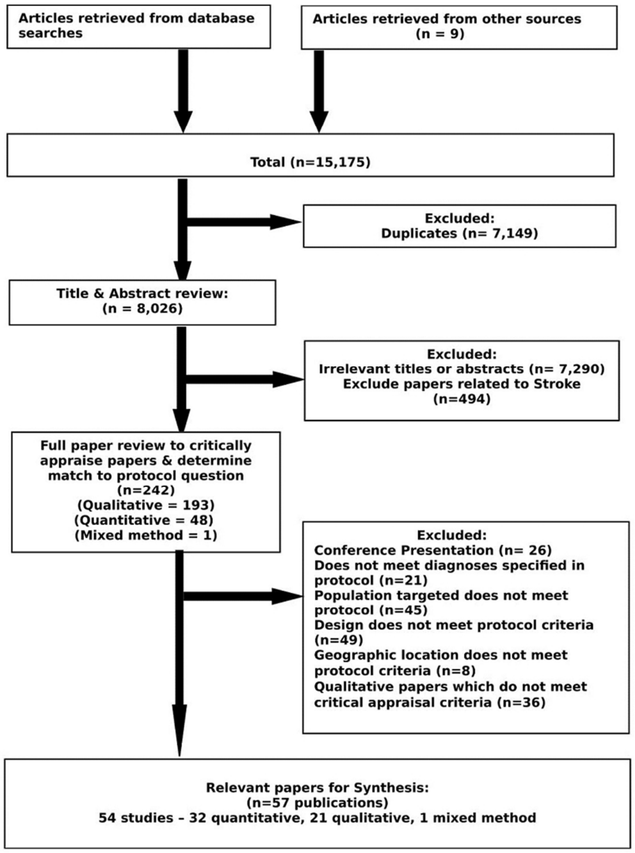

Figure 1 PRISMA

93x131mm (300 x 300 DPI)

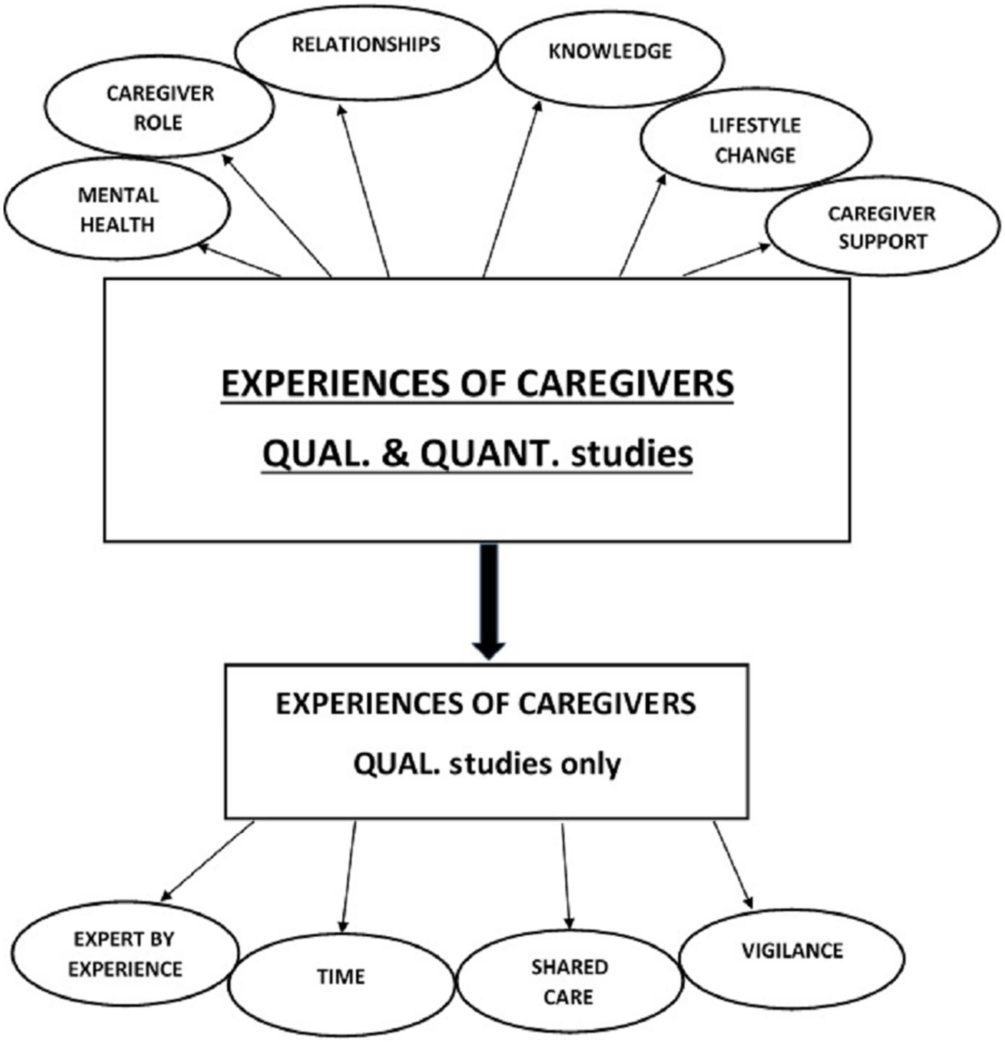

Figure 2 Caregiver Concepts

Supplementary:

Table 1: List of search terms

Medline:

MeSH: Caregivers

“Caregivers”.ti,ab OR “care-giver”.ti,ab OR “carer*.ti,ab” OR “informal car*”.ti,ab

AND

MeSH: Quality of life

“Quality of life” OR “outcome measures” OR “caregiver outcomes” OR “care-giver outcomes” OR “time use” OR “time-use” OR “occupations” OR “occupational engagement” OR “self-efficacy” OR “self efficacy” OR “experience” OR “emotion*” OR “psychological health impact” OR “physical health impact” OR “confidence” OR “self-confidence” OR “satisfaction” OR “dissatisfaction” OR “activities of daily living” OR “resilience” OR “social adj2 (interaction or engagement or support) OR “Social adj2 (participation)” OR “caregiver adj2 (support or health professional or medical team or nurse or patient or relationship* or knowledge)” OR “information exchange” OR “coping strategies” OR “leisure activity” OR “conflict” OR “caregiver responsib*” OR care-giver responsib*” OR “caregiver expectation” OR “care-giver expectation” OR “caregiver role” OR “care-giver role” OR “role adjustment” OR “caregiver or care-giver adj2 (attitude to health)”

AND

MeSH: Heart Failure, Heart Diseases, Myocardial Ischaemia, Coronary Artery Disease, Pulmonary Disease, Chronic Obstructive, Pulmonary Heart Disease, Chronic Obstructive Pulmonary Disease, Acute Coronary Syndrome, Cardiovascular Diseases, Coronary Artery Disease, Coronary Artery Bypass, Coronary Disease, Coronary Aneurysm, Stroke, Myocardial Infarction

“Heart Failure”.ti,ab OR “cardiac failure”.ti,ab OR “myocardial failure”.ti,ab OR “left ventricular failure”.ti,ab OR “right ventricular failure”.ti,ab OR “cardiomyopathy”.ti,ab, OR “systolic failure”.ti,ab OR “diastolic failure”.ti,ab OR “Chronic Obstructive Pulmonary Disease”.ti,ab OR “COPD”.ti,ab OR “chronic obstructive lung disease”.ti,ab OR “pulmonary disease”.ti,ab OR “pulmonary disorder”.ti,ab OR “respiratory disease”.ti,ab OR “respiratory disorder”.ti,ab OR “dyspnea”.ti,ab OR “dyspnoea”.ti,ab OR “Stroke”.ti,ab OR “stroke disability”.ti,ab OR “stroke disease”.ti,ab OR “stroke disorder”.ti,ab OR “CVA”.ti,ab OR “cerebrovascular accident”.ti,ab OR “transient ischaemic attack”.ti,ab OR “transient ischemic attack”.ti,ab OR “haemorrhage”.ti,ab OR “hemorrhage”.ti,ab OR “cerebral haemorrhage”.ti,ab OR “cerebral hemorrhage”.ti,ab OR “aneurysm”.ti,ab OR “atrial fibrillation”.ti,ab OR “Coronary Artery Disease”.ti,ab OR “acute coronary syndrome”.ti,ab OR “atherosclerosis”.ti,ab OR “arteriosclerosis”.ti,ab OR “ischaemic heart disease”,ti.ab OR “ischemic heart disease”.ti,ab OR “myocardial infarction”,ti.ab OR “coronary revascularisation”.ti,ab OR “coronary revascularization”.ti,ab OR “angina”.ti,ab OR “CABG”.ti,ab OR “coronary artery bypass graft”.ti,ab OR “stable angina”.ti,ab OR “angina pectoris”.ti,ab

For peer review only

CINAHL:

MeSH: Caregivers, caregiver burden, caregiver supports, caregiver role strain, risk for caregiver role strain, family caregiver status, caregiving endurance potential, caregiver-patient relationship, caregiver well-being, caregiver support, caregiver stressors, caregiver physical health, caregiver performance, caregiver performance: direct care, caregiver performance indirect care, caregiver lifestyle disruption, caregiver emotional health, caregiver strain index, caregiver role strain.

"Caregiver".ti,ab OR "care-giver".ti,ab OR "carer*.ti,ab" OR "carer".ti,ab OR "carers".ti,ab OR "informal care*".ti,ab

AND

MeSH: Quality of life

"Quality of life" OR "outcome measures" OR "caregiver outcomes" OR "time use" OR "occupations" OR "occupational engagement" OR "self-efficacy" OR "self efficacy" OR "experience" OR "emotion*" OR "psychological health impact" OR "physical health impact" OR "confidence" OR "self-confidence" OR "satisfaction" OR "dissatisfaction" OR "activities of daily living" OR "resilience" OR "social adj2 (interaction or engagement or support) OR "Social participation" OR "caregiver adj2 (support or health professional or medical team or nurse or patient or relationship* or knowledge)" OR "information exchange" OR "coping strategies" OR "leisure activity" OR "conflict" OR "caregiver responsib*" OR care-giver responsib*" OR "caregiver expectation" OR "care-giver expectation" OR "caregiver role" OR "care-giver role" OR "role adjustment" OR "caregiver or care-giver adj2 (adjustment)" OR "caregiver or care-giver adj2 (acceptance) OR "caregiver or care-giver adj2 (readiness)" OR "caregiver or care-giver adj2 (attitude to health)"

AND

MeSH: Heart Failure, Treatment Failure, Pulmonary Disease, Chronic Obstructive Pulmonary Disease, Chronic Obstructive Pulmonary Disease, Acute Coronary Syndrome, Coronary Arteriosclerosis, Coronary Artery Bypass, Coronary Disease, Coronary Aneurysm, Stroke OR Coronary Artery Disease

"Heart Failure".ti,ab OR "cardiac failure".ti,ab OR "myocardial failure".ti,ab OR "left ventricular failure".ti,ab OR "right ventricular failure".ti,ab OR "cardiomyopathy".ti,ab, OR "systolic failure".ti,ab OR "diastolic failure".ti,ab OR "COPD".ti,ab OR "chronic obstructive lung disease".ti,ab OR "pulmonary disease".ti,ab OR "pulmonary disorder".ti,ab OR "respiratory disease".ti,ab OR "respiratory disorder".ti,ab OR "dyspnea".ti,ab OR "dyspnoea".ti,ab OR "Stroke".ti,ab OR "stroke disability".ti,ab OR "stroke disease".ti,ab OR "stroke disorder".ti,ab OR "CVA".ti,ab OR "cerebrovascular accident".ti,ab OR "transient ischaemic attack".ti,ab OR "transient ischemic attack".ti,ab OR "haemorrhage".ti,ab OR "hemorrhage".ti,ab OR "cerebral haemorrhage".ti,ab OR "cerebral hemorrhage".ti,ab OR "aneurysm".ti,ab OR "atrial fibrillation".ti,ab OR "Coronary Artery Disease".ti,ab OR "acute coronary syndrome".ti,ab OR "atherosclerosis".ti,ab OR "arteriosclerosis".ti,ab OR "ischaemic heart disease",ti.ab OR "ischemic heart disease".ti,ab OR "myocardial infarction",ti.ab OR "coronary revascularisation".ti,ab OR "coronary revascularization".ti,ab OR "angina".ti,ab OR "CABG".ti,ab OR "coronary artery bypass graft".ti,ab OR "stable angina".ti,ab OR "angina pectoris".ti,ab

For peer review only

EMBASE:

MeSH: Caregivers

“Caregivers”.ti,ab OR “care-givers”.ti,ab OR “Caregiver”.ti,ab OR “care-giver”.ti,ab OR “carer*.ti,ab” OR “informal car*”.ti,ab

AND

MeSH: Quality of life

“Quality of life” OR “outcome measures” OR “caregiver outcomes” OR “care-giver outcomes” OR “time use” OR “occupations” OR “occupational engagement” OR (“self-efficacy” OR “self efficacy”) OR “experience” OR “emotion*” OR “psychological health impact” OR “physical health impact” OR (“confidence” OR “self-confidence”) OR “satisfaction” OR “dissatisfaction” OR “activities of daily living” OR “resilience” OR “social adj2 (interaction or engagement or support) OR “Social participation” OR “caregiver OR care-giver adj2 (support or health professional or medical team or nurse or patient or relationship* or knowledge)” OR “information exchange” OR “coping strategies” OR “leisure activity” OR “conflict” OR “caregiver responsib*” OR care-giver responsib*” OR “caregiver expectation” OR “care-giver expectation” OR “caregiver role” OR “care-giver role” OR “role adjustment” OR “caregiver or care-giver adj2 (adjustment or acceptance or readiness)” OR “caregiver or care-giver adj2 (attitude to health)”

AND

MeSH: Heart Failure, Chronic Obstructive Pulmonary Disease, Bronchitis, Chronic Obstructive, Disease exacerbation, Pulmonary Disease, Cardiovascular Disease, Stroke, Cerebrovascular Accident, Coronary Artery Disease, Ischemic Heart Disease, Coronary Artery Disease, Acute Coronary Syndrome, Heart Infarction, Coronary Aneurysm, Coronary Artery Aneurysm, Coronary Artery Bypass, Coronary Artery Bypass Graft, Coronary arteriosclerosis, Coronary Artery Atherosclerosis

“heart failure”,ti,ab OR “cardiac failure”.ti,ab OR “myocardial failure”.ti,ab OR “left ventricular failure”.ti,ab OR “right ventricular failure”.ti,ab OR “cardiomyopathy”.ti,ab, OR “systolic failure”.ti,ab OR “diastolic failure”.ti,ab OR “chronic obstructive pulmonary disease” OR “COPD”.ti,ab OR “chronic obstructive lung disease”.ti,ab OR “pulmonary disease”.ti,ab OR “pulmonary disorder”.ti,ab OR “respiratory disease”.ti,ab OR “respiratory disorder”.ti,ab OR “stroke disorder”.ti,ab OR “CVA”.ti,ab OR “cerebrovascular accident”.ti,ab OR “transient ischaemic attack”.ti,ab OR “transient ischemic attack”.ti,ab OR “haemorrhage”.ti,ab OR “hemorrhage”.ti,ab OR “cerebral haemorrhage”.ti,ab OR “cerebral hemorrhage”.ti,ab OR “aneurysm”.ti,ab OR “atrial fibrillation”.ti,ab OR “Coronary Artery Disease”.ti,ab OR “acute coronary syndrome”.ti,ab OR “atherosclerosis”.ti,ab OR “arteriosclerosis”.ti,ab OR “ischaemic heart disease”,ti.ab OR “ischemic heart disease”.ti,ab OR “myocardial infarction”,ti.ab OR “coronary revascularisation”.ti,ab OR “coronary revascularization”.ti,ab OR “angina”.ti,ab OR “CABG”.ti,ab OR “coronary artery bypass graft”.ti,ab OR “stable angina”.ti,ab OR “angina pectoris”.ti,ab

For peer review only

PsycInfo:

MeSH: Caregivers

“caregiver”.ti,ab OR “care-giver”.ti,ab OR “carer*.ti,ab” OR “informal car*”.ti,ab

AND

MeSH: Quality of life, Occupations, Well-being, Activities of daily living, Mental Health, Health Behaviour, Emotional Responses, Life satisfaction, Role satisfaction, Resilience, Life Experience, Communication, Strategies, Anxiety, Coping Behaviour, Distress, Recreation, Leisure Time, Participation, Adjustment, Couples

“Quality of life” OR “outcome measures” OR (“caregiver outcomes” OR “care-giver outcomes”) OR (“time-use” OR “time use”) OR “occupations” OR “occupational engagement” OR (“self-efficacy” OR “self efficacy”) OR “experience” OR “emotion*” OR “psychological health impact” OR “physical health impact” OR (“confidence” OR “self-confidence” OR self confidence) OR “satisfaction” OR “dissatisfaction” OR “activities of daily living” OR “resilience” OR “social adj2 (interaction or engagement or support or participation) OR “caregiver OR care-giver adj2 (support or health professional or medical team)” OR “information exchange” OR “coping strategies” OR “leisure activity” OR “conflict” OR “caregiver responsib*” OR care-giver responsib*” OR “caregiver expectation” OR “care-giver expectation” OR “caregiver role” OR “care-giver role” OR “role adjustment” OR “caregiver or care-giver adj2 (adjustment or acceptance or readiness)” OR “caregiver or care-giver adj2 (attitude to health)”

AND

MeSH: Heart Failure, Health Behaviour, Heart Disorders, Cardiovascular Disorders, Self-care skills, Intervention, Treatment Outcomes, Chronic Obstructive Pulmonary Disease, Pulmonary Heart Disease, Stroke, Treatment Outcomes, Intervention, Cerebrovascular Accidents, Coronary Artery Disease, Myocardial Infarctions, Ischemia, Aneurysms

“heart failure”,ti,ab OR “cardiac failure”.ti,ab OR “myocardial failure”.ti,ab OR “left ventricular failure”.ti,ab OR “right ventricular failure”.ti,ab OR “cardiomyopathy”.ti,ab, OR “systolic failure”.ti,ab OR “diastolic failure”.ti,ab OR “chronic obstructive pulmonary disease” OR “COPD”.ti,ab OR “chronic obstructive lung disease”.ti,ab OR “pulmonary disease”.ti,ab OR “pulmonary disorder”.ti,ab OR “respiratory disease”.ti,ab OR “respiratory disorder”.ti,ab OR “dyspnea”.ti,ab OR “dyspnoea”,ti,ab OR “stroke disorder”.ti,ab OR “CVA”.ti,ab OR “cerebrovascular accident”.ti,ab OR “transient ischaemic attack”.ti,ab OR “transient ischemic attack”.ti,ab OR “haemorrhage”.ti,ab OR “hemorrhage”.ti,ab OR “cerebral haemorrhage”.ti,ab OR “cerebral hemorrhage”.ti,ab OR “aneurysm”.ti,ab OR “atrial fibrillation”.ti,ab OR “Coronary Artery Disease”.ti,ab OR “acute coronary syndrome”.ti,ab OR “atherosclerosis”.ti,ab OR “arteriosclerosis”.ti,ab OR “ischaemic heart disease”,ti.ab OR “ischemic heart disease”.ti,ab OR “myocardial infarction”,ti.ab OR “coronary revascularisation”.ti,ab OR “coronary revascularization”.ti,ab OR “angina”.ti,ab OR “CABG”.ti,ab OR “coronary artery bypass graft”.ti,ab OR “stable angina”.ti,ab OR “angina pectoris”.ti,ab

For peer review only

Web of Science

TI = Title

TS = Topic

TI & TS = “caregiver” OR “care-giver” OR “carer*” OR “informal care*”

AND

TS = “Quality of life” OR “outcome measures” OR (“caregiver outcomes” OR “care-giver outcomes”) OR (“time-use” OR “time use”) OR “occupations” OR “occupational engagement” OR (“self-efficacy” OR “self efficacy”) OR “experience” OR “emotion*” OR “psychological health impact” OR “physical health impact” OR (“confidence” OR “self-confidence” OR self confidence) OR “satisfaction” OR “dissatisfaction” OR “activities of daily living” OR “resilience” OR “social adj2 (interaction or engagement or support or participation) OR “caregiver OR care-giver adj2 (support or health professional or medical team)” OR “information exchange” OR “coping strategies” OR “leisure activity” OR “conflict” OR “caregiver responsib*” OR care-giver responsib*” OR “caregiver expectation” OR “care-giver expectation” OR “caregiver role” OR “care-giver role” OR “role adjustment” OR “caregiver or care-giver adj2 (adjustment or acceptance or readiness)” OR “caregiver or care-giver adj2 (attitude to health)”

AND

TI & TS = “heart failure” OR “cardiac failure” OR “myocardial failure” OR “left ventricular failure” OR “right ventricular failure” OR “cardiomyopathy” OR “systolic failure” OR “diastolic failure” OR “chronic obstructive pulmonary disease” OR “COPD” OR “chronic obstructive lung disease” OR “pulmonary disease” OR “pulmonary disorder” OR “respiratory disease” OR “respiratory disorder” OR “dyspnea” OR “dyspnoea” OR “stroke disorder” OR “CVA” OR “cerebrovascular accident” OR “transient ischaemic attack” OR “transient ischemic attack” OR “haemorrhage” OR “hemorrhage” OR “cerebral haemorrhage” OR “cerebral hemorrhage” OR “aneurysm” OR “atrial fibrillation” OR “Coronary Artery Disease” OR “acute coronary syndrome” OR “atherosclerosis” OR “arteriosclerosis” OR “ischaemic heart disease” OR “ischemic heart disease” OR “myocardial infarction” OR “coronary revascularisation” OR “coronary revascularization” OR “angina” OR “CABG” OR “coronary artery bypass graft” OR “stable angina” OR “angina pectoris”

ProQuest: Global Dissertations & Theses/ Applied Social Sciences Index and Abstracts (ASSIA)

“caregiver”.ti,ab OR “care-giver”.ti,ab OR “carer*.ti,ab” OR “informal car*”.ti,ab

AND

“Quality of life” OR “outcome measures” OR (“caregiver outcomes” OR “care-giver outcomes”) OR (“time-use” OR “time use”) OR “occupations” OR “occupational engagement” OR (“self-efficacy” OR “self efficacy”) OR “experience” OR “emotion*” OR “psychological health impact” OR “physical health impact” OR (“confidence” OR “self-confidence” OR self confidence) OR “satisfaction” OR “dissatisfaction” OR “activities of daily living” OR “resilience” OR “social adj2 (interaction or engagement or support or participation) OR “caregiver OR care-giver adj2 (support or health professional or medical team)” OR “information exchange” OR “coping strategies” OR “leisure activity” OR “conflict” OR “caregiver responsib*” OR care-giver responsib*” OR “caregiver expectation” OR “care-giver expectation” OR “caregiver role” OR “care-giver role” OR “role adjustment” OR “caregiver or care-giver adj2 (adjustment or acceptance or readiness)” OR “caregiver or care-giver adj2 (attitude to health)”

AND

“heart failure”,ti,ab OR “cardiac failure”.ti,ab OR “myocardial failure”.ti,ab OR “left ventricular failure”.ti,ab OR “right ventricular failure”.ti,ab OR “cardiomyopathy”.ti,ab, OR “systolic failure”.ti,ab OR “diastolic failure”.ti,ab OR “chronic obstructive pulmonary disease” OR “COPD”.ti,ab OR “chronic obstructive lung disease”.ti,ab OR “pulmonary disease”.ti,ab OR “pulmonary disorder”.ti,ab OR “respiratory disease”.ti,ab OR “respiratory disorder”.ti,ab OR “dyspnea”.ti,ab OR “dyspnoea”,ti,ab OR “stroke disorder”.ti,ab OR “CVA”.ti,ab OR “cerebrovascular accident”.ti,ab OR “transient ischaemic attack”.ti,ab OR “transient ischemic attack”.ti,ab OR “haemorrhage”.ti,ab OR “hemorrhage”.ti,ab OR “cerebral haemorrhage”.ti,ab OR “cerebral hemorrhage”.ti,ab OR “aneurysm”.ti,ab OR “atrial fibrillation”.ti,ab OR “Coronary Artery Disease”.ti,ab OR “acute coronary syndrome”.ti,ab OR “atherosclerosis”.ti,ab OR “arteriosclerosis”.ti,ab OR “ischaemic heart disease”,ti.ab OR “ischemic heart disease”.ti,ab OR “myocardial infarction”,ti.ab OR “coronary revascularisation”.ti,ab OR “coronary revascularization”.ti,ab OR “angina”.ti,ab OR “CABG”.ti,ab OR “coronary artery bypass graft”.ti,ab OR “stable angina”.ti,ab OR “angina pectoris”.ti,ab

For peer review only

Ethos – British Library

CAREGIVER or CARE-GIVER.ti,ab

Searches initially included Heart Failure, Chronic Obstructive Pulmonary Disease, Stroke and Coronary Artery Disease as "AND" terms, however these yielded no results

For peer review only

Table 2 (a)

Caregiver experience - Concepts/Mapping Table – Qualitative Papers

MENTAL HEALTH = 1 **ROLE = 2** **LIFESTYLE CHANGE = 3**
SUPPORT = 4 **KNOWLEDGE/INFORMATION = 5**
RELATIONSHIPS = 6

Concepts only in Qualitative Papers: **EXPERT BY EXPERIENCE = 7** **VIGILANCE = 8**
TIME = 9 **SHARED CARE = 10**

	1	2	3	4	5	6	7	8	9	10
Andersen (2015) **(HF)**	✓		✓	✓	✓					✓
Baker (2010) **(HF)**	✓	✓	✓	✓		✓		✓		✓
Bove (2016) **(COPD)**	✓	✓	✓	✓	✓	✓	✓	✓	✓	✓
Burke (2014) **(HF)**	✓	✓	✓	✓	✓	✓	✓	✓		✓
Clark (2008) **(HF)**		✓	✓	✓	✓	✓	✓	✓	✓	✓
Figueiredo (2015) **(COPD)**	✓	✓	✓	✓	✓	✓	✓	✓	✓	
• Halm (2016) **(CAD)**	✓	✓	✓	✓	✓	✓	✓	✓	✓	✓
• Halm (2017) **(CAD)**	✓	✓	✓	✓	✓	✓	✓	✓	✓	✓
Hynes (2012) **(COPD)**	✓	✓	✓	✓	✓	✓	✓	✓	✓	✓
Imes (2011) **(HF)**	✓	✓	✓	✓	✓	✓		✓	✓	✓
Kitko (2010) **(HF)**	✓	✓	✓	✓	✓	✓	✓	✓	✓	✓
Liljeroos (2014) **(HF)**	✓		✓	✓	✓	✓		✓	✓	✓
Lindqvist (2013) **(COPD)**	✓	✓	✓	✓	✓	✓		✓	✓	✓
Luttik (2007) **(HF)**	✓	✓	✓	✓	✓	✓	✓	✓	✓	✓
Marcuccilli (2011) **(HF)**	✓		✓	✓		✓	✓	✓	✓	✓
Marcuccilli (2014) **(HF)**	✓	✓	✓	✓	✓	✓		✓	✓	
Pattenden (2007) **(HF)**	✓	✓	✓	✓		✓		✓		
Rolley (2010) **(CAD)**	✓	✓	✓	✓	✓	✓		✓		
Spence (2008) **(COPD)**	✓	✓	✓	✓	✓	✓	✓	✓	✓	✓

For peer review only

Strom (2015) **(HF)**	✓	✓	✓	✓		✓	✓	✓		✓
Wallin (2013) **(CAD)**	✓	✓	✓	✓	✓	✓			✓	
Wingham (2015) **(HF)**	✓	✓	✓	✓	✓	✓	✓	✓	✓	✓
**Heart Failure**	**12**	**10**	**13**	**13**	**9**	**12**	**7**	**12**	**8**	**11**
**COPD**	**5**	**5**	**5**	**5**	**5**	**5**	**4**	**5**	**5**	**4**
**CAD**	**3**	**3**	**3**	**4**	**3**	**3**	**1**	**2**	**2**	**1**
**Total**	**20**	**18**	**21**	**21**	**17**	**20**	**12**	**19**	**15**	**16**

• = same study: counted as one

For peer review only

Table 2 (b)

Caregiver experience - Concepts/Mapping – Quantitative Papers

MENTAL HEALTH IMPACT = 1 **ROLE = 2** **LIFESTYLE CHANGE = 3**
SUPPORT = 4 **KNOWLEDGE/INFORMATION = 5**
RELATIONSHIPS = 6

	**1**	**2**	**3**	**4**	**5**	**6**
Agren (2010) **(HF)**	✓					✓
Al-Rawashdeh (2017) **(HF)**	✓		✓			✓
Badr (2017) **(COPD)**	✓		✓			✓
Bakas (2006) **(HF)**	✓	✓	✓			
Chung (2016) **(HF)**	✓	✓	✓			
Cossette (1993) **(COPD)**	✓	✓		✓		
Evangelista (2002) **(HF)**	✓					✓
Figueiredo (2013) **(COPD)**	✓		✓			✓
Figueiredo (2014) **(COPD)**	✓					✓
Grigorovich (2017) **(HF)**	✓		✓	✓		
• Halm (2006) **(CAD)**	✓			✓		✓
• Halm (2007) **(CAD)**	✓			✓		✓
Hess (2009) **(HF)**		✓				
Hooley (2005) **(HF)**	✓					✓
Hwang (2011) **(HF)**	✓	✓		✓		✓
Karmilovich (1994) **(HF)**	✓	✓			✓	✓
Kneeshaw (1999) **(CAD)**		✓	✓	✓	✓	✓
Loftus (2004) **(HF)**	✓	✓		✓		✓
Lum (2014) **(HF)**						✓
Luttik (2009) **(HF)**						✓

For peer review only

Miravitlles (2015) **(COPD)**			✓			✓
Nakken (2017) **(COPD)**	✓		✓	✓		✓
Park (2013) **(CAD)**		✓	✓			✓
Pi-Ming Yeh (2012) **(HF)**	✓			✓		
Pressler (2013) **(HF)**	✓	✓	✓			✓
Riegner (1996) **(COPD)**		✓		✓		✓
• Saunders (2008) **(HF)**	✓			✓		✓
• Saunders (2009) **(HF)**	✓			✓		✓
Saunders (2010) **(HF)**	✓	✓				✓
Schwarz (2003) **(HF)**	✓			✓		
Scott (2000) **(HF)**	✓	✓	✓	✓		
Takata (2008) **(COPD)**	✓	✓	✓	✓	✓	
Vellone (2015) **(HF)**	✓				✓	✓
Woolfe (2007) **(COPD)**	✓		✓	✓	✓	
**Heart Failure**	**15**	**9**	**6**	**7**	**2**	**13**
**COPD**	**5**	**3**	**6**	**5**	**2**	**6**
**CAD**	**4**	**2**	**2**	**3**	**1**	**3**
**Mixed Method Study**	**1**					
**Total**	**25**	**14**	**14**	**15**	**5**	**22**

• = same study: counted as one

For peer review only

Table 2 (c)

Caregiver experience - Concepts/Mapping – Mixed Method Papers

MENTAL HEALTH IMPACT = 1 **ROLE = 2** **LIFESTYLE CHANGE = 3**
SUPPORT = 4 **KNOWLEDGE/INFORMATION = 5**
RELATIONSHIPS = 6

	**1**	**2**	**3**	**4**	**5**	**6**
Näsström (2017) **(HF)**	✓			✓	✓	✓
**TOTAL**	**1**			**1**	**1**	1

For peer review only

PRISMA 2009 Checklist

Section/topic	#	Checklist item	Reported on page #
**TITLE**			
Title	1	Identify the report as a systematic review, meta-analysis, or both.	1
**ABSTRACT**			
Structured summary	2	Provide a structured summary including, as applicable: background; objectives; data sources; study eligibility criteria, participants, and interventions; study appraisal and synthesis methods; results; limitations; conclusions and implications of key findings; systematic review registration number.	2
**INTRODUCTION**			
Rationale	3	Describe the rationale for the review in the context of what is already known.	4,5
Objectives	4	Provide an explicit statement of questions being addressed with reference to participants, interventions, comparisons, outcomes, and study design (PICOS).	4,5,6
**METHODS**			
Protocol and registration	5	Indicate if a review protocol exists, if and where it can be accessed (e.g., Web address), and, if available, provide registration information including registration number.	2
Eligibility criteria	6	Specify study characteristics (e.g., PICOS, length of follow-up) and report characteristics (e.g., years considered, language, publication status) used as criteria for eligibility, giving rationale.	5,6
Information sources	7	Describe all information sources (e.g., databases with dates of coverage, contact with study authors to identify additional studies) in the search and date last searched.	5
Search	8	Present full electronic search strategy for at least one database, including any limits used, such that it could be repeated.	5
Study selection	9	State the process for selecting studies (i.e., screening, eligibility, included in systematic review, and, if applicable, included in the meta-analysis).	5-24
Data collection process	10	Describe method of data extraction from reports (e.g., piloted forms, independently, in duplicate) and any processes for obtaining and confirming data from investigators.	6
Data items	11	List and define all variables for which data were sought (e.g., PICOS, funding sources) and any assumptions and simplifications made.	5,6
Risk of bias in individual studies	12	Describe methods used for assessing risk of bias of individual studies (including specification of whether this was done at the study or outcome level), and how this information is to be used in any data synthesis.	5,6,7,28,29,30,31
Summary measures	13	State the principal summary measures (e.g., risk ratio, difference in means).	6,7
Synthesis of results	14	Describe the methods of handling data and combining results of studies, if done, including measures of consistency (e.g., I^2) for each meta-analysis.	6,7

For peer review only - http://bmjopen.bmj.com/site/about/guidelines.xhtml

PRISMA 2009 Checklist

Section/topic	#	Checklist item	Reported on page #
Risk of bias across studies	15	Specify any assessment of risk of bias that may affect the cumulative evidence (e.g., publication bias, selective reporting within studies).	5,6,7,28,29,30,31,40,41
Additional analyses	16	Describe methods of additional analyses (e.g., sensitivity or subgroup analyses, meta-regression), if done, indicating which were pre-specified.	6,7
**RESULTS**			
Study selection	17	Give numbers of studies screened, assessed for eligibility, and included in the review, with reasons for exclusions at each stage, ideally with a flow diagram.	12
Study characteristics	18	For each study, present characteristics for which data were extracted (e.g., study size, PICOS, follow-up period) and provide the citations.	13-24
Risk of bias within studies	19	Present data on risk of bias of each study and, if available, any outcome level assessment (see item 12).	5,6,7,28,29,30,31
Results of individual studies	20	For all outcomes considered (benefits or harms), present, for each study: (a) simple summary data for each intervention group (b) effect estimates and confidence intervals, ideally with a forest plot.	13-24, 26,27
Synthesis of results	21	Present results of each meta-analysis done, including confidence intervals and measures of consistency.	13-24, 32-39
Risk of bias across studies	22	Present results of any assessment of risk of bias across studies (see Item 15).	5,6,7,28,29,30,31,40,41
Additional analysis	23	Give results of additional analyses, if done (e.g., sensitivity or subgroup analyses, meta-regression [see Item 16]).	38,39
**DISCUSSION**			
Summary of evidence	24	Summarize the main findings including the strength of evidence for each main outcome; consider their relevance to key groups (e.g., healthcare providers, users, and policy makers).	35-39,41-42
Limitations	25	Discuss limitations at study and outcome level (e.g., risk of bias), and at review-level (e.g., incomplete retrieval of identified research, reporting bias).	41,42
Conclusions	26	Provide a general interpretation of the results in the context of other evidence, and implications for future research.	42
**FUNDING**			
Funding	27	Describe sources of funding for the systematic review and other support (e.g., supply of data); role of funders for the systematic review.	42

From: Moher D, Liberati A, Tetzlaff J, Altman DG, The PRISMA Group (2009). Preferred Reporting Items for Systematic Reviews and Meta-Analyses: The PRISMA Statement. PLoS Med 6(7): e1000097. doi:10.1371/journal.pmed1000097

For more information, visit: **www.prisma-statement.org**.

For peer review only - http://bmjopen.bmj.com/site/about/guidelines.xhtml

BMJ Open

“Who Cares?” The Experiences of Caregivers of Adults Living with Heart Failure, Chronic Obstructive Pulmonary Disease and Coronary Artery Disease – A Mixed Methods Systematic Review

Journal:	*BMJ Open*
Manuscript ID	bmjopen-2017-020927.R3
Article Type:	Research
Date Submitted by the Author:	30-May-2018
Complete List of Authors:	Noonan, Miriam; University of Exeter Medical School, European Centre for Environment and Human Health<br>Wingham, Jennifer; Royal Cornwall Hospitals NHS Trust, Research, Development and Innovation; University of Exeter Medical School,<br>Taylor, Rod; University of Exeter, Peninsula Medical School
**Primary Subject Heading**:	Patient-centred medicine
Secondary Subject Heading:	Evidence based practice, Cardiovascular medicine, Respiratory medicine, Research methods, Health services research
Keywords:	Caregivers, Heart failure < CARDIOLOGY, Coronary heart disease < CARDIOLOGY, Chronic obstructive pulmonary disease, Mixed methods systematic review

SCHOLARONE™
Manuscripts

For peer review only

"Who Cares?" The Experiences of Caregivers of Adults Living with Heart Failure, Chronic Obstructive Pulmonary Disease and Coronary Artery Disease – A Mixed Methods Systematic Review

M Noonan[1], J Wingham,[2,] RS Taylor[3]

Author Affiliations:

1. Miriam Noonan. University of Exeter Medical School, European Centre for Environment and Human Health, Knowledge Spa, Royal Cornwall Hospital, Truro, Cornwall, UK.

2. Dr. Jennifer Wingham. Royal Cornwall Hospitals NHS Trust, Research, Development and Innovation, F37, Knowledge Spa, Royal Cornwall Hospital, Truro, Cornwall & University of Exeter Medical School, Exeter, UK.

3. Professor Rod S. Taylor. University of Exeter Medical School, Exeter, UK.

Correspondence to
Miriam C. Noonan
University of Exeter Medical School
European Centre for Environment and Human Health,
Knowledge Spa, Royal Cornwall Hospital, Truro, Cornwall, UK TR13HD, UK
mn354@exeter.ac.uk

(Word Count 4,495)

For peer review only

For peer review only - http://bmjopen.bmj.com/site/about/guidelines.xhtml

ABSTRACT

Objective To assess the experiences of unpaid caregivers providing care to people with heart failure (HF) or chronic obstructive pulmonary disease (COPD) or coronary artery disease (CAD).

Design Mixed methods systematic review including qualitative and quantitative studies.

Data Sources Databases searched: Medline Ebsco, PsycInfo, CINAHL Plus with Full Text, Embase, Web of Science, Ethos: The British Library and ProQuest. Grey literature identified using: Global Dissertations and Theses and Applied Sciences Index and hand searches and citation checking of included references. Search time frame: 1st January 1990 to 30th August 2017.

Eligibility criteria for selecting studies Inclusion was limited to English language studies in unpaid adult caregivers (>18 years), providing care for patients with HF, COPD or CAD. Studies that considered caregivers for any other diagnoses and studies undertaken in low and middle income countries were excluded. Quality assessment of included studies was conducted by two authors.

Data analysis/synthesis - A results based convergent synthesis was conducted

Results Searches returned 8,026 titles and abstracts. 54 studies - 21 qualitative, 32 quantitative, and 1 mixed were included. This totalled 26,453 caregivers who were primarily female (63 %), with average age of 62.2 years. Narrative synthesis yielded six concepts related to caregiver experience: (1) mental health (2) caregiver role (3) lifestyle change (4) support for caregivers (5) knowledge and (6) relationships. There was a discordance between paradigms regarding emerging concepts. Four concepts emerged from qualitative papers which were not present in quantitative papers (1) expert by experience (2) vigilance (3) shared care and (4) time.

Conclusion Caregiving is life altering and complex with significant health implications. Health professionals should support caregivers who in turn can facilitate the recipient to manage their long term condition. Further longitudinal research exploring the evolution of caregiver experiences over time of patients with chronic cardiopulmonary conditions is required.

Systematic review registration - The study protocol was registered on PROSPERO: CRD42016053412.

For peer review only - http://bmjopen.bmj.com/site/about/guidelines.xhtml

Strengths and limitations of this study

• This mixed methods systematic review provides the opportunity for a broadened and deeper understanding of the qualitative and quantitative literature on the experiences of unpaid caregivers' providing care to people with heart failure, chronic obstructive pulmonary disease, and coronary artery disease.

• This review provides an integration of the type and extent of caregiver's experiences and predictors of caregiver experiences.

• To maximise applicability we included studies from higher income countries only.

• Quality of evidence limited by assessment of caregiver experience at single point of time and there is need for future studies that employ longitudinal or repeated measures design.

For peer review only

For peer review only - http://bmjopen.bmj.com/site/about/guidelines.xhtml

INTRODUCTION

A caregiver is anyone providing unpaid care, to a friend or family member who is unable to care for themselves.[1] This may be emotional support; someone to talk to, or practical support; dressing wounds, mobility assistance or medication checking.[2] There are 43.5million caregivers in the United States, 2.86 million in Australia and 6.5million in the UK.[3] Between 2001 to 2011 the number of unpaid caregivers in the UK grew at a faster rate than population growth.[4] The annual value of unpaid care provided to an individual with a chronic illness is estimated to be £132billion.[5]

Focus groups examining a caregivers' life conducted by "The Institute of Public Care" (2017), based at Oxford Brookes University; described caregivers as the 'Skilled Helper' performing a series of roles.[6] Seltzer & Wailing (2000) describe a dynamic process of transitions to being a caregiver.[7] These transitions comprise participating in the role before identifying as a caregiver, acceptance of the role, engaging in it with awareness and sometimes moving beyond the caregiving role when the patient moving to paid care settings or bereavement occurs. This process is not linear and people move through the different transitions at varying rates. Acknowledging this, it is imperative for caregivers to receive a caregiver needs assessment as legally stipulated by the 2014 Care Act. [8] Additionally, the National Institute for Health and Care Excellence (NICE) clinical guidelines for heart failure (HF) (CG108)[9] and chronic obstructive pulmonary disease (COPD) (CG101)[10] both recommend that family members or caregivers are provided with support and included in discussions about care.

Cardio-pulmonary disease is a primary cause of illness. Cardiovascular disease is responsible for 45% deaths in Europe[11] and 1 in 4 deaths in the United States.[12] By 2020, COPD is projected to be in the global top five of diagnoses causing years lost through early mortality or disability-adjusted life years.[13]Caregivers of HF patients have a multitude of unmet needs due to fluctuations in the trajectory of HF.[14] COPD has frequent unplanned hospital admissions and a high morbidity rate.[15] Caregivers experience depressed mood, greater anxiety and increased subjective burden when their support needs are not met.[16,17] The unpredictability of HF and COPD leads to caregivers constantly adjusting their role, creating a need to continuously re-asses what caregiver needs are.[18,19] Spousal caregivers of myocardial infarct patients experience increased levels of stress, lifestyle impact and emotional distress.[20] Caring for coronary artery bypass graft patients in tasks such as monitoring and provision of emotional support increased caregiver burden to a level described as moderate.[21] COPD and cardiovascular disease are both increasing in prevalence and frequently co-exist. [22, 23] We know of no systematic review that synthesises quantitative and qualitative studies to combine caregivers' experiences of people with HF, COPD or coronary artery disease (CAD).

For peer review only - http://bmjopen.bmj.com/site/about/guidelines.xhtml

Using a mixed methods systematic review methodology including both qualitative and quantitative literature, this study aims to understand the experiences of adult caregivers when supporting people with HF, COPD or CAD.

METHODS

We conducted and reported this systematic review in accordance with the PRISMA (Preferred Reporting Items for Systematic Reviews and Meta-Analyses) statement.[24]

Patient and Public Involvement

There was no patient and or public involvement in this systematic review.

Study design

This study employed a mixed methods systematic review assessing both qualitative and quantitative studies.[25] The rationale for using a mixed methods review approach was multifaceted. Firstly, to gain a qualitative assessment of the type and extent of caregiver's experiences. Second to assess the quantitative predictors of caregiver's experiences. Thirdly, to develop a holistic perspective of what caregiver's experience. Finally, we wanted to assess the degree of convergence between qualitative and quantitative experiences.

Search Strategy

Our search strategy was designed in conjunction with a Health Services Librarian and Information Specialists. Search terms included condition specific terms, i.e., "heart failure", "COPD" and "coronary artery disease", caregiver specific, plus experience related terms, "experience", "quality of life" "activities of daily living", "occupational engagement", "time use", "self-efficacy", "coping strategies", "leisure activity", "information exchange" and "caregiver expectation"". (See supplementary file table 1 for complete list of search terms.) Databases searched included: Medline Ebsco, PsycInfo, CINAHL Plus with Full Text, Embase, Web of Science, Ethos: The British Library and ProQuest. Grey literature was identified using Global Dissertations and Theses and Applied Sciences Index and hand searches and citation checking of included references. To ensure the contemporary nature of the evidence considered, the search time frame was January 1990 to August 2017. A single researcher (MN) initially screened titles and abstracts. Selection of full papers was performed by two researchers (MN and either JW or RST) and cross checked with the eligibility criteria.

Study Selection

Studies were included if they addressed 'caregiver experience' which was defined as encompassing the daily activities of caregivers and the impact of these activities on their lives. These were English

For peer review only - http://bmjopen.bmj.com/site/about/guidelines.xhtml

language studies involving unpaid adult caregivers (>18 years), providing care for patients with HF, COPD or CAD living in the community and not residential settings with paid care staff. Qualitative, quantitative and grey literature studies were all included in the search strategy. Conference papers were excluded. Outcomes of interest included psychological and physical outcomes reported, occupational engagement and routine. As we sought to inform the practice of UK and other high income countries we excluded studies undertaken in low and middle income countries. [26]

Data extraction

Data extracted from retained studies included: study design, sample and recruitment, study description, method, findings, discussion and author's conclusions and limitations. Caregiver quotes were extracted from qualitative studies. For quantitative studies, data extraction also included details of attrition and data analysis.

Study quality assessment

Qualitative studies were appraised utilising the Critical Appraisal (CASP) Tool.[27]In absence of an existing quality tool that could be used to appraise quantitative studies addressing the specific question of this study, a quality assessment tool was developed by the research team based on what were deemed to be the appropriate core biases i.e. (1) Was the study design longitudinal (score of 1) or cross sectional (score of 0). (2) How was the sample recruited? Purposive (score of 1) or convenience (score of 0). (3) Was the level of attrition/response rate acceptable? Attrition of 20%/lower or response rate of 80% or above (score of 1) or attrition of more than 20% or response rate less than 80% (score of 0). (4) Was a validated quantitative outcome(s) used? Validated (score of 1), non-validated (score of 0). (5) Were the methods of data analysis appropriate? Multivariate (score of 1) or univariate (score of 0). Based on their quality assessment, scores were totalled and studies were ranked: 1 or 2 "low quality", 3 "medium quality" and 4 or 5 "high quality". Data extraction and quality appraisal was first conducted by a single researcher (MN) and checked by one of two researchers (JW or RST).

Data analysis and synthesis

The methodology of mixed methods data synthesis is an emerging one and no single approach has yet been universally accepted.[28] In this study a results based convergent design was chosen.[29,30] This requires transformation of one method into another. Due to the heterogeneity of the quantitative methods a meta-analysis was not appropriate. Instead, applying a narrative profile formation, quantitative data was converted into qualitative data.[31] Extracted data from quantitative and qualitative studies were imported into separate spreadsheets. A meta-ethnographic approach was utilised to synthesise qualitative studies.[32] A narrative formation approach[33] was utilised to

For peer review only - http://bmjopen.bmj.com/site/about/guidelines.xhtml

synthesise the quantitative data into a qualitative data set. Narrative formation is a verbal description via the use of profiles of each of the studies. The five profiles are modal, average, holistic, comparative and normative.[33] Table 1 provides an example of this approach. This resulted in two qualitative data sets[34] from which concepts emerged. A mapping table was completed in order to provide an audit trail of how the overall concepts across all papers were derived (see supplementary file one table 2a, 2b and 2c). Initial synthesis was conducted by a single researcher (MN) and corroborated by two experienced researchers in quantitative (RST) and qualitative (JW) research.

Table 1

**Profile**	**Explanation of Profile**	**Modal narrative extracted from study**	**Emerging from study**	**Concept**
**Modal**	This is a narrative description of the group being studied	102 dyads, predominantly spousal caregivers. Group is caregivers of patients with HF. Comparing depressed and non-depressed HF pts and the	This study considers whether caregiver experiences are different for caregivers when caring for depressed or non-depressed patients<br><br>Spouse as a caregiver. Impact of depression on the caregiver.	
**Average**	This is a detailed narrative description based on the mean (average) attributes of the individuals/<br><br>situations being studied	Caregiver Mean age 56.7, 78% female, white (94%).<br><br>41% of pts NYHA III-IV<br><br>“42% caregivers reported severe burden (the Zarit Burden Interview	Caregiving resulted in burden experienced by caregivers in this study	Mental Health<br><br>(burden)

For peer review only

For peer review only - http://bmjopen.bmj.com/site/about/guidelines.xhtml

		(ZBI)≥17)”[35]		
**Comparative**	This is a description comparing studies and comparing individuals being studied.	Caregivers who provided care for depressed patients reported higher burden than those caring for non-depressed patients.<br><br>Caregivers related their burden to social life limitations, poor perceived control, stress of family obligations, and patients’ dependency<br><br>“Caregivers of patients with depressive symptoms had a higher level of burden (25±13 vs 13.5±12 on the ZBI; p<0.001), spent more time caregiving (37±12 vs 30±11 on the Oberst Caregiving Burden Scale; p=0.004), and reported worse mental quality of life (46±10 vs 51±10 on the SF-12v2; p=0.026) than those of patients without depressive	Patient illness severity impacts on caregiver<br><br>Life changes negatively impacted on caregivers<br><br>Depressive symptoms of patients are associated with poor outcomes	Lifestyle adjustment<br><br>Mental health

For peer review only - http://bmjopen.bmj.com/site/about/guidelines.xhtml

		symptoms."[35]	of caregivers<br><br>Caregiver's subjective and objective response to the patient's illness severity	
**Normative**	A comparison of the study individuals with the general population	27% of patients in this study scored 14 or higher on Becks Depression Index<br><br>(a score of 14 or higher is clinically significant for depression)<br><br>Family members caring for HF patients with depressive symptoms had significantly higher levels of caregiving burden and worse quality of life compared to those caring for patients without depressive symptoms.<br><br>"Most difficult task for both sets of caregivers – providing emotional support (M=3.3, SD=1.2)"[35]	Greater impact on caregivers lives when patients were depressed	Mental health

For peer review only - http://bmjopen.bmj.com/site/about/guidelines.xhtml

				Role of caregivers
**Holistic (also called inferential or summative)**	A description of the overall perception of the investigator		Female, white caregivers, experienced greater levels of burden, loss of roles and greater distress when patients were depressed. This could be due to the increased need for practical and emotional support, feeling they need to be constantly present	

For peer review only

For peer review only - http://bmjopen.bmj.com/site/about/guidelines.xhtml

RESULTS

Study selection

Study selection process is summarised in a PRISMA Flow diagram shown in Figure 1. Following removal of duplicates the search strategy yielded a total of 8,026 title and abstracts. Of these 242 full papers were reviewed, of which 57 papers (54 studies) were included for synthesis. A detailed summary of included studies is provided in Table 2. (A comprehensive outline of study results and concepts generated by each study is included in supplementary file 2)

For peer review only

For peer review only - http://bmjopen.bmj.com/site/about/guidelines.xhtml

For peer review only

For peer review only - http://bmjopen.bmj.com/site/about/guidelines.xhtml

Table 2

First Author/Year	Diagnosis	Aims (as stated by authors)	Methods	Country	Data Collection Sampling	Participants Caregiver (Time caring)	Mean Age (Caregiver /Recipient)
Ågren (2010)	HF (NYHA II-IV)	Describe the levels and identify independent predictors of cg. burden. in partners of pts with HF.	QUANT (Correlational)	SWEDEN	Cross Sectional Purposive	135 (101F, 34M) (N/S)	69/71
Al-Rawashdeh (2017)	HF (NYHA I-IV)	To examine whether individuals' sleep disturbance predicted their own & their partners' QoL in HF	QUANT	USA	Cross Sectional Purposive	78 Dyads (58F, 20M) (N/S)	62.2./59.5
Andersen (2015)	HF	Obtain knowledge on experiences and views and the desire for knowledge of family cgs of HF pts, their competence and support required	QUAL	NORWAY	Interviews Convenience	19 (17F, 2M) (N/S)	63/N/ S
Badr (2017)	COPD	Individual-level predictors of pt and and cgs depression in COPD as well as how dyad members effect each other's depression	QUANT	USA	Cohort Study Purposive	89 (68F, 21M) (N/S)	54.8/67

For peer review only - http://bmjopen.bmj.com/site/about/guidelines.xhtml

First Author/Year	Diagnosis	Aims (as stated by authors)	Methods	Country	Data Collection Sampling	Participants Caregiver (Time caring)	Mean Age (Caregiver /Recipient)
Bakas (2006)	HF (NYHA II-IV)	Examine relationships among age, perceived control over managing HF perceived difficulty with tasks, perceived outcomes, and perceived mental and general health among cgs of persons with HF	QUANT (Descriptive Correlational)	USA	Cross Sectional Convenience	21 (20F, 1M) (N/S)	59.6/62.7
Baker (2010)	HF (LVAD in situ)	To describe experiences of cgs of pts who received LVAD therapy as a bridge to transplantation	QUAL (Phenomenological) (Descriptive)	USA	Interviews Convenience	6 (5F, 1M) (26-372 days)	51/N/S
Bove (2016)	COPD (GOLD C&D)	Explore how spouses of pts with severe COPD experience their role	QUAL	DENMARK	Focus Groups Purposive	22(13F, 9M) (N/S)	69.4/N/S
Burke (2014)	HF (NYHA II-IV)	Understand what roles cgs perceive and desire for themselves, and to compare and contrast these roles with those they perceive to be desired by the health care system	QUAL (Inductive)	USA	Interviews Purposive	20 (18F, 1M, 1N/S) (<1 - > 8 hours per week)	59/64

For peer review only

For peer review only - http://bmjopen.bmj.com/site/about/guidelines.xhtml

First Author/Year	Diagnosis	Aims (as stated by authors)	Methods	Country	Data Collection Sampling	Participants Caregiver (Time caring)	Mean Age (Caregiver /Recipient)
Chung (2016)	HF (NYHA II-IV)	Examine differences in cg outcomes between cgs who care for HF pts with and without depressive symptoms	QUANT	USA	Cross Sectional Convenience	102 Dyads (79F, 23M) (N/S)	56.7/61.4
Clark (2008)	HF (NYHA II – IV)	To examine the complexity of caregiving for pts with HF	QUAL	SCOTLAND	Interviews Convenience	30 (23F, 7M) (N/S)	68F, 67M/N/S
Cossette (1993)	COPD (GOLD III-V)	Examine relationship between type, number and disturbance of caring tasks and impact on mental health of cg's Examine influence of social support	QUANT	CANADA	Cross Sectional Convenience	89 (F) (Mean = 13yrs)	65/68.6
Evangelista (2002)	HF (NYHA I – IV)	Describe emotional wellbeing of HF pts and cgs Identify factors associated with emotional wellbeing of pts. Determine gender differences in emotional wellbeing of pts and cgs	QUANT (Descriptive) Correlational)	USA	Cross Sectional Convenience	103 Dyads (73F, 30M) (N/S)	59.4/57.6

For peer review only - http://bmjopen.bmj.com/site/about/guidelines.xhtml

First Author/Year	Diagnosis	Aims (as stated by authors)	Methods	Country	Data Collection Sampling	Participants Caregiver (Time caring)	Mean Age (Caregiver /Recipient)
Figueiredo (2013)	COPD (GOLD I-IV)	Examine coping strategies of family cg's of pts with early and advanced COPD and how those relate to their subjective health	QUANT (Correlational)	PORTUGAL	Cross Sectional Convenience	158 (120F, 38M) (> 4 years)	58.4(early COPD) 60.8 (Adv. COPD) /69.4
Figueiredo (2014)	COPD (GOLD I-IV)	To analyse subjective burden of family cg's of early and advanced COPD pts and its predictor variables	QUANT (Correlational)	PORTUGAL	Cross Sectional Convenience	167 (125F, 42M) (> 4 years)	58.3(early COPD) 60.5 (Adv. COPD) /69.3
Figueiredo (2015)	COPD (Mod – Severe)	Obtain knowledge on experience of husbands &sons providing care to a family member	QUAL	PORTUGAL	Interviews Purposive	12 (M) (> 4 years)	70.9(Husbands) 43.4 (Sons)/72.1
Grigorovich (2017)	HF (NYHA II-IV)	To examine changes in cg's wellbeing over time. Identify pt and cg factors associated with positive and negative outcomes	QUANT (Repeated measures)	CANADA	Longitudinal Convenience	50 (31F, 19M) (Mean = 18 months)	58/61.6

For peer review only - http://bmjopen.bmj.com/site/about/guidelines.xhtml

First Author/Year	Diagnosis	Aims (as stated by authors)	Methods	Country	Data Collection Sampling	Participants Caregiver (Time caring)	Mean Age (Caregiver /Recipient)
*Halm et al. (2006) *Halm et al. (2007)	CAD	To determine cg burden after CABG surgery	QUANT	USA	Cross Sectional Convenience	166(136F, 30M) (≤12 months)	64.7/66.8
*Halm (2016) *Halm (2017)	CAD	To describe the concerns needs, strategies and advice of CABG cg's during the first 3 months post- surgery To explore cg burden by age and gender	QUAL	USA	Interviews Purposive	32(16F, 16M) (N/S)	60.6 (M<70) /60.1 61.5 (F<70) /62.5 75.9(M>70) /74.4 73.6(F>70) /77.6
Hess (2009)	HF	To examine the association between cg literacy and medication administration	QUANT (Correlational)	USA	Cross Sectional Convenience	5 (F) (N/S)	65/72.8
Hooley (2005)	HF (NYHA III or IV)	To explore if greater cg burden is associated with increasing disease burden and depressive symptoms in pts and cg	QUANT	CANADA	Cohort Study Convenience	50 (40F, 10M) (N/S)	61/72

For peer review only - http://bmjopen.bmj.com/site/about/guidelines.xhtml

First Author/Year	Diagnosis	Aims (as stated by authors)	Methods	Country	Data Collection Sampling	Participants Caregiver (Time caring)	Mean Age (Caregiver /Recipient)
Hwang (2011)	HF (NYHA I – IV)	To identify factors associated with the impact of caregiving	QUANT	USA	Cross Sectional Convenience	76 Dyads (54F, 22M) (Mean = 53.4 months)	53.4/53.8
Hynes (2012)	COPD	To explore the experiences of cg's providing care to a family member with COPD	QUAL (Phenomenological)	IRELAND	Interviews Convenience	11(9F, 2M) (1 – 15 years)	N/S/N/S
Imes (2011)	HF (NYHA III-IV)	To describe cg's experience of living with HF	QUAL (Descriptive)	USA	Interviews Convenience	14(11F, 3M) (N/S)	64.8/68
Karmilovich (1994)	HF (NYHA III OR IV)	To examine cg demands and components of caring. Assess stress and correlation with cg burden. Assess relationship of	QUANT (Correlational)	USA	Descriptive survey Purposive	41 (30F, 11M) (N/S)	56.7/N/S
Kitko (2010)	HF	To gain a deeper understanding the type of work in spousal caregiving	QUAL	USA	Interviews Convenience	20(14F, 6M) (2 months – 9 years)	67/70

For peer review only - http://bmjopen.bmj.com/site/about/guidelines.xhtml

First Author/Year	Diagnosis	Aims (as stated by authors)	Methods	Country	Data Collection Sampling	Participants Caregiver (Time caring)	Mean Age (Caregiver /Recipient)
Kneeshaw (1999)	CAD	To examine cg mutuality and preparedness for caring post CABG surgery.	QUANT	USA	Longitudinal Convenience	49 (32F, 17M) (N/S)	50.1/72.6
Liljeroos (2014)	HF	To understand perceived caring needs in dyads and understand areas of support for cgs	QUAL	SWEDEN	Focus Groups Convenience	19Dyads (7F, 12M) (N/S)	70/72
Lindqvist (2013)	COPD (Mild to severe)	To describe conceptions of daily life for women caring for men with COPD	QUAL (Phenomenological)	SWEDEN	Interviews Purposive	21(F) (N/S)	72/N/S
Loftus (2004)	HF (NYHA II-IV)	Investigate outcomes of caregiving in late stage HF	QUANT (Correlational)	UK	Longitudinal Convenience	53 (41F, 12M) (6.66 hours per day)	66.7/76.3
Lum (2014)	HF (NYHA II-IV)	Measure of relationship quality and cg benefit, burden and depressive	QUANT (Correlational)	USA	Cross Sectional Purposive	19 (7F, 12M) (<1 - >8 hours per week)	59/69

For peer review only - http://bmjopen.bmj.com/site/about/guidelines.xhtml

First Author/Year	Diagnosis	Aims (as stated by authors)	Methods	Country	Data Collection Sampling	Participants Caregiver (Time caring)	Mean Age (Caregiver /Recipient)
Luttik (2007)	HF	Experiences and needs of cgs wellbeing factors	QUAL	NETHERLANDS	Interviews Convenience	13(10F, 3M) (N/S)	66/88.6
Luttik (2009)	HF (NYHA II-IV)	Investigate QOL in cg's of HF pts versus QOL in people with healthy partners	QUANT	NETHERLANDS	Cross Sectional Purposive	303 (N/S)	67/6
Marcuccilli (2011)	HF –LVAD in situ	Explore life adjustments of cg's caring for long term LVAD pts.	QUAL (Phenomenological)	USA	Interviews Convenience	5 (5F) (N/S)	56.6/N/S
Marcuccilli (2014)	HF-LVAD as DT	Explore experience of caring for family member with HF	QUAL (Phenomenological)	USA	Interviews Purposive	7(6F, 1M) (18 – 24 hours per day)	65/N/S
Miravitlles (2015)	COPD	Analyse burden of cg's	QUANT	SPAIN	Survey Representative (Mean = 12.7 hours daily, severe COPD)	22,795	56.5/72
Nakken (2017)	COPD	Investigate differences in male and female cg's and their perception of pts symptoms	QUANT (Correlational)	NETHERLANDS	Cross Sectional Convenience	188 Dyads (103F, 85M) (N/S)	65.4/63.3F 65.1/68.7M

For peer review only
For peer review only - http://bmjopen.bmj.com/site/about/guidelines.xhtml

First Author/Year	Diagnosis	Aims (as stated by authors)	Methods	Country	Data Collection Sampling	Participants Caregiver (Time caring)	Mean Age (Caregiver /Recipient)
Näsström (2017)	HF	Cg's participation & perspective of home care services	MIXED METHOD	SWEDEN	Interviews Purposive	15 (11F, 4M) (N/S)	77/N/S
Park (2013)	CAD	Difficulty and demands of cg tasks for older cg's of CABG pt.'s	QUANT	USA	Cross Sectional Convenience	35(29F, 6M) (Mean = 19days)	60/N/S
Pattenden (2007)	HF	Explore how pts and cgs cope with daily life with HF	QUAL	UK	Interviews Purposive	20(18F, 2M) (N/S)	67.8/N/S
Pi-Ming Yeh (2012)	HF	Explore burden on family cg's of older HF pts	QUANT (Correlational)	USA	Cross Sectional Purposive	50 (35F, 15M) (< 6 months - > 1 year)	60.3/77.6
Pressler (2013)	HF (NYHA I-IV)	Examine changes in cg burden and HRQOL Determine different perceptions between cg's of low HF pts and cgs of high HF pts Estimate time spent on cg tasks	QUANT (Repeated Measures)	USA	Longitudinal Convenience	65(48F, 17M) (Mean = 9.3 years)	59.7/69

For peer review only - http://bmjopen.bmj.com/site/about/guidelines.xhtml

First Author/Year	Diagnosis	Aims (as stated by authors)	Methods	Country	Data Collection Sampling	Participants Caregiver (Time caring)	Mean Age (Caregiver /Recipient)
Riegner (1996)	COPD	To understand QOL and its association with role strain, humour and support in cg's and pt's.	QUANT (Correlational)	USA	Cross Sectional Convenience	83(Dyads) (50F, 33M) (N/S)	63.2/65.6
Rolley (2010)	CAD	Describe experience of cgs of pt.'s undergoing percuatenous coronary intervention	QUAL	AUSTRALIA	Focus Groups Convenience	18 (F) (N/S)	N/S/N/S
*Saunders (2009) *Saunders (2008)	HF (NYHA I-IV)	To determine indicators of cg HRQOL	QUANT (Correlational)	USA	Cross Sectional Purposive	50 (42 F, 8M) (Mean = 5.9 years)	58.1/77.6
Saunders (2010)	HF	Compare employed and unemployed cg's on depression and wellbeing	QUANT	USA	Cross Sectional Convenience	41 (37F, 4M) (2.9 – 6 years)	59(Unemp.)/78 52(Emp.)/77
Schwarz (2003)	HF	Evaluate support on stress for cg's.	QUANT	USA	Cross Sectional Convenience	75 (55F, 20M) (Mean = 6 years)	63/N/S
Scott (2000)	HF	HRQOL of cg's and pts receiving community-based inotropic infusions	QUANT	USA	Cross Sectional Purposive	18 (16F, 2M)	63/69.3

For peer review only - http://bmjopen.bmj.com/site/about/guidelines.xhtml

First Author/Year	Diagnosis	Aims (as stated by authors)	Methods	Country	Data Collection Sampling	Participants Caregiver (Time caring)	Mean Age (Caregiver /Recipient)
Spence (2008)	COPD (Adv.)	Needs and experiences of family cg's	QUAL (Descriptive)	N.IRELAND	Interviews Purposive	7 (6F, 1M) (1 – 4 years)	N/S/N/S
Strom (2015)	HF	Next of kin's experience and responsibilities when caring	QUAL	NORWAY	Interviews Convenience	19 (17F, 2M) (N/S)	Median 63/NS
Takata (2008)	COPD (Long term O2 Therapy)	Explore cg burden	QUANT	JAPAN	Cross Sectional Convenience	45 Dyads (37F, 8M) (N/S)	68/75.2
Vellone (2015)	HF (NYHA I-IV)	Examine cg self-efficacy and contribution to pt self-care	QUANT (Correlation)	ITALY	Cross Sectional Convenience	515 Dyads (270F, 245M)	56.6/75.6
Wallin (2013)	CAD	To describe cg's need for support and information and impact after a cardiac event	QUAL (Descriptive)	SWEDEN	Interviews Purposive	20 (14F, 6M) (N/S)	55/N/S
Woolfe (2007)	COPD	Identify needs of cg's and how this impacts cg wellbeing	QUANT (Descriptive)	AUSTRALIA	Cross Sectional Convenience	63(39F, 24M) (N/S)	N/S/N/S

For peer review only - http://bmjopen.bmj.com/site/about/guidelines.xhtml

First Author/Year	Diagnosis	Aims (as stated by authors)	Methods	Country	Data Collection Sampling	Participants Caregiver (Time caring)	Mean Age (Caregiver /Recipient)
Wingham (2015)	HF	Identify needs of cg's to inform development of a caregiver resource for use in a home- based self-management intervention	QUAL	UK	Interviews (I)<br>Focus Groups (FG)<br>Purposive	22 (16F, 6M)<br>(6 months – 8 years)	(I)67/N/S<br>(FG)62 /N/S

For peer review only - http://bmjopen.bmj.com/site/about/guidelines.xhtml

Study characteristics

Of the 54 studies, 21 were qualitative, 32 quantitative and 1 mixed methods. 34 focused on HF, 14 COPD and 6 CAD. The total number of caregiver participants was 26,453. Caregivers were primarily female (63%), with a median age of 62 years. Patient median age was 69. A summary of study characteristics is provided in Table 3.

For peer review only

For peer review only - http://bmjopen.bmj.com/site/about/guidelines.xhtml

Table 3

Summary of Study Characteristics	n = 54 Studies	
**Aetiology of patients**		
CAD n (%)	6 (11)	
HF n (%)	34 (63)	
COPD n (%)	14 (26)	
**Caregiver Participants***		**Patients ***
Median age (range)	62 (43-77)	69 (36-93)
Median % of female (range)	63% (5–270)	38% (1-229)
**Relationship between patient and caregiver (n = 26,008 caregivers)**		
Spousal/Partner n (%)	2,321 (9)	
Son/Daughter n (%)	610 (2)	
Sibling n (%)	22 (<1)	
Parent n (%)	10 (<1)	
Friend/Relative n (%)	228 (<1)	
Not stated	22,961 (88)	
**Type of study**		
Qualitative n (%)	21 (39)	
Quantitative n (%)	32 (59)	
Mixed n (%)	1 (2)	
**Study Design**		
Cross-Sectional n (%)	24 (44)	
Longitudinal n (%)	4 (7)	
Cohort n (%)	2 (4)	
Quant. (survey) n (%)	2 (4)	
Qual. (Interview/ Focus Group) n (%)	16 (31)	
Phenomenological n (%)	5 (8)	
Inductive n (%)	1 (2)	
**Continents of publication**		
Europe n (%)	22 (41)	
North America n (%)	29 (54)	
Australasia n (%)	3 (5)	
**Date of publication**	**n= 57 Publications●**	
1990-1995	2	

For peer review only - http://bmjopen.bmj.com/site/about/guidelines.xhtml

For peer review only

1996-2001	3
2002-2007	10
2008-2013	22
2014-2017	20

For peer review only

Quality assessment

For peer review only - http://bmjopen.bmj.com/site/about/guidelines.xhtml

Studies of insufficient quality were excluded, qualitative papers were appraised and only high quality qualitative studies were included.[36] A total of 21 out of 193 qualitative studies were classified as high quality. Quantitative studies were classified as follows; 3 high quality, 12 medium quality and 17 low quality. See Table 4(a) and 4(b) for quality appraisal tables. Given of the number of high quality qualitative studies and in accord with current guidelines for the synthesis of qualitative evidence, we limited inclusion to qualitative studies of high quality only.[36] In contrast, given the low number of number of high quality quantitative studies, to ensure comprehensiveness of our review, we included all quantitative studies, regardless of quality.

For peer review only

For peer review only - http://bmjopen.bmj.com/site/about/guidelines.xhtml

Table 4(a)

	Design	Recruitment	Data Collection	Data Analysis	Findings	Total
Andersen (2015)	1	1	1	0	1	**4 [H]**
Baker (2010)	1	0	1	1	1	**4 [H]**
Bove (2016)	1	1	1	1	1	**5 [H]**
Burke (2014)	1	1	1	1	1	**5 [H]**
Clark (2008)	1	1	1	1	0	**4 [H]**
Figueiredo (2015)	0	1	1	1	1	**4 [H]**
•Halm (2016)	1	1	1	1	1	**5 [H]**
•Halm (2017)	1	1	1	1	1	**5 [H]**
Hynes (2012)	1	1	1	0	1	**4 [H]**
Imes (2011)	1	1	1	1	1	**5 [H]**
Kitko (2010)	1	1	1	1	1	**5 [H]**
Liljeroos (2014)	1	1	1	1	1	**5 [H]**
Lindqvist (2013)	1	1	1	1	1	**5 [H]**
Luttik (2007)	1	1	1	1	1	**5 [H]**
Marcuccilli (2011)	1	0	1	1	1	**4 [H]**
Marcuccilli (2014)	1	1	1	1	1	**5 [H]**
*Näsström (2017)	1	1	1	1	1	**5 [H]**
Pattenden (2007)	1	1	1	1	0	**4 [H]**
Rolley (2010)	1	1	1	1	0	**4 [H]**
Spence (2008)	1	0	1	1	1	**4 [H]**
Strom (2015)	1	1	1	1	1	**5 [H]**
Wallin (2013)	1	0	1	1	1	**4 [H]**
Wingham (2015)	1	1	1	1	1	**5 [H]**

For peer review only - http://bmjopen.bmj.com/site/about/guidelines.xhtml
For peer review only

Table 4(b)

**First Author**	**Study design**	**Participant sampling**	**Participant attrition**	**Outcome Measures**	**Data Analysis**	Overall score
Ågren (2010)	CS	Purp [+1]	0% [+1]	Non-V	MV [+1}	**3 [M]**
Al-Rawashdeh (2017)	CS	Purp [+1]	NS	V [+1]	MV [+1]	**3 [M]**
Badr (2017)	CS	Con	15.5% [+1]	Non-V	MV [+1]	**2 [L]**
Bakas (2006)	CS	Con	NS	V [+1]	MV [+1]	**2 [L]**
Chung (2016)	CS	Con	NS	V [+1]	UV	**1 [L]**
Cossette (1993)	CS	Con	NS	V [+1]	MV [+1]	**2 [L]**
Evangelista (2002)	CS	Con	20% [+1]	V [+1]	MV [+1]	**3 [M]**
Figueiredo (2013)	CS	Con	17% [+1]	V [+1]	MV [+1]	**3 [M]**
Figueiredo (2014)	CS	Con	11% [+1]	Non-V	MV [+1]	**2 [L]**
Grigorovich (2017)	LS [+1]	Con	NS	V [+1]	MV [+1]	**3 [M]**
• Halm (2006)	CS	Con	64%	V [+1]	MV [+1]	**2 [L]**
• Halm (2007)	CS	Con	64%	V [+1]	MV [+1]	**2 [L]**
Hess (2009)	CS	Con	NS	V [+1]	MV [+1]	**2 [L]**

For peer review only - http://bmjopen.bmj.com/site/about/guidelines.xhtml

Hooley (2005)	CS	Con	0% [+1]	V [+1]	UV	**2 [L]**
Hwang (2011)	CS	Con	35%	V [+1]	MV [+1]	**2 [L]**
Karmilovich (1994)	CS	Purp [+1]	24%	V [+1]	MV [+1]	**3 [M]**
Kneeshaw (1999)	LS [+1]	Con	32.7%	V [+1]	MV [+1]	**3 [M]**
Loftus (2004)	LS [+1]	Con	36%	V [+1]	MV [+1]	**3 [M]**
Lum (2014)	CS	Purp [+1]	5% [+1]	V [+1]	MV [+1]	**4 [H]**
Luttik (2009)	CS	Purp [+1]	31%	Non-V	MV [+1]	**3 [M]**
Miravitlles (2015)	CS	Rand [+1]	0% [+1]	Non-V	MV [+1]	**3 [M]**
Nakken (2017)	CS	Con	58%	Non-V	MV [+1]	**1 [L]**
*Näsström (2017)	LS [+1]	Purp [+1]	7% [+1]	V [+1]	MV [+1]	**5 [H]**
Park (2013)	CS	Con	NS	V [+1]	UV	**1 [L]**
Pi-Ming Yeh (2012)	CS	Purp [+1]	39%	V [+1]	MV [+1]	**4 [H]**
Pressler (2013)	LS [+1]	Con	16% [+1]	V [+1]	MV [+1]	**4 [H]**
Riegner (1996)	CS	Con	71.8%	V [+1]	MV [+1]	**2 [L]**
•Saunders (2008)	CS	Purp [+1]	36%	V [+1]	MV [+1]	**3 [M]**
•Saunders (2009)	CS	Purp [+1]	36%	V [+1]	MV [+1]	**3 [M]**

For peer review only - http://bmjopen.bmj.com/site/about/guidelines.xhtml

Saunders (2010)	CS	Con	NS	V [+1]	UV	**1 [L]**
Schwarz (2003)	CS	Con	NS	V [+1]	MV [+1]	**2 [L]**
Scott (2000)	CS	Purp [+1]	10% [+1]	Non-V	MV [+1]	**3 [M]**
Takata (2008)	CS	Con	NS	V [+1]	UV	**1 [L]**
Vellone (2015)	CS	Con	NS	V [+1]	MV [+1]	**2 [L]**
Woolfe (2007)	CS	Con	37%	V [+1]	UV	**1 [L]**

For peer review only

Findings

For peer review only - http://bmjopen.bmj.com/site/about/guidelines.xhtml

Six concepts relating to caregiver experience were identified, (1) mental health, (2) caregiver role, (3) lifestyle change, (4) support for caregivers, (5) knowledge and (6) relationships. Four additional concepts were identified from qualitative papers only (6) expert by experience, (7) vigilance, (8) time and (9) shared-care (See Figure 2). The concepts are reflected in caregiver quotes in Table 5.

For peer review only

Figure 2

For peer review only - http://bmjopen.bmj.com/site/about/guidelines.xhtml

For peer review only

Table 5

For peer review only - http://bmjopen.bmj.com/site/about/guidelines.xhtml

**Mental Health**	*"The mental strain is difficult. I feel so trapped"*<br><br>*""You fall into a huge hole, then the world gets so tiny, it all gets so narrow that it is almost unbearable"*<br><br>*"I feel like sleeping beauty. The hawthorn hedge has closed around me, and I cannot do anything about it."*
**Role**	*"I can sum my role up in three words, I am a cheerleader, drill sergeant, and negotiator"*
**Lifestyle Change**	*"Our life has come down. The two of us used to go out dancing. We loved dancing and then it all stopped."*
**Knowledge**	*"I wish I had had more education on the 'what ifs'. When I was leaving the hospital nobody really said, "OK now this is what's going to happen and this is what you're going to have to do." If there would've been any kind of complications I would've been totally in the dark. I didn't know all the things I needed to know."*
**Relationships**	*"I just love him and I find that every day when I see him, what else could I do to try and make him a wee bit … better? It's very satisfying to know that he appreciates what I do and it's nice to know that you are helping someone."*<br><br>*""It's like having another child sometimes because you are sort of responsible and I feel he is my responsibility. I feel that he is not anybody else's responsibility…"*

For peer review only

For peer review only - http://bmjopen.bmj.com/site/about/guidelines.xhtml

**Support**	***"And then I really felt alone in it all. Because everybody would call and come over and ask, how is John? Hardly anyone asked "how are you doing"?*** <br>***"Doctors (do) not realize that 1 day your life is jut normal and then this comes and smashes everything to bits, you know- and there are so many questions."*** <br>***"I would be lost without, our heart failure nurse, and, all the other input we've had from all the other professionals, like the podiatrist and GP. . .. . . You can do it, but in partnership with everybody else."***
**Vigilance**	***"Every morning I put my ear to his chest and listen to his heart, that is how we first discovered he was in atrial fibrillation so now I do it every morning before I leave. I monitor him very closely and there are days in which I do not feel comfortable leaving for work so those days I work at home. I call everyday from work and we have our routine, if I am not aware of anything he had planned for the day, I then immediately call my neighbour to check on him"***
**Shared Care**	***"There were days I thought to myself, where are we going from here? But we mastered it together and tried to do things at his pace."***
**Time**	***"At first it was overwhelming. I didn't think I could do it. When they first told me I was like, "I can't do that," you know. And then they explained to me, like, yes you can. It's like getting a new baby. You know, you learn how to take care of them step by step and then it's just part of the routine. And that's really the way it was"***
**Expert by Experience**	***"It's so frustrating when she goes into hospital and the nurses and the doctors say it's her condition, you know. I'm like I'm with her twenty-four hours a day, I know how breathless she is without***

For peer review only - http://bmjopen.bmj.com/site/about/guidelines.xhtml

	infection and I know how breathless she is with an infection there's a major difference" <br>***"I see him every day, they are just little subtle changes, they are not showing up in the numbers the doctors are concerned with but I see it"***

1. Mental Health

Twenty-five quantitative, [35,37-62] twenty qualitative [63-83] and one mixed method[84] study addressed mental health. This encompassed depression and burden. Caregivers described an internal and external conflict of emotions, recognising a psychological change within themselves and the care recipient. Maintaining hope and positivity, versus managing worries, fears and anxieties was predominant.[62-82] Pressler's (2013) HF study; identified caregivers had moderately poor health at baseline and at 8 months but they had fewer depressive symptoms over time.[54] Burden arose due to greater responsibilities.[65, 68,73,81,82] Pi-Ming Yeh (2012), noted the quality of relationship and lack of family support significantly predicted greater family caregiver burden.[53] Näsström (2017) reported, caregiver burden was concerned with the future and their fears of potential demands.[84] Those with greater resiliency appeared to adjust and cope better with the illness trajectory.[64, 65, 76, 77] Caregivers described mental adjustment after an acute event.[77] Living through an acute event was long lasting and some experienced post traumatic symptoms.[80]

2. Caregiver Role

This is addressed in eighteen qualitative [64-72,74,75,77-83,85] and fourteen quantitative studies.[35,39-41, 49-51, 54, 57, 59, 60, 86, 87, 88,89] Caregiver role is complex and requires much co-ordination. [74, 81, 83] Caregivers describe significant role change such as increasing domestic tasks. [63, 66, 69,, 71,76 -79, 82] Role loss is prevalent [64, 65, 70] and caregivers need to reframe their identity.[72, 80] Societal expectation regarding the relationship and gender, influences caregivers adjusting to their roles.[65, 67, 68, 74, 79] Caring can be positive and rewarding. Caregivers learn about themselves and strengths they have. [65, 75-77, 80, 81, 83] Pressler (2013), describes the tasks involved; domestic, emotional support, managing dietary needs and transport.[54] Pressler also reports caregivers of persons with greater HF symptoms experience more difficulty with their role.[54]

3. Lifestyle Changes

For peer review only - http://bmjopen.bmj.com/site/about/guidelines.xhtml

Fourteen quantitative [35,38-40, 43, 45, 52,54, 59, 60, 62, 87,88, 90] and twenty-one qualitative[63-83,85] studies addressed lifestyle changes. Caregivers experienced leisure, social and work related problems. [35,39,90] Caring interrupted and eliminated tasks from their routine.[35,39,59] Contrastingly, Pressler (2013) reported caregivers perceptions of how their lives changed as a result of caregiving was neutral and improved from baseline to 4 and 8 months.[54] Caregivers became adaptable in their new role.[72,80,85] There was less personal time for leisure and hobbies either alone or with the care recipient[67-70,76,77,82,83] Caregivers described daily "ups & downs" and had to adjust their routines dependent on the presentation of the care recipient. [63,64,66,71,73,75,78,79,81,83]

4. Support for caregivers

Fifteen quantitative[41, 45-47, 49, 51-53,55,56,58-60, 62,87,89], twenty-one qualitative[63-83,85] and one mixed method[84] study examine support. This includes healthcare, family and social support. The weight of perceived external expectations, the necessity of being pro-active in obtaining support and maintaining a social role was described across all diagnoses.[45,46,48-50, 53-57, 61, 89] Pi-Ming Yeh (2012) identified lack of family support as a significant issue.[53] Caregivers felt abandoned by healthcare teams. After hospital discharge they had to provide care without advice or medical support.[66, 72, 78] Positive interactions were reported. Namely access to healthcare professionals via telephone or home support.[63, 64, 77, 84]

5. Knowledge

This was addressed in seventeen qualitative[63,65-75,77,79,80,82,83,85] five quantitative[50,60-62,87] and one mixed method[84] study. This describes caregivers understanding of the diagnosis and need for knowledge throughout the duration of illness. [63,67,70,75,83,84,85] Caregivers report information from health professionals was often inadequate. [71, 73, 74] Timing and format of information was significant. Caregivers received information verbally or by leaflets in hospital but describe being left alone to provide care in the long term [65, 68,69,79,82] Caregivers had difficulty understanding how to navigate the care system. [72, 80] They had to make decisions without full knowledge of the consequences of their decision making, particularly during acute exacerbations.[65] The quantitative element of Näsström's (2017) mixed methods study correlated with qualitative studies; receipt of sufficient information was central to managing HF and was associated with better perceived health of caregivers.[84]

6. Relationships

For peer review only - http://bmjopen.bmj.com/site/about/guidelines.xhtml

For peer review only

Twenty qualitative,[64-83,85] twenty-two quantitative[36-38, 42-44,46-52,54-57,61,87,90,91,92] and one mixed method[84] study examined relationships. In HF studies caring for individuals with more symptoms resulted in poorer perceived experiences.[54,91] Higher relationship quality resulted in less burden and more benefit from the relationship. The relationship prior to diagnosis influenced the current relationship. Perspective of the relationship was either a sense of duty [65,74,80,81,84] or this was a valuable second chance.[66,75,82,83] Caregivers reported difficulty communicating about the condition leading to isolation, stress and conflict between caregiver and care recipient.[71,73] The relationship requires negotiation.[69,85] Caregivers prioritised the care recipient over their own needs. [64, 72,74,77,82]

7. Expert by Experience

Twelve qualitative studies[65-70,72,75,76,80,81,83,85] addressed this concept. Caregivers learnt new skills. They became "experts by experience" discovering through "doing" and observing health professionals.[66,68,83] They developed "proto-professional skills"; in medication administration [65,80,85] judging care recipients level of functioning [79] and decision making in times of exacerbations.[70] Caregivers observed the nuances of change in the care recipient often not perceived by healthcare teams or other family members such as skin colour or irritability.[72,75,81]

8. Vigilance

Vigilance was recurring in caregivers' narrative across all diagnoses and was present in nineteen qualitative studies.[64-80,81,83,85] Caregivers were always on the alert observing the care recipient. [66,67,70,72-74,77-79,81] They lay awake at night listening for their partners' breath.[69,71,75,85] This impacted on caregivers' health creating constant fatigue, worry and stress.[65,79] Caregivers recognised that the need for vigilance came from themselves and their insecurities. [64, 76, 83]

9. Time

Time explores how caregivers adjusted to living with the illness and was present in fifteen qualitative studies.[65,67-77,80,82,83,85] Caregivers adapted to a new life, referring to "then", how life was and "now" their current life.[69, 70,75,76,83] The duration of caregiving and severity of illness influenced caregiver's ability to adjust.[66, 73, 76] Caregivers lived day by day [83] and viewed the future, with hope or uncertainty about what lay ahead. [65, 70,72,79,82]

10. Shared Care

Shared care was present in sixteen qualitative studies.[63-66,68-76,80,81,83,85] This demonstrates caregiver and care recipient working together managing the illness. Jointly administering medication [68, 81] and

For peer review only - http://bmjopen.bmj.com/site/about/guidelines.xhtml

attending appointments.[73] The presence of illness was a process they adjusted to together. [76, 80] Caregivers referred to themselves and the care recipient as "we", when discussing dealing with the illness.[63,71,75] The mutual perspective between caregiver and care recipient served to isolate them from the world, the illness was *"taking a life of its own…it's like this third person"*, (Hynes, 2012, p. 1071)

There were differences in caregiver experience for each of the diagnoses and these are discussed below.

Heart Failure

HF caregivers experienced an "ebb and flow" in caring, an underlying worry, fear and anxiety which at times of change or illness heightened.[49,51, 59,71,77, 81, 83,85,92] Lifestyle changes were long lasting and sustained.[39, 59, 64, 71, 77,81, 83,85,92] Obtaining knowledge was necessary throughout all stages of the illness. [50, 63, 66, 73, 83, 85, 92] Sourcing information and communication with health professionals was often difficult. [63, 66, 71, 85, 92] In spousal relationships they predominantly viewed the care recipient as another child or as a "duty." [50, 51, 64, 66, 71, 73, 77, 84]

Chronic Obstructive Pulmonary Disease

COPD caregivers experienced a prolonged impact on their mental health similar to HF caregivers. [41, 44, 52,60, 65,70,80] Severity of illness was influential on their experience of burden.[38,43,44,60] Role change was long lasting and profound for many.[70,80,65,67] They expressed concerns with perceived lack of knowledge.[62,65,70,74,80] During exacerbations COPD caregivers experienced anxiety and fear of their loved one dying.[65,67,70,74] COPD caregivers highlighted the loss of social roles while trying to maintain their loved ones dignity.[65,74,70,80,89,90] The coughing and spitting associated with COPD often left the care recipient embarrassed.[67,65,80] The caregiver tried to avoid situations where this would happen. The dynamics of spousal relationships changed, caregivers described losing the intimate love they had for their partner.[65,70,74, 80]

Coronary Artery Disease

Caregivers of CAD patients experienced intense role change on discharge from hospital and in the acute phase of illness.[47,68,79,82,87,88] They initially engaged with a high volume of tasks which reduced over time.[46,47,68,82,79,87,88] CAD caregivers experienced post-traumatic type symptoms if they witnessed the recipient experience an acute event.[79,82] Caregivers described being unable to talk about this and reliving the event in their heads. Anxiety did ease over time for many.[79,82] Caregivers

For peer review only - http://bmjopen.bmj.com/site/about/guidelines.xhtml

felt unprepared at hospital discharge and highlighted not realising how much their routine would be disrupted.[68, 79,82] Caregivers reported viewing the experience as a second chance and had a renewed sense of love and appreciation for the relationship.[79,82]

DISCUSSION

This mixed methods systematic review demonstrates the similarities and differences in caregiver experiences across three common cardio-respiratory conditions. It highlighted the differences in experiences obtained from qualitative and quantitative research. Commonly occurring experiences included the exacerbation of caregiver physical and mental health due to the role. This correlates with Kang et al.'s (2011) systematic review of HF caregivers identifying that caregiving resulted in a multitude of changes in caregiver's lives regardless of age, gender and ethnicity.[93] Addressing both patient and caregiver needs in order to maintain wellbeing for both is important[19] and recognises the value of "shared-care" between patient and caregiver. The prevalence of mental health needs in this review demonstrates the need for psychosocial support for caregivers. This concurs with AasbØ et al. (2016), identifying caregivers need to be in "emotional control"[94] and Wingham et al. (2017) describing the "enduring anguish" experienced by caregivers.[95] Lawton et al. (1991) attribute caregiver well-being to the commitment of the caregiver to the role and dealing with competing demands which can increase burden and negatively impact affect. Spousal caregivers may be more ready to accept the role of caregiving than adult children who may view it as an imposition on an already established lifestyle.[96] In this review societal expectations had an impact on how caregivers adjust to their role. Additionally, the quality of the relationship prior to becoming a caregiver had an influence on the caregiver subjective experience of burden.

Caregivers had predominantly negative experiences of support and described uncertainty of how to obtain this. Caregivers' need greater support and knowledge transfer to conduct their role.[97] They should be included in clinical appointments[98] to ensure they are not isolated in providing care and to allow for knowledge exchange. Caregivers in Giacomini et al.'s (2012) review of living and dying with COPD reported increasing isolation in addition to their own health issues.[18] They described pressure balancing their variety of roles; similar experiences to the caregivers in this synthesis across all diagnoses. Caregivers emphasised their need to be vigilant. This falls into five categories as defined by Feeney Mahoney's (2003) study of Alzheimer's caregivers; "watchful supervision", "protective intervening", "anticipating", "on duty" and "being there". Caregivers in this synthesis described overt vigilance, putting one's head on the chest of the recipient to check breathing or covert vigilance; observing them throughout the day.[99] Healthcare professionals must be aware of these levels of vigilance and the constant presence of them to support caregivers in their role.

For peer review only - http://bmjopen.bmj.com/site/about/guidelines.xhtml

Caregivers are valuable providers of care. Caregiver's needs should be assessed systematically and in a formalised manner in healthcare settings.[16] When developing collaborative models of care the inclusion of caregivers is imperative.[100]

Strengths and limitations

This review demonstrates the complexity of what it means to be a caregiver and should inform clinical care development of interventions. A mixed methods review can be contentious[101] due to the synthesis of differing paradigms. In this review it required transformation of quantitative data into qualitative data.[15,102] We aimed to present a convergence of caregiver experiences by conducting a mixed methods synthesis. However, it demonstrated four differing concepts between the two paradigms. This highlights the challenge of synthesising multiple methods. It is worth exploring how the four additional qualitative concepts could be captured quantitatively in order to inform healthcare intervention. This mixed methods synthesis is, to our knowledge the first to combine caregiver experiences in HF, COPD or CAD. It examines the differences and similarities in experiences, establishing a comprehensive assessment of the knowledge base of caregiver experiences in common cardiorespiratory conditions.

There are limitations to this study; both in our review methods and the nature of included studies. First, we acknowledge that the inclusion of lower quality quantitative studies may lead to risk of bias: the majority of quantitative studies utilised convenience sampling, had a high attrition and low response rate. Non-validated outcome measures were utilised in some quantitative studies with the majority of studies conducting univariate rather than multivariate analysis. However, given the limited number of high quality quantitative studies (4 studies) we believe this broader inclusion increased the scope of our review in order to achieve a holistic understanding of caregiver experiences. Furthermore, we would note that the conclusions of this review were broadly the same with consideration of only the high quality quantitative studies. Second, studies were restricted to English language only, from high income countries and excluding caregivers of nursing home residents. This may limit the applicability of findings to other settings. Third, converting quantitative data into a qualitative data set risks the quantitative data set being oversimplified. This was managed with regular research team meetings to review each stage of this process. Fourth qualitative synthesis is an interpretation of other researcher's interpretations. To minimise individual interpretative bias a second researcher was utilised to seek confirmation of the results. Finally,

For peer review only - http://bmjopen.bmj.com/site/about/guidelines.xhtml

For peer review only

included qualitative and quantitative studies were primarily cross-sectional in design, therefore considering caregiver experience only at a single point of time.

Implications for practice and future research

There are a number implications following this review. It has demonstrated there are similarities and differences in the caregiver experience in HF, COPD or CAD. The impact on caregiver's lives of those with HF and COPD appears longer lasting and more turbulent than caring for CAD patients. CAD caregiver's experience of hospital during exacerbations increased distress at discharge. This review reflects the complexity of the caregiver's role. The mixed method approach indicted differences in what is being investigated. This is important in demonstrating an understanding of the caregiver experience when dealing with complex conditions. Future research should focus on involving caregivers in the design and delivery of interventions for patients with cardiopulmonary disease. Best practice interventions for CAD caregivers in the discharge process from hospital to home must be formalised. There appears to be a focus on the mental health of caregivers of those with HF, however, further research is needed to explore this in COPD and CAD caregivers. Exploration of this via support groups for caregivers of cardiorespiratory conditions is merited. Clinically, the healthcare team need to identify who the caregiver is and be aware of their needs with the use of a carer's needs assessment. There must be a greater understanding of caregiver support needs, what they avail of; and are they aware of what is available to them in the community. This can be achieved in conversation between the healthcare team and caregivers and warrants further research as to how and whether caregivers avail of external supports.

Consideration needs to be given as to whether quantitative research tools to explore caregiver expertise, view of the future, experience of shared care and vigilance can be developed to capture these qualitative concepts to inform the development of self-management interventions for patients and caregivers. Repeated measures examining perceived control and caregiver needs may contribute to a greater understanding of caregiver experiences which arose in qualitative studies. Additionally, longitudinal studies with repeated assessment need to be conducted to assess the stability of caregiver experiences and whether they are liable to much change over time. In this review only four out of thirty-two quantitative studies examined caregiver's experiences longitudinally. Understanding whether there are caregiver changes over time will facilitate greater understanding of caregiver needs for health professionals when working with this population. The emergence of additional concepts from qualitative studies emphasises the role of mixed methods

For peer review only - http://bmjopen.bmj.com/site/about/guidelines.xhtml

research when examining lived experiences. The additional concepts also demonstrated the nuanced expertise of the caregiving experience. It is important for researchers to consider how to reflect this in quantitative investigation so as to inform funders in order to develop and trial interventions in HF, COPD and CAD. The quality of quantitative studies in COPD and CAD were medium or low. There is a need for more empirically robust studies examining the experiences of these caregivers. Additionally, greater understanding of caregiver's experiences with this population will facilitate the development of robust evidence based guidelines for health services when working with HF, COPD and CAD.

CONCLUSIONS

This mixed methods systematic review provides a holistic synthesis of caregiver experiences of people with HF, COPD or CAD. It demonstrates there are a number of implications when an individual becomes a caregiver for those with chronic cardiopulmonary disease. Caregivers re-frame their identity and change their life course. Caregivers learn a multitude of skills and develop expertise in their new role. Their expertise is invaluable and should be acknowledged in healthcare interventions for these conditions. The quality of evidence was limited by assessment of caregiver experience at single time point. There is need for future studies that employ longitudinal designs examining the change in caregiver experience over time. Caregiving can be positive if caregivers have access to support, are well informed and part of the healthcare team. Understanding the experiences of caregivers for people with these conditions allows healthcare professionals and policy makers to reflect on our approach. Health services must consider caregivers in the design and delivery of interventions.

Contributors: MN, JW, RST: conceived and designed the review and quality appraised the included studies. MN wrote the initial draft of the manuscript. JW AND RST provided feedback and edits to manuscript drafts.

Funding

This study was supported by a University of Exeter Postgraduate Studentship Grant

Copyright/license for publication

"The Corresponding Author has the right to grant on behalf of all authors and does grant on behalf of all authors, a worldwide licence to the Publishers and its licensees in perpetuity, in all forms, formats and media (whether known now or created in the future), to i) publish, reproduce, distribute, display and store the Contribution, ii) translate the Contribution into other languages, create adaptations, reprints, include within collections and create summaries, extracts and/or,

For peer review only - http://bmjopen.bmj.com/site/about/guidelines.xhtml

abstracts of the Contribution, iii) create any other derivative work(s) based on the Contribution, iv) to exploit all subsidiary rights in the Contribution, v) the inclusion of electronic links from the Contribution to third party material where-ever it may be located; and, vi) licence any third party to do any or all of the above."

Patient consent

Not applicable

Competing Interests

None declared

Data Sharing

Any requests for additional information about the study can be accessed via contacting the primary author.

Acknowledgements

We would like to thank the PenCLARHC Evidence Synthesis Team at University of Exeter Medical School**.** Katy Oaks and Catriona Organ, Librarians at Royal Cornwall Hospital Library, Knowledge Spa, Truro. Dr. Ruth Garside, Senior Lecturer in Evidence Synthesis, European Centre for Environment and Human Health for their assistance throughout the process of this review.

References:

1. Carers Trust (2015). Available from: https://carers.org/what-carer (Accessed 26th June 2017).
2. Wolff, J, Spillman, BC, Freedman, VA, Kasper, JD. A national profile of family and unpaid caregivers who assist older adults with health care activities. *JAMA Intern Med*. 2016; 176(3):372-379. Available from doi:10.1001/jamainternmed.2015.7664.
3. International Alliance of Carer Organizations. *Global carer facts*. Available from: http://www.internationalcarers.org/carer-facts/global-carer-stats/(Accessed 1st October 2017).
4. White, C. 2011 Census Analysis: Unpaid care in England and Wales, 2011 and comparison with 2001. *Office of National Statistics.* 2013. Available from: https://www.ons.gov.uk/peoplepopulationandcommunity/healthandsocialcare/healthcaresystem/articles/2011censusanalysisunpaidcareinenglandandwales2011andcomparisonwith2001/2013-02-15 (Accessed 10th October 2017).
5. Buckner, L, Yeandle, S. Valuing Carers 2015. The rising value of carers' support. *Carers UK 2015.* Available from *http://www.carersuk.org/for-professionals/policy/policy-library/valuing-carers-2015* (Accessed 7th July 2017).
6. The Institute of Public Care. *A Carer's Life: Implications and Considerations for Commissioning.* 2017. Available from: http://ipc.brookes.ac.uk/publications/A_carers_life.html (Accessed 30th September 2017).
7. Seltzer, MM, Wailing Li, L. The dynamics of caregiving: transitions during a three-year prospective study. *The Gerontologist.* 2000; 40(2):165–178. Available from doi.org/10.1093/geront/40.2.165.

For peer review only - http://bmjopen.bmj.com/site/about/guidelines.xhtml

8. The Care Act. Chapter 23. *The Care Act UK: The Stationery Office.* 2014. Available from: http://www.legislation.gov.uk/ukpga/2014/23/contents.enacted/data.htm (Accessed 11th October 2016).
9. National Institute for Health and Clinical Excellence. *Chronic heart failure in adults: management.* 2010. NICE guideline (CG108).
10. National Institute for Health and Clinical Excellence. *Chronic obstructive pulmonary disease in over 16s: diagnosis and management.* 2010. NICE guideline (CG101).
11. Wilkins, E, Wilson, L, Wickramasinghe K, Bhatnagar, P, Leal, J, Luengo-Fernandez, R, Townsend, N. European Cardiovascular Disease Statistics 2017. *European Heart Network*, Brussels. Available from: http://www.ehnheart.org/images/CVD-statistics-report-August-2017.pdf (Accessed 25th August 2017).
12. Center for Disease Control and Prevention. *Heart Disease Facts and Statistics*. Available from: www.cdc.gov (Accessed 9th November 2016).
13. Raherison, C, Girodet, PO. Epidemiology of COPD. *European Respiratory Review*. 2009;18: 213-221. Available from: doi:10.1183/09059180.00003609.
14. Nicholas Dionne-Odom, J., Hooker, S.A., Bekelman, D. et al. Heart Fail Rev (2017) 22: 543. Available from: https://doi.org/10.1007/s10741-017-9597-4.
15. Foster, F, Piggott, R, Teece, Beech, R. Patients with COPD tell their stories about living with the long-term condition: an innovative and powerful way to impact primary health care professionals' attitudes and behaviour? *Education for Primary Care*, 2016, 27:4, 314-319. Available from doi:10.1080/14739879.2016.1181699.
16. Farquhar, M. Assessing carer needs in chronic obstructive pulmonary disease. *Chronic Respiratory Disesae*. 2017, 1-10. Available from doi:10.1177/1479972317719086.
17. Mansfield, E, Bryant, J, Regan, T, Waller, A, Boyes, A, Sanson-Fisher, R. Burden and unmet needs of caregivers of chronic obstructive pulmonary disease patients: a systematic review of the volume and focus of research output. *Journal of Chronic Obstructive Pulmonary Disease. 2016;* 13(5):662-667. Available from: doi:10.3109/15412555.2016.1151488.
18. Giacomini, M, DeJean, D, Simeonov, D, Smith, A. Experiences of living and dying with COPD: a systematic review and synthesis of the qualitative empirical literature. *Ontario* Health *Technology* Assessment Series. 2012;12(13):1-47.
19. Doherty, L, Fitzsimons, D, McIlfatrick, S. Carers' needs in advanced heart failure: a systematic narrative review. European *Journal* of Cardiovascular Nursing. 2016; 15(4):203-212. Available from: doi: 10.1177/1474515115585237.
20. Stewart, M, Davidson, K, Meade, D, Hirth, A, Makrides, L. Myocardial infarction: survivors' and spouses' stress, coping, and support. *Journal of Advanced Nursing*. 2000; 31: 1351–1360. Available from: doi:10.1046/j.1365-2648.2000.01454.x
21. Stolarik A, Lindsay P, Sherrad H, Woodend K. Determination of the burden of care in families of cardiac surgery patients. Progress in Cardiovascular Nursing. 2000; 15:4–10
22. Falk, JA, Kadiev, S, Criner, GJ, Scharf, SM, Minai, OA, Diaz, P. Cardiac disease in chronic obstructive pulmonary disease. *Proceedings of the American Thoracic Society*. 2008;5(4): 543-548. Available from: doi:10.1513/pats.200708-142ET.
23. Müllerova, H, Mapel, DW. Cardiovascular comorbidity in COPD systematic literature review. *CHEST.* 2013;144(4):1163–1178. Available from: doi:10.1378/chest.12-2847.
24. Moher, D, Liberati, A, Tetzlaff, J, Altman, DG, The PRISMA Group. Preferred reporting items for systematic reviews and meta-analyses: The PRISMA statement. *PLoS Med.* 2009;6(7). Available from: doi:10.1371/journal.pmed.1000097.

For peer review only - http://bmjopen.bmj.com/site/about/guidelines.xhtml

25. Johnson, RB, Onwuegbuzie, AJ, Turner, LA. Toward a definition of mixed methods research. *Journal of Mixed Methods Research,* 2007;1:112–133. Available from: doi:10.1177/1558689806298224.
26. World Bank Data. Available from: http://www.worldbank.org/ (Accessed 10th June 2017).
27. Critical Appraisal Skills Programme. *Qualitative research checklist.* Available from: http://docs.wixstatic.com/ugd/dded87_25658615020e427da194a325e7773d42.pdf (Accessed 10th December 2016).
28. Sandelowski, M, Voils, CI, Barroso, J. Defining and Designing Mixed Research Synthesis Studies. *Research in the Schools : A Nationally Refereed Journal Sponsored by the Mid-South Educational Research Association and the University of Alabama.* 2006; 13(1):1-15.
29. Pluye, P, Hong, QN. *Annual Review of Public Health*. 2014. Available from: www.annualreviews.org (Accessed September 27th 2016).
30. Hong, QN, Pluye, P, Bujold, M, Wassef, M. Convergent and sequential synthesis designs: implications for conducting and reporting systematic reviews of qualitative and quantitative evidence. *Systematic Reviews*. 2017; 6(61). Available from: doi:10.1186/s13643-017-0454-2.
31. Tashakkori, A, Teddlie, C. Mixed Methodology: Combining Qualitative and Quantitative Approaches. *Applied Social Research Methods Series*. 1998; 46. SAGE Publications, Thousand Oaks, CA.
32. Noblit, GW. & Hare, RD. Meta-ethnography: synthesizing qualitative studies. 1988. SAGE Publications, Thousand Oaks, CA.
33. Tashakkori, A, Teddlie, C. Foundations of Mixed Methods Research: integrating quantitative and qualitative approaches in the social and behavioural sciences. 2009. SAGE Publications Thousand Oaks, CA.
34. Frantzen, KK, Fetters, MD. Meta-integration for synthesizing data in a systematic mixed studies review: insights from research on autism spectrum disorder. *Qual Quant.* 2015;50:2251–2277. Available from: doi:10.1007/s11135-015-0261-6.
35. Chung, ML, Lennie, TA, Mudd-Martin, G, Dunbar, SB, Pressler, SJ, Moser, DK. Depressive symptoms in patients with heart failure negatively affect family caregiver outcomes and quality of life. *European Journal of Cardiovascular Nursing. 2016;*15 (1):30-38. Available from: doi:10.1177/1474515114535329
36. Dixon-Woods, M, Fitzpatrick, R, Roberts, K. Including qualitative research in systematic reviews: opportunities and problems. Journal of Evaluation in Clinical Practice. 2001; 7(2):125-133. Available from: doi:10.1046/j.1365-2753.2001.00257.x.
37. Ågren, S, Evangelista, L, Strömberg, A. Do partners of patients with chronic heart failure experience caregiver burden? *European Journal of Cardiovascular Nursing*, 2010;9(4):254–262. Available from: doi:10.1016/j.ejcnurse.2010.03.001.
38. Al-Rawashdeh, SY, Lennie, TA, Chung, ML. The association of sleep disturbances with quality of life in heart failure patient–caregiver dyads. *Western Journal of Nursing Research*, 2017;39(4):492–506. Available from: doi: 10.1177/0193945916672647.
39. Badr, H, Federman, AD, Wolf, M, Revenson, TA Wisnivesky, JP. Depression in individuals with chronic obstructive pulmonary disease and their informal caregivers. *Aging & Mental Health. 2017;*21(9): 975-982. Available from: doi:10.1080/13607863.2016.1186153.
40. Bakas, T, Pressler, SJ, Johnson, E, Nauser, J, Shaneyfelt, T. Family caregiving in heart failure. *Nursing Research.* 2006;55(3):180-188.
41. Cossette, S, Levesque, L. Caregiving tasks as predictors of mental health of wife caregivers of men with chronic obstructive pulmonary disease. *Research in Nursing & Health*, 1993;16:251-263.

For peer review only - http://bmjopen.bmj.com/site/about/guidelines.xhtml

42. Evangelista, l, Dracup, K, Doering, L, Westalke, C, Fonarow, GC Hamilton, M. Emotional well-being of heart failure patients and their caregivers. *Journal of Cardiac Failure*. 2002;8(5): p.300-305. Available from: doi:10.1054/jcaf.2002.128005.
43. Figueiredo, D., Gabriel, R., Jácome, C. & Marques, A. Caring for people with early and advanced chronic obstructive pulmonary disease: how do family carers cope? *Journal of Clinical Nursing.* 2013;23:211–220. Available from: doi: 10.1111/jocn.12363.
44. Figueiredo, D, Gabriel, R, Jácome, C, Cruz, J, Marques, A. Caring for relatives with chronic obstructive pulmonary disease: how does the disease severity impact on family carers? *Aging & Mental Health.* 2014;18(3):385-393. Available from: doi:10.1080/13607863.2013.837146.
45. Grigorovich, A, Lee, A, Ross, H, Woodend, K, Forde, S, Cameron, JI. A longitudinal view of factors that influence the emotional well-being of family caregivers to individuals with heart failure. *Aging & Mental Health*. 2017;21(8):844-850. Available from: doi:10.1080/13607863.2016.1168361.
46. Halm, MA, Jacobson-Treat, D, Lindquist, R. Correlates of caregiver burden after coronary artery bypass surgery. *Nursing Research*. 2006;55(6):426-436.
47. Halm, MA, Bakas, TB. Factors associated with caregiver depressive symptoms, outcomes, and perceived physical health after coronary artery bypass surgery. *Journal of Cardiovascular Nursing*. 2007;22(6):508-515. Available from: doi:10.1097/01.JCN.0000297388.21626.6c.
48. Hooley, PJD, Butler, G, Howlett, JG. The relationship of quality of life, depression, and caregiver burden in outpatients with congestive heart failure. *Congestive Heart Failure.* 2005;11(6):303-310. Available from: doi:10.1111/j.1527-5299.2005.03620.x.
49. Hwang, B, Fleischmann, KE, Howie-Esquivel, J, Stotts, NA, Dracup, K. Caregiving for patients with heart failure: impact on patients' families. *American Journal of Critical Care.* 2011;20(6):431-441. Available from: doi: 10.4037/ajcc2011472.
50. Karmilovich, SE. Burden and stress associated with spousal caregiving for individuals with heart failure. *Progress in Cardiovascular Nursing.* 1994;9(1):33-38.
51. Loftus, L. The influence of illness and psychological factors on patient and caregiver outcomes in heart failure. PhD [dissertation]. Liverpool: University of Liverpool; 2004.
52. Nakken, N, Janssen, DJA, van Vilet, M, de Vries, GJ, Clappers-Gielen, GAL, Michels, AJ, Spruit, MA. Gender differences in' partners of patients with COPD and their perceptions about the patients. *International Journal of COPD*. 2017;12: 95–104. Available from: doi:https://doi.org/10.2147/COPD.S118871.
53. Ping-Meh, Y, Bull M. Use of the resiliency model of family stress, adjustment and adaptation in the analysis of family caregiver reaction among families of older people with congestive heart failure. *International Journal of Older People Nursing.* 2012;7:117–126. Available from: doi:10.1111/j.1748-3743.2011.00275.
54. Pressler, SJ, Gradus-Pizlo, I, Chubinski, SD, Smith, G, Wheeler, S, Sloan, R, Jung, M. Family caregivers of patients with heart failure: a longitudinal study. *Journal of Cardiovascular Nursing.* 2013;28(5):417-428. Available from: doi:10.1097/JCN.0b013e3182563877.
55. Saunders, MM. Factors associated with caregiver burden in heart failure family caregivers. *Western journal of nursing research.* 2008;30(8):943-959. Available from: doi: 10.1177/0193945908319990.
56. Saunders, MM. Indicators of health-related quality of life in heart failure family caregivers. *Journal of Community Health Nursing.* 2009;26:173–182. Available from: doi: 10.1080/07370010903259196.
57. Saunders, MM. Working and caregiving a comparison of employed and unemployed caregivers of older heart failure patients. *Holistic Nursing Practice*. 2010;24(1):16-22. Available from: doi:10.1097/HNP.0b013e3181c8e469.

For peer review only - http://bmjopen.bmj.com/site/about/guidelines.xhtml

58. Schwarz, KA, Dunphy, G. An examination of perceived stress in family caregivers of older adults with heart failure. *Experimental Aging Research*. 2003;29(2):221-235. Available from doi:10.1080/03610730303717.
59. Scott, LD. Caregiving and care receiving among a technologically dependent heart failure population. *Advances in Nursing Science.* 2000;23(2):82-97
60. Takata, S, Washio, M, Moriwaki, A, Tsuda, T, Nakayama, H, Iwanaga, T, Inoue, H. Burden among caregivers of patients with chronic obstructive pulmonary disease with long-term oxygen therapy. *International Medical Journal.* 2008;15(1):53-57.
61. Vellone, E, D'Agostino, F, Buck, HG, Fida, R, Spatola, CF, Petruzzo, A, Riegel, B. The key role of caregiver confidence in the caregiver's contribution to self-care in adults with heart failure. *European Journal of Cardiovascular Nursing*. 2015;14(5):372–381. Available from: doi:10.1177/1474515114547649.
62. Woolfe, P, McMillan, M, Conway, J. The needs of caregivers of people with COPD: a study. *Australian Journal of Primary Health.* 2007;13(1):28-35. Available from: doi: 10.2147/COPD.S76091.
63. Andersen, KL, Korneliussen, K, Fagermoen, MS. Family caregivers to a patient with chronic heart failure living at home: «co-workers» in a blurred health care system. *Journal of Cardiovascular Nursing*. 2015;20(3):162-9.
64. Baker, K, Flattery, M, Salyer, J, Haugh, KH, Maltby, M. Caregiving for patients requiring left ventricular assistance device support. *Heart & Lung.* 2010;39(3):196-200. Available from: doi:10.1016/j.hrtlng.2009.07.007.
65. Bove, DG, Zakrisson, AB, Midtgaard, J, Lomborg, K, Overgaard, D. Undefined and unpredictable responsibility: a focus group study of the experiences of informal caregiver spouses of patients with severe COPD. *Journal of Clinical Nursing*. 2016;25:483–493. Available from: doi:10.1111/jocn.13076 483.
66. Burke, RE, Jones, J, Ho, MJ, Bekelman, D. Caregivers' perceived roles in caring for patients with heart failure: what do clinicians need to know? *Journal of Cardiac Failure,* 2014;20 (10):731-738. Available from: doi.org/10.1016/j.cardfail.2014.07.011.
67. Figueiredo, D, Jácome, C, Gabriel, R, Marques, A. Family care in chronic obstructive pulmonary disease: what happens when the carer is a man? *Scandinavian Journal of Caring Sciences.* 2015;30:721-730. Available from: doi: 10.1111/scs.12298.
68. Halm, MA. Specific needs, concerns, strategies and advice of caregivers after coronary artery bypass surgery. *Heart & Lung*. 2016;45:416-422. Available from: doi.org/10.1016/j.hrtlng.2016.07.001.
69. Halm, MA. Age and gender influences on the needs, concerns and strategies of CABG caregivers. *Heart & Lung*. 2017;46:159-165. Available from: doi.org/10.1016/j.hrtlng.2017.01.012.
70. Hynes, G, Stokes, A, McCarron, M. Informal care-giving in advanced chronic obstructive pulmonary disease: lay knowledge and experience. *Journal of Clinical Nursing*. 2012;21:1068-1077. Available from: doi: 10.1111/j.1365-2702.2011.03944.
71. Imes, C, Dougherty, CM, Pyper, G, Sullivan, MD. Care of Patients with Heart Failure Descriptive study of partners' experiences of living with severe heart failure. *Heart & Lung*. 2011;40:208-216. Available from: doi:10.1016/j.hrtlng.2010.12.007.
72. Kitko, LA. The work of spousal caregiving in heart failure. PhD [dissertation]. State College, PA: Pennsylvania State University;2010.
73. Liljeroos, M, Ågren, S, Jaarsma, T, Strömberg, A. Perceived caring needs in patient-partner dyads affected by heart failure: a qualitative study. *Journal of Clinical Nursing.* 2014;23:2928–2938. Available from: doi: 10.1111/jocn.12588.

For peer review only - http://bmjopen.bmj.com/site/about/guidelines.xhtml

74. Lindqvist, G, Albin, B, Heikkila, K Hjelm, K. Conceptions of daily life in women living with a man suffering from chronic obstructive pulmonary disease. Primary Health Care Research & Development. 2012;14:40–51. Available from: doi.org/10.1017/S146342361200031X.
75. Luttik, MLL, Jaarsma, T. Living with heart failure: partner perspectives. *Journal of Cardiovascular Nursing.* 2007;22(2):131-137. Available from: doi:10.1097/00005082-200703000-00010.
76. Marcucilli, L, Casida, J. From insiders' perspectives: adjusting to caregiving for patients with left ventricular assist devices. *Progress in Transplantation.* 2011;21(2):137-43. Available from: doi:10.7182/prtr.21.2.dq53164u1464u683.
77. Marcucilli, L, Casida, J, Bakas, T, Pagani, FD. Family caregivers' inside perspectives: caring for an adult with a left ventricular assist device as a destination therapy. *Progress in Transplantation*. 2014;24(4):332-340. Available from: doi:10.7182/pit2014684.
78. Pattenden, JF, Roberts, H Lewin, HJP. Living with heart failure; patient and carer perspectives. *European Journal of Cardiovascular Nursing.* 2007;6:273-279. Available from: doi:10.1016/j.ejcnurse.2007.01.097.
79. Rolley, J, Smith, J, DiGiacomo, M, Salamonson, Y, Davidson, P. The caregiving role following percutaneous coronary intervention. *Journal of Clinical Nursing*. 2010;20:227–235. Available from: doi: 10.1111/j.1365-2702.2009.03104.
80. Spence, A, Hasson, F, Waldron, M, Kernohan, G, McLaughlin, D, Cochrane, B, Watson, B. Active carers: living with chronic obstructive pulmonary disease. *International Journal of Palliative Nursing*. 2008;14(8):368-372. Available from: doi:10.12968/ijpn.2008.14.8.30771.
81. Ström, A, Andersen, KL, Korneliussen, K, Fagermoen, MS. Being "on the alert" and "a forced volunteer": a qualitative study of the invisible care provided by the next of kin of patients with chronic heart failure. *Journal of Multidisciplinary Healthcare.* 2015;8:271–277. Available from: doi.org/10.2147/JMDH.S82239.
82. Wallin, E, Larsson, IM, Rubertsson, S, Kristoferzon, ML. Relatives' experiences of everyday life six months after hypothermia treatment of a significant other's cardiac arrest. *Journal of Clinical Nursing.* 2013;22:1639–1646. Available from: doi: 10.1111/jocn.12112.
83. Wingham, J, Frost, J, Britten, N, Jolly, K, Greaves, C, Abraham, C, Dalal, H. Needs of caregivers in heart failure management: a qualitative study. *Chronic Illness*. 2015;11(4):304-319. Available from: doi:10.1177/1742395315574765.
84. Näsström, L, Luttik, ML, Idvall, E, Strömberg, A. Exploring partners' perspectives on participation in heart failure home care: a mixed-method design. *Journal of Advanced Nursing.* 2017;73(5):1208–1219. Available from: doi: 10.1111/jan.13216.
85. Clark, AM, Reid, ME, Morrisson, CE, Capewell, S, Murdoch, DL, McMurray, JJ. The complex nature of informal care in home-based heart failure management. Journal of Advanced Nursing. 2008;61(4):373–383. Available from: doi:10.1111/j.1365-2648.2007.04527.x.
86. Hess, AM. *The health literacy, self-care, and medication hassles of patients with heart failure and their caregivers.* Masters [dissertation]. West Lafayette, IN: Purdue University;2009.
87. Kneeshaw, MF, Considine, RM, Jennings, J. Mutuality and preparedness of family caregivers for elderly women after bypass surgery. Applied Nursing Research. 1999;12(3):128-135.
88. Park, EO, Yates, BC, Schumacher, KL, Meza, J, Kosloski, K, Pullen, C. Caregiving demand and difficulty in older adult spousal caregivers after coronary artery bypass surgery. Geriatric Nursing. 2013;34:383-387. Available from: doi.org/10.1016/j.gerinurse.2013.05.007.
89. Riegner, J. *The relationship of role strain, perceived social support, and humor to quality of life among couples experiencing the life-limiting illness of chronic obstructive pulmonary disease.* PhD [dissertation]. Tallahassee, Florida: Florida State University;1996.

For peer review only - http://bmjopen.bmj.com/site/about/guidelines.xhtml

90. Miravitlles, M, María, L, Oliva-Moreno, J, Hidalgo-Vega, Á. Caregivers' burden in patients with COPD. *International Journal of COPD.* 2015;10(1):347—356. Available from: doi.org/10.2147/COPD.S76091.
91. Lum, HD, Lo, D, Hooker, S, Bekelman, D.B. Caregiving in heart failure: relationship quality is associated with caregiver benefit finding and caregiver burden. Heart & Lung. 2014;43:306-310. Available from: doi.org/10.1016/j.hrtlng.2014.05.002.
92. Luttik, ML, Jaarsma, T, Lesman, I, Sanderman, R, Hagedoorn, M. Quality of life in partners of people with congestive heart failure: gender and involvement in care. *Journal of Advanced Nursing.* 2009;65(7):1442–1451. Available from: doi: 10.1111/j.1365-2648.2009.05004.
93. Kang, X, Li, Z, Nolan, MT. Informal caregivers' experiences of caring for patients with chronic heart failure Systematic review and metasynthesis of qualitative studies. 2011; 26(5),386-394.
94. AasbØ, G, Nyheim Solbrække, K, Kristvik, E, Werner, A. Between disruption and continuity: challenges in maintaining the "biographical we" when caring for a partner with a severe chronic illness. *Sociology of health and illness*. 2016; 38(5):782-796. Available from: doi:10.1111/1467-9566.12396.
95. Wingham, J, Frost, J Britten, N. Behind the smile: qualitative study of caregivers' anguish and management responses while caring for someone living with heart failure. *BMJ Open*. 2017; 7:e014126. Available from: doi:10.1136/bmjopen-2016-014126.
96. Lawton, MP, Moss, M, Kleban, MH, Glicksman, A, Rovine, M. A Two-factor model of caregiving appraisal and psychological well-being. *Journal of Gerontology*. 1991; 46(4): 181–P189. Available from: https://doi.org/10.1093/geronj/46.4.P181.
97. Lee, CS Bidwell, JT, Paturzo, M, Alvaro, R, Cocchieri, A, Jaarsma, T, Stromberg, A, Riegel, B, Vellone, E. Patterns of self-care and clinical events in a cohort of adults with heart failure: 1 year follow-up. *Heart & Lung.* 2018; 47(1): 40-46. Available from: doi: 10.1016/j.hrtlng.2017.09.004
98. Nakken, N, Janssen, DJA, van den Bogaart, EHA, Wouters, EFM, Franssen, FME, Vercoulen, JH, Spruit, MA. *European Respiratory Review.* 2015; 24(137):498-504. Available from: doi: 10.1183/16000617.00010114.
99. Feeney Mahoney, D. Vigilance: Evolution and Definition for Caregivers of Family Members with Alzheimer's Disease. Journal of Gerontological Nursing. 2003;29(8):24-30. Available from: https://doi.org/10.3928/0098-9134-20030801-07
100. Buck, HG, Harkness, K, Wion, R, Carroll, SL, Cosman, T, Kaasalainen, S, Arthur, HM. Caregiver's contributions to heart failure self-care: a systematic review. *European Journal of Cardiovascular Nursing*. 2015;14(1):79-89. Available from: doi:10.1177/1474515113518434.
101. Dixon-Woods, M, Agarwal, S, Jones, D, Young, B, Sutton, A. Synthesising qualitative and quantitative evidence: a review of possible methods. *Journal of Health Services Research*. 2005;10(1):45-53
102. Bazely, P. Integrating analyses in mixed methods research. SAGE Publications London. 2018

For peer review only - http://bmjopen.bmj.com/site/about/guidelines.xhtml

For peer review only

Figure Legends

Figure 1: < PRISMA Flow Diagram>

Figure 2: < Conceptual Experiences of Caregivers>

Table Legends

For peer review only - http://bmjopen.bmj.com/site/about/guidelines.xhtml

Table 1: < Narrative Formation (Example: Chung, 2016)>

Table 2: <Summary of included studies>

HF = Heart Failure, COPD = Chronic Obstructive Pulmonary Disease, LVAD = Left Ventricular Assist Device, LVAD DT = Left Ventricular Assist Device Destination Therapy, CAD = Coronary Artery Disease, Cg/cg/cgs = Caregiver/s, Pt/Pts/pts = patient/s, N/S = Not stated, CABG = Coronary Artery Bypass Graft, QOL = Quality of Life, HRQOL = Health related quality of life, ADL = Activities of daily living, *= Same study

Table 3: < Study Characteristics>

CAD – Coronary Artery Disease
HF – Heart Failure
COPD – Chronic Obstructive Pulmonary Disease

*** Caregiver Data based on data collected in 50 studies**
***Patient Data based on data collected in 35 studies**
● There were 57 publications, however there were 54 studies. The following studies utilised the same data but produced two publications: Halm, 2006 & 2007. Saunders, 2008 & 2009. Halm 2016 & 2017.

For peer review only

Table 4(a): < Quality Appraisal – Qualitative Papers>
H = High Quality. 4/ 5 out of 5 quality criteria achieved
M= Medium Quality. 3 out of 5 quality criteria achieved
L= Low Quality. 1 or 2 out of 5 quality criteria achieved
•= same study ***Mixed methods study – qualitative component**

Table 4(b): < Quality Appraisal – Quantitative Papers>

For peer review only - http://bmjopen.bmj.com/site/about/guidelines.xhtml

Study design - CS: cross-sectional design; LS: longitudinal design
Participant sampling – Purp: purposive; Rand: random; Cons: consecutive; Con: convenience; NS; not stated/unclear
Attrition – 20% or less = +1; NS: Not Stated
Outcome measures – V: all validated outcomes; non-V: some or all non-validated; NS: not stated/unclear
Data analysis – MV: multivariate; UV: univariate
•= same study ***Mixed method study – quantitative component**

Overall quality rating
H = High Quality. 4/ 5 out of 5 quality criteria achieved
M= Medium Quality. 3 out of 5 quality criteria achieved
L= Low Quality. 1 or 2 out of 5 quality criteria achieved

Table 5: < Illustrative Quotes of caregiver experience – by concept>

For peer review only

For peer review only - http://bmjopen.bmj.com/site/about/guidelines.xhtml

For peer review only

For peer review only - http://bmjopen.bmj.com/site/about/guidelines.xhtml

For peer review only

For peer review only - http://bmjopen.bmj.com/site/about/guidelines.xhtml

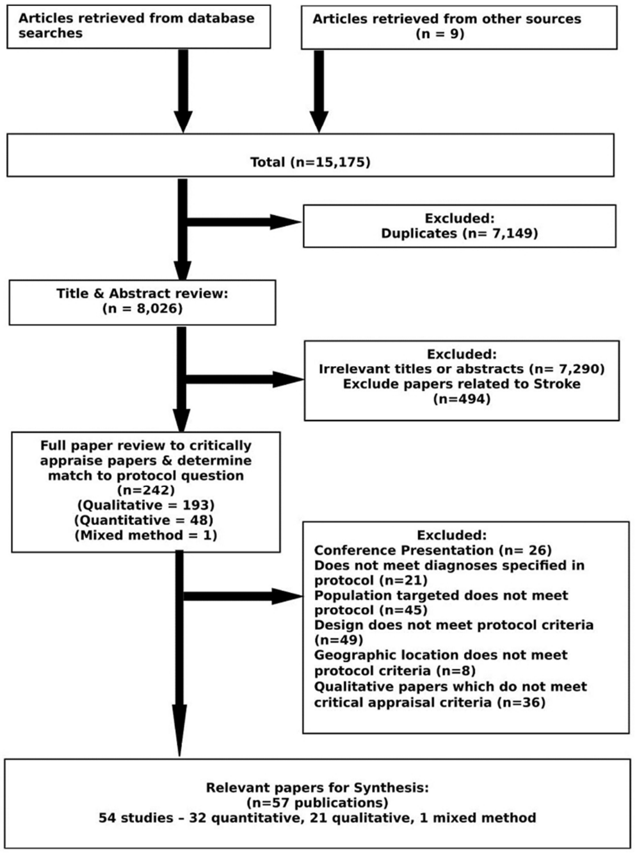

Figure 1 PRISMA

93x131mm (300 x 300 DPI)

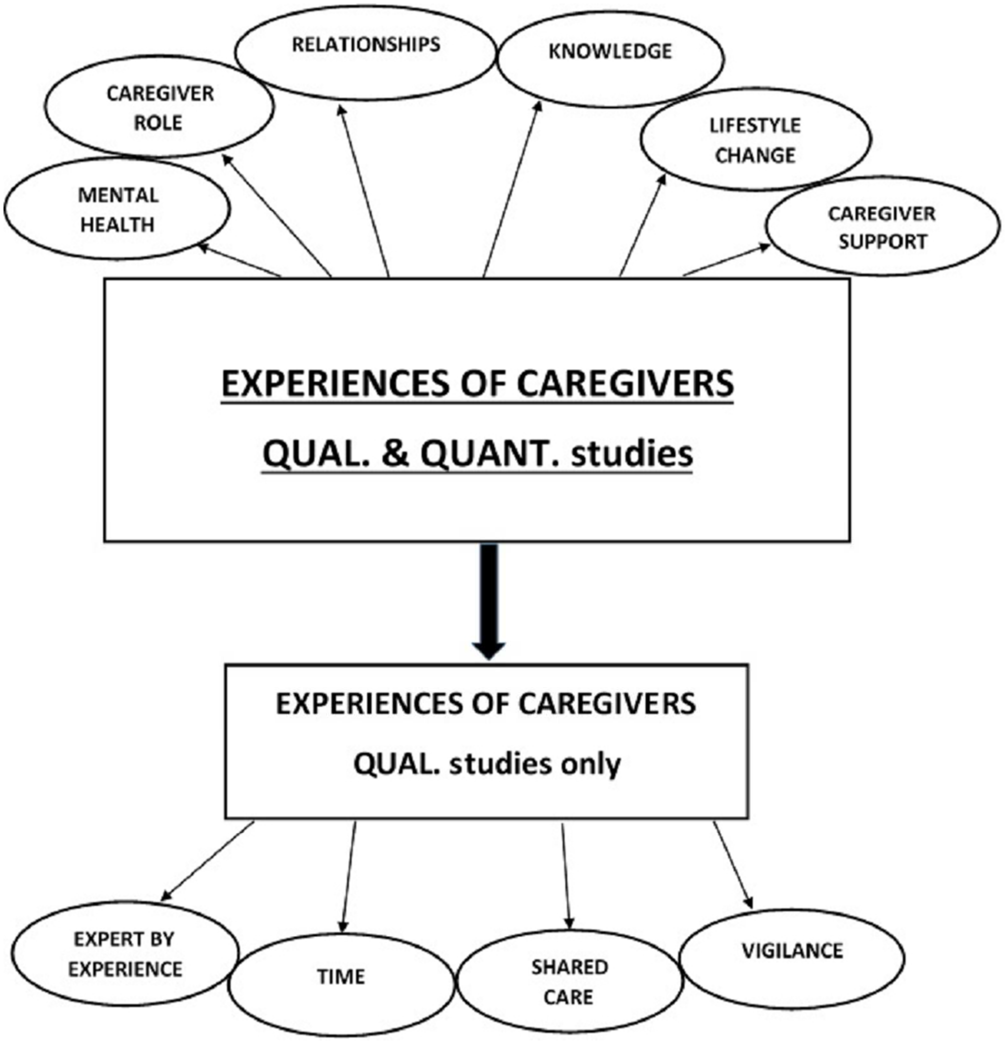

Figure 2 Caregiver Concepts

93x96mm (300 x 300 DPI)

Supplementary:

Table 1: List of search terms

Medline:

MeSH: Caregivers

“Caregivers”.ti,ab OR “care-giver”.ti,ab OR “carer*.ti,ab” OR “informal car*”.ti,ab

AND

MeSH: Quality of life

“Quality of life” OR “outcome measures” OR “caregiver outcomes” OR “care-giver outcomes” OR “time use” OR “time-use” OR “occupations” OR “occupational engagement” OR “self-efficacy” OR “self efficacy” OR “experience” OR “emotion*” OR “psychological health impact” OR “physical health impact” OR “confidence” OR “self-confidence” OR “satisfaction” OR “dissatisfaction” OR “activities of daily living” OR “resilience” OR “social adj2 (interaction or engagement or support) OR “Social adj2 (participation)” OR “caregiver adj2 (support or health professional or medical team or nurse or patient or relationship* or knowledge)” OR “information exchange” OR “coping strategies” OR “leisure activity” OR “conflict” OR “caregiver responsib*” OR care-giver responsib*” OR “caregiver expectation” OR “care-giver expectation” OR “caregiver role” OR “care-giver role” OR “role adjustment” OR “caregiver or care-giver adj2 (attitude to health)”

AND

MeSH: Heart Failure, Heart Diseases, Myocardial Ischaemia, Coronary Artery Disease, Pulmonary Disease, Chronic Obstructive, Pulmonary Heart Disease, Chronic Obstructive Pulmonary Disease, Acute Coronary Syndrome, Cardiovascular Diseases, Coronary Artery Disease, Coronary Artery Bypass, Coronary Disease, Coronary Aneurysm, Stroke, Myocardial Infarction

“Heart Failure”.ti,ab OR “cardiac failure”.ti,ab OR “myocardial failure”.ti,ab OR “left ventricular failure”.ti,ab OR “right ventricular failure”.ti,ab OR “cardiomyopathy”.ti,ab, OR “systolic failure”.ti,ab OR “diastolic failure”.ti,ab OR “Chronic Obstructive Pulmonary Disease”.ti,ab OR “COPD”.ti,ab OR “chronic obstructive lung disease”.ti,ab OR “pulmonary disease”.ti,ab OR “pulmonary disorder”.ti,ab OR “respiratory disease”.ti,ab OR “respiratory disorder”.ti,ab OR “dyspnea”.ti,ab OR “dyspnoea”.ti,ab OR “Stroke”.ti,ab OR “stroke disability”.ti,ab OR “stroke disease”.ti,ab OR “stroke disorder”.ti,ab OR “CVA”.ti,ab OR “cerebrovascular accident”.ti,ab OR “transient ischaemic attack”.ti,ab OR “transient ischemic attack”.ti,ab OR “haemorrhage”.ti,ab OR “hemorrhage”.ti,ab OR “cerebral haemorrhage”.ti,ab OR “cerebral hemorrhage”.ti,ab OR “aneurysm”.ti,ab OR “atrial fibrillation”.ti,ab OR “Coronary Artery Disease”.ti,ab OR “acute coronary syndrome”.ti,ab OR “atherosclerosis”.ti,ab OR “arteriosclerosis”.ti,ab OR “ischaemic heart disease”,ti.ab OR “ischemic heart disease”.ti,ab OR “myocardial infarction”,ti.ab OR “coronary revascularisation”.ti,ab OR “coronary revascularization”.ti,ab OR “angina”.ti,ab OR “CABG”.ti,ab OR “coronary artery bypass graft”.ti,ab OR “stable angina”.ti,ab OR “angina pectoris”.ti,ab

For peer review only

CINAHL:

MeSH: Caregivers, caregiver burden, caregiver supports, caregiver role strain, risk for caregiver role strain, family caregiver status, caregiving endurance potential, caregiver-patient relationship, caregiver well-being, caregiver support, caregiver stressors, caregiver physical health, caregiver performance, caregiver performance: direct care, caregiver performance indirect care, caregiver lifestyle disruption, caregiver emotional health, caregiver strain index, caregiver role strain.

“Caregiver”.ti,ab OR “care-giver”.ti,ab OR “carer*.ti,ab” OR “carer”.ti,ab OR “carers”.ti,ab OR “informal care*”.ti,ab

AND

MeSH: Quality of life

“Quality of life” OR “outcome measures” OR “caregiver outcomes” OR “time use” OR “occupations” OR “occupational engagement” OR “self-efficacy” OR “self efficacy” OR “experience” OR “emotion*” OR “psychological health impact” OR “physical health impact” OR “confidence” OR “self-confidence” OR “satisfaction” OR “dissatisfaction” OR “activities of daily living” OR “resilience” OR “social adj2 (interaction or engagement or support) OR “Social participation” OR “caregiver adj2 (support or health professional or medical team or nurse or patient or relationship* or knowledge)” OR “information exchange” OR “coping strategies” OR “leisure activity” OR “conflict” OR “caregiver responsib*” OR care-giver responsib*” OR “caregiver expectation” OR “care-giver expectation” OR “caregiver role” OR “care-giver role” OR “role adjustment” OR “caregiver or care-giver adj2 (adjustment)” OR “caregiver or care-giver adj2 (acceptance) OR “caregiver or care-giver adj2 (readiness)” OR “caregiver or care-giver adj2 (attitude to health)”

AND

MeSH: Heart Failure, Treatment Failure, Pulmonary Disease, Chronic Obstructive Pulmonary Disease, Chronic Obstructive Pulmonary Disease, Acute Coronary Syndrome, Coronary Arteriosclerosis, Coronary Artery Bypass, Coronary Disease, Coronary Aneurysm, Stroke OR Coronary Artery Disease

“Heart Failure”.ti,ab OR “cardiac failure”.ti,ab OR “myocardial failure”.ti,ab OR “left ventricular failure”.ti,ab OR “right ventricular failure”.ti,ab OR “cardiomyopathy”.ti,ab, OR “systolic failure”.ti,ab OR “diastolic failure”.ti,ab OR “COPD”.ti,ab OR “chronic obstructive lung disease”.ti,ab OR “pulmonary disease”.ti,ab OR “pulmonary disorder”.ti,ab OR “respiratory disease”.ti,ab OR “respiratory disorder”.ti,ab OR “dyspnea”.ti,ab OR “dyspnoea”.ti,ab OR “Stroke”.ti,ab OR “stroke disability”.ti,ab OR “stroke disease”.ti,ab OR “stroke disorder”.ti,ab OR “CVA”.ti,ab OR “cerebrovascular accident”.ti,ab OR “transient ischaemic attack”.ti,ab OR “transient ischemic attack”.ti,ab OR “haemorrhage”.ti,ab OR “hemorrhage”.ti,ab OR “cerebral haemorrhage”.ti,ab OR “cerebral hemorrhage”.ti,ab OR “aneurysm”.ti,ab OR “atrial fibrillation”.ti,ab OR “Coronary Artery Disease”.ti,ab OR “acute coronary syndrome”.ti,ab OR “atherosclerosis”.ti,ab OR “arteriosclerosis”.ti,ab OR “ischaemic heart disease”,ti.ab OR “ischemic heart disease”.ti,ab OR “myocardial infarction”,ti.ab OR “coronary revascularisation”.ti,ab OR “coronary revascularization”.ti,ab OR “angina”.ti,ab OR “CABG”.ti,ab OR “coronary artery bypass graft”.ti,ab OR “stable angina”.ti,ab OR “angina pectoris”.ti,ab

For peer review only

EMBASE:

MeSH: Caregivers

"Caregivers".ti,ab OR "care-givers".ti,ab OR "Caregiver".ti,ab OR "care-giver".ti,ab OR "carer*.ti,ab" OR "informal car*".ti,ab

AND

MeSH: Quality of life

"Quality of life" OR "outcome measures" OR "caregiver outcomes" OR "care-giver outcomes" OR "time use" OR "occupations" OR "occupational engagement" OR ("self-efficacy" OR "self efficacy") OR "experience" OR "emotion*" OR "psychological health impact" OR "physical health impact" OR ("confidence" OR "self-confidence") OR "satisfaction" OR "dissatisfaction" OR "activities of daily living" OR "resilience" OR "social adj2 (interaction or engagement or support) OR "Social participation" OR "caregiver OR care-giver adj2 (support or health professional or medical team or nurse or patient or relationship* or knowledge)" OR "information exchange" OR "coping strategies" OR "leisure activity" OR "conflict" OR "caregiver responsib*" OR care-giver responsib*" OR "caregiver expectation" OR "care-giver expectation" OR "caregiver role" OR "care-giver role" OR "role adjustment" OR "caregiver or care-giver adj2 (adjustment or acceptance or readiness)" OR "caregiver or care-giver adj2 (attitude to health)"

AND

MeSH: Heart Failure, Chronic Obstructive Pulmonary Disease, Bronchitis, Chronic Obstructive, Disease exacerbation, Pulmonary Disease, Cardiovascular Disease, Stroke, Cerebrovascular Accident, Coronary Artery Disease, Ischemic Heart Disease, Coronary Artery Disease, Acute Coronary Syndrome, Heart Infarction, Coronary Aneurysm, Coronary Artery Aneurysm, Coronary Artery Bypass, Coronary Artery Bypass Graft, Coronary arteriosclerosis, Coronary Artery Atherosclerosis

"heart failure",ti,ab OR "cardiac failure".ti,ab OR "myocardial failure".ti,ab OR "left ventricular failure".ti,ab OR "right ventricular failure".ti,ab OR "cardiomyopathy".ti,ab, OR "systolic failure".ti,ab OR "diastolic failure".ti,ab OR "chronic obstructive pulmonary disease" OR "COPD".ti,ab OR "chronic obstructive lung disease".ti,ab OR "pulmonary disease".ti,ab OR "pulmonary disorder".ti,ab OR "respiratory disease".ti,ab OR "respiratory disorder".ti,ab OR "stroke disorder".ti,ab OR "CVA".ti,ab OR "cerebrovascular accident".ti,ab OR "transient ischaemic attack".ti,ab OR "transient ischemic attack".ti,ab OR "haemorrhage".ti,ab OR "hemorrhage".ti,ab OR "cerebral haemorrhage".ti,ab OR "cerebral hemorrhage".ti,ab OR "aneurysm".ti,ab OR "atrial fibrillation".ti,ab OR "Coronary Artery Disease".ti,ab OR "acute coronary syndrome".ti,ab OR "atherosclerosis".ti,ab OR "arteriosclerosis".ti,ab OR "ischaemic heart disease",ti.ab OR "ischemic heart disease".ti,ab OR "myocardial infarction",ti.ab OR "coronary revascularisation".ti,ab OR "coronary revascularization".ti,ab OR "angina".ti,ab OR "CABG".ti,ab OR "coronary artery bypass graft".ti,ab OR "stable angina".ti,ab OR "angina pectoris".ti,ab

For peer review only

PsycInfo:

MeSH: Caregivers

“caregiver”.ti,ab OR “care-giver”.ti,ab OR “carer*.ti,ab” OR “informal car*”.ti,ab

AND

MeSH: Quality of life, Occupations, Well-being, Activities of daily living, Mental Health, Health Behaviour, Emotional Responses, Life satisfaction, Role satisfaction, Resilience, Life Experience, Communication, Strategies, Anxiety, Coping Behaviour, Distress, Recreation, Leisure Time, Participation, Adjustment, Couples

“Quality of life” OR “outcome measures” OR (“caregiver outcomes” OR “care-giver outcomes”) OR (“time-use” OR “time use”) OR “occupations” OR “occupational engagement” OR (“self-efficacy” OR “self efficacy”) OR “experience” OR “emotion*” OR “psychological health impact” OR “physical health impact” OR (“confidence” OR “self-confidence” OR self confidence) OR “satisfaction” OR “dissatisfaction” OR “activities of daily living” OR “resilience” OR “social adj2 (interaction or engagement or support or participation) OR “caregiver OR care-giver adj2 (support or health professional or medical team)” OR “information exchange” OR “coping strategies” OR “leisure activity” OR “conflict” OR “caregiver responsib*” OR care-giver responsib*” OR “caregiver expectation” OR “care-giver expectation” OR “caregiver role” OR “care-giver role” OR “role adjustment” OR “caregiver or care-giver adj2 (adjustment or acceptance or readiness)” OR “caregiver or care-giver adj2 (attitude to health)”

AND

MeSH: Heart Failure, Health Behaviour, Heart Disorders, Cardiovascular Disorders, Self-care skills, Intervention, Treatment Outcomes, Chronic Obstructive Pulmonary Disease, Pulmonary Heart Disease, Stroke, Treatment Outcomes, Intervention, Cerebrovascular Accidents, Coronary Artery Disease, Myocardial Infarctions, Ischemia, Aneurysms

“heart failure”,ti,ab OR “cardiac failure”.ti,ab OR “myocardial failure”.ti,ab OR “left ventricular failure”.ti,ab OR “right ventricular failure”.ti,ab OR “cardiomyopathy”.ti,ab, OR “systolic failure”.ti,ab OR “diastolic failure”.ti,ab OR “chronic obstructive pulmonary disease” OR “COPD”.ti,ab OR “chronic obstructive lung disease”.ti,ab OR “pulmonary disease”.ti,ab OR “pulmonary disorder”.ti,ab OR “respiratory disease”.ti,ab OR “respiratory disorder”.ti,ab OR “dyspnea”.ti,ab OR “dyspnoea”,ti,ab OR “stroke disorder”.ti,ab OR “CVA”.ti,ab OR “cerebrovascular accident”.ti,ab OR “transient ischaemic attack”.ti,ab OR “transient ischemic attack”.ti,ab OR “haemorrhage”.ti,ab OR “hemorrhage”.ti,ab OR “cerebral haemorrhage”.ti,ab OR “cerebral hemorrhage”.ti,ab OR “aneurysm”.ti,ab OR “atrial fibrillation”.ti,ab OR “Coronary Artery Disease”.ti,ab OR “acute coronary syndrome”.ti,ab OR “atherosclerosis”.ti,ab OR “arteriosclerosis”.ti,ab OR “ischaemic heart disease”,ti.ab OR “ischemic heart disease”.ti,ab OR “myocardial infarction”,ti.ab OR “coronary revascularisation”.ti,ab OR “coronary revascularization”.ti,ab OR “angina”.ti,ab OR “CABG”.ti,ab OR “coronary artery bypass graft”.ti,ab OR “stable angina”.ti,ab OR “angina pectoris”.ti,ab

For peer review only

Web of Science

TI = Title

TS = Topic

TI & TS = “caregiver” OR “care-giver” OR “carer*” OR “informal care*”

AND

TS = “Quality of life” OR “outcome measures” OR (“caregiver outcomes” OR “care-giver outcomes”) OR (“time-use” OR “time use”) OR “occupations” OR “occupational engagement” OR (“self-efficacy” OR “self efficacy”) OR “experience” OR “emotion*” OR “psychological health impact” OR “physical health impact” OR (“confidence” OR “self-confidence” OR self confidence) OR “satisfaction” OR “dissatisfaction” OR “activities of daily living” OR “resilience” OR “social adj2 (interaction or engagement or support or participation) OR “caregiver OR care-giver adj2 (support or health professional or medical team)” OR “information exchange” OR “coping strategies” OR “leisure activity” OR “conflict” OR “caregiver responsib*” OR care-giver responsib*” OR “caregiver expectation” OR “care-giver expectation” OR “caregiver role” OR “care-giver role” OR “role adjustment” OR “caregiver or care-giver adj2 (adjustment or acceptance or readiness)” OR “caregiver or care-giver adj2 (attitude to health)”

AND

TI & TS = “heart failure” OR “cardiac failure” OR “myocardial failure” OR “left ventricular failure” OR “right ventricular failure” OR “cardiomyopathy” OR “systolic failure” OR “diastolic failure” OR “chronic obstructive pulmonary disease” OR “COPD” OR “chronic obstructive lung disease” OR “pulmonary disease” OR “pulmonary disorder” OR “respiratory disease” OR “respiratory disorder” OR “dyspnea” OR “dyspnoea” OR “stroke disorder” OR “CVA” OR “cerebrovascular accident” OR “transient ischaemic attack” OR “transient ischemic attack” OR “haemorrhage” OR “hemorrhage” OR “cerebral haemorrhage” OR “cerebral hemorrhage” OR “aneurysm” OR “atrial fibrillation” OR “Coronary Artery Disease” OR “acute coronary syndrome” OR “atherosclerosis” OR “arteriosclerosis” OR “ischaemic heart disease” OR “ischemic heart disease” OR “myocardial infarction” OR “coronary revascularisation” OR “coronary revascularization” OR “angina” OR “CABG” OR “coronary artery bypass graft” OR “stable angina” OR “angina pectoris”

For peer review only

ProQuest: Global Dissertations & Theses/ Applied Social Sciences Index and Abstracts (ASSIA)

“caregiver”.ti,ab OR “care-giver”.ti,ab OR “carer*.ti,ab” OR “informal car*”.ti,ab

AND

“Quality of life” OR “outcome measures” OR (“caregiver outcomes” OR “care-giver outcomes”) OR (“time-use” OR “time use”) OR “occupations” OR “occupational engagement” OR (“self-efficacy” OR “self efficacy”) OR “experience” OR “emotion*” OR “psychological health impact” OR “physical health impact” OR (“confidence” OR “self-confidence” OR self confidence) OR “satisfaction” OR “dissatisfaction” OR “activities of daily living” OR “resilience” OR “social adj2 (interaction or engagement or support or participation) OR “caregiver OR care-giver adj2 (support or health professional or medical team)” OR “information exchange” OR “coping strategies” OR “leisure activity” OR “conflict” OR “caregiver responsib*” OR care-giver responsib*” OR “caregiver expectation” OR “care-giver expectation” OR “caregiver role” OR “care-giver role” OR “role adjustment” OR “caregiver or care-giver adj2 (adjustment or acceptance or readiness)” OR “caregiver or care-giver adj2 (attitude to health)”

AND

“heart failure”,ti,ab OR “cardiac failure”.ti,ab OR “myocardial failure”.ti,ab OR “left ventricular failure”.ti,ab OR “right ventricular failure”.ti,ab OR “cardiomyopathy”.ti,ab, OR “systolic failure”.ti,ab OR “diastolic failure”.ti,ab OR “chronic obstructive pulmonary disease” OR “COPD”.ti,ab OR “chronic obstructive lung disease”.ti,ab OR “pulmonary disease”.ti,ab OR “pulmonary disorder”.ti,ab OR “respiratory disease”.ti,ab OR “respiratory disorder”.ti,ab OR “dyspnea”.ti,ab OR “dyspnoea”,ti,ab OR “stroke disorder”.ti,ab OR “CVA”.ti,ab OR “cerebrovascular accident”.ti,ab OR “transient ischaemic attack”.ti,ab OR “transient ischemic attack”.ti,ab OR “haemorrhage”.ti,ab OR “hemorrhage”.ti,ab OR “cerebral haemorrhage”.ti,ab OR “cerebral hemorrhage”.ti,ab OR “aneurysm”.ti,ab OR “atrial fibrillation”.ti,ab OR “Coronary Artery Disease”.ti,ab OR “acute coronary syndrome”.ti,ab OR “atherosclerosis”.ti,ab OR “arteriosclerosis”.ti,ab OR “ischaemic heart disease”,ti.ab OR “ischemic heart disease”.ti,ab OR “myocardial infarction”,ti.ab OR “coronary revascularisation”.ti,ab OR “coronary revascularization”.ti,ab OR “angina”.ti,ab OR “CABG”.ti,ab OR “coronary artery bypass graft”.ti,ab OR “stable angina”.ti,ab OR “angina pectoris”.ti,ab

For peer review only

Ethos – British Library

CAREGIVER or CARE-GIVER.ti,ab

Searches initially included Heart Failure, Chronic Obstructive Pulmonary Disease, Stroke and Coronary Artery Disease as "AND" terms, however these yielded no results

For peer review only

Table 2 (a)

Caregiver experience - Concepts/Mapping Table – Qualitative Papers

MENTAL HEALTH = 1 **ROLE = 2** **LIFESTYLE CHANGE = 3**
SUPPORT = 4 **KNOWLEDGE/INFORMATION = 5**
RELATIONSHIPS = 6

Concepts only in Qualitative Papers: **EXPERT BY EXPERIENCE = 7** **VIGILANCE = 8**
TIME = 9 **SHARED CARE = 10**

	**1**	**2**	**3**	**4**	**5**	**6**	**7**	**8**	**9**	**10**
Andersen (2015) **(HF)**	✓		✓	✓	✓					✓
Baker (2010) **(HF)**	✓	✓	✓	✓		✓		✓		✓
Bove (2016) **(COPD)**	✓	✓	✓	✓	✓	✓	✓	✓	✓	✓
Burke (2014) **(HF)**	✓	✓	✓	✓	✓	✓	✓	✓		✓
Clark (2008) **(HF)**		✓	✓	✓	✓	✓	✓	✓	✓	✓
Figueiredo (2015) **(COPD)**	✓	✓	✓	✓	✓	✓	✓	✓	✓	
• Halm (2016) **(CAD)**	✓	✓	✓	✓	✓	✓	✓	✓	✓	✓
• Halm (2017) **(CAD)**	✓	✓	✓	✓	✓	✓	✓	✓	✓	✓
Hynes (2012) **(COPD)**	✓	✓	✓	✓	✓	✓	✓	✓	✓	✓
Imes (2011) **(HF)**	✓	✓	✓	✓	✓	✓		✓	✓	✓
Kitko (2010) **(HF)**	✓	✓	✓	✓	✓	✓	✓	✓	✓	✓
Liljeroos (2014) **(HF)**	✓		✓	✓	✓	✓		✓	✓	✓
Lindqvist (2013) **(COPD)**	✓	✓	✓	✓	✓	✓		✓	✓	✓
Luttik (2007) **(HF)**	✓	✓	✓	✓	✓	✓	✓	✓	✓	✓
Marcuccilli (2011) **(HF)**	✓		✓	✓		✓	✓	✓	✓	✓
Marcuccilli (2014) **(HF)**	✓	✓	✓	✓	✓	✓		✓	✓	
Pattenden (2007) **(HF)**	✓	✓	✓	✓		✓		✓		
Rolley (2010) **(CAD)**	✓	✓	✓	✓	✓	✓		✓		
Spence (2008) **(COPD)**	✓	✓	✓	✓	✓	✓	✓	✓	✓	✓

For peer review only

Strom (2015) **(HF)**	✓	✓	✓	✓		✓	✓	✓		✓
Wallin (2013) **(CAD)**	✓	✓	✓	✓	✓	✓			✓	
Wingham (2015) **(HF)**	✓	✓	✓	✓	✓	✓	✓	✓	✓	✓
**Heart Failure**	**12**	**10**	**13**	**13**	**9**	**12**	**7**	**12**	**8**	**11**
**COPD**	**5**	**5**	**5**	**5**	**5**	**5**	**4**	**5**	**5**	**4**
**CAD**	**3**	**3**	**3**	**4**	**3**	**3**	**1**	**2**	**2**	**1**
**Total**	**20**	**18**	**21**	**21**	**17**	**20**	**12**	**19**	**15**	**16**

• = same study: counted as one

For peer review only

Table 2 (b)

Caregiver experience - Concepts/Mapping – Quantitative Papers

MENTAL HEALTH IMPACT = 1 **ROLE = 2** **LIFESTYLE CHANGE = 3**
SUPPORT = 4 **KNOWLEDGE/INFORMATION = 5**
RELATIONSHIPS = 6

	**1**	**2**	**3**	**4**	**5**	**6**
Agren (2010) **(HF)**	✓					✓
Al-Rawashdeh (2017) **(HF)**	✓		✓			✓
Badr (2017) **(COPD)**	✓		✓			✓
Bakas (2006) **(HF)**	✓	✓	✓			
Chung (2016) **(HF)**	✓	✓	✓			
Cossette (1993) **(COPD)**	✓	✓		✓		
Evangelista (2002) **(HF)**	✓					✓
Figueiredo (2013) **(COPD)**	✓		✓			✓
Figueiredo (2014) **(COPD)**	✓					✓
Grigorovich (2017) **(HF)**	✓		✓	✓		
• Halm (2006) **(CAD)**	✓			✓		✓
• Halm (2007) **(CAD)**	✓			✓		✓
Hess (2009) **(HF)**		✓				
Hooley (2005) **(HF)**	✓					✓
Hwang (2011) **(HF)**	✓	✓		✓		✓
Karmilovich (1994) **(HF)**	✓	✓			✓	✓
Kneeshaw (1999) **(CAD)**		✓	✓	✓	✓	✓
Loftus (2004) **(HF)**	✓	✓		✓		✓
Lum (2014) **(HF)**						✓
Luttik (2009) **(HF)**						✓

For peer review only

Miravitlles (2015) **(COPD)**			✓			✓
Nakken (2017) **(COPD)**	✓		✓	✓		✓
Park (2013) **(CAD)**		✓	✓			✓
Pi-Ming Yeh (2012) **(HF)**	✓			✓		
Pressler (2013) **(HF)**	✓	✓	✓			✓
Riegner (1996) **(COPD)**		✓		✓		✓
• Saunders (2008) **(HF)**	✓			✓		✓
• Saunders (2009) **(HF)**	✓			✓		✓
Saunders (2010) **(HF)**	✓	✓				✓
Schwarz (2003) **(HF)**	✓			✓		
Scott (2000) **(HF)**	✓	✓	✓	✓		
Takata (2008) **(COPD)**	✓	✓	✓	✓	✓	
Vellone (2015) **(HF)**	✓				✓	✓
Woolfe (2007) **(COPD)**	✓		✓	✓	✓	
**Heart Failure**	**15**	**9**	**6**	**7**	**2**	**13**
**COPD**	**5**	**3**	**6**	**5**	**2**	**6**
**CAD**	**4**	**2**	**2**	**3**	**1**	**3**
**Mixed Method Study**	**1**					
**Total**	**25**	**14**	**14**	**15**	**5**	**22**

• = same study: counted as one

For peer review only

Table 2 (c)

Caregiver experience - Concepts/Mapping – Mixed Method Papers

MENTAL HEALTH IMPACT = 1 **ROLE = 2** **LIFESTYLE CHANGE = 3**
SUPPORT = 4 **KNOWLEDGE/INFORMATION = 5**
RELATIONSHIPS = 6

	1	2	3	4	5	6
Näsström (2017) **(HF)**	✓			✓	✓	✓
**TOTAL**	**1**			**1**	**1**	1

For peer review only

Table 1

Individual Study Results

First Author/Year	Diagnosis	Outcome Measure	Result/Findings
Ågren (2010)	HF<br>(NYHA II-IV)	Caregiver Burden Scale<br>Short Form (SF)-36<br>Beck Depression Inventory<br>Control Attitude Scale<br>Knowledge Questionnaire<br>Charlson Co-morbidity Index	68% of partners = low level burden<br>30% of partners = medium burden<br>2% of partners = high burden<br>Regression analysis showed poor mental health (p=0.001) and lower perceived control (p=0.001) in partners and poor patient physical health (p=0.001) predicts higher caregiver burden
Al-Rawashdeh (2017)	HF<br>(NYHA I-IV)	Beck Depression Inventory<br>Minnesota Living with HF (modified for caregivers)<br>Patient Health Questionnaire<br>Short-Form 12 Health Survey	Patients whose spouse has higher sleep disturbance have poorer mental well-being ($\beta = -2.19$, $p < 0.05$)<br>Caregiver sleep disturbance significantly correlated with their own physical ($\beta = -4.94$, $p < 0.001$) and mental well-being scores ($\beta = -6.62$, $p < 0.001$)
Andersen (2015)	HF	n/a	1) Involvement, willingness to assume responsibility and desire for knowledge<br>2) Unclear Responsibility and Insufficient Flow of Information<br>3) Available and Competent Supporters
Badr (2017)	COPD	Patient Health Questionnaire<br>COPD Severity Index<br>Activities of Daily Living scale<br>Lubben Social Network Scale<br>Zarit Burden Interview<br>Checklist created for this study re: co-morbidities (patients & caregivers)<br>Smoking status<br>Self-management behaviours – pts.	30% of patients and 20% of caregivers = mild depression<br>30% of patients and 8% of caregivers = moderate to severe depression.<br>Depression levels of patients and caregivers were significantly correlated (r=0.28, P= 0.01)

For peer review only

First Author/Year	Diagnosis	Outcome Measure	Result/Findings
Bakas (2006)	HF<br>(NYHA II-IV)	Control Attitudes Scale<br>Oberst Caregiving Burden Scale<br>Bakas Caregiving Outcomes Scale<br>Medical Outcomes Study<br>General Health	Caregivers reported their lives had changed for the worse due to providing care (M= 3.7, SD =1.5, 1 worst, 7 best<br>Performing household tasks and managing patient behaviours were most difficult, and the caregiver's emotional<br>and financial well-being, time for social activities, and general health had deteriorated. (ρ -0.46, p < 0.05)
Baker (2010)	HF<br>(LVAD in situ)	n/a	1) Commitment
Bove (2016)	COPD<br>(GOLD C&D)	n/a	1) Undefined and unpredictable responsibility
Burke (2014)	HF<br>(NYHA II-IV)	n/a	1) Health Manager/Care Plan Enforcer<br>2) Advocate for Improved Quality of Life<br>3) Expert in the Lived Experience of HF<br>4) Expressions of Role Conflict and Role Strain
Chung (2016)	HF<br>(NYHA II-IV)	Beck Depression Inventory-II<br>Zarit Burden Interview<br>Oberst Caregiving Burden Scale<br>Bakas Caregiving Outcome Scale<br>Medical Outcome Study	42% reported severe burden<br>Caregivers of patients with depressive symptoms had a higher level of burden (25±13 vs 13.5±12 on the ZBI; p<0.001),<br>Caregivers reported worse mental quality of life (46±10 vs 51±10 on the -12v2; p=0.026) than those of patients without depressive symptoms
Clark (2008)	HF<br>(NYHA II – IV)	n/a	1) The sharing of caring<br>2) The basis of care: formal and informal forms of knowledge about CHF<br>3) The activities of informal care: visible and invisible
Cossette (1993)	COPD<br>(GOLD III-V)	The SCL-90 scale<br>The subjective Stress Scale<br>Caregiving tasks index<br>The family/friend support index	Nearly 40% of wives used psychotropic drugs<br>Difficult emotional support significantly predicted somatization (β= 0.26, p ≤ 0.05), depression (β= 0.34, p ≤ 0.01), obsession compulsion (β= 0.43, p ≤ 0.01), anxiety (β= 0.26, p ≤ 0.05), and hostility (β= 0.37, p ≤ 0.01)

First Author/Year	Diagnosis	Outcome Measure	Result/Findings
Evangelista (2002)	HF (NYHA I – IV)	SF-12 (Mental Component Summary Scale)	Caregiver emotional wellbeing associated with patient's emotional wellbeing<br>Caregivers' mental health score: Adjusted R2 = 0 .536, F = 40.299, p = .000<br>Caregivers reported higher mental wellbeing than patients (P < .001, p = 0.018 (patients), p = 0.118).
Figueiredo (2013)	COPD (GOLD I-IV)	Carers Assessment of Managing Index (Portuguese version)<br>International Classification of Functioning Disability and Health	Better physical health perception was associated with an increasing use of problem-solving strategies (r_s = -0.313; p = 0.029)
Figueiredo (2014)	COPD (GOLD I-IV)	International Classification of Functioning Disability and Health<br>Hospital Anxiety and Depression Scale (Portuguese version)<br>Carers' Assessment of Difficulties Index (Portuguese version)	61.9% = caregivers of early COPD patients observed to be anxious<br>70.4% = caregivers of advanced COPD patients observed to be anxious<br>Depression significantly more frequent in caregivers of advanced COPD patients (p = 0.030)<br>Self-rated physical (p = 0.035) and mental health (p = 0.011) worse in caregivers of advanced COPD patients
Figueiredo (2015)	COPD (Mod – Severe)	n/a	1) Meaning<br>2) Challenges and constraints<br>3) Fears and concerns about the future<br>4) Needs<br>5) Positive aspects
Grigorovich (2017)	HF (NYHA II-IV)	Center for Epidemiological Studies-Depression (CES-D)<br>Positive Affect Scale<br>Caregiver Impact Scale<br>Caregiver Assistance Scale<br>Pearlin Mastery Scale<br>Medical Outcomes Study Social, Support Survey, Personal Gain Scale, Brain Impairment Behaviour, MLWHFQ	Caregivers depression remained stable over1 year (mean -0.17 ± SE 0.11, p > 0.1<br>Caregivers depression associated with participation restriction (p< 0.05)<br>Caregivers positive affect remained stable over 1year (0.10 ± 0.10, p > 0.1)<br>Positive affect associated (p<0.05) with caregivers feelings of personal gain and social support

For peer review only

First Author/Year	Diagnosis	Outcome Measure	Result/Findings
*Halm et al. (2006)	CAD	Caregiving Burden Scale<br>Bakas Caregiving Outcomes Scale<br>SF-12 (spouse proxy ratings)<br>Karnofsky Performance Scale<br>Caregiver Competence Scale<br>State-Trait Anxiety Inventory<br>Center for Epidemiologic Studies Depression Scale<br>Mutuality Scale<br>Expressive Support Scale	Higher burden scores were associated with patient's gender (female) (β = .21, p = .02), increased personal gain (β = .29, p = .004), increased caregiver competence (β = .20, p = .05).<br>Lower burden was associated with better health status of the patient (β= -.22, p = .02), higher caregiver mental HRQL (β = -.38, p = .01).<br>caregiver burden was not significantly different at 3, 6, or 12 months post-surgery
*Halm et al. (2007)	CAD	(as above Halm, 2006)	Controlling for caregiver and patient characteristics, caregivers who perceived less mastery (β = 0.45, p = .001) had higher depressive symptoms
*Halm (2016)	CAD	n/a	1) Knowing what I am supposed to be doing<br>2) Managing multiple medications<br>3) Assisting with mobility<br>4) Dealing with unmonitorable symptoms<br>5) Managing poor appetite<br>6) Keeping spirits up<br>7) Navigating memory loss & confusion<br>8) Surviving moodiness<br>9) Dealing with financial matters
*Halm (2017)	CAD	n/a	1) Caregiving work – comfort, monitoring, support & functional
Hess (2009)	HF	Short-Form Test of Functional Health Literacy<br>Self- Care Index of Heart Failure<br>Family Caregiver Medication Administration Hassles (FCMAHS)	Analyses are the individual results for caregiver health literacy and medication medication administration hassles as the study was insufficiently powered<br>80% of caregivers had adequate health literacy<br>Average FCMAHS score was 27.80 (S.D. = 14.74), with a range of 10-44. Higher scores (out of 100) indicate greater medication administration hassle.

For peer review only

First Author/Year	Diagnosis	Outcome Measure	Result/Findings
Hooley (2005)	HF	Minnesota Living with Heat Failure Questionnaire<br>Beck Depression Inventory II<br>Zarit Caregiver Burden Interview	18% of caregivers had high likelihood of clinical depression<br>Significant correlation between: caregiver burden and caregiver depression scores (r=0.61; p<0.0001), and caregiver burden and patient depression scores (r=0.28; p<0.05)
Hwang (2011)	HF<br>(NYHA I – IV)	Dutch Objective Burden Inventory (English version)<br>Medical Outcome Study Social Support Survey<br>Control Attitudes Scale-Revised<br>Caregiver Reaction Assessment<br>Short Form 36 Health Survey<br>Charlson Co-morbidity Index	Lack of family support for caregivers correlated with the patient's NYHA class, caregivers' relationship to the patient, p=0.003), caregivers' perceived control p <.001) and social support (p <.001)<br>impact of caregiving on caregivers' health was associated with time since the patient's last hospital discharge, (p= .008 caregivers' perceived control, (p = .03) and social support (p <.001)
Hynes (2012)	COPD<br>(NYHA III or IV)	n/a	1) Then and Now<br>2) Awareness of the disease and constant state of anxiety<br>3) The Caring Role<br>4) Perceived illness effects: striving for normalisation and symptom burden<br>5) Support<br>6) Contact with healthcare services
Imes (2011)	HF<br>(NYHA III-IV)	n/a	1) My experience of HF in My Loved One<br>2) Experience with Healthcare providers<br>3) Patient's experience of HF as Perceived by the Partner
Karmilovich (1994)	HF<br>(NYHA III OR IV)	Caregiver Demands Scale (physical demands/role alteration)<br>Brief Symptom Inventory	Significant positive correlation between the number of helping behaviours and the level of stress (r = 0.32, p=0.04)<br>Positive correlation between perceived difficulty performing caring activity and level of stress (r = 0.43, p=0.01)<br>Burden score Mean = 15.27 (SD 11.3)

For peer review only

First Author/Year	Diagnosis	Outcome Measure	Result/Findings
Kitko (2010)	HF	n/a	1) The work of providing care<br>2) Work of living with the illness<br>3) The Work of navigating the system<br>4) The work of maintaining self<br>5) The work of managing the household<br>6) The work of vigilance<br>7) The work of normalcy
Kneeshaw (1999)	CAD	Mutuality Scale<br>Preparedness for Caregiving Scale<br>Recovery Inventory<br>OARS activities of daily living	Significant decrease between the mutuality scale means at hospital discharge and at 3 months (p < .05)<br>Preparedness for caring - most common concern of the caregivers was fear of the unknown, especially an emergency
Liljeroos (2014)	HF	n/a	1) Dyads perceive a need for continuous guidance throughout difference phases of the illness trajectory<br>2) Dyads perceive a need to share burden and support with each other and other dyads
Lindqvist (2013)	COPD<br>(Mild to severe)	n/a	(1) Unchanged life situation where no support was needed<br>(2) Socially restricted life and changed roles where support is needed<br>(3) Changes in health where support is needed<br>(4) Changes in the couple's relationship and their need for support.
Loftus (2004)	HF<br>(NYHA II-IV)	Self-reported health status questionnaire<br>State-Trait Anxiety Inventory<br>Pearlin scale of Mastery<br>Rosenberg Self-Esteem Scale<br>Social Support Scale<br>Dyadic Adjustment Scale<br>Caregiver Distress Scale<br>Sense of Competence Questionnaire<br>Centre for Epidemiological Studies depression Scale	Severity of heart failure and level of patient disability predictive of caregiver distress (personal cost, ANCOVA, F=3.94, p<. 05; depression, ANCOVA, F=7.15, p< .05) independent of caregiver neuroticism<br>Satisfaction with social support was a better predictor of distress than a global measure of social support

For peer review only

First Author/Year	Diagnosis	Outcome Measure	Result/Findings
Lum (2014)	HF<br>(NYHA II-IV)	Zarit Burden Inventory<br>Benefit Finding Scale<br>Patient Health Questionnaire	Higher relationship quality associated with less caregiver burden and more caregiver benefit - (r = -0.54, 95% CI: [-0.81, -0.10], P = 0.02)
Luttik (2007)	HF	n/a	1) Changes in life<br>2) Anxiety<br>3) Changes in relationship<br>4) Sexuality<br>5) Coping/Support
Luttik (2009)	HF<br>(NYHA II-IV)	Medical Outcome Study<br>General Health Survey (RAND-36)<br>Dutch Objective Burden Inventory<br>Cantril's Ladder	Partners of people with CHF reported lower general health than partners of healthy persons (F (1,598) = 4.066, P < 0.05)<br>Interaction effect between group (partners of people with CHF vs. individuals living with a healthy partner) and gender for general well-being, F(1,581) = 4.526, P < 0.05, and for perceived health change, F(1,604) = 4.283, P < 0.05
Marcuccilli (2011)	HF –LVAD<br>in situ	n/a	1) Caregiving – a "24/7" Responsibility<br>2) Coping Strategies<br>3) Caregiving satisfaction
Marcuccilli (2014)	HF-LVAD	n/a	1) Advanced Heart Failure is a Life Changing Event<br>2) Self-doubt about LVAD Caregiving Improved with Time<br>3) Lifestyle Adjustments come with Time<br>4) Persistent Worry and Stress<br>5) Caregiving is not a Burden – it's part of life
Miravitlles (2015)	COPD	Survey on Disabilities, Personal Autonomy, and Dependency Situations (National Institute of Statistics in Spain)	35% of caregivers had health problems<br>83% of caregivers had social/leisure problems<br>38% of those of working age had profession related problems

For peer review only

First Author/Year	Diagnosis	Outcome Measure	Result/Findings
Nakken (2017)	COPD	Charlson comorbidity index<br>Dutch relationship questionnaire<br>Care dependency scale<br>Utrecht Coping List<br>Hospital Anxiety and Depression Scale<br>EuroQol-5 Dimensions<br>Medical Outcome Study Social Support<br>Family Appraisal of Caregiving	Depression levels (P=0.261) social support (P=0.222 to 0.897) and caregiver burden (P=0.410 to 0.720) were comparable for male and female caregivers<br>Female caregivers had more symptoms of anxiety (P<0.0001) and worse health status (P=0.001<br>Lower levels of social support were associated with higher levels of caregiver strain<br>No differences in caregiver burden between gender (P=0.401 to 0.724)
Näsström (2017)	HF	Charlson comorbidity index<br>Relatives' perception of quality of care<br>Dutch Objective Burden Inventory<br>EuroQol<br>The Patient Health Questionnaire	1) Adapting to the caring needs and illness trajectory<br>2) Coping with caregiving demands<br>3) Interacting with healthcare providers<br>4) Need for knowledge to comprehend the health situation<br>More information was associated with better perceived health (rho 0.56, P < 0
Park (2013)	CAD	Caregiving Burden Scale	Subscale scores of caregiving demand and caregiving difficulty were 31.1 and 22.9<br>Demanding caregiving activities perceived by caregivers were: providing transportation, additional household tasks, providing emotional support, and two tied for fourth: monitoring symptoms and additional tasks outside the home
Pattenden (2007)	HF	n/a	1) Symptoms, multiple medications, co-morbidities<br>2) Confusion, anxiety and depression<br>3) Adapting life to heart failure<br>4) Changing roles for carers
Pi-Ming Yeh (2012)	HF	JAREL Spiritual Well-being Scale<br>Carers Assessments of Managing Index<br>Care Continuity Scale<br>Caregiver Reaction Assessment<br>Caregivers Esteem Subscale<br>Lack of Family Support Subscale	People expressed medium levels of ADL dependency (Mean = 9.56, SD = 3.93)<br>Higher scores of the older people's ADL dependence (β = 0.47, P < 0.001), lower scores of quality of relationship (β = -0.39, P < 0.01) and higher scores of lack of family support (β = 0.41, P < 0.001) were found to predict significantly greater family caregiver burden

For peer review only

First Author/Year	Diagnosis	Outcome Measure	Result/Findings
Pressler (2013)	HF<br>(NYHA I-IV)	Oberst Caregiving Burden Scale<br>Control Attitudes Scale<br>Patient Health Questionniare-8<br>Brief Symptom Inventory Anxiety Subscale<br>Bakas Caregiving Outcomes Scale<br>Medical Outcomes Study Short Form-12<br>Charlson Comorbidity Index<br>Family Assessment Device	Caregivers perception of life changed - neutral on average and improved from baseline to 4 (P=0.054) and 8 (P= 0.030) months<br>Poor physical and emotional HRQOL at baseline<br>Caregivers of patients with high HF symptoms spent more time on caregiving tasks at 4mo.(P = 0.001) & 8 mo.(P=0.003), perceived greater difficulty with caregiving tasks (baseline P=0.031 & 4 mo. P <0.001) and reported higher bassline anxiety (P=0.019) and poorer physical HRQOL at baseline (P < 0.001) and 4 mo. (P=0.008)
Riegner (1996)	COPD	Caregiver Strain Index<br>Social Support Questionnaire – Short Form<br>Situational Humor Response Questionnaire<br>Coping Humor Scale<br>Life Situation Survey	Role Strain significantly associated with quality of life (β = 0.15, F=11.5, p <.001)<br>For caregivers; social support satisfaction made a significant contribution to quality of life with β= 0.26, F=22.90, p.<.0001, and R^2 of .26
Rolley (2010)	CAD	n/a	1) A gendered approach to health, illness and caring<br>2) Shock, disbelief and the process of adjustment following PCI<br>3) Challenges and changes of the carer-patient relationship<br>4) The needs of the carer: support and information
*Saunders (2009)	HF	CES-D 10<br>Caregiver Reaction Assessment<br>Quality of Life Index	Caregiver burden is associated with lower CG HRQOL; increased burden in support (r=–0.50, p < .01.), finances (r=–0.52 p < .01), schedule (r=–0.62, p < .01) and health (r=–.71, p < .01) = decreased HRQOL
*Saunders (2008)	HF	(as above Saunders, 2009)	76% of caregivers had health problems in past month<br>Highest to lowest burden ranked: schedule (M = 3.20, SD = 1.09), finances (M = 2.39, SD = 1.18), family (M = 2.38, SD = 1.10), and health (M = 2.21, SD = .98).
Saunders (2010)	HF	Centre for Epidemiological Studies Short Depression Scale<br>Quality of Life Index (QLI)	Caregiver depression not difference between employed and unemployed caregivers (t= 1.0, p < 0.05)<br>Employed caregivers perceived higher well-being except for psychological/spiritual domain, well-being (t =-2.9, p<0.01), health/function (t =-2.3, p<0.05), socioeconomic (t = -2.7, p<0.05), family (t =-3.4, P<0.01)

For peer review only

First Author/Year	Diagnosis	Outcome Measure	Result/Findings
Schwarz (2003)	HF	Perceived Stress Scale<br>Center for Epidemiological Stress<br>Studies Depression Scale<br>Inventory of Socially Supportive Behaviours Scale<br>Clinical Assays Gamma Coat<br>Cortisol	The effects model with salivary cortisol was not significant (F=1.9, p=.15).<br>as measured by the PSS, was the only significant variable (t=5.80, p=.000)<br>No significant associations between salivary cortisol and the PSS (F=1.059, p=.384)
Scott (2000)	HF	Caregiver Preparedness Scale<br>Minnesota Living with Health Failure Questionnaire<br>Caregiver Reaction Assessment<br>The Mental Health Inventory-5<br>Quality of Life Index	Caregivers reported being least prepared for stress associated with role (M = 2.11)<br>89% of caregiver scores for mental health were less than age norm<br>55% of the caregivers have to eliminate things from their schedule/interrupt current activities to provide care<br>39% of the caregivers reported constant fatigue
Spence (2008)	COPD (Adv.)	n/a	1) Impact of family caregiving<br>2) Unmet Support Needs<br>3) Carers' perceptions of patients
Strom (2015)	HF	n/a	1) Being on the alert<br>2) Being a forced volunteer
Takata (2008)	COPD	Zarit Burden Interview<br>(Japanese version)<br>Barthel Index	53.3% of caregivers were depressed<br>Caregivers providing longer term care were more burdened<br>HB caregivers want to use social services such as Nursing Home 90.9% v 34.8% (p<0.05)
Vellone (2015)	HF<br>(NYHA I-IV)	Caregiver Contribution to<br>Self-care of heart failure Index	54.2% of caregivers were unable to recognize the signs and symptoms of a heart failure exacerbation quickly or very quickly
Wallin (2013)	CAD	n/a	1) Difficulties managing a changed life situation<br>2) Feeling like I come second<br>3) Feeling new hope for the future
Woolfe (2007)	COPD	Family Caregiver<br>Needs Survey	Information is the most important need for caregivers (M= 6.37, SD = 0.8<br>Important but poorly met needs = information (27.7), psychological (26.5) and personal (25.3)

For peer review only

First Author/Year	Diagnosis	Outcome Measure	Result/Findings
Wingham (2015)	HF	n/a	1) Providing support<br>2) Transition to becoming a caregiver<br>3) Engaging help

For peer review only

HF = Heart Failure, COPD = Chronic Obstructive Pulmonary Disease, LVAD = Left Ventricular Assist Device, LVAD DT = Left Ventricular Assist Device Destination Therapy, CAD = Coronary Artery Disease, n/a = not applicable, PCI = Percutaneous coronary intervention, MLWHFQ = Minnesota Living with Heart Failure Questionnaire

*** = same study**

For peer review only

PRISMA 2009 Checklist

Section/topic	#	Checklist item	Reported on page #
**TITLE**			
Title	1	Identify the report as a systematic review, meta-analysis, or both.	1
**ABSTRACT**			
Structured summary	2	Provide a structured summary including, as applicable: background; objectives; data sources; study eligibility criteria, participants, and interventions; study appraisal and synthesis methods; results; limitations; conclusions and implications of key findings; systematic review registration number.	2
**INTRODUCTION**			
Rationale	3	Describe the rationale for the review in the context of what is already known.	4,5
Objectives	4	Provide an explicit statement of questions being addressed with reference to participants, interventions, comparisons, outcomes, and study design (PICOS).	4,5,6
**METHODS**			
Protocol and registration	5	Indicate if a review protocol exists, if and where it can be accessed (e.g., Web address), and, if available, provide registration information including registration number.	2
Eligibility criteria	6	Specify study characteristics (e.g., PICOS, length of follow-up) and report characteristics (e.g., years considered, language, publication status) used as criteria for eligibility, giving rationale.	5,6
Information sources	7	Describe all information sources (e.g., databases with dates of coverage, contact with study authors to identify additional studies) in the search and date last searched.	5
Search	8	Present full electronic search strategy for at least one database, including any limits used, such that it could be repeated.	5
Study selection	9	State the process for selecting studies (i.e., screening, eligibility, included in systematic review, and, if applicable, included in the meta-analysis).	5-24
Data collection process	10	Describe method of data extraction from reports (e.g., piloted forms, independently, in duplicate) and any processes for obtaining and confirming data from investigators.	6
Data items	11	List and define all variables for which data were sought (e.g., PICOS, funding sources) and any assumptions and simplifications made.	5,6
Risk of bias in individual studies	12	Describe methods used for assessing risk of bias of individual studies (including specification of whether this was done at the study or outcome level), and how this information is to be used in any data synthesis.	5,6,7,28,29,30,31
Summary measures	13	State the principal summary measures (e.g., risk ratio, difference in means).	6,7
Synthesis of results	14	Describe the methods of handling data and combining results of studies, if done, including measures of consistency (e.g., I^2) for each meta-analysis.	6,7

For peer review only

For peer review only - http://bmjopen.bmj.com/site/about/guidelines.xhtml

PRISMA 2009 Checklist

Section/topic	#	Checklist item	Reported on page #
Risk of bias across studies	15	Specify any assessment of risk of bias that may affect the cumulative evidence (e.g., publication bias, selective reporting within studies).	5,6,7,28,29,30,31,40,41
Additional analyses	16	Describe methods of additional analyses (e.g., sensitivity or subgroup analyses, meta-regression), if done, indicating which were pre-specified.	6,7
**RESULTS**			
Study selection	17	Give numbers of studies screened, assessed for eligibility, and included in the review, with reasons for exclusions at each stage, ideally with a flow diagram.	12
Study characteristics	18	For each study, present characteristics for which data were extracted (e.g., study size, PICOS, follow-up period) and provide the citations.	13-24
Risk of bias within studies	19	Present data on risk of bias of each study and, if available, any outcome level assessment (see item 12).	5,6,7,28,29,30,31
Results of individual studies	20	For all outcomes considered (benefits or harms), present, for each study: (a) simple summary data for each intervention group (b) effect estimates and confidence intervals, ideally with a forest plot.	13-24, 26,27
Synthesis of results	21	Present results of each meta-analysis done, including confidence intervals and measures of consistency.	13-24, 32-39
Risk of bias across studies	22	Present results of any assessment of risk of bias across studies (see Item 15).	5,6,7,28,29,30,31,40,41
Additional analysis	23	Give results of additional analyses, if done (e.g., sensitivity or subgroup analyses, meta-regression [see Item 16]).	38,39
**DISCUSSION**			
Summary of evidence	24	Summarize the main findings including the strength of evidence for each main outcome; consider their relevance to key groups (e.g., healthcare providers, users, and policy makers).	35-39,41-42
Limitations	25	Discuss limitations at study and outcome level (e.g., risk of bias), and at review-level (e.g., incomplete retrieval of identified research, reporting bias).	41,42
Conclusions	26	Provide a general interpretation of the results in the context of other evidence, and implications for future research.	42
**FUNDING**			
Funding	27	Describe sources of funding for the systematic review and other support (e.g., supply of data); role of funders for the systematic review.	42

From: Moher D, Liberati A, Tetzlaff J, Altman DG, The PRISMA Group (2009). Preferred Reporting Items for Systematic Reviews and Meta-Analyses: The PRISMA Statement. PLoS Med 6(7): e1000097. doi:10.1371/journal.pmed1000097

For peer review only - http://bmjopen.bmj.com/site/about/guidelines.xhtml
